# A polymorphic helix of a Salmonella needle protein relays signals defining distinct steps in type III secretion

**DOI:** 10.1371/journal.pbio.3000351

**Published:** 2019-07-01

**Authors:** Emily Z. Guo, Daniel C. Desrosiers, Jan Zalesak, James Tolchard, Mélanie Berbon, Birgit Habenstein, Thomas Marlovits, Antoine Loquet, Jorge E. Galán

**Affiliations:** 1 Department of Microbial Pathogenesis, Yale University School of Medicine, New Haven, Connecticut, United States of America; 2 Institute of Molecular Biotechnology (IMBA), Vienna Biocenter (VBC), Vienna, Austria; 3 Institute of Chemistry and Biology of Membranes and Nano-objects, CBMN-CNRS Université de Bordeaux, Pessac, France; 4 Research Institute of Molecular Pathology (IMP), Vienna Biocenter (VBC), Vienna, Austria; 5 Center for Structural Systems Biology (CSSB), University Medical Center Hamburg-Eppendorf (UKE) and German Electron Synchrotron Centre (DESY), Hamburg, Germany; Swiss Federal Institute of Technology Lausanne (EPFL), SWITZERLAND

## Abstract

Type III protein-secretion machines are essential for the interactions of many pathogenic or symbiotic bacterial species with their respective eukaryotic hosts. The core component of these machines is the injectisome, a multiprotein complex that mediates the selection of substrates, their passage through the bacterial envelope, and ultimately their delivery into eukaryotic target cells. The injectisome is composed of a large cytoplasmic complex or sorting platform, a multiring base embedded in the bacterial envelope, and a needle-like filament that protrudes several nanometers from the bacterial surface and is capped at its distal end by the tip complex. A characteristic feature of these machines is that their activity is stimulated by contact with target host cells. The sensing of target cells, thought to be mediated by the distal tip of the needle filament, generates an activating signal that must be transduced to the secretion machine by the needle filament. Here, through a multidisciplinary approach, including solid-state NMR (SSNMR) and cryo electron microscopy (cryo-EM) analyses, we have identified critical residues of the needle filament protein of a *Salmonella* Typhimurium type III secretion system that are involved in the regulation of the activity of the secretion machine. We found that mutations in the needle filament protein result in various specific phenotypes associated with different steps in the type III secretion process. More specifically, these studies reveal an important role for a polymorphic helix of the needle filament protein and the residues that line the lumen of its central channel in the control of type III secretion.

## Abstract 2

Type III secretion machines evolved to inject bacterial effector proteins into eukaryotic cells, becoming active upon bacterial contact with the target cells. Here we provide insight into the mechanism by which the contact signal is transduced to activate the protein secretion machine.

## Introduction

Many pathogenic and symbiotic bacteria use type III secretion systems (T3SSs) to establish infection or take up residence within a desired host [1–3]. These T3SSs inject effector proteins into host cells to subvert cellular functions [4]. *Salmonella enterica* serovar Typhimurium (*S*. Typhimurium), a gram-negative bacterium responsible for innumerable cases of acute gastroenteritis worldwide [5,6], utilizes two T3SSs, encoded within its pathogenicity islands 1 (SPI-1) and 2 (SPI-2), to invade cells and establish an intracellular replicative niche [7,8]. Collectively, these T3SSs secrete >50 effectors capable of modulating a variety of host cell functions.

The SPI-1 T3SS is composed of several substructures that together form an approximately 4-Md nanomachine or injectisome [1,9–12]. The core component of this machine is the envelope-associated needle complex, which consists of a multiring base, an inner rod, and a needle filament extension that protrudes several nanometers from the bacterial surface [9,11,13,14]. The entire structure is traversed by an approximately 2-nm channel that serves as a conduit for the T3SS protein substrates as they pass through the bacterial envelope [11,14–16]. A group of polytopic inner membrane proteins, known as the export apparatus, forms a channel that allows passage of the secreted proteins through the bacterial inner membrane [17]. The needle complex is associated with a large cytoplasmic complex known as the sorting platform, which selects the proteins destined to travel the type III secretion pathway and initiates them into the secretion process [18]. Assembly of the injectisome occurs in a sequential manner, initiated by the assembly of the export apparatus, which templates the assembly of the inner rings of the base substructure of the needle complex, followed by the assembly of the outer rings [19–21]. The fully assembled base recruits the cytoplasmic sorting platform and begins to operate as a protein-secretion machine with a specificity limited to the proteins necessary for the completion of the assembly of the needle complex (early substrates), including the needle (PrgI) and inner rod (PrgJ) proteins and the regulatory proteins InvJ and OrgC [16,22,23]. The fully assembled injectisome switches substrate specificity, becoming competent for the secretion of the needle tip (SipD) and translocase (SipB and SipC) proteins (middle substrates), which mediate the passage of the effectors (late substrates) through the target cell plasma membrane.

A distinctive feature of T3SSs is the requirement of an activation signal prior to the secretion of protein translocases and effectors [24,25]. In many T3SSs, the activating signal is derived from their contact with target eukaryotic cells. Although this aspect of T3SS function is poorly understood, it is thought to involve a sensing step likely mediated by the tip protein complex [24–28]. This sensing step is thought to be followed by a signal transduction process that is initiated by the needle filament and must be subsequently conveyed to the inner rod, export apparatus, and cytoplasmic sorting platform substructures. The mechanisms involved in these steps are not understood but are likely to involve defined conformational changes in the different components of the injectisome. Consistent with this hypothesis, previous studies have identified mutations in the needle filaments of *Shigella* [29] and *Yersinia* [30,31] and the inner rod of *S*. Typhimurium [32], which lead to altered secretion patterns.

Studies involving protein crystallography, solution- and solid-state NMR (SSNMR), and rosetta modeling have provided major insight into the structure of the 80–amino acid *Salmonella* needle filament protein PrgI, both in solution as well as within the polymerized needle structure [12,33–36]. After the original submission of this manuscript, a cryo-EM structure of isolated, in vivo assembled needle filaments have also become available [37]. However, no structure of the needle filament attached to the base has been reported. In solution, PrgI adopts a helix-loop-helix structure with a P-X-X-P motif defining the loop segment and a largely unstructured N-terminus. Unlike the structure in solution, in the polymerized needle filament, the N-terminus of PrgI adopts a defined configuration that engages in an extensive set of intra- and intermolecular interactions. In the middle of the N-terminal α-helix, three residues (V20–N22) define a pronounced kink that may be important for signal transduction. Furthermore, four residues at the end of the C-terminal α-helix, three charged (K66, D70, and R80) and one polar uncharged (Q77), form the lumen of the channel and create an electrostatic surface potential of alternating charges, which spiral upwardly about the needle [12], a feature that has been proposed to be important for substrate translocation [1].

In this study, we have carried out an extensive mutagenesis analysis of the *S*. Typhimurium needle filament protein PrgI. Coupled with SSNMR and cryo-EM analysis of wild-type and mutant proteins, this study identified various altered secretion phenotypes that implicate a polymorphic helix in the C-terminus of the needle filament protein and the residues that line the lumen of its central channel in controlling signal transduction and reprogramming in the type III secretion machine.

## Results

### Most PrgI mutants with altered secretion phenotype clustered within its C-terminus

**Fig 1:**
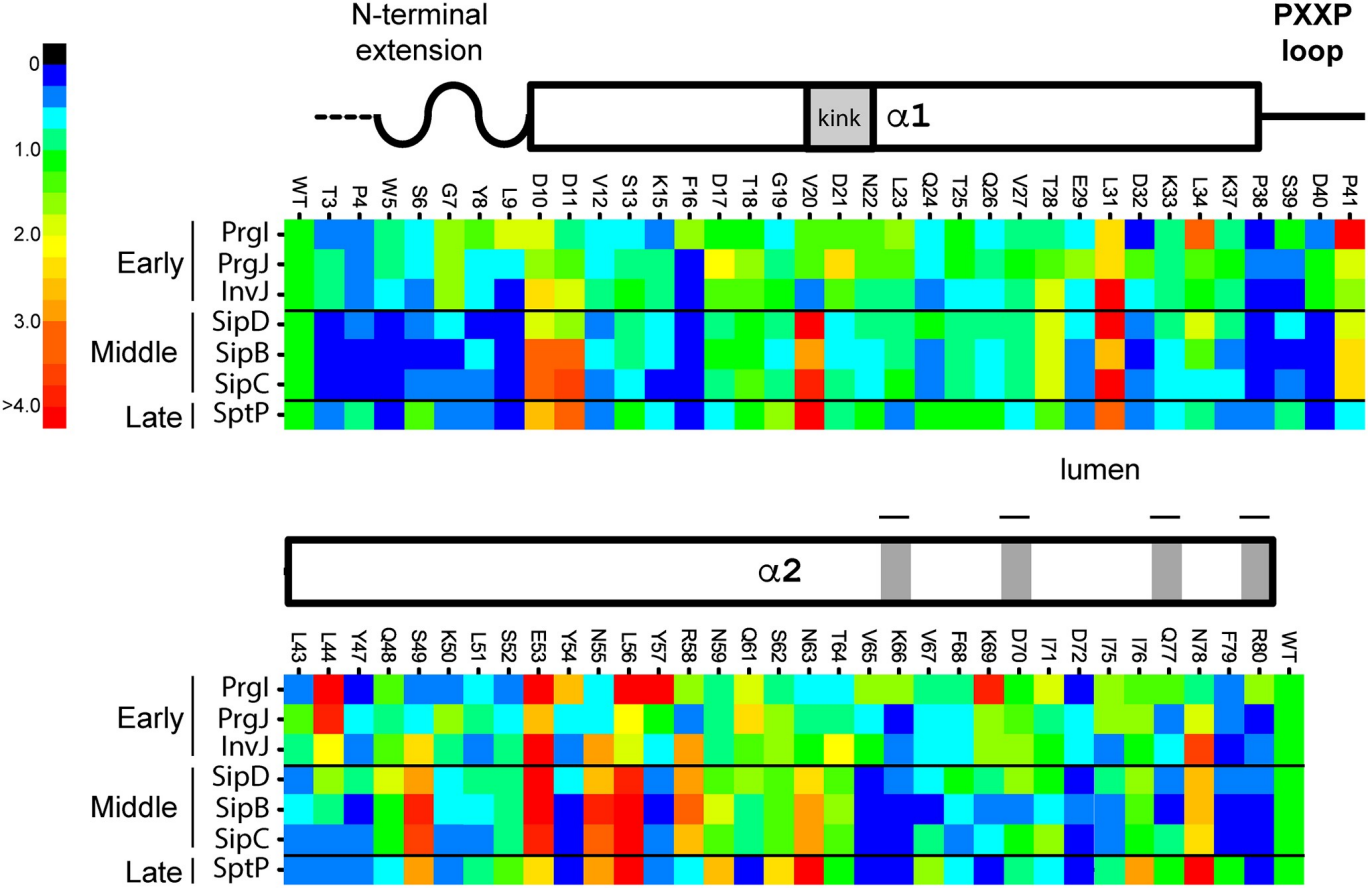
The majority of PrgI mutants with a secretion phenotype cluster at its C-terminus. Heat map showing the secretion profiles of all 68 alanine mutants juxtaposed with the secondary structural elements of PrgI. Individual mutants starting with T3A at the N-terminus (left-hand side) and ending with R80A at the C-terminus (right-hand side) are designated on top of the heat map, while the respective relative quantities of secreted early, middle, and late substrates are represented in columnar fashion from top to bottom, as indicated on the left-hand side. The relative quantities were normalized to WT, which equals a value of 1. The heat map only reflects the mean of three independent experiments. The color code for these values is shown on the far left-hand side. A WT level of secretion is represented as green. The numerical data from which the heat map was derived are shown in S2 Fig. The underlying data for this figure can be found in S1 Data. WT, wild-type.

To survey the function of the *Salmonella* SPI-1 injectisome needle substructure, an alanine-scanning mutagenesis (ASM) approach of its subunit protein PrgI was combined with a quantitative immunoblotting assay to identify mutants with altered ability to secrete early (InvJ, PrgJ, and PrgI) [22,25,38], middle (SipD, SipB, and SipC) [39,40], or late (SptP) [41] substrates. A total of 68 nonalanine positions out of 80 were targeted for ASM. Of all the targeted mutants assayed, 19 displayed altered secretion, of which 12 (approximately 65%) lay within a region of the C-terminal α-helix spanning positions 49–80 (Fig 1, S1 and S2 Figs, and Table 1), suggesting a critical functional role for the C-terminus of PrgI in needle function. The mutagenesis analysis identified three major secretion phenotypes: 1) mutants displaying enhanced secretion of middle and late substrates, 2) mutants able to secrete only early substrates, and 3) mutants displaying a selective deficiency in the secretion of only the middle substrate SipB. The stability and biochemical behavior of all the purified mutant proteins that displayed a secretion phenotype was equivalent to that of the wild-type PrgI, suggesting that the mutations did not result in gross conformational changes (S3 Fig).

**Table 1:** List of phenotypes and alanine mutations identified from screen.

Secretion Phenotype	Mutation
Constitutive	D10, D11, V20, L31, S49, E53, N55, L56, R58, N63, and N78
Early substrates only	T3, L9, F16, and V65
SipB deficient	V67, D70, I75, and Q77

### Constitutive secretion mutants highlight signal transduction relay points in the interface between the needle filament and the SipD tip structure

**Fig 2:**
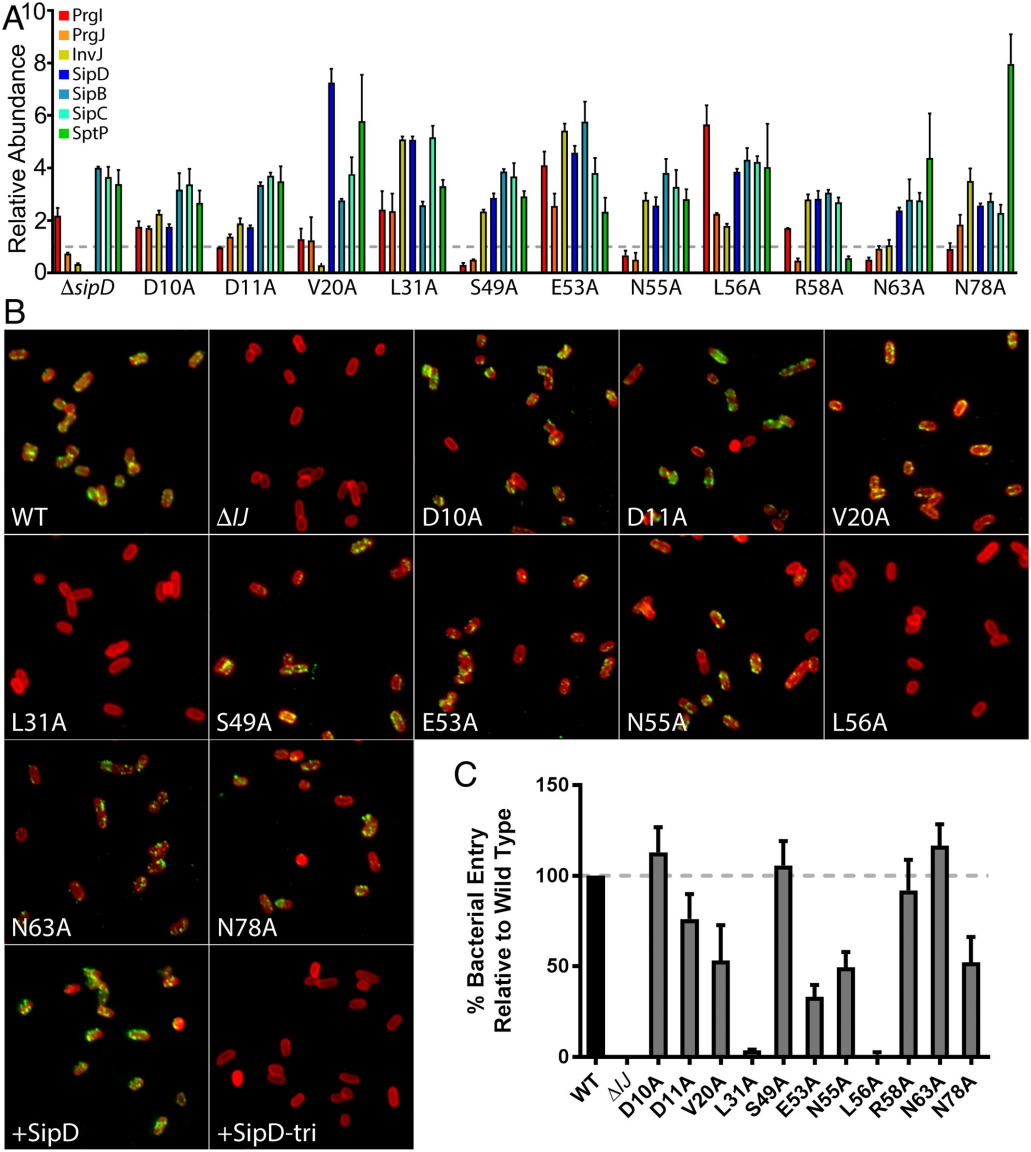
Constitutive secretion mutants highlight signal relay point with the SipD tip structure. (A) Bar graph detailing the secretion phenotypes of *S*. Typhimurium expressing the indicated PrgI constitutive mutant strains compared to the secretion profile of a Δ*sipD S*. Typhimurium strain. The relative abundance of the secreted substrates has been standardized relative to the values of the WT strains, which was given a value of 1 and is demarcated by a gray dashed line. All values represent the mean ± the standard deviation of three independent experiments. The assay data of the constitutive secretion mutants were compiled from the data presented in S2 Fig. The underlying data for this panel can be found in S2 Data. (B) Fluorescence microscopy images of intact, nonpermeabilized *Salmonella* expressing FLAG-tagged SipD probed for SipD binding to the needle of the T3SS with antibodies specific for lipopolysaccharide (red) and the FLAG-tag epitope (green). Shown is the *ΔprgI ΔprgJ S*. Typhimurium strain complemented with WT PrgI and PrgJ, an empty vector (Δ*IJ*), or the indicated constitutive PrgI mutants (along with WT PrgJ). SipD+ and SipD-tri represent controls, in which a Δ*sipD* strain was complemented with FLAG-tagged WT SipD (SipD+) or a triple mutant (SipD^D320R/D323K/S327R^) incapable of binding to the needle substructure (SipD-tri). (C) Cultured epithelial cell invasion ability of *S*. Typhimurium strains expressing the indicated constitutive PrgI mutants. Numbers represent the percentage of the inoculum that survived antibiotic treatment due to internalization and are the mean ± standard deviation of three independent experiments normalized to WT, which was set to 100%. The underlying data for this panel can be found in S3 Data. T3SS, type III secretion system; WT, wild-type.

A total of 11 PrgI mutants displayed a distinct phenotype characterized by uncontrolled and/or heightened secretion of substrates (Fig 2A). Four of the mutants (D10A, D11A, V20A, and L31A) mapped to the N-terminus, while seven (S49A, E53A, N55A, L56A, R58A, N63A, and N78A) were located within the region spanning the C-terminal α-helix. Upon sensing some aspect of the host cell, the tip complex is thought to undergo conformational changes signaling secretion activation [42–44], which is presumably transmitted to the secretion apparatus through the needle structure. By unknown mechanisms, the absence of the tip complex protein SipD causes the needle to adopt a conformation that results in the heightened secretion of middle and late substrates [24,39]. Therefore, using an immunofluorescence microscopy-based assay [45], we investigated whether any of the mutants exhibiting constitutive secretion display this phenotype because of their inability to bind SipD. We found that *S*. Typhimurium strains expressing the PrgI mutants D10A, D11A, V20A, S49A, E53A, N55A, R58A, N63A, and N78A retained the ability to display SipD at the tip of the needle filament (Fig 2B). In contrast, strains expressing the L31A and L56A mutants did not (Fig 2B). As expected, SipD was absent from *S*. Typhimurium strains lacking the needle filament *(ΔprgI ΔprgJ*) or expressing a mutant form of SipD (SipD^D320R/D323K/S327R^) previously shown to be unable to bind PrgI [46]. Moreover, bacterial invasion assays, a measure of the SPI-1 T3SS function [47], corroborated these results by showing that the mutants that retain the ability to bind SipD invaded mammalian culture cells, whereas mutants that did not were noninvasive (Fig 2C). These results suggest that D10A, D11A, V20A, S49A, E53A, N55A, R58A, N63A, and N78A impart upon the needle and the T3SS an active conformation that does not disrupt the ability of the tip complex to form. In contrast, L31A and L56A impart upon the needle and the T3SS an active conformation by hindering the formation of the tip complex, thus defining interaction domains of PrgI with SipD. Our results are consistent with previous NMR protein–protein interaction analyses, which broadly defined the interface between PrgI and SipD [46]. These studies revealed that two sides of PrgI, which are involved in lateral interactions within the needle polymer, are involved in its interaction with SipD, such that the spaces between the protomers at the apical end of the needle allow the tip protein to interdigitate between them. Our results, combined with the NMR observations, suggest that one face of PrgI (*i* + 5) linked to SipD could comprise D10, L31, N55, and N63, while the opposite face (*i* + 6) could comprise D11, V20, S49, E53, L56, R58, and N78 (S4 Fig). These positions in PrgI possibly represent signal transduction relay points, which receive the conformational changes of the tip structure upon activation following contact with host cells.

### A subset of PrgI mutants exhibiting enhanced secretion reveals interaction networks relevant for in vivo needle stability

**Fig 3:**
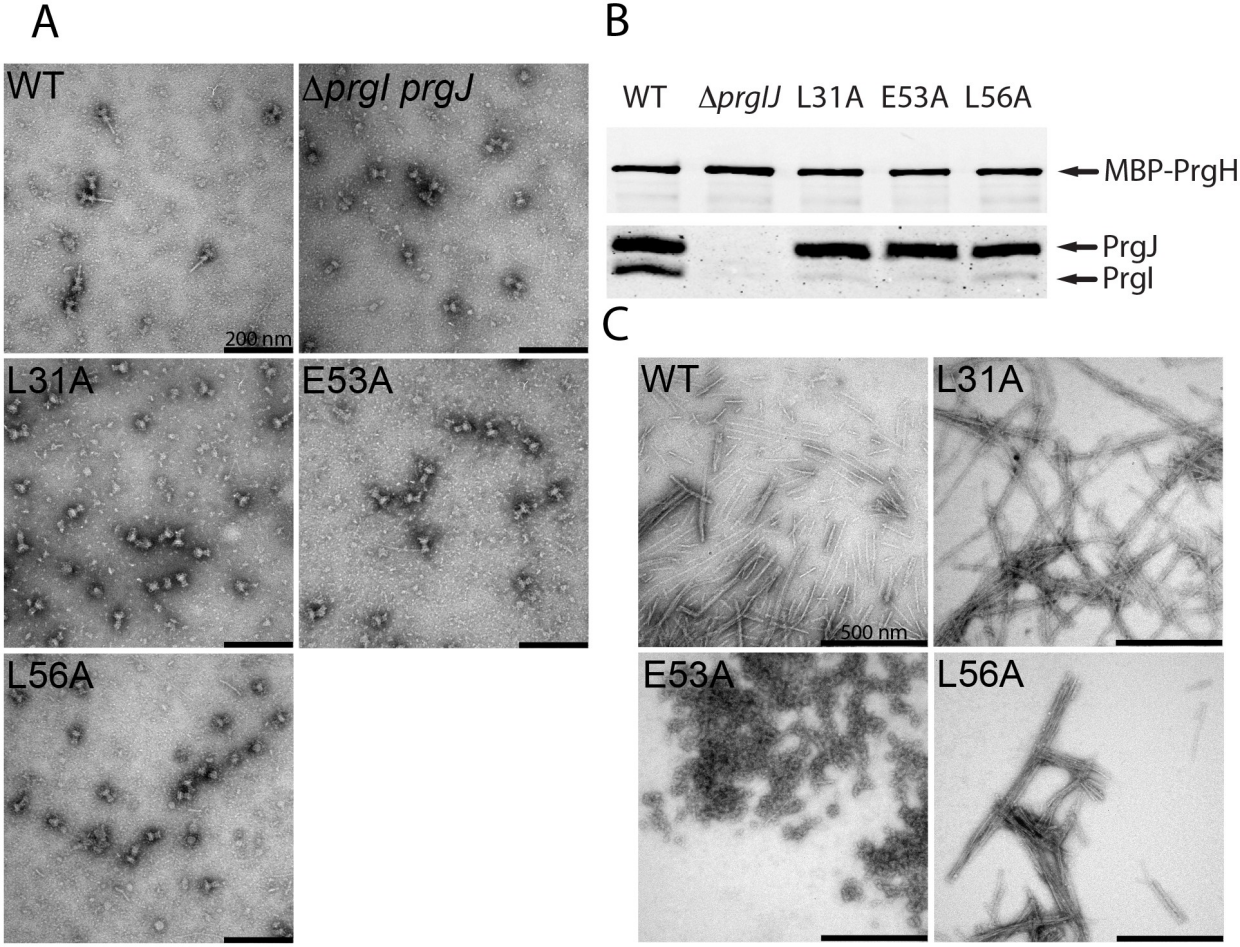
A subset of mutants exhibiting enhanced secretion reveals interaction networks relevant for in vivo needle assembly and stability. (A) Electron micrographs of needle complexes isolated by affinity purification from the indicated *S*. Typhimurium strains. (B) Immunoblot analyses of the corresponding needle complexes probing for PrgI and PrgJ. The loading of the samples was standardized based on the amount of the base component PrgH from the WT strain. (C) Electron micrographs of purified recombinant WT PrgI and the L31A, E53A, and L56A constitutive mutants following in vitro polymerization. WT, wild-type.

A subset of the PrgI mutants exhibiting an increased secretion phenotype (L31A, L56A, and E53A) were distinct in that, in contrast to the *ΔsipD* or other constitutive mutants, their culture supernatants exhibited increased levels of not only middle (translocases) or late (effector) substrates but also the early substrates PrgI, PrgJ, and InvJ (Fig 2A). These observations may reflect defects in needle stability that may lead to the shedding of needle and inner rod subunits to the culture supernatant. Consistent with this hypothesis, two of the three mutants (L56A and L31A) did not display SipD on the bacterial surface (Fig 2B). We isolated needle complexes from *S*. Typhimurium strains expressing the PrgI mutants L31A, L56A, and E53A and examined them by negative staining and electron microscopy. Consistent with the hypothesis that these mutations affect needle stability, we found that needle complexes obtained from these mutants lack needle filaments (Fig 3A). Immunoblot analyses of these preparations detected the presence of wild-type levels of the inner rod protein PrgJ, indicating the presence of an assembled inner rod substructure in these needle complexes. In contrast, this analysis revealed the presence of little to no needle filament protein PrgI (Fig 3B), an observation consistent with the lack of observable needle filaments by electron microscopy (Fig 3A). To gain further insight into the phenotype of these mutants, we explored their ability to polymerize in vitro. The different PrgI mutants were recombinantly expressed, purified, polymerized in vitro, and examined under the electron microscope after negative staining. We found that the PrgI^E53A^ preparation did not yield assembled filaments, appearing as amorphous aggregates. In contrast, PrgI^L31A^ and PrgI^L56A^ were able to polymerize into filaments in a manner and appearance indistinguishable from wild type (Fig 3C). A complete absence of needle filament results in lack of type III secretion [22], presumably because without the needle filament, the outer ring may be closed and substrates may not be able to cross the outer membrane. In addition, in the absence of the needle substructure, substrate switching does not occur and therefore middle (translocases) and late (effectors) substrates cannot be secreted. Consequently, since T3SSs assembled with PrgI^L31A^ and PrgI^L56A^ are able to secrete all substrates (Fig 2A), it follows that these mutants must be able to form a filament structure in vivo that must be able to facilitate the passage of substrates through the outer membrane. Importantly, the needle filament must be able to signal the secretion machine for substrate switching since these mutants are able to secrete middle and late substrates. We hypothesize that these mutants are able to assemble into needles in vivo but the stability of the filament may be compromised, resulting in the shedding of the subunits in vivo (i.e., increased secretion of PrgI and PrgJ; see Fig 2A) and the complete disassembly of the filament during isolation. The structure of the needle filament indicates that L31 and L56 engage in various intra- and intermolecular interactions [12]. For example, L31 forms a prominent hydrophobic intramolecular interaction with Y47 between the N- and C-terminal α-helices, and L56 forms a local network of intermolecular interactions between adjacent C-terminal α-helices with K69, I71, D72, and I76. The alteration of any of these interaction networks may therefore interfere with in vivo needle stability and signal transduction.

### Mutants with an early substrates-only phenotype reveal PrgI determinants for in vivo needle filament assembly and stability

**Fig 4:**
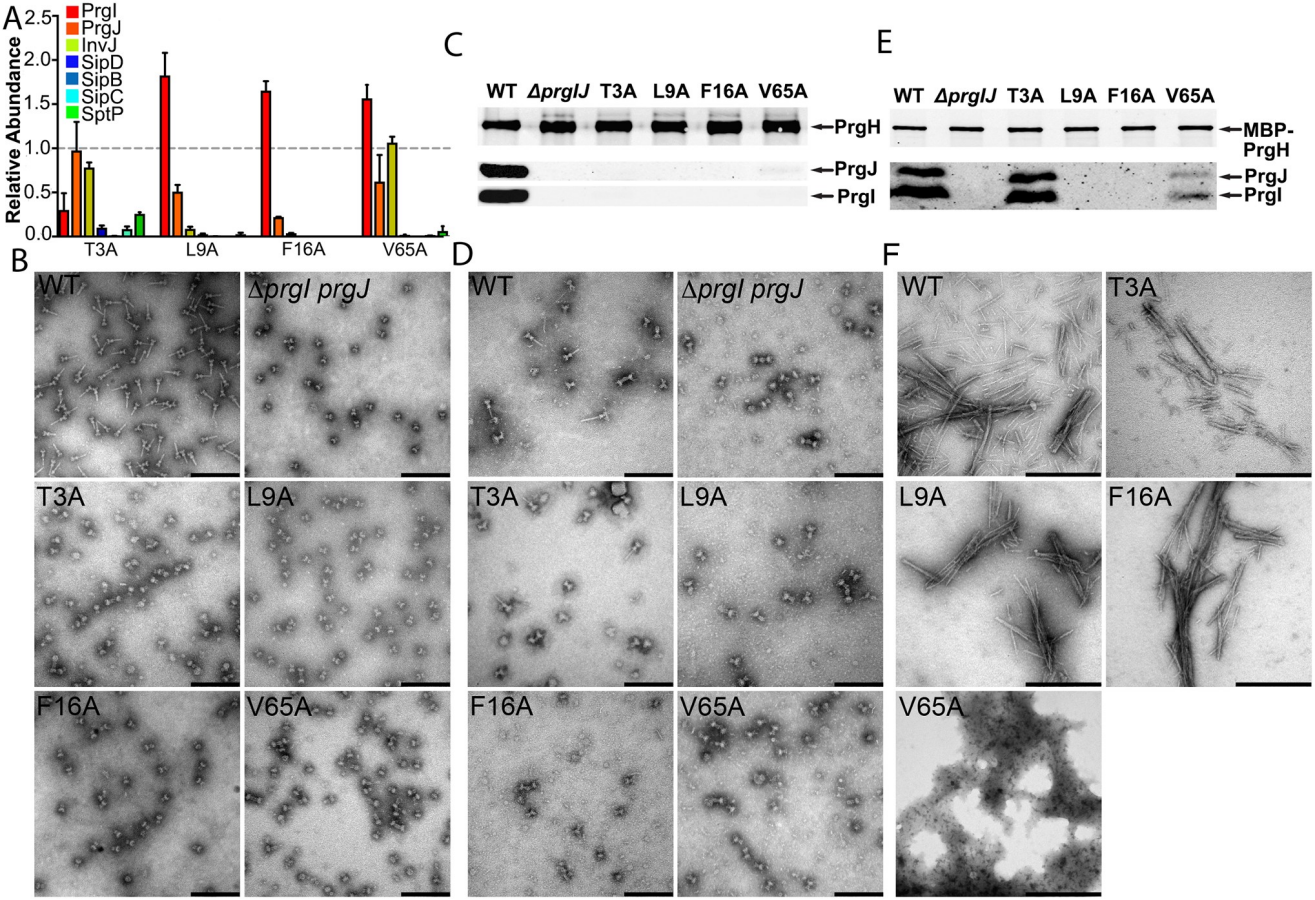
Mutants with an early substrates–only phenotype revealed PrgI determinants for in vivo stability of the needle filament. (A) Bar graph detailing the secretion phenotypes of *S*. Typhimurium expressing the indicated PrgI mutants. The relative abundance of the secreted substrates has been standardized relative to WT, which was given a value of 1 and is demarcated by a gray dashed line. All values represent the mean ± the standard deviation of three independent experiments. The data was compiled from the data presented in S2 Fig. The underlying data for this panel can be found in S4 Data. (B–E) Electron micrographs (B and D) and western blot analysis (C and E) of needle complexes isolated by isopycnic ultracentrifugation (B and C) or by affinity purification (D and E). Samples for the western blot analysis were standardized based on the amount of PrgH. Scale bars in B and D: 200 nm; scale bars in E: 500 nm. (F) Electron micrographs of purified recombinant WT PrgI and the T3A, L9A, F16A, and V65A early substrates–only mutants following in vitro polymerization. WT, wild-type.

Four PrgI mutants (T3A, L9A, F16A, and V65A) identified in our ASM screen can only secrete early substrates (InvJ, PrgJ, and PrgI; Fig 4A). This secretion pattern suggests that the T3SS is “locked” in a state unable to undergo substrate switching and, as a result, cannot secrete middle and late substrates. Alternatively, it is possible that the phenotype observed is the result of defective needle assembly and that the inability to build the needle substructure prevents substrate switching. To distinguish between these possibilities, we examined the needle complex structures of these mutant strains by electron microscopy using two isolation protocols that when used in wild-type bacteria, yield fully assembled needle complexes: a more stringent protocol involving harsher conditions and a milder protocol in which needle complexes are isolated by affinity purification [48] (see Materials and methods for details). We found that needle complexes from the L9A, F16A, and V65A isolated with the harsher or milder protocols did not yield needle substructures, indicating that most likely, these needle filaments did not assemble in vivo. Consistent with this observation, immunoblotting analysis indicated that the base structures isolated under either condition lacked inner rod and needle structures (Fig 4C and 4E). Furthermore, these mutants were unable to enter cultured epithelial cells, a measure of T3SS function. To get further insight into the phenotype of these mutants, we examined the ability of the purified proteins to assemble into filaments in vitro. We found that the V65A mutant was unable to form needle filaments in vitro, indicating that the mutation must affect needle filament assembly. However, both the L9A and F16A mutants were able to assemble filaments in a manner indistinguishable from wild type. In vitro needle filament polymerization differs significantly from in vivo polymerization in that prior to their addition to the growing end, subunits do not need to transit through the nascent filament channel [35,49]. Consequently, these results suggest that PrgI^L9A^ and PrgI^F16A^ are unable to assemble into needle filaments in vivo, thus preventing substrate switching and the secretion of middle and late substrates.

In contrast to the phenotype of the L9A, F16A, and V65A mutants, needle complexes from the T3A mutant did not show needle filaments when isolated using the harsher isolation protocol but showed needle complexes with needle substructure associated to the base when isolated under milder conditions (Fig 4B). Immunoblotting analysis of these preparations showed the presence of the inner rod and needle proteins in samples obtained with the milder isolation protocol but not in samples obtained with the harsher protocol (Fig 4C and 4E). In addition, purified PrgI^T3A^ was able to assemble into needle filaments in vitro. Furthermore, an *S*. Typhimurium strain expressing this mutant exhibited a reduced but still significant ability to invade cultured epithelial cells (S5 Fig). These observations suggest that the T3A mutant is able to assemble into needle filaments in vivo, but the stability of the filaments may be compromised, resulting in inefficient substrate switching.

### Secretion defects associated with C-terminal residues reveals a complex role for the lumen of the needle filament in type III secretion

**Fig 5:**
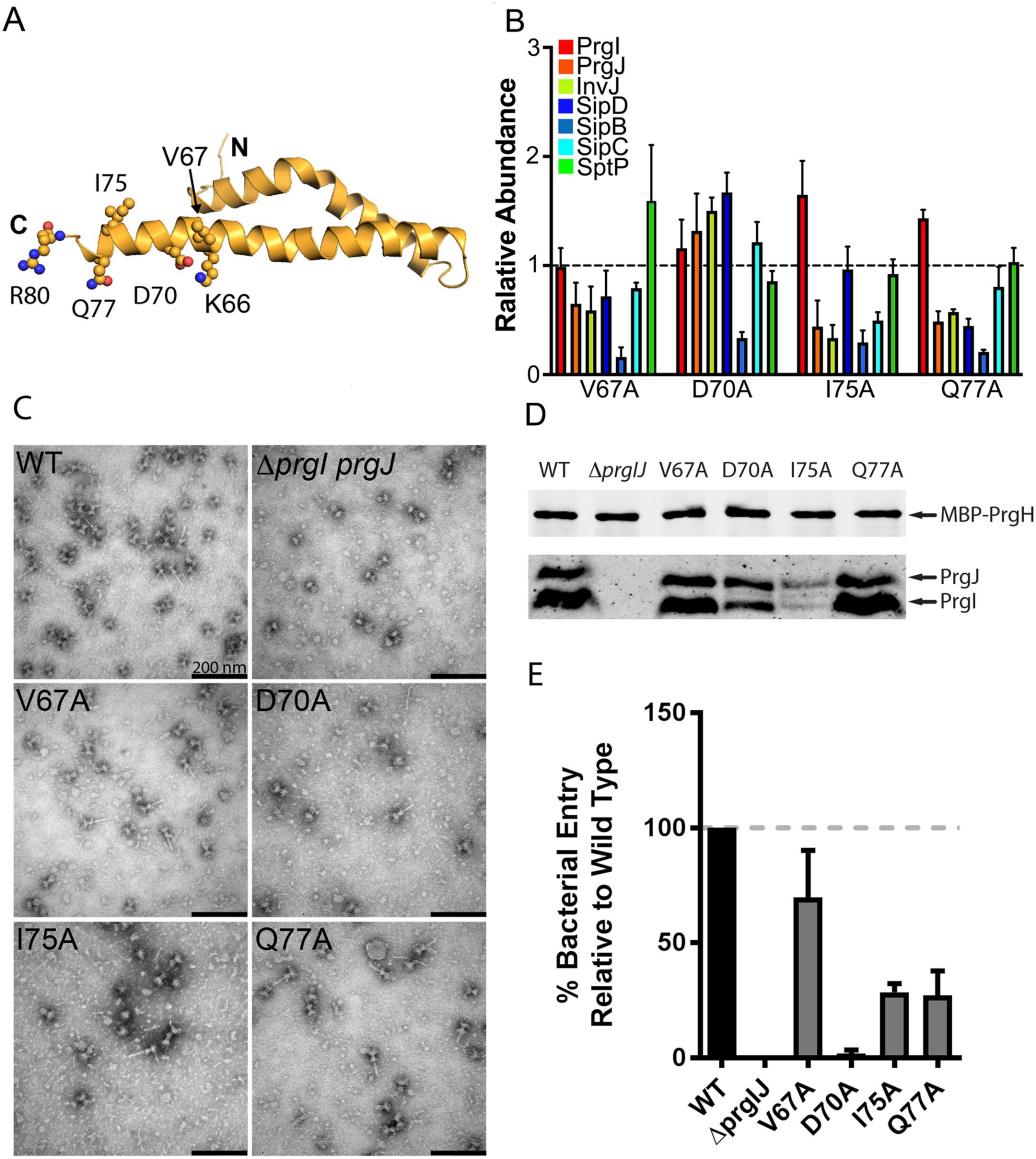
Secretion defects associated with C-terminal residues reveals a complex role for the lumen of the needle filament in type III secretion. (A) Representation of the location of relevant residues within the atomic structure of PrgI. (B) Bar graph detailing the secretion phenotypes of *S*. Typhimurium expressing the indicated PrgI mutants. The relative abundance of the secreted substrates has been standardized relative to WT, which was given a value of 1 and is demarcated by a gray dashed line. All values represent the mean ± the standard deviation of three independent experiments. The data was compiled from the data presented in S2 Fig. The underlying data for this panel can be found in S5 Data. (C and D) Electron micrographs (C) and western blot analysis (D) of affinity purified needle complexes obtained from *S*. Typhimurium strains expressing the indicated PrgI mutants. Samples for the western blot analysis were standardized based on the amount of PrgH. (E) Cultured epithelial cell invasion ability of *S*. Typhimurium strains expressing the indicated PrgI mutants. Numbers represent the percentage of the inoculum that survived antibiotic treatment due to internalization and are the mean ± standard deviation of three independent experiments normalized to WT, which was set to 100%. The underlying data for this panel can be found in S6 Data. WT, wild-type.

The lumen of the needle substructure is a unique surface in that unlike other structural elements of the needle filament, it interacts with the various substrates as they are secreted by the T3SS. The electrostatic surface potential of the lumen residues creates an alternating positive and negative charge pattern, which spirals upwardly about the needle. PrgI residue K66, the amine group of Q77, and residue R80 form the positively charged surface, while residues D70 and the carbonyl group of Q77, and possibly the main chain of the peptide backbone, form the negatively charged surface (Fig 5A). Our ASM screen identified a unique phenotype manifested as a specific deficiency in the secretion of the translocase SipB (Fig 5B). The PrgI mutations resulting in this phenotype include D70A and Q77A, which physically line the surface of the lumen, and V67A and I75A, which lie in close proximity to the lumen (Fig 5A). EM analysis of negatively stained isolated needle complexes from these mutants showed fully assembled needle complexes, although the structures obtained from the D70A and I75A mutants appeared to have a reduced number of needle filaments compared to wild type (Fig 5C). Confirming these observations, immunoblot analyses showed that the levels of PrgI and PrgJ in needle complexes obtained from *S*. Typhimurium strains expressing the PrgI mutants V67A or Q77A were equal to those obtained from wild type, whereas the levels of these proteins were reduced in complexes obtained from the D70A and I75A mutant strains (Fig 5D). The observation of fully assembled needle complexes is consistent with the fact that these mutants exhibit a selective defect in the secretion of SipB but no observable defect in the secretion of other substrates of the T3SS (Fig 5B). To evaluate whether the selective reduction in SipB secretion observed in these mutants resulted in a reduction of type III secretion function, we tested their ability to invade cultured epithelial cells, an assay that strictly requires the protein translocase SipB. We found that *S*. Typhimurium expressing PrgI^D70A^ was drastically affected in its ability to enter into host cells, indicating that this mutant strain is not able to deploy the protein translocase SipB upon contact with host cells (Fig 5E). This strong phenotype is striking considering the observation that needle complexes from this mutant strain are apparently normal and able to secrete all the other substrates of the T3SS. In contrast, *S*. Typhimurium expressing PrgI^V67A^, and to a lesser extent PrgI^I75A^ and PrgI^Q77A^, were able to invade cultured epithelial cells, indicating that despite a reduction in the secretion of SipB, the injectisomes assembled with these mutant needle proteins are able to deploy a functional translocon on the target cell membrane (Fig 5E). Taken together, these results indicate that conformational changes in the lumen of the central channel of the needle filament and, more specifically, in the C-terminal α-helix, which provides the relevant residues to form the lumen surface, can have a significant impact in the secretion process. Furthermore, these observations suggest a mechanistic framework for signal transduction and protein secretion reprogramming of the T3SS machine through conformational changes that may affect the properties of the lining of the needle filament lumen.

**Fig 6:**
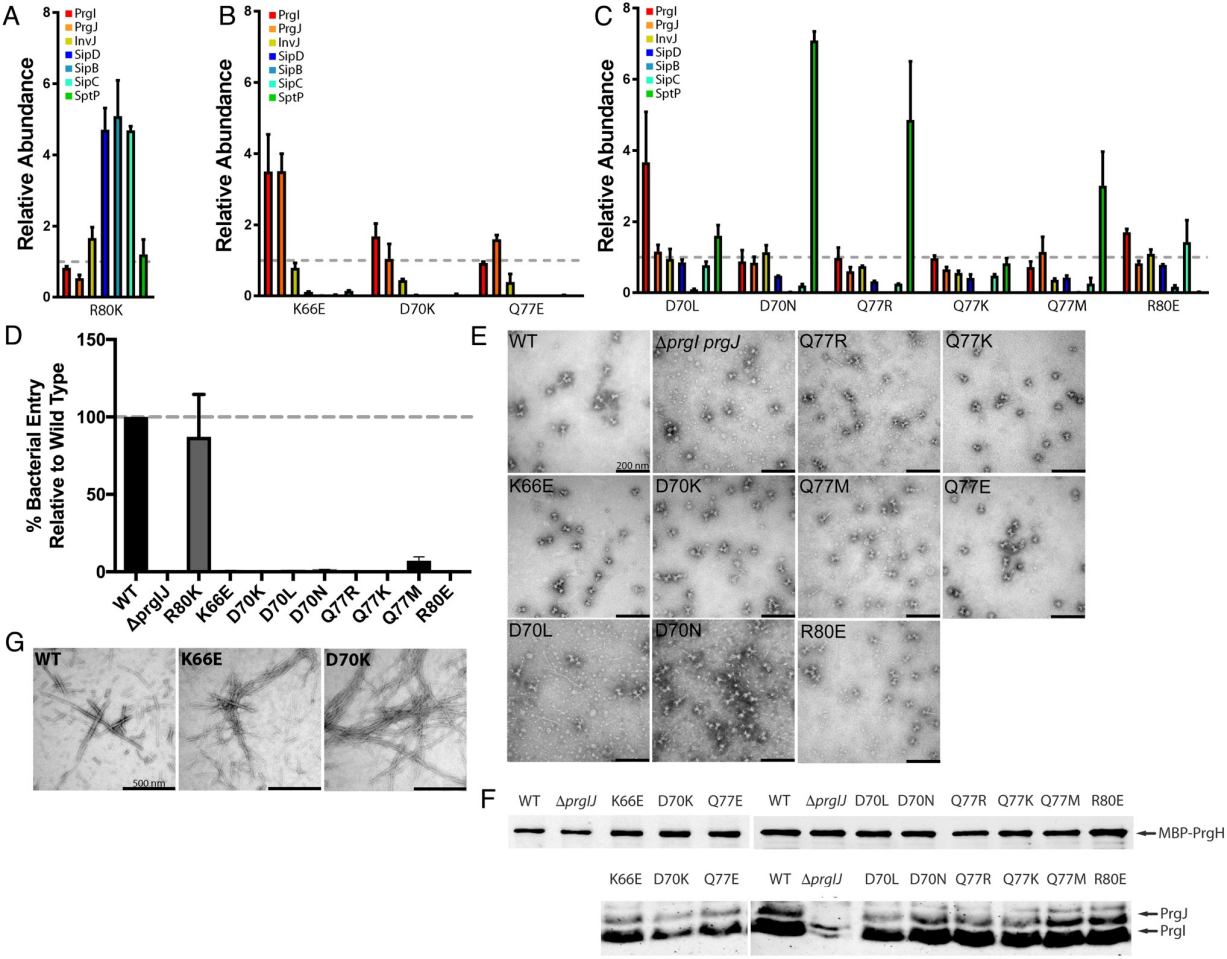
Targeted mutagenesis of PrgI lumen residues results in multiple phenotypes. Bar graphs detailing the secretion phenotypes of the targeted lumen mutants. (A–C) Bar graph detailing the constitutive (A), early substrate–only (B), and SipB-deficient (C) phenotypes of *S*. Typhimurium expressing the indicated PrgI mutants. The relative abundance of the secreted substrates has been standardized relative to WT, which was given a value of 1 and is demarcated by a gray dashed line. All values represent the mean ± the standard deviation of three independent experiments. The underlying data for panels A, B, and C can be found in S7, S8 and S9 data, respectively. (D) Cultured epithelial cell invasion ability of *S*. Typhimurium strains expressing the indicated PrgI mutants. Numbers represent the percentage of the inoculum that survived antibiotic treatment due to internalization and are the mean ± standard deviation of three independent experiments normalized to WT, which was set to 100%. The underlying data for this panel can be found in S10 Data. (E and F) Electron micrographs (E) and western blot analysis (F) of affinity-purified needle complexes obtained from *S*. Typhimurium strains expressing the indicated PrgI mutants. Samples for the western blot analysis were standardized based on the amount of PrgH. The western blot has been cropped and rearranged so that the order of the mutants follows the text. (G) Electron micrographs of purified recombinant WT PrgI and the K66E and D70K lumen mutants following in vitro polymerization. WT, wild-type.

To test this hypothesis, we sought to identify PrgI mutants that could potentially recapitulate the various phenotypes identified in this screen (i.e., constitutive, early substrates only, and SipB-deficient secretion only) by targeting residues that are predicted to affect the needle filament lumen structure. We specifically mutagenized the needle filament lumen residues K66, D70, Q77, and R80 by replacing them with similarly or oppositely charged (R, K, and/or E), polar uncharged (N or Q), or hydrophobic (L or M) residues. We then tested the resulting mutants for their ability to secrete early, middle, or late substrates or to mediate bacterial invasion, a measure of type III secretion function. Remarkably, we found that through these changes, we were able to identify mutants exhibiting each one of the phenotypes detected in our ASM screen targeting the entire needle protein. Secretion assays revealed that all the targeted mutants displayed a secretion phenotype. Thus, the R80K displayed a constitutive phenotype (Fig 6A); the K66E, D70K, and Q77E mutants were able to secret only early substrates (Fig 6B); and the D70L, D70N, Q77R, Q77K, Q77M, and R80E mutants showed a selective defect in the secretion of SipB only (Fig 6C).

Like other PrgI mutants displaying a constitutive secretion phenotype (e.g., S49A and R58A), *S*. Typhimurium expressing PrgI^R80K^ was able to invade cells (Fig 6D), indicating that the altered secretion profile was not due to the inability of the needle filament to bind the tip protein. Modeling of the PrgI^R80K^ mutant needle filament predicts a lumen with a charge distribution similar to that of wild type like (S6 Fig). It is interesting, however, that a minor change in a residue’s side chain (i.e., a guanidine group to an ammonium group) could have such a dramatic effect on secretion.

The needle filament lumen residues whose mutation resulted in secretion of only early substrates (K66E, D70K, and Q77E) are devoid of any inter- and intramolecular contacts in the assembled needle filament [12]. However, EM examination of negatively stained needle complexes obtained from strains expressing these PrgI mutants revealed that two of these mutants (K66E and D70K) yielded bases with no visible or much shorter needle filaments, suggesting an assembly defect (Fig 6E). Consistent with this observation, immunoblot analyses of these mutants showed reduced levels of both the inner rod and filament proteins PrgJ and PrgI (Fig 6F). However, purified PrgI^K66E^ and PrgI^D70K^ needle proteins were able to assemble into filaments in vitro in a manner indistinguishable from wild type (Fig 6G). Since in vivo needle assembly requires the transit of the needle filament subunits through the lumen of the needle filament while in vitro assembly does not, these observations suggest that the K66E and D70K mutations may alter the needle filament protein transport process. Modeling of the electrostatic surface of the predicted lumens of filaments assembled with these PrgI mutants exhibited a variety of features ranging from negatively (K66E) or positively (D70K) charged or a surface with alternating positive and negatively charge surface (Q77E) similar to the wild-type–like lumen surface (S6 Fig). These alterations of the electrostatic surface of the predicted needle filament lumen surface may account for the phenotypes observed. Consistent with the defect in needle complex assembly, neither of the mutants of K66E or D70K were competent for invasion of cultured epithelial cells, a measure of the functionality of the SPI-1 T3SS (Fig 6D). These results indicate that the resulting mutant needle structures are unable to transduce the activating signal required for the secretion of middle and late substrates.

The targeting of the needle filament lumen for mutagenesis revealed six PrgI mutations displaying a SipB-deficiency phenotype: D70L, D70N, Q77R, Q77K, Q77M, and R80E (Fig 6C). Underscoring the complex role of the needle filament lumen in T3SS function in addition to the SipB secretion deficiency observed on these mutants, additional secretion alterations were also apparent, including deficiencies in the secretion of SipC (D70N, Q77R, and Q77M) or SptP (R80E) or even increased secretion of SptP (D70N and Q77R). These altered secretion patterns observed in these mutants translated into a complete deficiency in their ability to invade cultured epithelial cells, a measure of T3SS activity (Fig 6D). However, these mutants retained the ability to assemble needle complexes, which by electron microscopy appear indistinguishable from wild type (Fig 6E and 6G). This is noteworthy, as electrostatic surface potential calculations predict drastically different properties for the lumens of needle filaments assembled with the different PrgI mutants (S6 Fig). These calculations predict a positively charged lumen for D70L, D70N, Q77R, and Q77K, a wild-type–like lumen surface for Q77M, and a negatively charged lumen for R80E. Taken together, these observations are consistent with a complex role of the needle filament both in signal transduction as well as other aspects of the regulation of type III secretion function.

### Cryo-EM structures of PrgI needle filament mutants revealed differences in helical assembly

**Fig 7:**
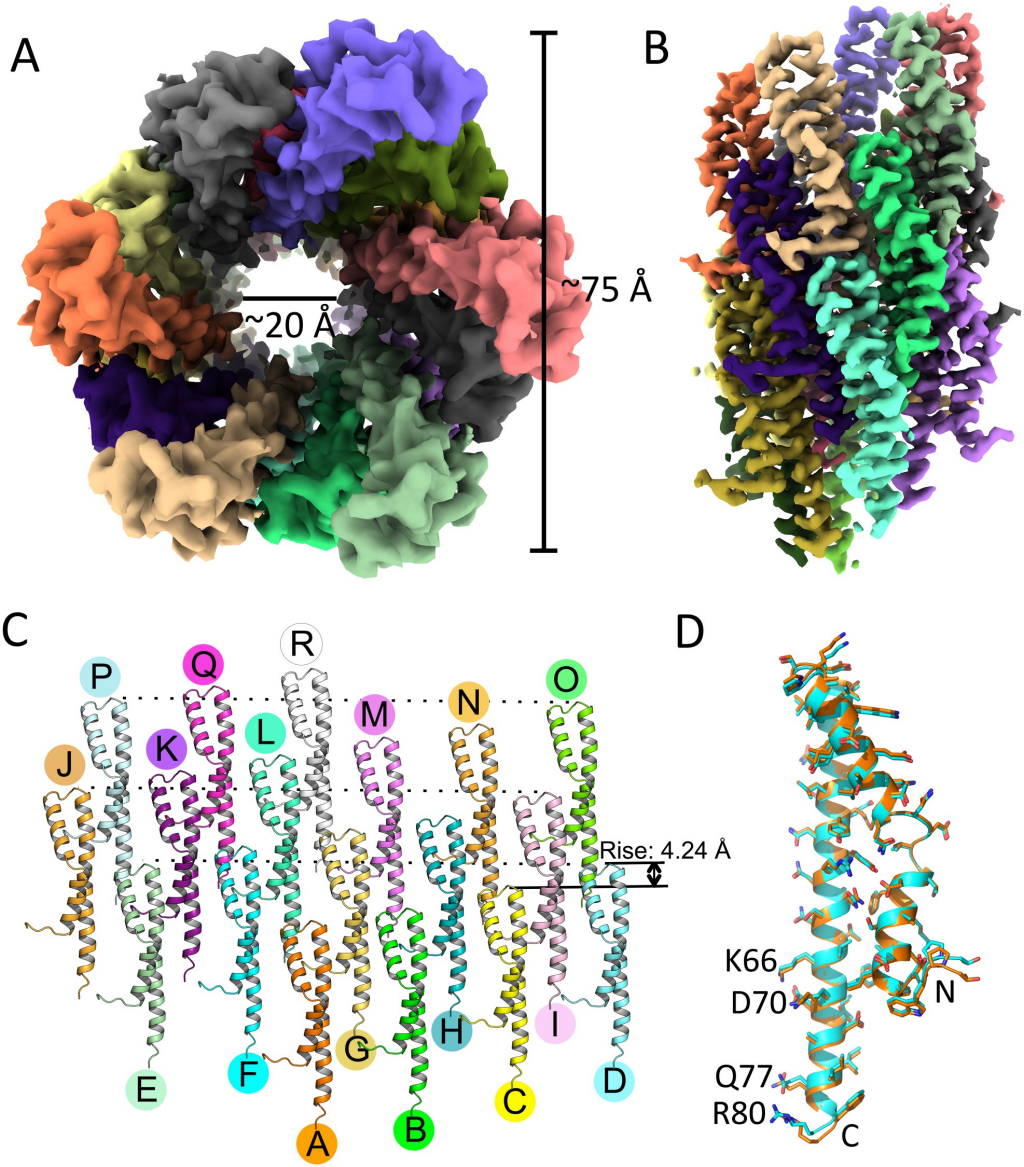
Cryo-EM reconstruction of PrgI needles attached to the base. (A and B) Top (A) and side (B) views of the PrgI needle. The outer diameter is approximately 75 Å, and the inner diameter is approximately 20 Å. PrgI subunits are rendered in different colors. (C) The PrgI subunits in the needle structure arranged in roughly three turns are shown. (D) Superimposition of a single PrgI subunit extracted from the needle structure attached to the base (cyan), with a PrgI subunit extracted from the structure of isolated PrgI filaments removed from the base (PDB: 6DWB; orange). cryo-EM; PDB, Protein Data Bank.

To gain insight into the structural bases for some of the phenotypes observed in our studies, we sought to compare the cryo-EM structures of needle filaments assembled with selected PrgI mutant proteins to those assembled with wild-type PrgI. A previous SSNMR study provided the first atomic model for the PrgI needle filaments assembled in vitro [12] and, after the original submission of this manuscript, Hu and colleagues reported a cryo-EM structure of isolated needle filaments (detached from the base) from an *S*. Typhimurium *ΔprgI* mutant strain overexpressing the PrgI protein [37]. However, no high-resolution structure of needle filaments in association with the base structure has been reported. As it is possible that constrains imposed on the filaments by their tight association to the base could result in structural differences, we sought to obtain structural information for needle filaments attached to the base substructure. Fully assembled needle complexes isolated by affinity chromatography were subjected to cryo-EM analysis (S7 Fig). PrgI needles were boxed out from fully assembled, intact needle complexes and segmented into square particles for 2D classification. The α-helices in each PrgI subunit and their N-terminal loops pointing to the solvent were clearly resolved in 2D class averages of the helical filaments (S7 Fig). The helical parameters, rise and twist, determined by helical indexing of the averaged power spectrum were measured to be 4.24 Å and 63.35°, respectively (S8 Fig), which are in close agreement with the previously reported values for the cryo-EM structure of isolated needle filaments. By applying helical symmetry in the EM reconstruction, we obtained a 3D map of the needle filament at 3.7 Å resolution as determined by the Fourier shell correlation (FSC) at 0.143 cutoff (S9 Fig). The first two residues of PrgI are flexible and could not be resolved in our map, although the 3D map allowed the unambiguous building of most of the side chains. The resulting atomic model is in good agreement with the cryo-EM structure of isolated needle filaments, except for a slightly smaller rise (4.24 Å versus 4.33 Å in isolated needle filaments) in the helical parameters (Fig 7A and 7B). The structure of the PrgI monomer extracted from the cryo-EM structure of filaments attached to the base is also entirely consistent with the structure of the monomer extracted from the cryo-EM structure of isolated needle filaments (the Cα root mean square deviation [RMSD] is 0.509 Å over 78 residues) (Fig 7D). These observations indicate that the process of isolation and overproduction of the needle filaments do not grossly alter the filament structure at least in a manner that can be discerned at this level of resolution. However, unlike the multiple class averages observed in isolated needle filaments [50], after 3D classification, all class averages converged into one final map, suggesting a greater degree of homogeneity in our sample, perhaps due to constrains imposed by the base substructure (S10 Fig).

In contrast, and as previously reported for the cryo-EM structure of isolated filaments [50], our structure differs from the SSNMR structure of in vitro assembled filaments. Even the closest structure of all 10 SSNMR models reported shows a difference in the twist (63.35° in our model versus 64.00° in the SSNMR model), which although relatively minor, leads to accumulating differences that ultimately results in noticeable variance in subunit packing. Consequently, the overall fitting of the two structures is poor (S11 Fig). The reasons for the observed differences between the cryo-EM and the SSNMR structures could be due to structural differences resulting from the different assembly mechanisms (i.e., in vitro versus in vivo) or differences strictly related to the methodologies utilized to determine the structures (i.e., cryo-EM versus SSNMR). To clarify this issue, we solved the cryo-EM structure of in vitro polymerized PrgI needle filaments (S12 Fig). We found that the structures of in vitro polymerized needle filaments is virtually identical to the structure of in vivo assembled filaments attached to the base structure, exhibiting equivalent helical twist and rise (S13 Fig). These results indicate that the differences between the cryo-EM structures of in vivo assembled filaments and the SSNMR structure of in vitro assembled needle filaments is due to differences in the experimental approaches utilized to solve the different structures rather than differences resulting from the assembly mechanisms.

**Fig 8:**
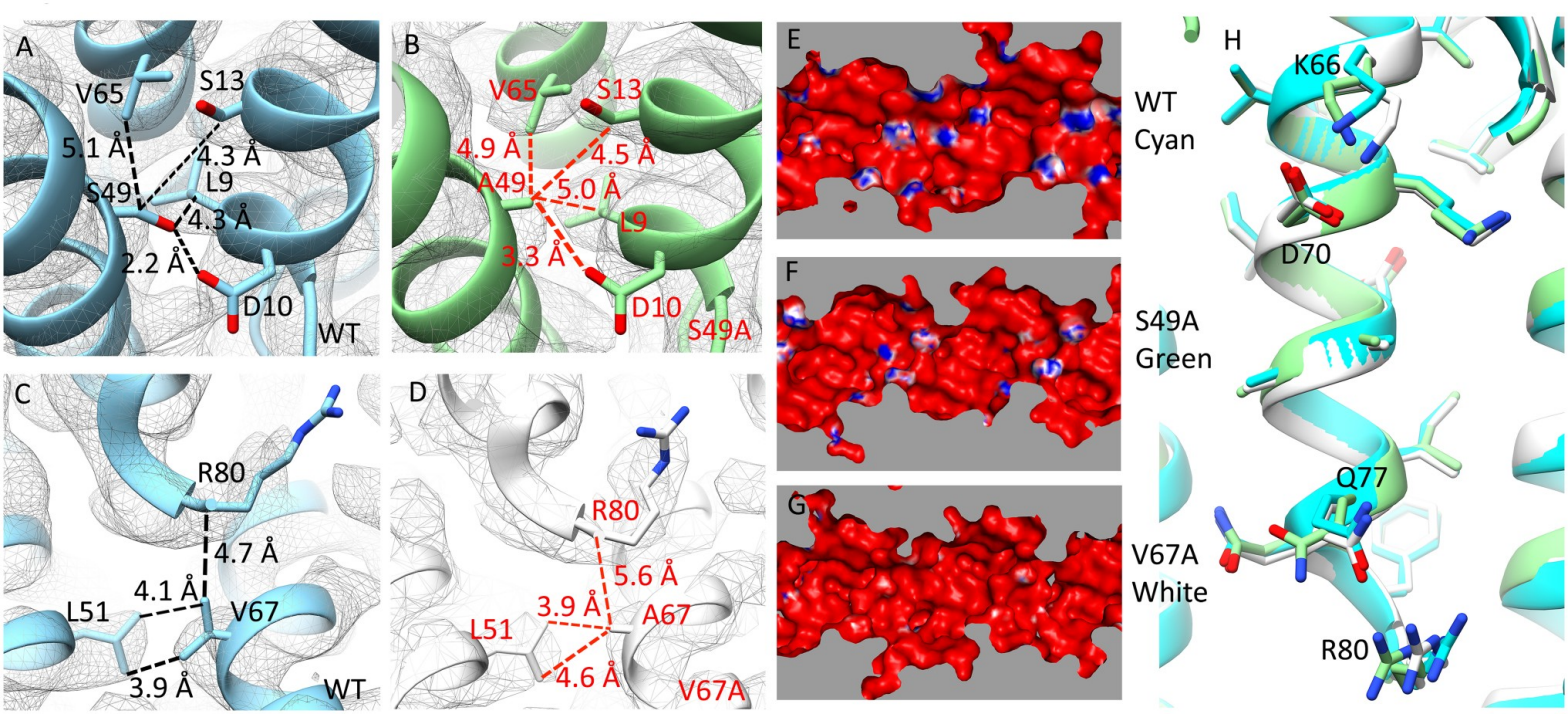
The cryo-EM structures of PrgI mutant proteins revealed structural differences. (A–D) Hydrogen bonds and hydrophobic interactions of S49 and V67 in WT needle filament and PrgI^S49A^ and PrgI^V67A^ filaments. (E–G) Electrostatic potential maps of the lumen of PrgI^WT^, PrgI^S49A^, and PrgI^V67A^ filaments. (H) Cross-section view of the PrgI filaments lumen alignment (PrgI^WT^ [cyan], PrgI^S49A^ [green], and PrgI^V67A^ [white]). cryo-EM, cryo electron microscopy; WT, wild-type.

Since we observed no differences between the structures of in vivo and in vitro polymerized needle filaments, we solved the cryo-EM structures of PrgI^S49A^ and PrgI^V67A^ filaments assembled in vitro from purified mutant proteins. These mutants are located within the C-terminal α-helix and resulted in specific phenotypes, such as constitutive secretion (PrgI^S49A^) or deficiency in the secretion of SipB (PrgI^V67A^). The 3D reconstruction of needle filaments assembled with PrgI^S49A^ resulted in two different class averages (S14 Fig): 1) a main class average involving 41,261 particles that generated a map virtually indistinguishable from wild type and 2) a class average involving a smaller (14,070) but significant number of particles, which resulted in a structure that while exhibiting the same helical twist as wild type, had a slightly shorter helical rise (4.19 Å compared with 4.24 Å of the wild type). The difference in helical rise observed in the latter class, while small, results in accumulating differences of the entire filament structure, leading to differences in subunit packing. Together, these two class averages accounted for virtually all (approximately 95%) of the manually picked and segmented particles. The 3D reconstruction of needle filaments assembled with PrgI^V67A^ fell into a single class average involving more than 99% of the particles (S15 Fig). The 3D reconstruction after 2D classification generated a map with an overall resolution of 2.9 Å. Although the helical rise is the same, PrgI^V67A^ filaments have a smaller twist than wild type (63.27° versus 63.35°). Although limited by the resolution, the differences in helical symmetry in the two mutants analyzed resulted in small but measurable local differences in the distance between the mutated residues and neighboring residues from adjacent PrgI subunits in the assembled filaments (Fig 8A–8D). However, no significant difference among PrgI^WT^, PrgI^S49A^, and PrgI^V67A^ was detected in the structures of the monomers extracted from the assembled filaments, with a Cα RMSD over 77 residues of 0.152 Å between PrgI^WT^ and PrgI^S49A^ and 0.225 Å between PrgI^WT^ and PrgI^V67A^ (S16 Fig).

The electrostatic potential of the residues lining the lumen of the needle filament show an alternating pattern of negative and positive charge, and it has been proposed that such a pattern may be important for the secretion mechanism [1]. This electrostatic pattern is largely determined by the side chains of K66, K69, D70, Q77, Q78, and R80 (S17 Fig). Limited by resolution or due to the flexibility of the residues, we cannot see the complete density of K69, Q77, and R80 in our maps (S18 Fig). However, the predicted electrostatic potential of the lumens of filaments assembled with the PrgI^S49A^ and PrgI^V67A^ mutants showed significant differences with wild type (Fig 8E–8G). These differences could be due to differences in side chain conformations (Fig 8H) or helical symmetry. To clarify this issue, we fitted PrgI^V67A^ monomer into the map of PrgI^WT^ filaments and found that the resulting lumen electrostatic potential was essentially the same as that of the PrgI^V67A^ filaments (S19 Fig). These results argue that differences in helicity itself may not account for the observed differences in the electrostatic potential of the lumen surface. Rather, it suggests that slight differences in side-chain configuration may be ultimately responsible for the observed differences in the electrostatic pattern of the lumen. However, it is also possible that the difference in side chain conformation themselves is the result of differences in helicity. Taken together, these results indicate that the PrgI mutations resulted in defined structural changes altering the surface properties of the lumen, which may be responsible for the phenotypes observed in bacteria expressing these mutant filament proteins.

### NMR analyses of PrgI mutants with altered phenotypes revealed long-range structural changes that suggest a mechanism for signal transduction

**Fig 9:**
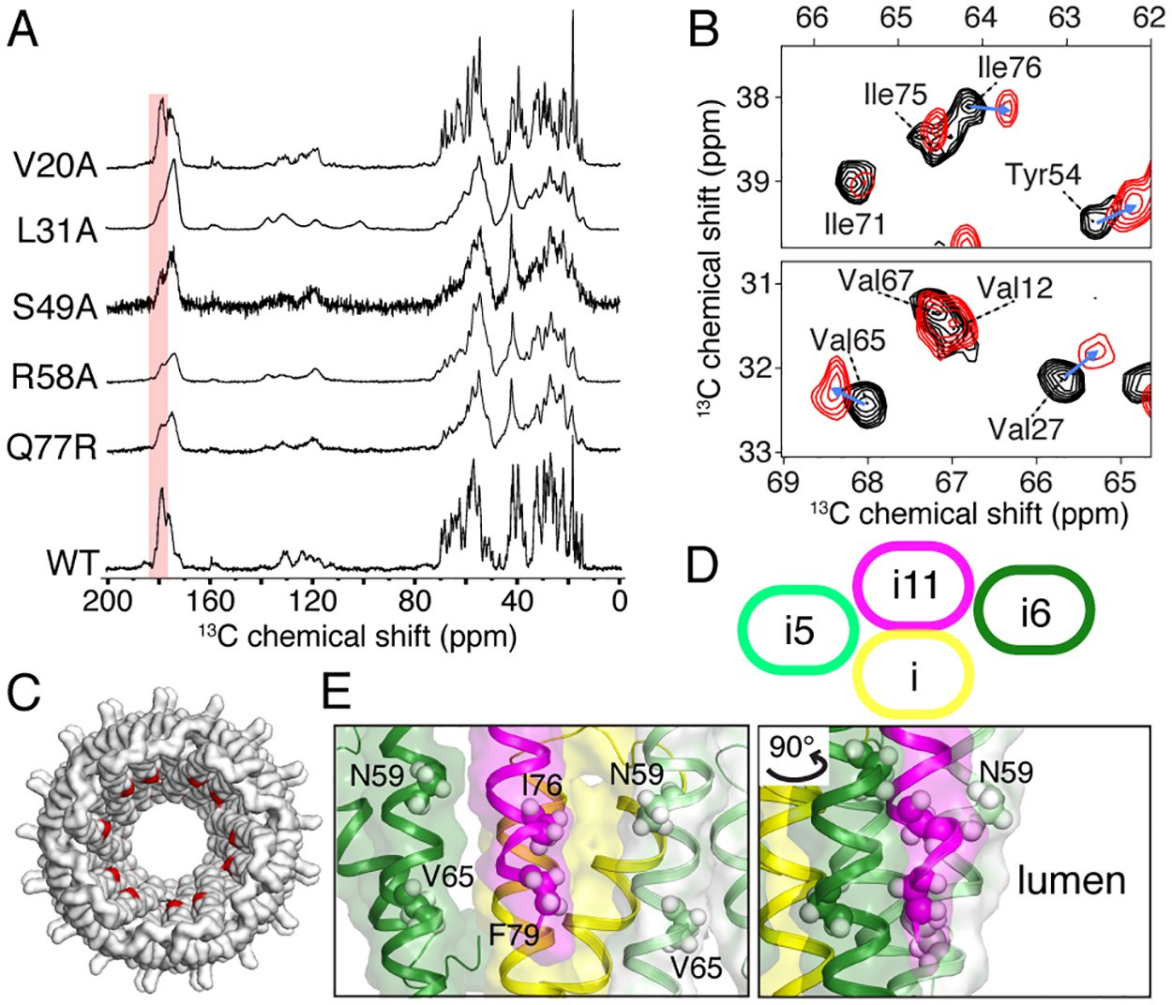
SSNMR studies of PrgI mutant needle filaments. (A) 1D ^13^C-detected SSNMR spectra of WT and different PrgI needle mutants. The carbonyl region indicative of α-helical conformation is highlighted in red. (B) ^13^C-detected chemical shift perturbations between WT (in black) and V20A (in red) PrgI needles detected on a 2D ^13^C–^13^C correlation experiment. (C) Top view of the T3SS needle structure [12]; long-range perturbations observed in the V20A mutant are colored in red. (D) Schematic representation of the intermolecular interfaces in the T3SS needle between subunits (i), (i + 5), (i + 6), and (i + 11), composed of an axial interface (i − i + 11) and two lateral interfaces (i − i + 5 and i − i + 6). (E) SSNMR chemical shift perturbations are located at the C-terminal helix for several residues forming the axial interfaces and buried in the subunit–subunit hydrophobic network. SSNMR, solid-state NMR; T3SS, type III secretion system; WT, wild-type.

To shed more light on the structural changes underpinning the mechanism of signal transduction, we employed magic-angle spinning solid-state NMR (MAS-SSNMR) spectroscopy to investigate the molecular conformation of different PrgI mutants. The needle filament conformation of a subset of the identified mutants (V20A, L31A, S49A, R58A, Q77R) was examined by MAS-SSNMR and compared to the wild-type needle filament conformation determined in previous studies [12,35]. NMR chemical shifts are informative reporters on protein secondary structure and represent a powerful approach for scrutinizing structural changes of the needle filament structure at atomic resolution; the ^13^C carbonyl region (170–185 ppm) in particular is highly sensitive to the secondary structure. Based on their chemical shift signatures, we observed two PrgI conformations among the mutants examined (Fig 9A). PrgI^V20A^, like wild type, exhibits a mostly α-helical SSNMR signature associated with carbonyl signal approximately 177–178 ppm. In contrast, the PrgI mutants L31A, S49A, R58A, and Q77R filaments exhibit a less pronounced α-helical–rich conformation as observed by additional signal at approximately 173–174 ppm. As a measure of the structural homogeneity of the PrgI subunits in the filamentous assembly, SSNMR line widths reveal that the PrgI subunits are packed in a very homogeneous manner for the V20A mutant, while important structural polymorphism is observed for L31A, S49A, R58A, and Q77R, reflected by broad NMR lines. Previous work reported that PrgI subunits polymerize in two different filamentous structural states, one observed for wild-type PrgI [12] and another for the double mutant V65A/V67A [35]. In addition, wild-type MxiH needles (from Shigella) polymerized in vitro [36] [51] or natively grown [52] indicate a comparable 3D architecture compared to wild-type PrgI needles. The SSNMR spectral features observed in the present study indicate that the PrgI mutant V20A assembles into a structural state similar to the wild type (α-helical conformation, single PrgI polymorph), while L31A, S49A, R58A, and Q77R present the structural features previously observed in the V65A/V67A mutant (less pronounced α-helical conformation, many and indistinguishable PrgI polymorphs) [35]. We next addressed the local structural changes associated with the point mutation V20A, since in contrast to the other mutations, these filaments lead to high-resolution SSNMR data. We employed multidimensional ^13^C–^13^C SSNMR spectroscopy to derive ^13^C chemical shift perturbations on the local level by comparing V20A to the wild-type PrgI chemical shifts reported by Loquet and colleagues [53] (Fig 9B). A threshold of 0.3 ppm on Cα resonances (corresponding to approximately two times the ^13^C line width [53]) was used to identify meaningful structural perturbations, here observed for residues W5, T18, V27, T28, A35, A36, P41, Y54, N59, V65, I76, and F79. Besides residues in close proximity to the mutation site (W5, T18, A35, A36, P41, and Y54), we identified several long-range conformational perturbations for residues N59, V65, I76, and F79, located at the C-terminal helix. Visual inspection of these long-range perturbed residues on the T3SS needle structure indicates that they are not exposed to the surface (Fig 9C–9E) but rather buried in the subunit–subunit network, as also reflected in the calculated accessible surface area (S20 Fig). The supramolecular needle organization is stabilized by tight subunit–subunit interactions at the axial interface between subunit (i) to (i + 11) and at two lateral interfaces between (i to i + 5) and (i) to (i + 6) [12] (Fig 9D). The V20A mutation leads to long-range effects on lumen-oriented residues, involved in numerous needle-stabilizing contacts. The residues N59, V65, I76, and F79 are close in space along the two lateral interfaces between (i) − (i + 5/6) (Fig 9E), with a peripheral access to the inner lumen. These residues have been previously shown to be involved in the lateral interface stabilizing the wild-type needle architecture, which is formed by tight hydrophobic interactions between the C-terminal helices of adjacent PrgI subunits [12]. Moreover, this C-terminal region has been previously identified in a V65A/V67A mutant to be prone to structural polymorphism associated with conformational transition between α-helical to β-sheet secondary structure [35]. Our results pinpoint to the plasticity and crucial role of the C-terminal helix in maintaining the wild-type needle structural state. Point mutations located at the C-terminal helix (S49A, R58A, and Q77R studied here and V65A/V67A reported previously [35]) lead to an alternative needle structural state that might be linked to a putative activated state [27]. We observed a similar behavior for the L31A mutant, and as this position is crucial in stabilizing the intramolecular PrgI fold through the contact L31-Y47 [12], we hypothesize that the small local perturbation at the L31 position has a direct effect on the C-terminal helix, leading to similar effects compared to point mutations at this helix.

Previous work has reported the very high structural rigidity of PrgI subunit in the context of the needle assembly [12]. We demonstrate here that local perturbations induced by the V20A mutant trigger long-range perturbations at the C-terminal helix, importantly at residues involved in stabilizing the lateral helix–helix interface that maintains the lumen of the needle filament. We assume that the global needle architecture is highly similar between the wild-type and the V20A mutant since very similar SSNMR fingerprints were observed for these needle filaments. Intuitively, we propose the hypothesis that signal transduction events might result in conformational changes in the C-terminal helix that maintain the lumen architecture. These variations may be mediated by local effects, exemplified by the point mutations in the C-terminal domain or at residues involved in the helix–helix interactions, and by subtle long-range perturbations, exemplified by the V20A mutant described here. While the structural basis of these long-range effects is unknown, future investigations combining high-resolution cryo-EM and SSNMR might bridge the gap between the determination of long-range symmetries (subunit–subunit interfaces and helical handedness parameters) and local residue–residue perturbations to delineate the conformational variations at the lumen surface.

### Discussion

Multiple lines of evidence indicate that the activation of type III secretion machines derives from bacterial contact with the target eukaryotic cells (reviewed in [27]). It is also believed that the sensing step is carried out by the tip complex, which is located at the distal end of the needle filaments. Conformational changes in the tip complex are thought to generate an activating signal that must be transduced by the needle filament to the secretion apparatus components on the inner membrane and cytoplasm of the bacterial cell. Although these concepts are well accepted in the field, the mechanisms by which the secretion machine senses the target cell, generates an activating signal, and transduces it to the secretion machine are not understood. Through a mutagenesis screen targeting most residues of the *S*. Typhimurium needle filament protein PrgI, we have obtained new insight into a potential signal transduction mechanism that leads to the activation and reprogramming of the secretion machine. The screen identified several mutants with defined phenotypes that suggest a complex role of the needle filament in the control of type III secretion.

One of the phenotypes observed was characterized by PrgI mutations that resulted in the secretion of only early substrates. Needle complexes isolated from most of these mutants (e.g., L9A and F16A) lacked needle filaments even though purified PrgI mutants were able to assemble into filaments in vitro in a manner indistinguishable from wild type. The in vitro and in vivo needle filament assembly mechanisms differ significantly in that, in contrast to in vitro, in vivo assembly requires the needle subunits to travel through the central channel of the needle filament prior to their addition to the growing end of the needle filament. Therefore, PrgI residues L9 and F16 must play a specific role in in vivo assembly. Previous SSNMR data indicate that L9 and F16 are part of an intramolecular interaction network centering about the bottom portion of PrgI near the N- and C-termini [12]. Specifically, L9 interacts with the C-terminal residue K69, and F16 interacts with three C-terminal residues (A60, Q61, and T64). A change in any of these interactions could have an effect in the ability of PrgI to polymerize into a filament by disrupting the helix-turn-helix intramolecular fold. Furthermore, given the location of these residues, these mutations may affect the ability of PrgI to interact with the inner rod protein PrgJ, thus destabilizing both the needle filament and the inner rod, resulting in the shedding of these proteins into the culture supernatant.

Several PrgI mutations resulted in enhanced secretion, a phenotype that has been previously observed in mutant strains lacking the tip complex. Consistent with these observations, some of these PrgI mutants (L31A and L56A) were unable to display the tip protein SipD on the bacterial surface, thus defining critical residues for SipD–PrgI interactions. However, several PrgI mutants (D10A, D11A, V20A, S49A, E53A, L56A, R58A, N63A, and N78A) exhibiting enhanced secretion retained the ability to display SipD on their surface. We hypothesize that some of these mutations may represent signal transduction relay points that transmit to the needle filament the putative conformational changes in the tip protein, resulting from the activating signal. This hypothesis is supported by previous NMR studies that have mapped the atomic interface between SipD and PrgI [46]. Previous studies identified mutations in the needle filament proteins from *Shigella* (MxiH) [29] and *Yersinia* (YscF) [30,31] that led to constitutive secretion. However, the number and location of the mutants identified in PrgI differ with these previous observations. The study of MxiH [29], which shares a high degree of sequence and structural similarity with PrgI, identified three alanine mutations resulting in the constitutive secretion of effectors, which corresponds to P41, Q48, and D70 of PrgI. However, none of these mutations resulted in a constitutive phenotype in our study. Two studies of *Yersinia* YscF [30,31], which shares a low degree of sequence similarity with PrgI, identified six constitutive mutants mapping to a location within its predicted N-terminal extension. Although due to sequence divergence, it is difficult to exactly pinpoint the structural equivalent of these positions in PrgI, these residues would roughly correspond to D21, S39, D40, and A60 of PrgI. However, alanine substitutions at these positions did not result in measurable phenotypes. These results suggest important differences between the different needle filament proteins that may relate to differences in the compositions of the different tip structures in these T3SSs.

An intriguing observation in our analysis is the finding of PrgI mutations that are specifically altered in their ability to secrete the protein translocase SipB while retaining the ability to assemble an apparently wild-type needle filament and secrete other middle (SipD and SipC) and late (effectors) substrates in a manner similar to wild type. How this observation of selective secretion defect may relate to the mechanisms of reprograming of the secretion machine is unclear but suggests that there may be conformational states of the needle filament that may render it permissive for the secretion of some substrate but not others. Intriguingly, the mutations that led to this phenotype involved amino acids that line the surface of the lumen of the needle filament (i.e., D70A and Q77A) or that lie in close proximity to the lumen (i.e., V67A and I75A). These observations suggest a potential critical role for the lumen of the needle filament in decoding signal transduction events that lead to the reprograming of the secretion machine. Consistent with this notion, we were able to generate all the different phenotypes observed through the entire PrgI ASM screen (i.e., early substrates only, constitutive secretion, and SipB deficiency) by targeting the residues that line the lumen of the needle filament. In this context, it is intriguing that the majority of the mutations exhibiting measurable phenotypes mapped to the carboxy-terminal helix, which also line the surface of the lumen filament. Our cryo-EM analysis of selected mutants also supports the notion that some of the phenotypes observed may be the result of alteration of the surface properties of the lumen of the filament structure. Indeed, the two mutants analyzed by this approach showed subtle but distinct changes on the electrostatic properties of the lumen surface even though the mutated residues do not directly participate in configuring the lumen surface. The cryo-EM analysis of the mutants also suggests that changes in the helical properties of the assembled filament may participate in the mechanisms of signal transduction, perhaps by indirectly influencing the configuration of the needle filament lumen.

Our NMR analysis of different mutants provides further support for a potential role of the carboxy-terminal helix of PrgI in signal transduction. Previous SSNMR studies have revealed a significant structural rigidity for the wild-type needle filament [53]. However, the NMR chemical shifts observed for all but one of the mutants analyzed showed a remarkable polymorphism in the carboxy terminal helix, which exhibit a pronounced β-sheet–rich conformation with many indistinguishable PrgI polymorphs. Interestingly, this β-sheet–rich conformation has been previously reported for a PrgI mutant (V65A/V67A), which had been isolated in an attempt to generate a soluble form of PrgI [35]. It was concluded that the C-terminal α-helix to β-sheet conformational change in the needle filament protein was essential for its assembly. However, although a superficial analysis of the mutant used in those studies indicated that it retained wild-type function, our analysis indicates that mutations in these residues result in defined secretion phenotypes (see Table 1). Furthermore, the β-sheet configuration is not observed in the wild-type needle filament. Instead, we propose that the conformational plasticity of the C-terminal α-helix of PrgI may be central for the signal transduction mechanisms. We hypothesize that localized changes in the needle filament originating at the tip of the needle may translate into long-range effects, resulting in conformational changes in the C-terminal α-helix that may lead to changes in the lumen surface, which we have shown here may be central for the reprograming of the secretion machine. In fact, consistent with this hypothesis, our NMR analyses of one of the identified mutants (V20A) indicate that the localized changes triggered by the mutation translate into a long-range effect, resulting in perturbations at the C-terminal helix, in particular, in residues involved in stabilizing the lateral helix–helix interface that maintains the lumen architecture of the needle filament.

In summary, our studies have provided major insight into potential mechanisms of signal transduction of the needle filament of T3SSs. Given the conservation of the general architecture of the needle filament in other T3SSs, this information may have broad implications for the function of T3SSs in general.

## Materials and methods

### Bacterial strains and plasmids

Bacterial strains are derivatives of either SJW2941 [54] or SL1344 strains [55]. Allelic exchange was employed to generate Δ*prgI* Δ*prgJ*, Δ*sipD*, mbp*-prgH*, and triple FLAG-tagged *sipD* and *sptP* strains, as previously described [56]. Standard cloning methodologies were used to introduce mutations in *prgI*, which was encoded in the pWSK29 low-copy plasmid vector [57]. To maintain equal expression levels of *prgI* and *prgJ* for complementation studies, the plasmid also encoded *prgJ* in the same arrangement as that of the chromosomal locus and was expressed in *S*. Typhimurium Δ*prgI ΔprgJ* mutant background. Out of the 80 amino acids of the full-length PrgI protein, 68 nonalanine-containing positions were targeted for ASM. For in vitro polymerization studies, wild-type PrgI and various mutants were cloned into pET15b (Novagen) such that the constructs contained an N-terminal, thrombin-cleavable 6 × His-tag. S1 and S2 Tables list the strains and plasmids used in these studies, respectively.

### Protein secretion assays and quantitative immunoblotting

*S*. Typhimurium strains expressing the different PrgI mutants were assessed for substrate secretion by growing the bacteria in LB supplemented with 0.3 M NaCl to late logarithmic growth phase, a condition that stimulates the expression of the SPI-1 T3SS [58]. Culture supernatants were obtained and, subsequently, treated with trichloroacetic acid to precipitate proteins, as previously described [39]. SDS-PAGE was used for the resolution of proteins >20 kDA, whereas tricine-PAGE was used for the resolution of proteins <20 kDA (i.e., PrgI [8.8 kDA] and PrgJ [10.9 kDA]). Immunoblot analysis was utilized to detect early (PrgI, PrgJ, and InvJ), middle (SipD, SipB, and SipC), and late (SptP) substrates with primary antibodies specific to the protein or a FLAG epitope tag (Sigma-Aldrich). The LI-COR Odyssey imaging system (Lincoln, Nebraska) allowed for the infrared fluorescent detection of DyLight conjugated secondary antibodies (emission 800 nm [ThermoFisher Scientific, Waltham, Massachusetts]). Quantitation of specific bands was performed with Odyssey v3.0 software (LI-COR). To determine the secretion profile for each mutant, the abundance of each substrate was normalized relative to the wild-type control. All values represent the mean ± the standard deviation of three independent experiments.

### SipD surface display assay

Immunofluorescence experiments for the detection of SipD at the tip of T3SSs in *Salmonella* were performed as previously described [45] with some modifications. Bacteria expressing the mutant of interest were grown as described above, harvested, washed with 1 × PBS, and fixed in 4% paraformaldehyde in 1 × PBS. Following 20-min incubation at room temperature, the fixed bacteria were gently washed as above and blocked with 250 μl of 3% BSA in 1 × PBS. After 30 min, the bacteria were then probed for 1 hr with an antibody directed against the FLAG epitope to detect SipD (dilution of 1:1,000). The sample was washed once and then added (1:5 dilution) to a polylysine-coated glass cover slip submerged in 500 μl of 1 × PBS in 12-well culture plate. Following 2-hr incubation at room temperature, the adhered bacteria were gently washed once with 1 × PBS, covered with 300 μl of 3% BSA in 1 × PBS containing antibodies (dilution of 1:10,000) directed against *S*. Typhimurium lipopolysaccharide (LPS), and incubated overnight at 4 °C. In the following morning, the labeled bacteria were washed three times with 1 × PBS and subsequently probed with conjugated antibodies Alexa Fluor 594 anti-rabbit and Alexa Fluor 488 anti-mouse for the detection of LPS and SipD, respectively (Invitrogen, Carlsbad, California), and coverslips were mounted onto slides. Immunofluorescence microscopy was performed on a Nikon TE2000 microscope, and images were acquired using an Andor Zyla 5.5 sCMOS camera (Belfast, United Kingdom) run by the software Micromanager (https://www.micro-manager.org).

### Cell invasion assays

To assess the function of a T3SS expressing a particular PrgI mutant, we used a gentamycin protection assay to report on bacterial internalization within a host cell, as previously described [47]. Briefly, human intestinal epithelial cells (Henle-407) were maintained and grown in DMEM supplemented 10% fetal bovine serum. To a 12-well plate that was nearly confluent, bacteria grown under SPI-1 T3SS inducing conditions were used to infect the epithelial cells at a multiplicity of infection (MOI) of 30. After 30-min incubation, cells were washed three times with prewarmed (37 °C) Hanks’s balanced salt solution (HBSS) and then incubated for 1 hr with HBSS supplemented with 50 μg/ml of gentamycin to kill any remaining extracellular bacteria. Cells were washed as above and then lysed with 300 μl of 0.1% sodium deoxycholate (DOC) in HBSS to release intracellular bacteria, which were enumerated by plating dilutions onto LB agar plates. Values for each mutant were normalized relative to those determined for wild type.

### Needle complex isolation

Details of isopycnic ultracentrifugation methods for obtaining purified needle complexes have been reported elsewhere [13]. Briefly, bacteria were grown in L broth to an absorbance at OD600 of 0.8 to 1.0, pelleted, and resuspended in 1/10 of the original volume in a solution of 0.5 M sucrose and 0.15 M Trisma base. Lysozyme and EDTA were added to a final concentration of 1 mg/ml and 10 mM, respectively, and cells were incubated for 1 hr at 4 °C. Spheroplasts were lysed by the addition of lauryidimethylamine oxide (LDAO) to a final concentration of 1%, and the samples were further incubated for 2 hrs. MgSO_4_ was added to a final concentration of 10 mM, and samples were centrifuged at 5,000 g for 20 min. The clarified sample was adjusted to pH 11 with NaOH, incubated for 1 hr at 4°C, and centrifuged at 60,000 g for 1 hour. The pellet was resuspended in a solution containing 0.1 M KCI-KOH (pH 11), 0.5 M sucrose, and 0.1% LDAO and centrifuged at 60,000 g for 1 hour. The pellet was resuspended in TET buffer (10 mM tris-HCI [pH 8.0], 5 mM EDTA, and 0.1% LDAO) and loaded onto a 30% (w/v) CsCI density gradient. Gradient fractions were centrifuged at 60,000 g for 1 hr, and the pellets were washed with TET buffer. Needle complex isolation using affinity purification has been described elsewhere [59]. Briefly, an MBP-tagged PrgH allele was introduced into the different *S*. Typhimurium strains expressing the different PrgI mutations. Bacterial strains were grown in LB broth, cells were recovered by centrifugation and resuspended in lysis buffer (200mM Tris pH 7.5, 20% sucrose, 1mM EDTA, 0.25mg/ml of lysozyme, and cOmplete EDTA-free protease inhibitor cocktail [Sigma 4693159001]) and incubated on ice for 1 hr. Cells were lysed by the addition of 0.5% N-Dodecyl- *ß-*D-maltoside (DDM) (Anatrace D310S). Lysates were clarified by centrifugation, transferred to a fresh tube, and needle complex recovered by affinity purification on amylose resin.

### Expression and purification of recombinant PrgI for in vitro polymerization

Recombinant His-tagged PrgI was expressed in *Eschericia coli* BL21(DE3). The strains were grown in 250 ml of LB at 37 °C until the *A*_600_ reached 0.5–0.8. Subsequently, the cultures were cooled down to 30 °C, and the expression of PrgI was induced with 0.15 mM IPTG. Following expression overnight, the bacteria were harvested by centrifugation, washed with 1 × PBS, and resuspended with 1 × PBS supplemented with 50 mg of DNase I and 0.2 mM MgCl_2_. Cells were lysed using the One Shot cell disrupter per manufacturer’s instructions (Constant Systems Ltd., Daventry, United Kingdom). Inclusion body pellets containing His-tagged PrgI were obtained by centrifugation at 20,000 × *g*, 4 °C and for 30 min. Inclusion bodies were solubilized by resuspending with denaturing buffer (10 mM Tris, pH 8.0, 100 mM NaH_2_PO_4_, and 8 M urea) and placed on a rocking platform for 1.5 hrs. The sample was clarified by centrifuging as above and the resulting solution was mixed with Ni-NTA resin previously equilibrated with denaturing buffer. After incubating for 1 hr, the resin slurry was applied to a column and washed extensively with denaturing buffer. Refolding of His-tagged PrgI was initiated by washing with and incubating the bound resin in 1 × PBS overnight at room temperature. The protein was then eluted with 50 mM Tris, pH 7.5, 150 mM NaCl, and 250 mM imidazole. Following elution, the His-tag was cleaved off the protein with 1 U of thrombin. Polymerization of PrgI was initiated by concentrating the sample using an Amicon Ultra-15 centrifugal filter unit with a nominal molecular weight cut-off of 10 kDa. This process also allowed for buffer exchange into 20 mM MES, pH 5.5. Once a concentration of approximately 1 mg/ml was achieved, the sample was transferred to a fresh tube and allowed to further polymerize overnight at room temperature. Protein concentration was determined by measuring the *A*_280_ in 20 mM sodium phosphate, pH 6.5, and 6 M guanidine hydrochloride. A molar extinction coefficient of 11,460 was used for PrgI.

### Conventional electron microscopy

Purified needle complex and in vitro polymerized PrgI samples were deposited onto glow-discharged carbon-coated copper grids (300 mesh) and negatively stained with 2% phosphotungstic acid, approximately pH 7.0 (Electron Microscopy Sciences, Hatfield, Pennsylvania). Images were collected using an Olympus 6 megapixel CCD camera (Munster, Germany) run by the Morada Soft Imaging system in-line with a Tecnai Biotwin transmission electron microscope operating at 80 kV (FEI Company, Hillsboro, Oregon).

### Bioinformatic analyses

All 3D models of mutants for electrostatic calculations (see below) were predicted by the SWISS-MODEL server. The 23-mer needle structure of PrgI served as the template (PDB code: 2PLZ). Electrostatic surface potentials of the models were calculated using APBS software [60]. The molecular graphics program PyMol was used to generate all structural representations. The solvent accessible surface area of all of the residues in PrgI in relation to the needle was determined using *calc-surface* [61]. For each residue, the surface area was translated into a percentage by dividing the calculated surface area of a particular residue of PrgI by a reference surface area corresponding to the residue in a Gly-X-Gly peptide (X is any residue). The atomic coordinates of the needle structure (PDB code: 2PLZ) were used for these calculations. Chain K, which resides in the middle of the 23 mer of the needle, was chosen as representative for the bar graph shown in S6 Fig.

### Cryo-EM sample freezing and data acquisition

All protein samples and freezing conditions were screened on a Tecnai F20 electron microscope (FEI company) operated at 200 kV. For the imaging of fully assembled needle complexes three microliters of sample was applied onto glow-discharged holy carbon C-flat 2/1 400-mesh Cu grid (Protochips, Inc) with a thin carbon layer and plunged into liquid ethane using Vitrobot Mark III (FEI company) or IV (Thermo Fisher) at 10 °C and >95% humidity. Cryo-EM data were recorded on a Titan Krios electron microscope (Thermo Fisher) operating at 300 kV accelerating voltage, at a calibrated magnification of 47,000 × g using a K2 Summit direct electron detector (Gatan, Inc.) in super resolution mode, corresponding to 1.715 Å per physical pixel. In total, 1,424 movies with defocus values in the range of −1.0 to −3.5 μm were recorded with a dose rate of 9.2 electrons per pixel^2^ per second. The total exposure time was set to 16.125 sec, resulting in an accumulated dose of 47 electrons per Å^2^ fractioned into 43 frames. In vitro-assembled wild-type and mutant PrgI filaments were sonicated to obtain short and individual filaments. Three microliters of each sample were applied onto a holy carbon C-flat 2/1 400-mesh Cu grid and plunged into liquid ethane using a Vitrobot (Thermo Fisher). All the data sets were collected on a Titan Krios electron microscope (Thermo Fisher) operating at 300 kV accelerating voltage at a calibrated magnification of 130 k× using a K2 Summit direct electron detector (Gatan, Inc.) in super-resolution mode, corresponding to 1.05 Å per physical pixel. In total, about 1,500–2,000 movies of each sample with defocus values in the range of −1.0 to −3.5 μm were recorded. The dose rate was set to 5.9 electrons per Å^2^ per second. The total exposure time was set to 8 sec, leading to an accumulated dose of 47.2 electrons per Å^2^, fractioned into 32 frames per movie stack.

### Image processing

A total of approximately 1,400–2,000 dose-fractionated image stacks for each sample were subjected to beam-induced motion correction using MotionCor2 [62]. CTF parameters for each micrograph were determined by CTFFIND4 [63].

For the in vivo assembled PrgI needle filaments attached to the base, particle picking of the needle complex was done using sxhelixboxer.py in SPARX [64]. 2D and 3D classification of needle structures boxed out from fully assembled needle complexes was performed using RELION. In total, 60,297 particle segmentations were selected and subjected to reference-free 2D classification to discard particles categorized in poorly defined classes resulting in 53,866 particles that were used for further 3D calculations. A featureless cylinder generated with RELION was used as initial reference model for maximum-likelihood–based 3D classification. The initial helical symmetry parameters were imposed on the reconstruction. All four classes showed detailed features for all subunits and were subsequently subjected to 3D refinement that led to one map. The final map has a global nominal resolution of 3.7 Å.

For in vitro polymerized PrgI^WT^, PrgI^S49A^, and PrgI^V67A^ filaments, manual particle picking, 2D and 3D classification were done using RELION3 [65]. Particles from good 2D classes with detailed features were selected and subjected to 3D classification and refinement. The 3D map of PrgI needle (attached to the base) was used as initial map and the helical symmetry parameters of this structure were imposed on the reconstruction. To explore the possibility of different conformations, we did 3D classification of most of the 2D classes. For PrgI^WT^ and PrgI^V67A^ filaments, all the reconstructed 3D maps exhibited the same helical parameters (the twist and rise of PrgI^WT^ filaments are 63.34° and 4.25 Å; the twist and rise of PrgI^V67A^ filaments are 63.27° and 4.24 Å). In the case of PrgI^S49A^ filaments, two final maps were reconstructed with the same twist (63.34°) but different rise (4.25 Å and 4.19 Å). Detailed workflow of all the calculations are shown in S12, S14 and S15 Figs. A soft-edged 3D mask with a radius of 50% of the box size was created for post-processing.

All density maps were corrected for the modulation transfer function (MTF) of the K2 summit direct detector and then sharpened by applying a B factor that was estimated using postprocessing in RELION. Reported resolutions are based on the FSC using the 0.143 criterion. Layer-line images were calculated from map projections with SPARX (project and periodogram commands) [64]. All the image processing was carried out on the Yale High Performance Computing servers.

### Model building and fitting

The initial template was derived from in vitro polymerized PrgI filaments solved by SSNMR (PDB ID: 2LPZ). Models were docked into the EM density maps using Chimera, followed by iterative manual adjustments in Coot [66] and real-space refinements with Phenix [50]. The rise (translation) and twist (rotation) for the helices of each model in SSNMR ensemble (PDB ID: 2LPZ) were calculated with Chimera (match showMatrix command) using two adjacent subunits in the models. Figures of structures were generated with Coot, PyMOL, and Chimera.

### SSNMR

SSNMR experiments were carried out as previously described [12,53]. Briefly, SSNMR experiments recorded on a 800-MHz spectrometer (Bruker Biospin) equipped with a 3.2-mm triple resonance MAS probe. Chemical shifts were calibrated using DSS. The MAS frequency was set at 11 kHz. Sample temperature was set at approximately 278 K. 1D ^13^C-detected experiments were recorded using a cross-polarization transfer with a contact time of 1 ms and proton decoupling of 85 kHz applied during acquisition time. 2D ^13^C–^13^C experiments were recorded using a proton-driven spin pulse sequence diffusion with a mixing time of 50 ms. Acquisition times were set to 10 ms and 20 s in indirect and direct dimensions, respectively, for a recycle delay of 3 s, leading to a total experimental time of approximately 4 days.

## Supporting information

S1 Fig Type III secretion profile of the different PrgI mutants. Three representative western blots for the *S*. Typhimurium strain and one for each one of the mutants is shown. Individual substrates identified in the immunoblot were cropped and subsequently placed in an order representing the hierarchy of secretion (i.e., early, middle, and late). The uncropped blots that serve as the source for this figure can be found in S11 through S21 Data. WT, wild-type.
(TIF)

S2 Fig Bar graphs detailing the relative abundance of individual substrates secreted by the different PrgI alanine mutants as indicated. The relative abundance of the secreted substrates is the result of standardization to wild type, which was given a value of 1 and is demarcated by a gray dashed line. All values represent the mean ± the standard deviation of three independent experiments. The underlying data for this figure can be found in S22 Data.
(TIF)

S3 Fig Stability of the different PrgI mutant proteins. The indicated mutant proteins were expressed and purified as indicated in Materials and methods and analyzed by western immunoblot. The yield and stability of the different mutant proteins was equivalent to wild type. Some western blot lanes were spliced and arranged into a single image for presentation purposes but were original from different blots developed at the same time, under the same conditions, and scanned together to minimize differences in intensity resulting from methodological variance.
(TIF)

S4 Fig PrgI–SipD signal relay interface model based on alanine mutations resulting in a constitutive secretion phenotype. In the middle is a cartoon depicting a single PrgI subunit (green), which would be at the top of the needle, flanked by two SipD subunits (represented as blue ovals) at the *i* + 6 and *i* + 5 positions. To clearly indicate all the interface residues (shown as spheres), the same PrgI subunit was rotated and flipped (approximately 90°) to show the *i* + 6 interface residues (D11, V20, S49, E53, L56, N78) on the left-hand side and the *i* + 5 interface residues (D10, L31, N55, R58, N63) on the right-hand side. A black dashed semicircular line demarcates the lumen.
(TIF)

S5 Fig Invasion of Henle-407 cells by WT *S*. Typhimurium and strains expressing the indicated PrgI mutants. Values represented the percentage of the inoculum that survived antibiotic treatment as a consequence of bacterial internalization and are the mean ± standard deviation of three independent experiments normalized to WT, which was set to 100%. The underlying data for this figure can be found in S23 Data. WT, wild-type.
(TIF)

S6 Fig Calculated electrostatic surface potentials of various lumen mutants. Cut-away view cartoons of the needle depicting both the solvent accessible surface area and electrostatic surface potential of the lumen. Red is negative, blue is positive, and white is neutral.
(TIF)

S7 Fig Cryo-EM reconstruction of purified needle complex. (A) Cryo-EM micrograph of purified needle complexes. (B) Representative 2D class averages of segmented needles from fully assembled needle complexes. cryo-EM, cryo electron microscopy.
(TIF)

S8 Fig Power spectra of needle filaments from fully assembled needle complexes. (A) Averaged power spectrum of all the helix segments boxed out from needle complex micrographs. (B) Averaged power spectrum of map projections. Equator line (*n* = 0; l = 0) and rise line (*n* = 0; l = 17) are indicated with red and yellow lines, respectively. Rise = (pixel size) × (layer-line image size)/(layer-line height) = 1.708 × 4,096/1,650 = 4.24 Å.
(TIF)

S9 Fig Global resolution estimation. FSC curve for maps constructed from two half data sets of PrgI needle filaments boxed out from fully assembled needle complexes. A cutoff value of 0.143 is used. FSC, Fourier shell correlation.
(TIF)

S10 Fig Workflow for the WT needle filament structure calculation. (A) Class distribution and helical symmetry of the four maps from 3D classification. (B) Resolution and helical symmetry of the final map after 3D refinement and post processing. WT, wild-type.
(TIF)

S11 Fig Comparison between the cryo-EM structure of needle filaments boxed out from fully assembled needle complexes and the SSNMR model structure of in vitro polymerized needle filaments. (A) Helical symmetry of an ensemble of 10 SSNMR models from the SSNMR data (PDB: 2LPZ). The tenth model is closest to our model in terms of helical symmetry and highlighted in red. (B) 3D density map of PrgI needle filaments attached to base with fitted atomic model. (C) 3D density map of PrgI needle attached to base with fitted NMR model (PDB: 2LPZ). (D) Superimposition of our model with the SSNMR model (PDB: 2LPZ). cryo-EM, cryo electron microscopy; SSNMR, solid-state NMR; PDB, Protein Data Bank.
(TIF)

S12 Fig Structure calculation workflow for PrgI filaments assembled in vitro. (A) Representative micrograph of negative-stained in vitro polymerized PrgI filaments after sonication. (B) Representative micrograph of the in vitro polymerized PrgI filaments in vitrified ice after sonication. (C) FSC curve for maps constructed from two half data sets of the PrgI filaments. A cutoff value of 0.143 was used. The twist and rise of the final map are 63.35° and 4.25 Å. (D) Selected reference-free 2D class averages. (E) Particle distribution and helical symmetry of the four maps from the 3D classification. (F) Resolution and helical symmetry of the different maps after 3D refinement. FSC, Fourier shell correlation.
(TIF)

S13 Fig Superimposition of the final maps of PrgI needle filaments attached to the base (magenta) on in vitro polymerized (cyan). (TIF)

S14 Fig Structure calculation workflow for PrgI^S49A^ filaments. (A) FSC curve for maps constructed from two half data sets of the PrgI^S49A^ filaments. A cutoff value of 0.143 was used. The twist and rise of the final map generated from 14,070 particles are 63.35° and 4.19 Å. (B) Selected reference-free 2D class averages. (C) Particle distribution and helical symmetry of the four maps from the 3D classification. (D) Resolution and helical symmetry of the final map after 3D refinement. (E) 3D density map of the PrgI^S49A^ filaments with fitted atomic model. FSC, Fourier shell correlation.
(TIF)

S15 Fig Structure calculation workflow for PrgI^V67A^ filaments. (A) FSC curve for maps constructed from two half data sets of the PrgI^V67A^ filaments. A cutoff value of 0.143 was used. The twist and rise of the final map generated from 22,143 particles are 63.27° and 4.24 Å. (B) Selected reference-free 2D class averages. (C) Particle distribution and helical symmetry of the four maps from the 3D classification. (D) Resolution and helical symmetry of the maps after 3D refinement. (E) 3D density map of the PrgI^V67A^ filaments with the fitted atomic model. FSC, Fourier shell correlation.
(TIF)

S16 Fig Alignment of one PrgI subunits extracted from PrgI^WT^ (cyan), PrgI^S49A^ (green), and PrgIV67 (white) filaments. WT, wild-type.
(TIF)

S17 Fig Close-up view of the electrostatic potential map of PrgI^WT^ filaments showing the charged residues in the lumen. WT, wild-type.
(TIF)

S18 Fig Cross-section view of the lumen of needle filament structures assembled with WT PrgI (grey), PrgI^S49A^ (blue), and PrgI^V67A^ (yellow). The lumen residues K66, K69, D70, A73, A74, Q77, N78, and R80 are indicated. WT, wild-type.
(TIF)

S19 Fig Electrostatic potential maps of the lumen of PrgIV67A subunits fitted in PrgIWT map. WT, wild-type.
(TIF)

S20 Fig Calculated solvent-accessible surface area of a PrgI subunit in a needle. The percentage of solvent exposure is represented in the bar graph with respect to the residues of PrgI. The underlying data for this figure can be found in S24 Data.
(TIF)

S1 Data Numerical values Fig 1. (XLSX)

S2 Data Numerical values Fig 2A. (XLSX)

S3 Data Numerical values Fig 2C. (XLSX)

S4 Data Numerical values Fig 4A. (XLSX)

S5 Data Numerical values Fig 5B. (XLSX)

S6 Data Numerical values Fig 5E. (XLSX)

S7 Data Numerical values Fig 6A. (XLSX)

S8 Data Numerical values Fig 6B. (XLSX)

S9 Data Numerical values Fig 6C. (XLSX)

S10 Data Numerical values Fig 6D. (XLSX)

S11 Data Uncropped blots for S1 Fig. (ZIP)

S12 Data Uncropped blots for S1 Fig. (ZIP)

S13 Data Uncropped blots for S1 Fig. (ZIP)

S14 Data Uncropped blots for S1 Fig. (ZIP)

S15 Data Uncropped blots for S1 Fig. (ZIP)

S16 Data Uncropped blots for S1 Fig. (ZIP)

S17 Data Uncropped blots for S1 Fig. (ZIP)

S18 Data Uncropped blots for S1 Fig. (ZIP)

S19 Data Uncropped blots for S1 Fig. (ZIP)

S20 Data Uncropped blots for S1 Fig. (ZIP)

S21 Data Uncropped blots for S1 Fig. (ZIP)

S22 Data Numerical values S2 Fig. (XLSX)

S23 Data Numerical values S5 Fig. (XLSX)

S24 Data Numerical values S20 Fig. (XLSX)

S1 Table (PDF)

S2 Table (PDF)

## Abbreviations

ASM: alanine-scanning mutagenesis
cryo-EM: cryo electron microscopy
FSC: Fourier shell correlation
MAS-SSNMR: magic-angle spinning solid-state NMR
RMSD: root mean square deviation
SPI: pathogenicity island
SSNMR: solid-state NMR
T3SS: type III secretion system
