# Supplementary figures and images for: A polymorphic helix of a Salmonella needle protein relays signals defining distinct steps in type III secretion (part 1 of 3)
Source: PLoS Biol. 2019 Jul 1;17(7):e3000351. doi: 10.1371/journal.pbio.3000351 (PMC6625726; doi:10.1371/journal.pbio.3000351)

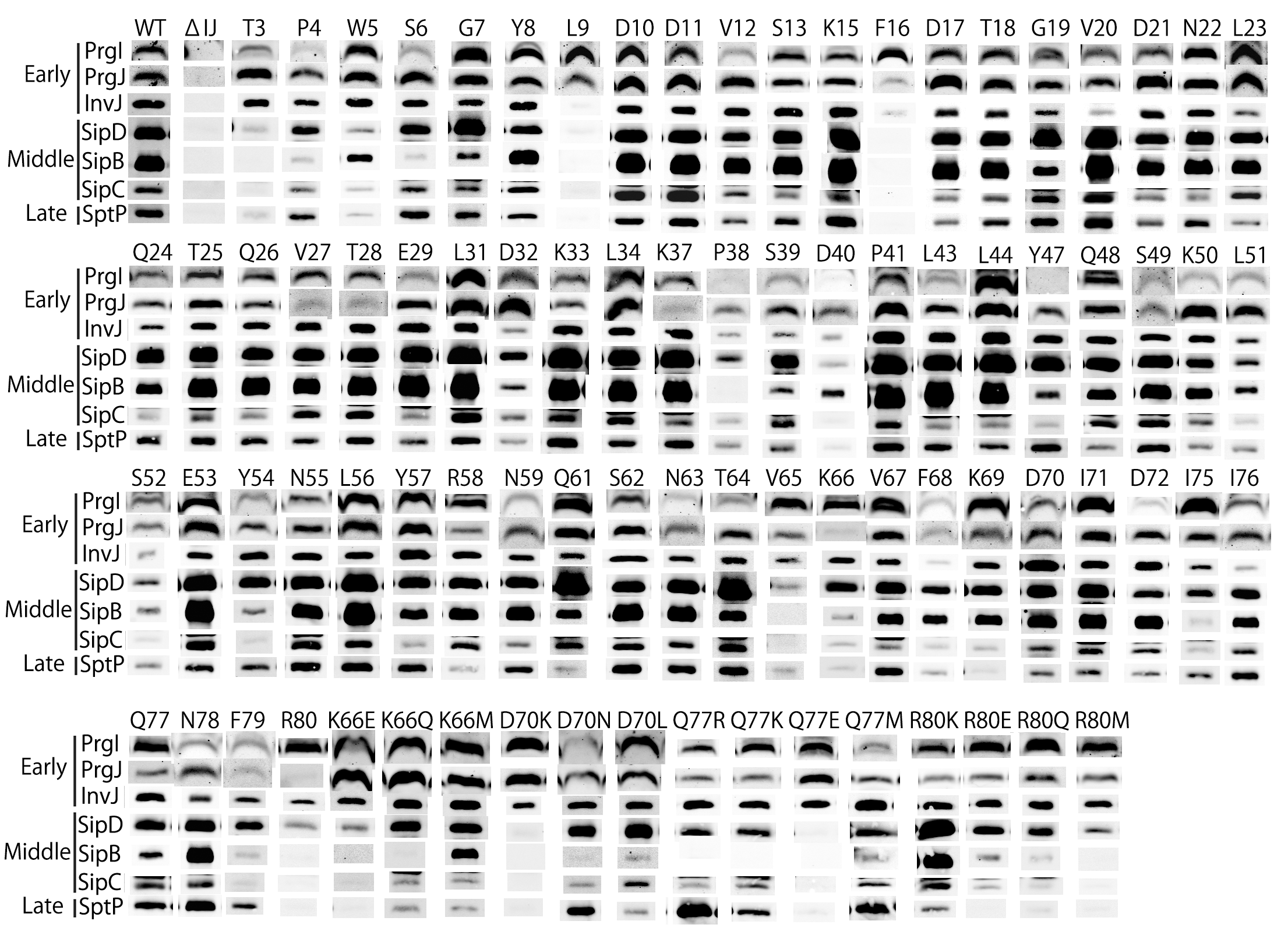

Supplement: S1 Fig — Three representative western blots for the S. Typhimurium strain and one for each one of the mutants is shown. Individual substrates identified in the immunoblot were cropped and subsequently placed in an order representing the hierarchy of secretion (i.e., early, middle, and late). The uncropped blots that serve as the source for this figure can be found in S11 through S21 Data. WT, wild-type. (TIF) [file pbio.3000351.s001.tif]

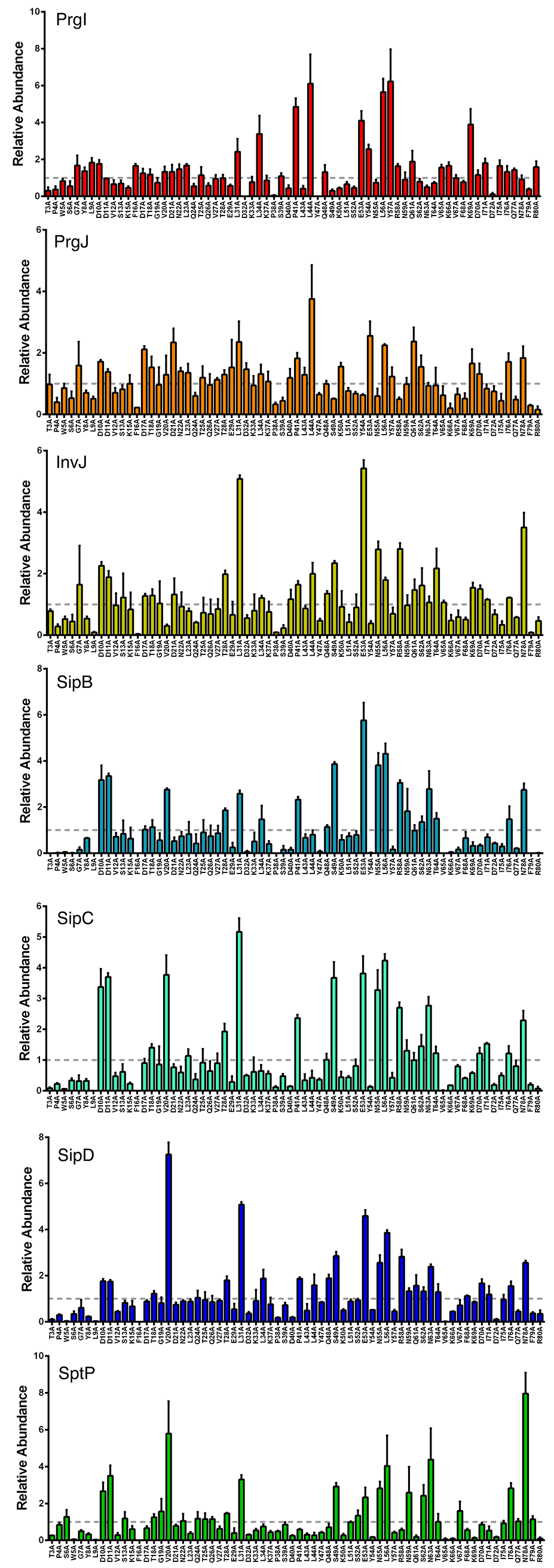

Supplement: S2 Fig — The relative abundance of the secreted substrates is the result of standardization to wild type, which was given a value of 1 and is demarcated by a gray dashed line. All values represent the mean ± the standard deviation of three independent experiments. The underlying data for this figure can be found in S22 Data. (TIF) [file pbio.3000351.s002.tif]

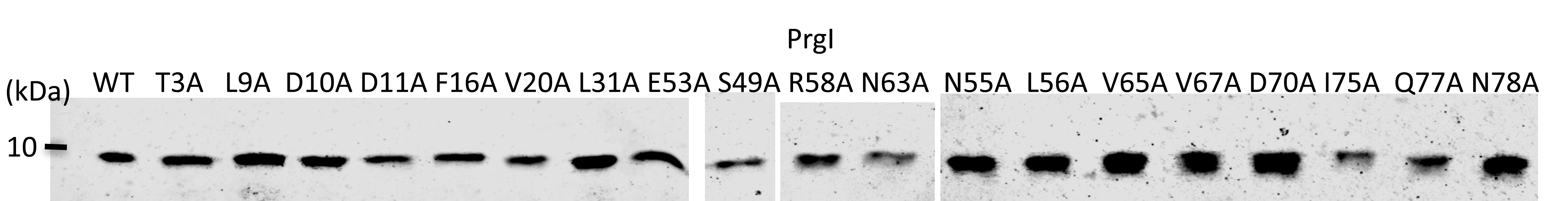

Supplement: S3 Fig — The indicated mutant proteins were expressed and purified as indicated in Materials and methods and analyzed by western immunoblot. The yield and stability of the different mutant proteins was equivalent to wild type. Some western blot lanes were spliced and arranged into a single image for presentation purposes but were original from different blots developed at the same time, under the same conditions, and scanned together to minimize differences in intensity resulting from methodological variance. (TIF) [file pbio.3000351.s003.tif]

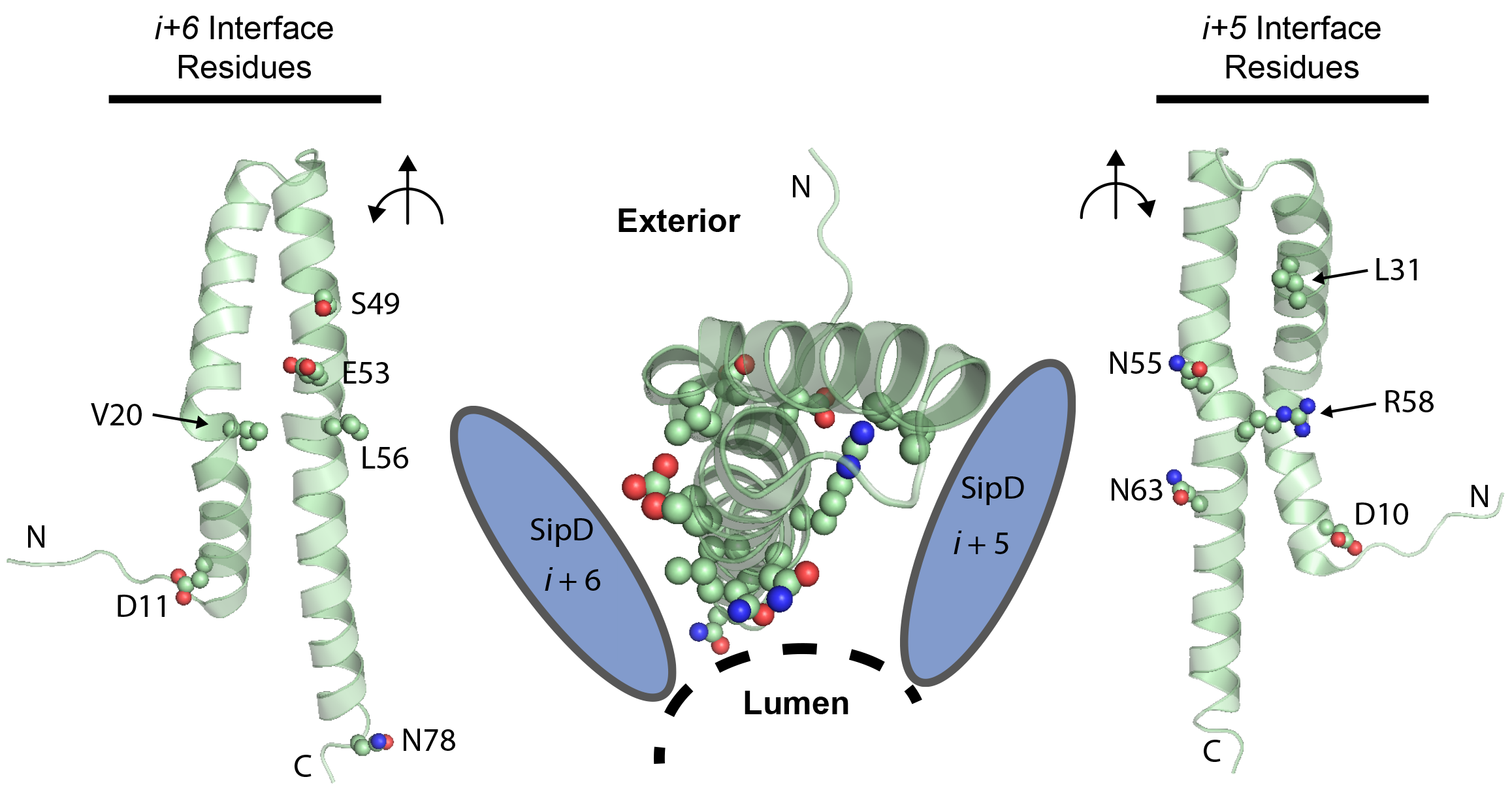

Supplement: S4 Fig — In the middle is a cartoon depicting a single PrgI subunit (green), which would be at the top of the needle, flanked by two SipD subunits (represented as blue ovals) at the i + 6 and i + 5 positions. To clearly indicate all the interface residues (shown as spheres), the same PrgI subunit was rotated and flipped (approximately 90°) to show the i + 6 interface residues (D11, V20, S49, E53, L56, N78) on the left-hand side and the i + 5 interface residues (D10, L31, N55, R58, N63) on the right-hand side. A black dashed semicircular line demarcates the lumen. (TIF) [file pbio.3000351.s004.tif]

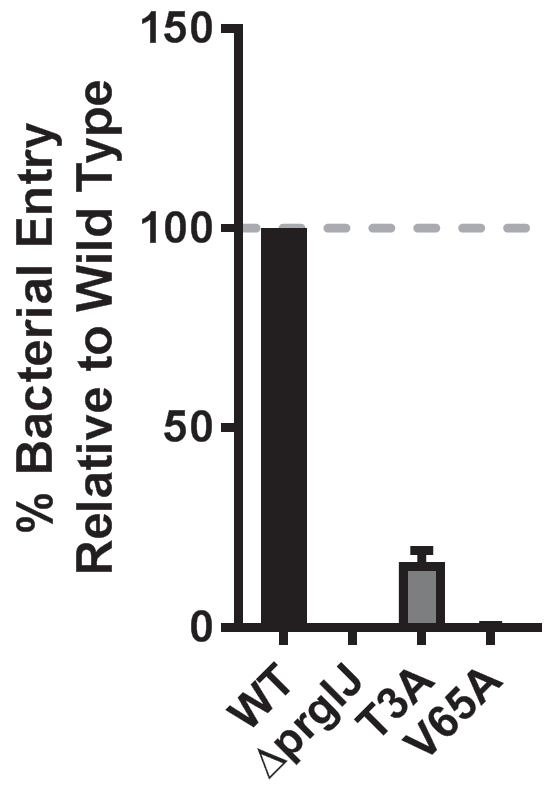

Supplement: S5 Fig — Values represented the percentage of the inoculum that survived antibiotic treatment as a consequence of bacterial internalization and are the mean ± standard deviation of three independent experiments normalized to WT, which was set to 100%. The underlying data for this figure can be found in S23 Data. WT, wild-type. (TIF) [file pbio.3000351.s005.tif]

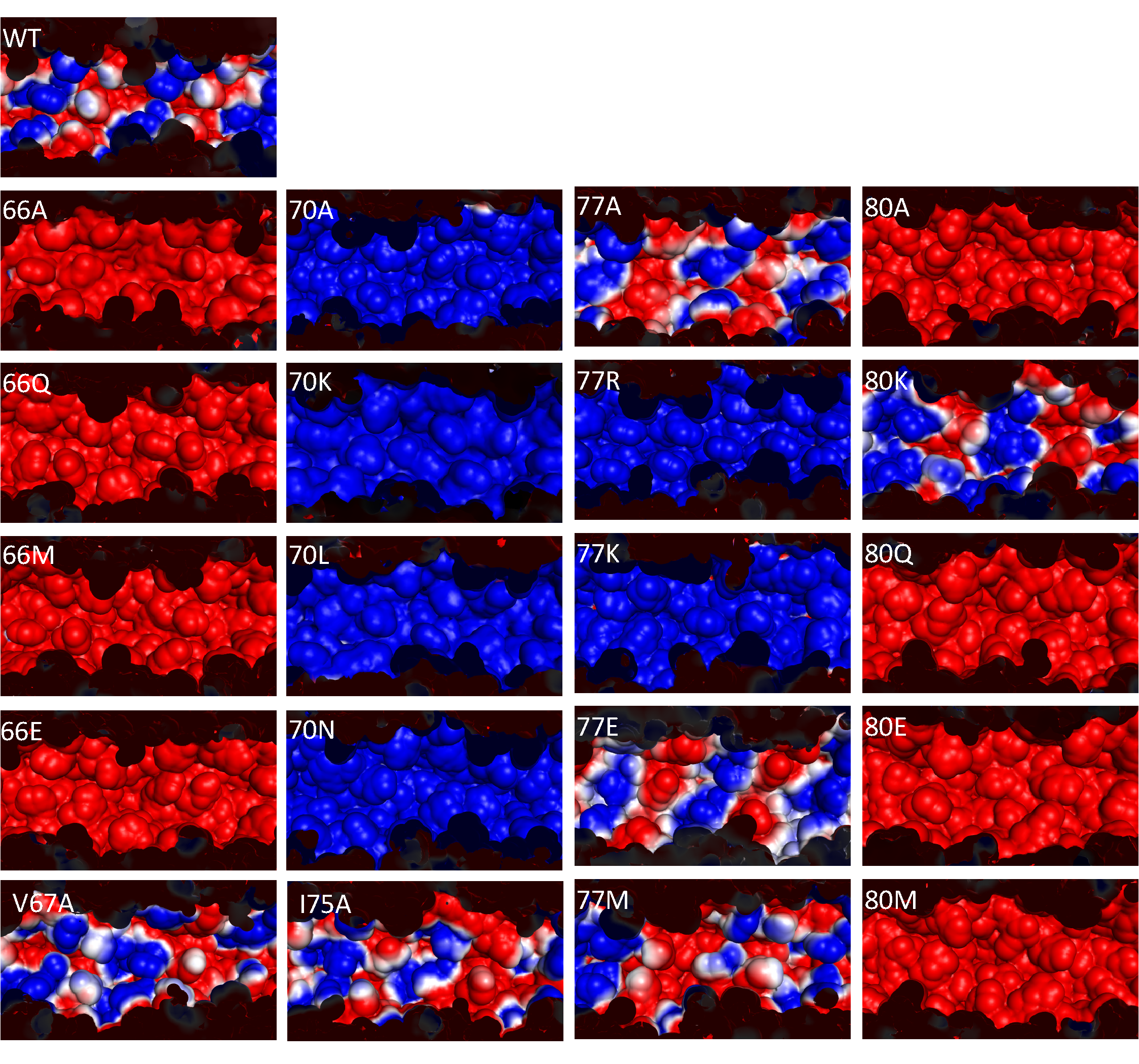

Supplement: S6 Fig — Cut-away view cartoons of the needle depicting both the solvent accessible surface area and electrostatic surface potential of the lumen. Red is negative, blue is positive, and white is neutral. (TIF) [file pbio.3000351.s006.tif]

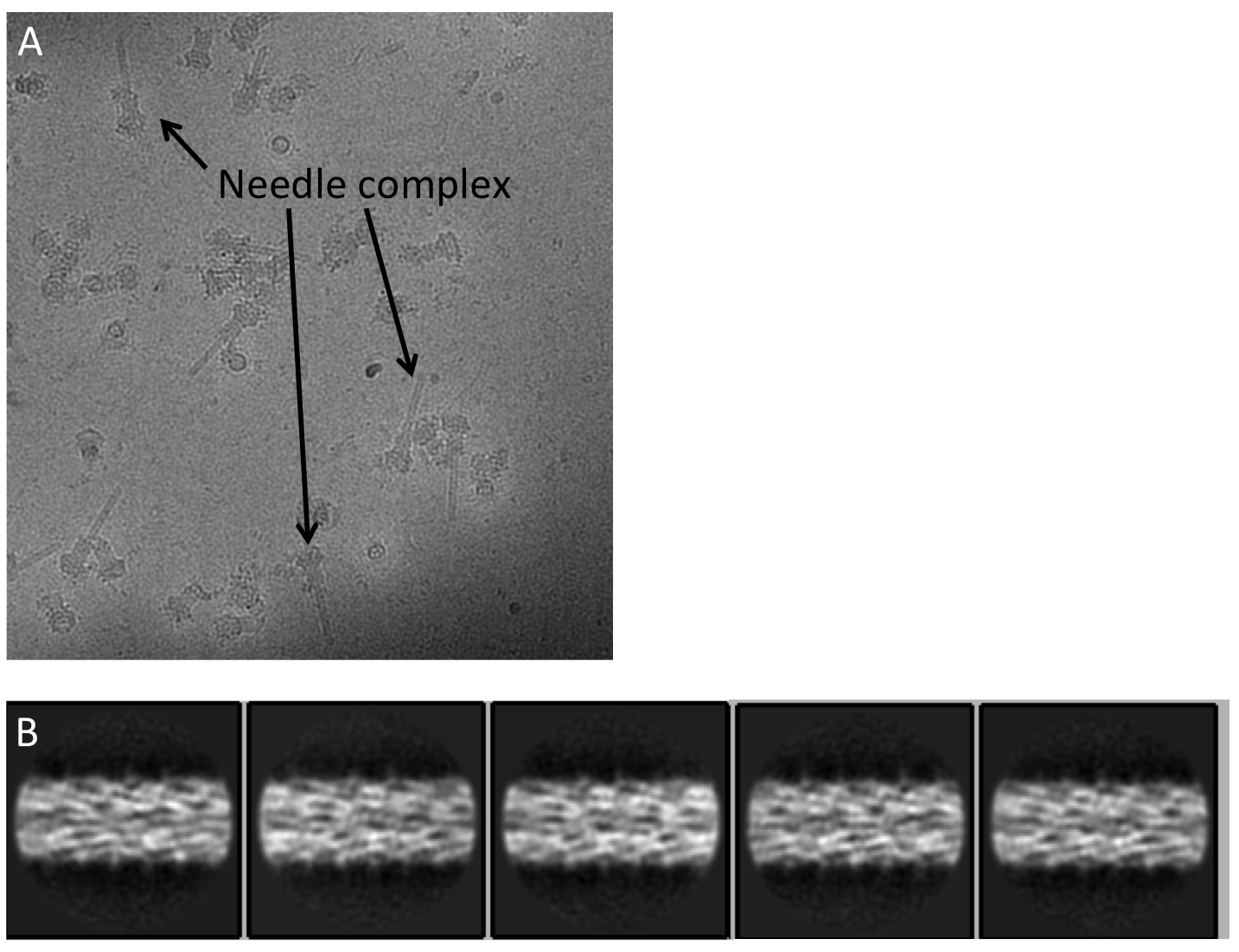

Supplement: S7 Fig — (A) Cryo-EM micrograph of purified needle complexes. (B) Representative 2D class averages of segmented needles from fully assembled needle complexes. cryo-EM, cryo electron microscopy. (TIF) [file pbio.3000351.s007.tif]

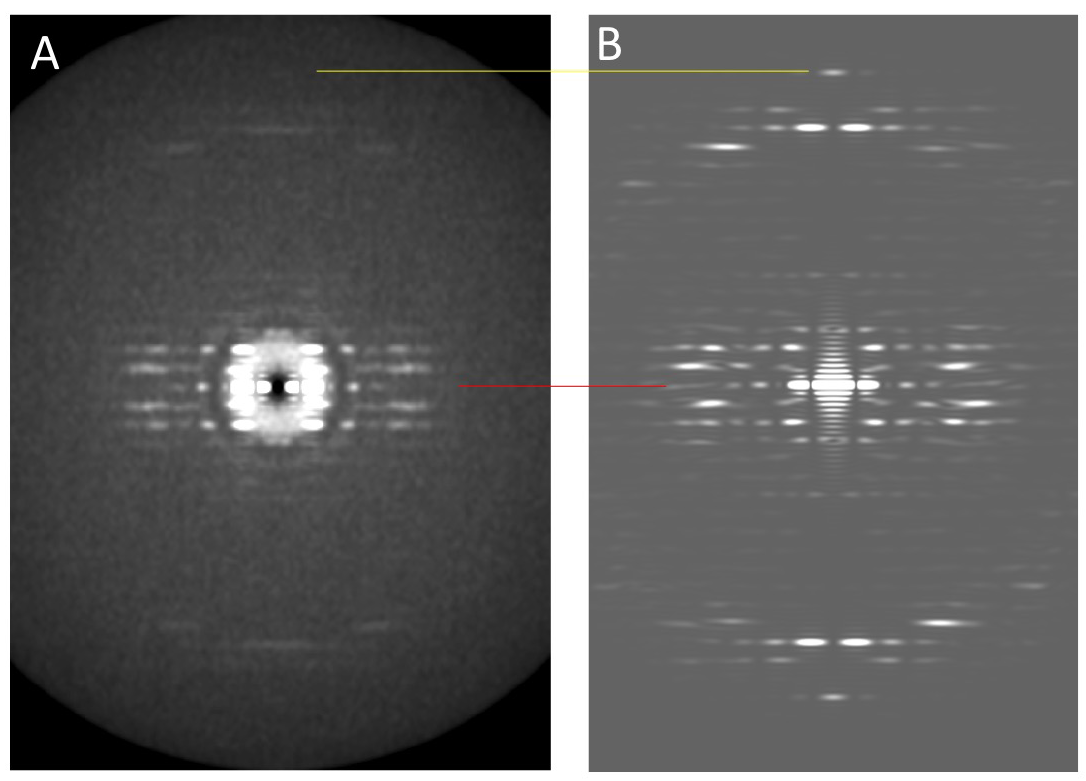

Supplement: S8 Fig — (A) Averaged power spectrum of all the helix segments boxed out from needle complex micrographs. (B) Averaged power spectrum of map projections. Equator line (n = 0; l = 0) and rise line (n = 0; l = 17) are indicated with red and yellow lines, respectively. Rise = (pixel size) × (layer-line image size)/(layer-line height) = 1.708 × 4,096/1,650 = 4.24 Å. (TIF) [file pbio.3000351.s008.tif]

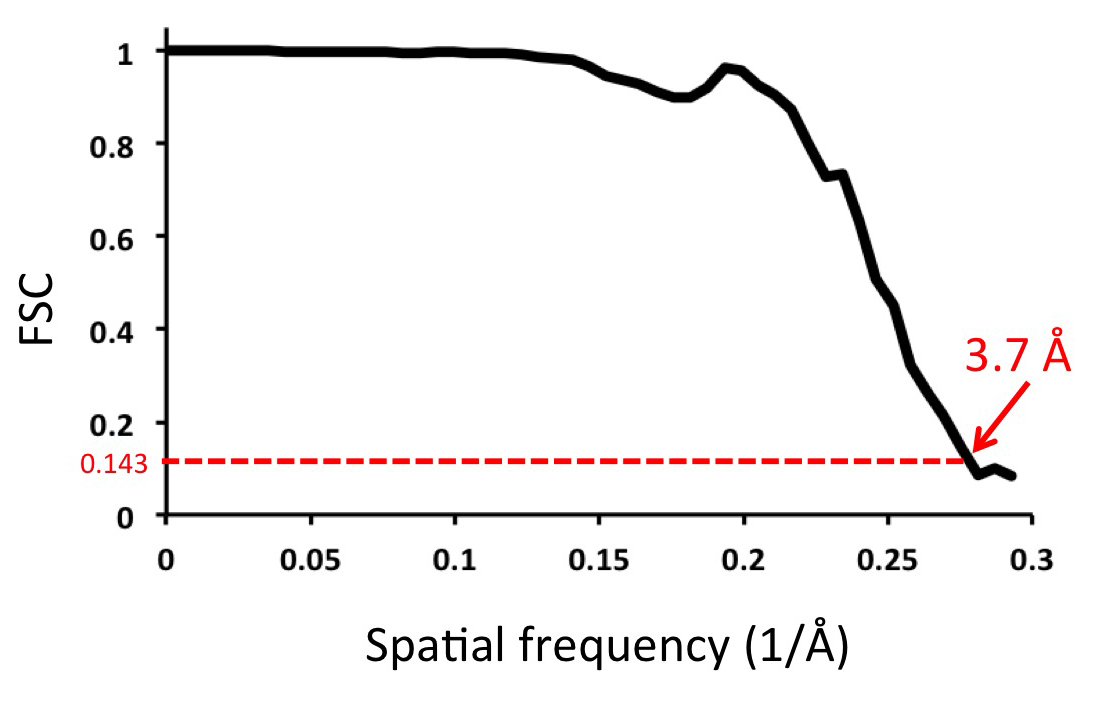

Supplement: S9 Fig — FSC curve for maps constructed from two half data sets of PrgI needle filaments boxed out from fully assembled needle complexes. A cutoff value of 0.143 is used. FSC, Fourier shell correlation. (TIF) [file pbio.3000351.s009.tif]

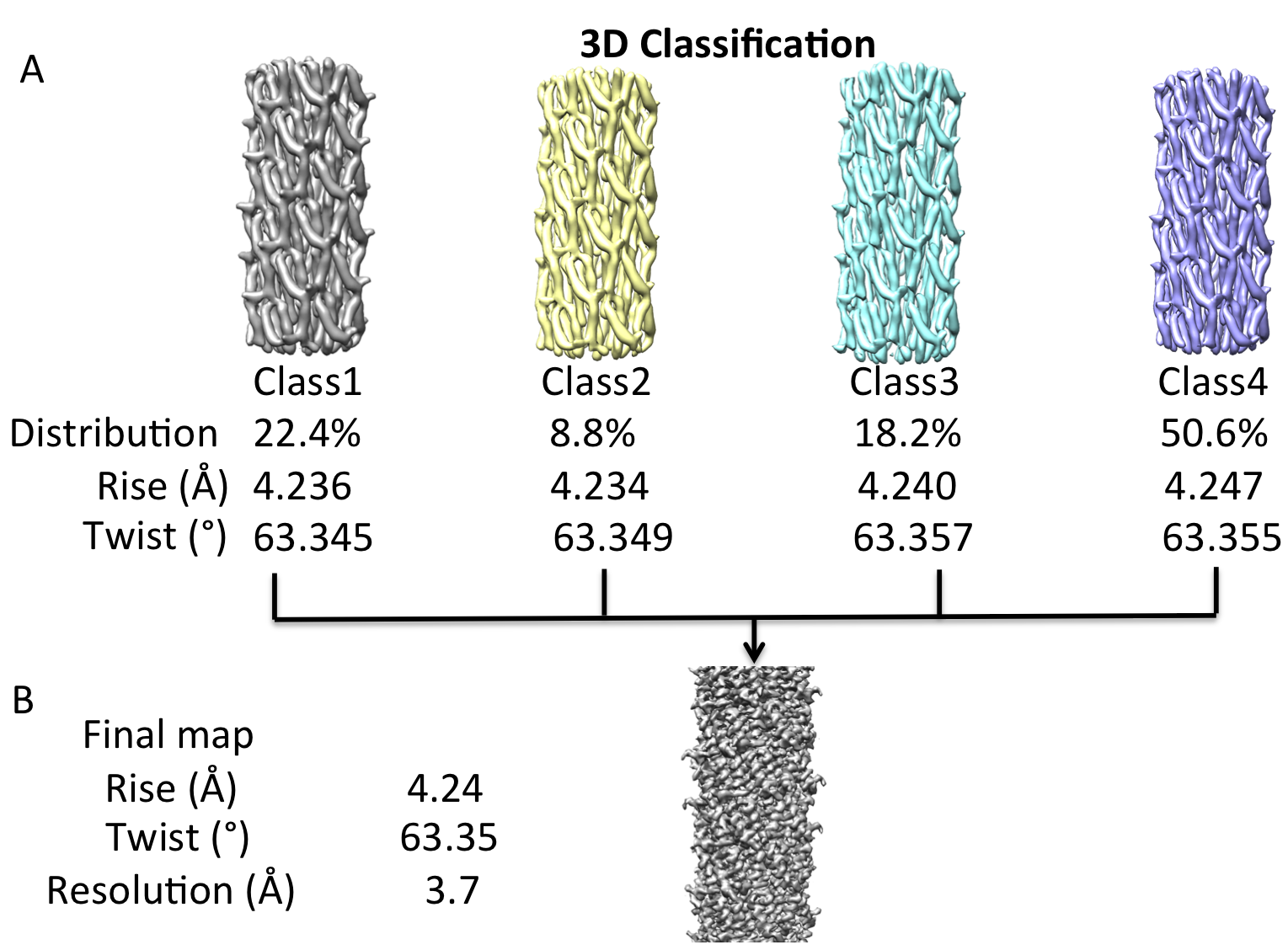

Supplement: S10 Fig — (A) Class distribution and helical symmetry of the four maps from 3D classification. (B) Resolution and helical symmetry of the final map after 3D refinement and post processing. WT, wild-type. (TIF) [file pbio.3000351.s010.tif]

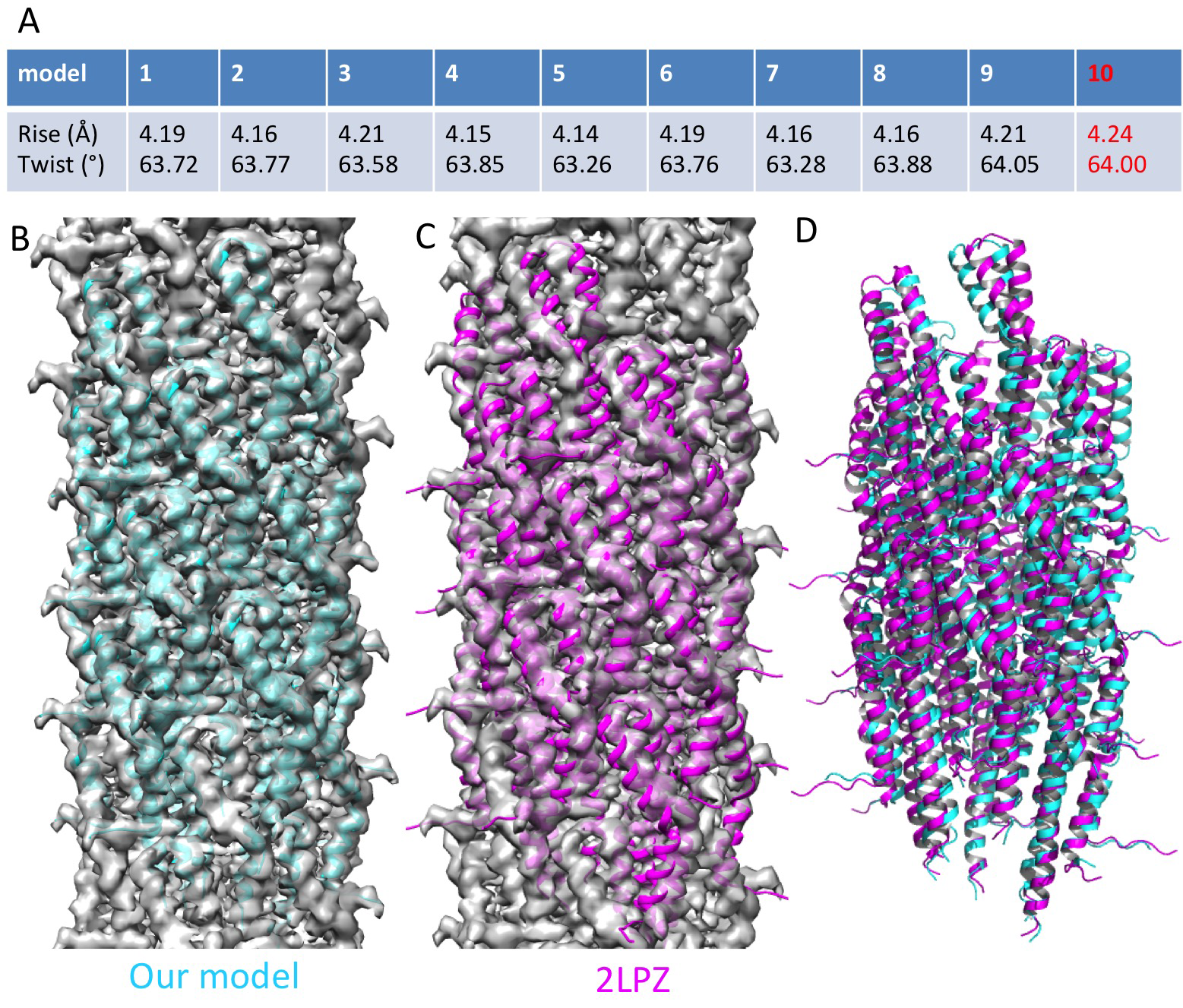

Supplement: S11 Fig — (A) Helical symmetry of an ensemble of 10 SSNMR models from the SSNMR data (PDB: 2LPZ). The tenth model is closest to our model in terms of helical symmetry and highlighted in red. (B) 3D density map of PrgI needle filaments attached to base with fitted atomic model. (C) 3D density map of PrgI needle attached to base with fitted NMR model (PDB: 2LPZ). (D) Superimposition of our model with the SSNMR model (PDB: 2LPZ). cryo-EM, cryo electron microscopy; SSNMR, solid-state NMR; PDB, Protein Data Bank. (TIF) [file pbio.3000351.s011.tif]

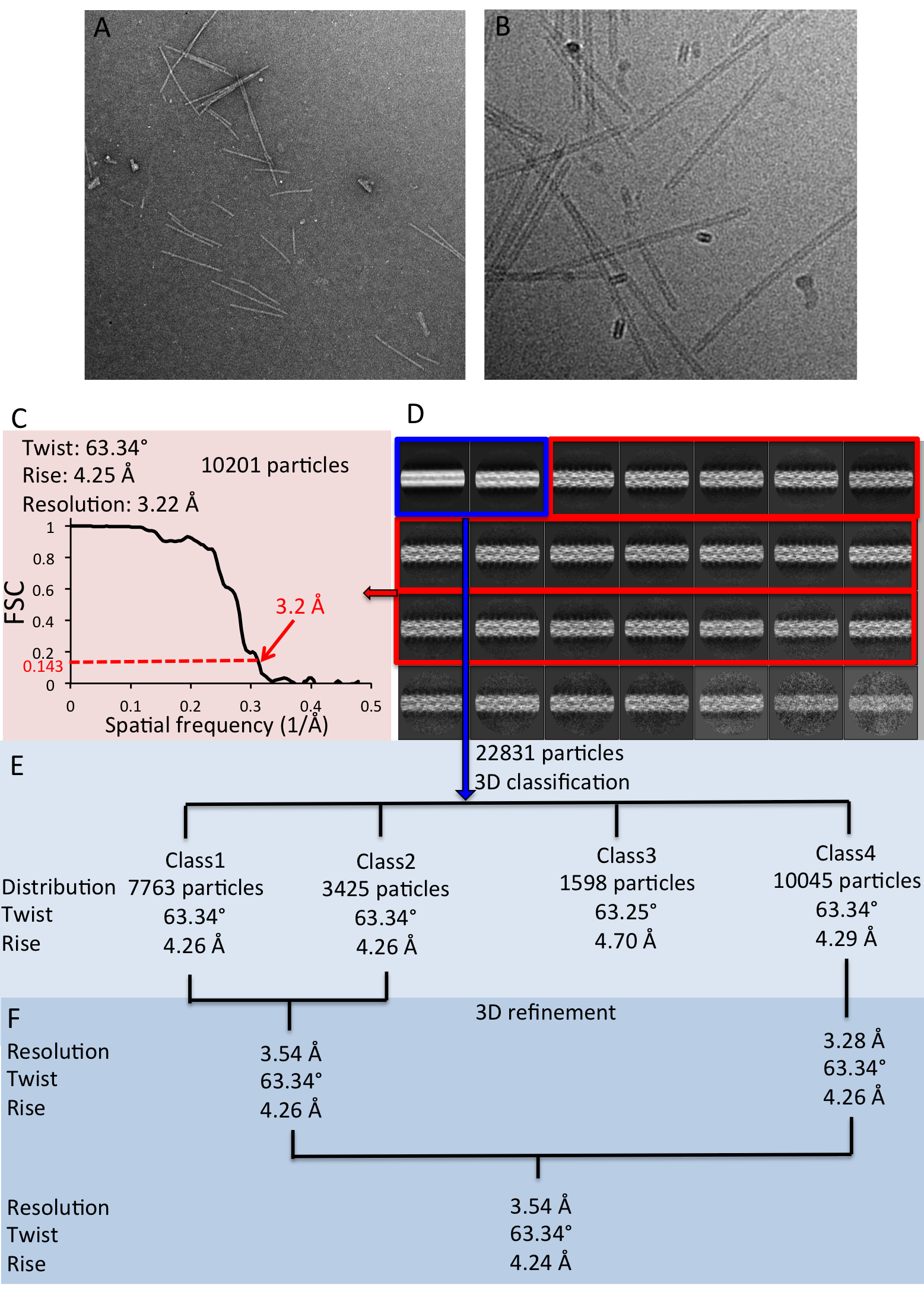

Supplement: S12 Fig — (A) Representative micrograph of negative-stained in vitro polymerized PrgI filaments after sonication. (B) Representative micrograph of the in vitro polymerized PrgI filaments in vitrified ice after sonication. (C) FSC curve for maps constructed from two half data sets of the PrgI filaments. A cutoff value of 0.143 was used. The twist and rise of the final map are 63.35° and 4.25 Å. (D) Selected reference-free 2D class averages. (E) Particle distribution and helical symmetry of the four maps from the 3D classification. (F) Resolution and helical symmetry of the different maps after 3D refinement. FSC, Fourier shell correlation. (TIF) [file pbio.3000351.s012.tif]

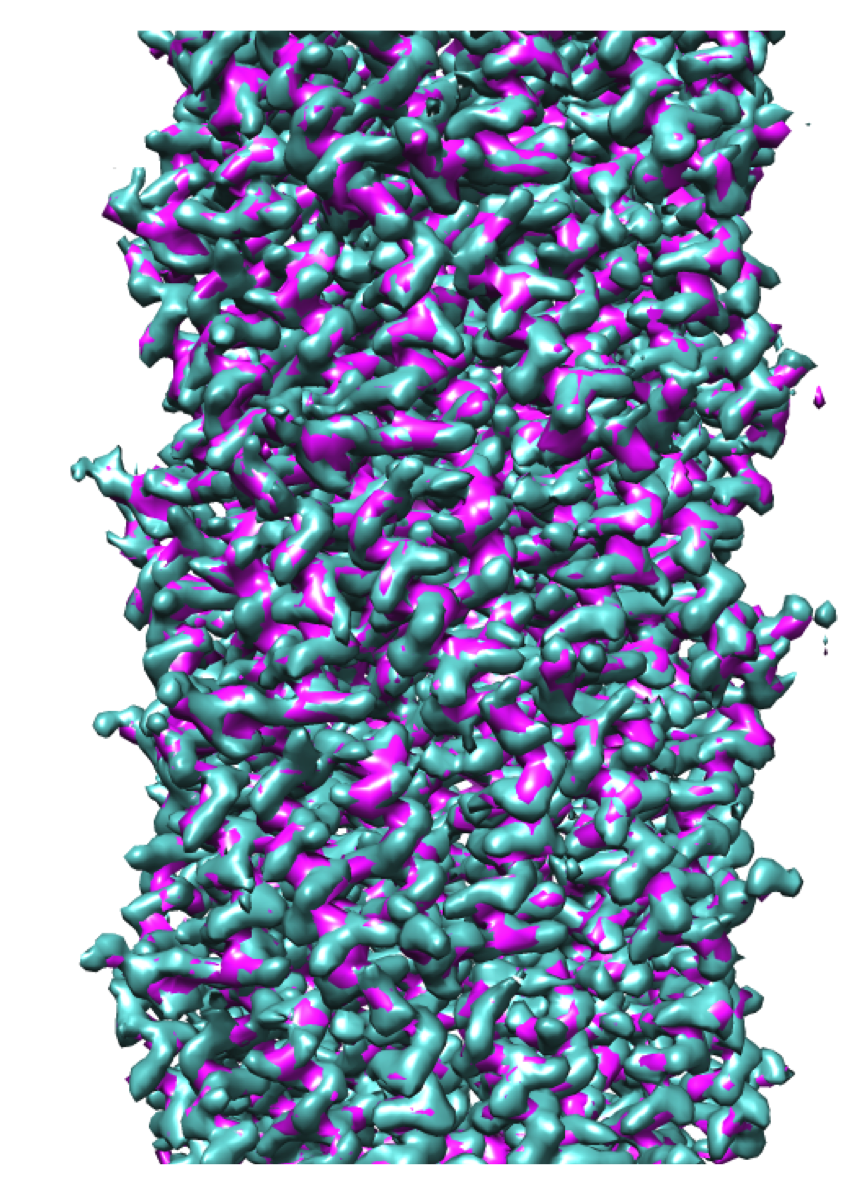

Supplement: S13 Fig — (TIF) [file pbio.3000351.s013.tif]

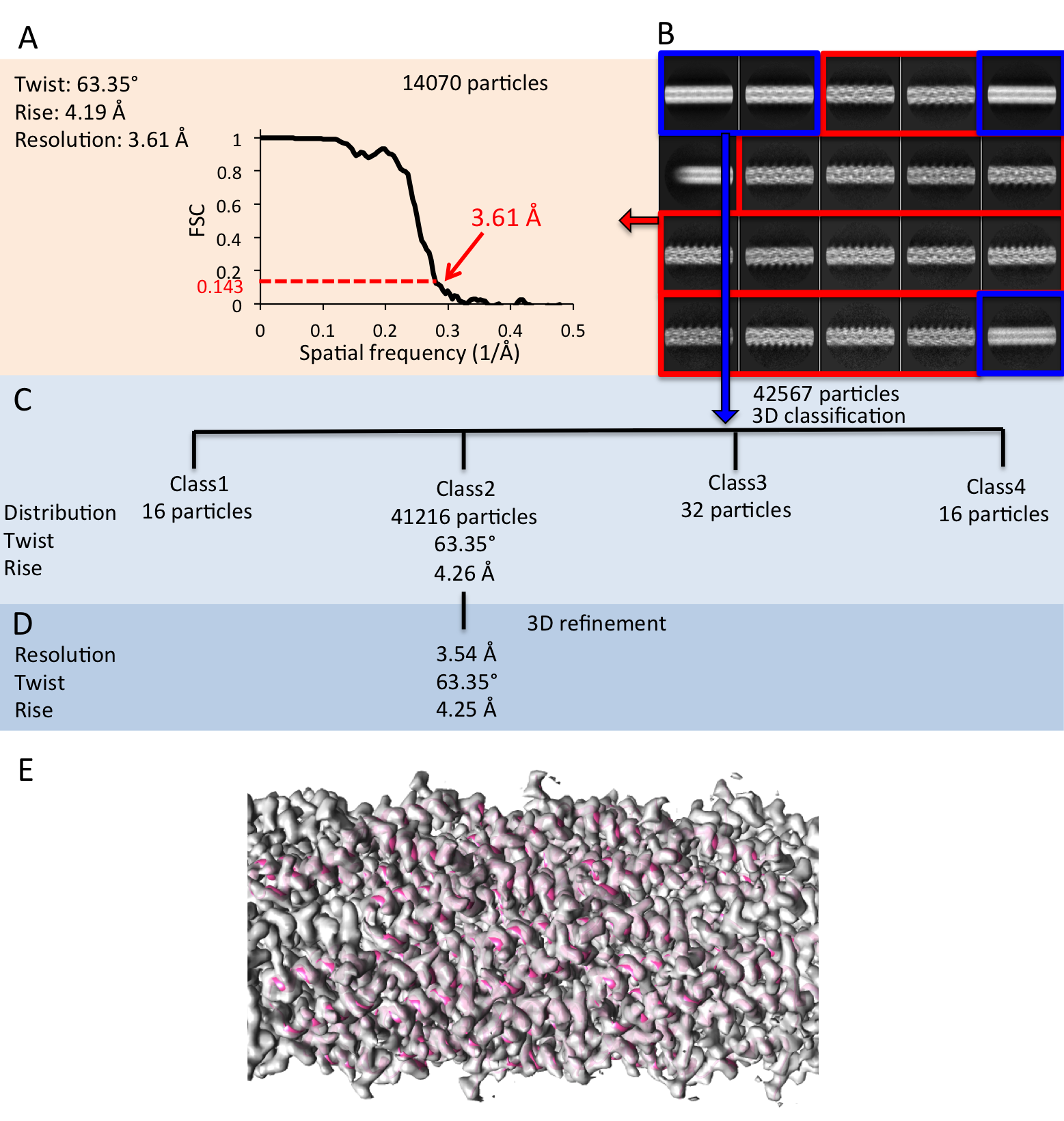

Supplement: S14 Fig — (A) FSC curve for maps constructed from two half data sets of the PrgIS49A filaments. A cutoff value of 0.143 was used. The twist and rise of the final map generated from 14,070 particles are 63.35° and 4.19 Å. (B) Selected reference-free 2D class averages. (C) Particle distribution and helical symmetry of the four maps from the 3D classification. (D) Resolution and helical symmetry of the final map after 3D refinement. (E) 3D density map of the PrgIS49A filaments with fitted atomic model. FSC, Fourier shell correlation. (TIF) [file pbio.3000351.s014.tif]

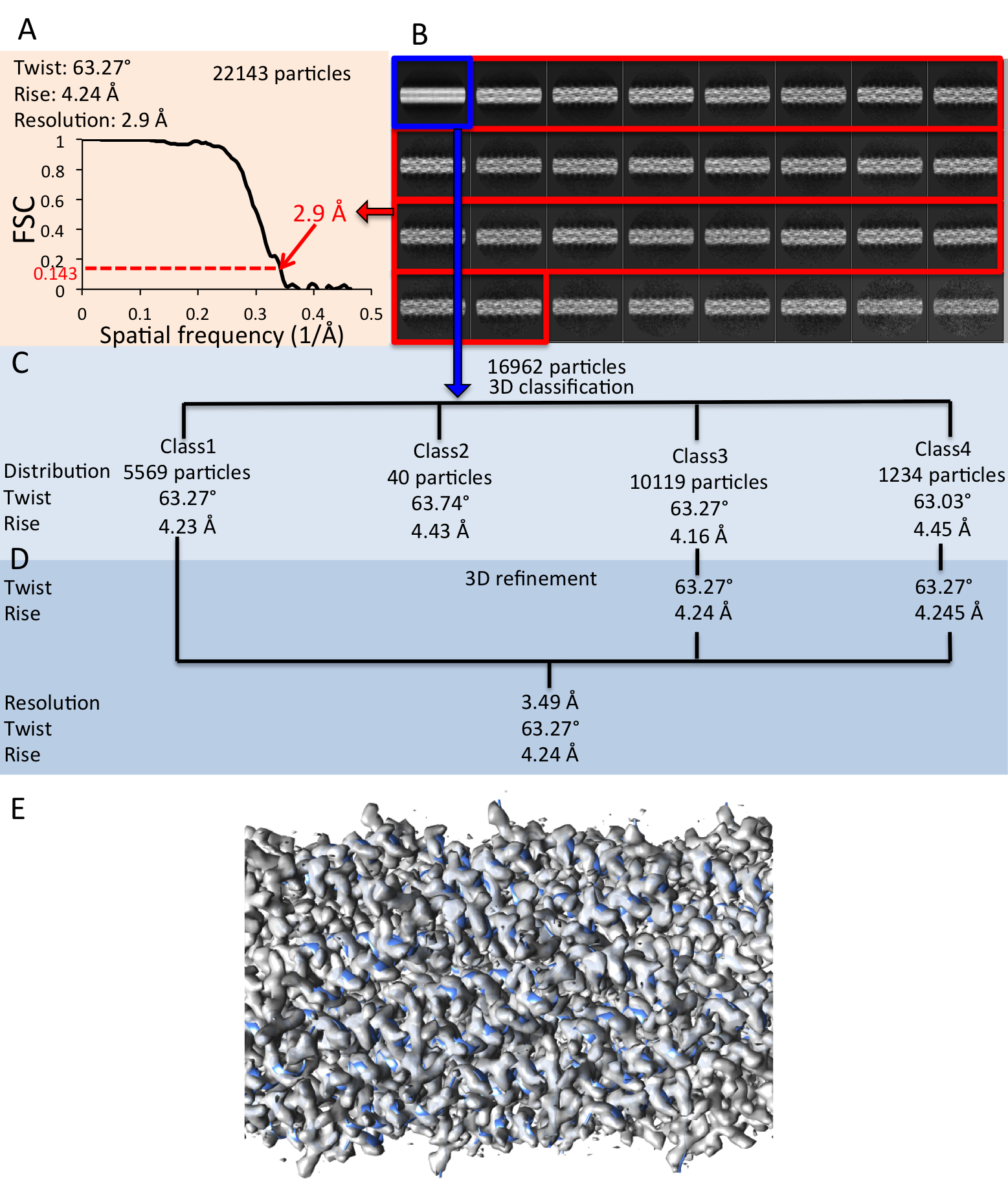

Supplement: S15 Fig — (A) FSC curve for maps constructed from two half data sets of the PrgIV67A filaments. A cutoff value of 0.143 was used. The twist and rise of the final map generated from 22,143 particles are 63.27° and 4.24 Å. (B) Selected reference-free 2D class averages. (C) Particle distribution and helical symmetry of the four maps from the 3D classification. (D) Resolution and helical symmetry of the maps after 3D refinement. (E) 3D density map of the PrgIV67A filaments with the fitted atomic model. FSC, Fourier shell correlation. (TIF) [file pbio.3000351.s015.tif]

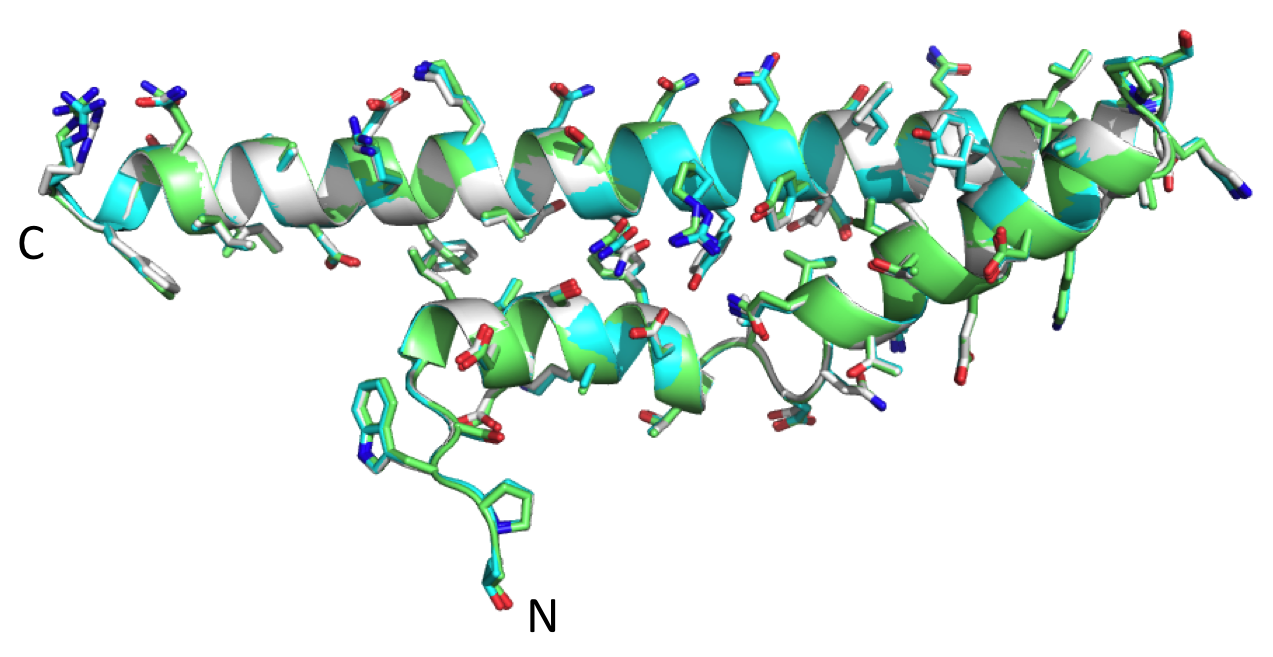

Supplement: S16 Fig — WT, wild-type. (TIF) [file pbio.3000351.s016.tif]

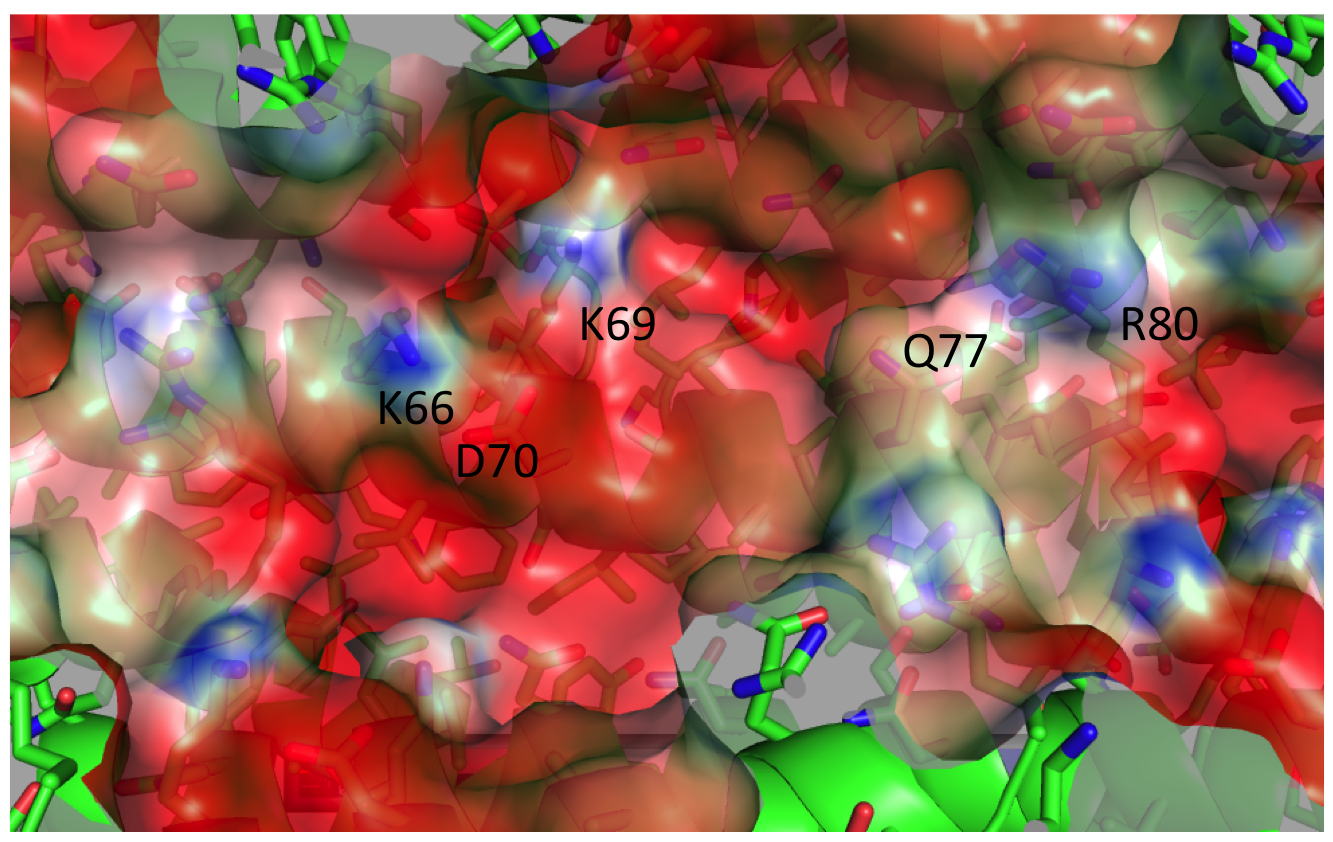

Supplement: S17 Fig — WT, wild-type. (TIF) [file pbio.3000351.s017.tif]

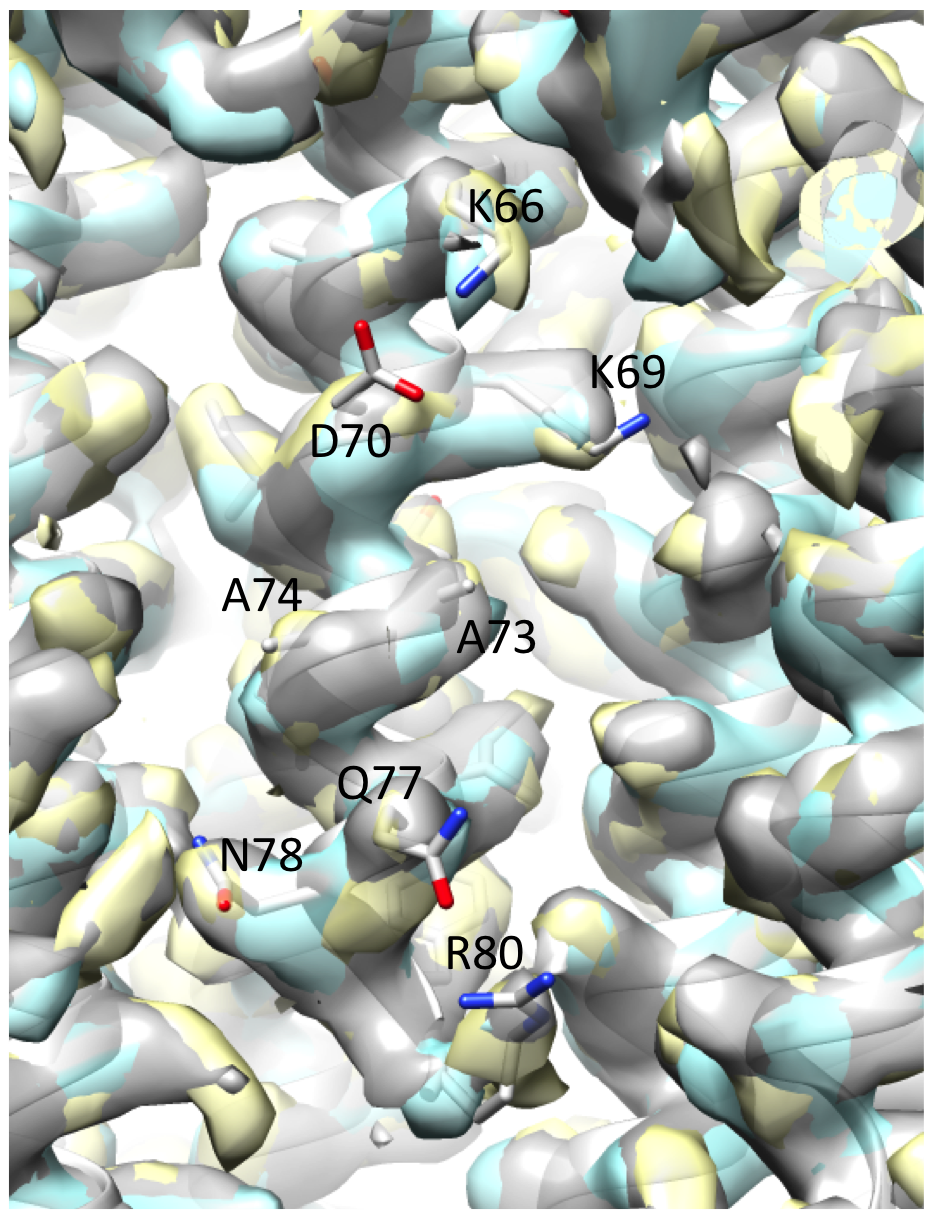

Supplement: S18 Fig — The lumen residues K66, K69, D70, A73, A74, Q77, N78, and R80 are indicated. WT, wild-type. (TIF) [file pbio.3000351.s018.tif]

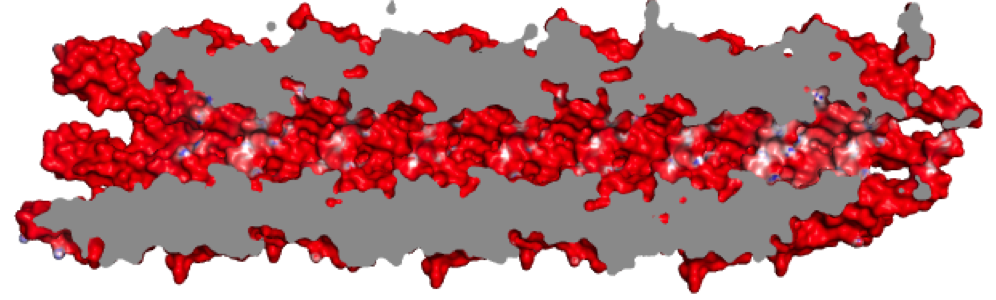

Supplement: S19 Fig — WT, wild-type. (TIF) [file pbio.3000351.s019.tif]

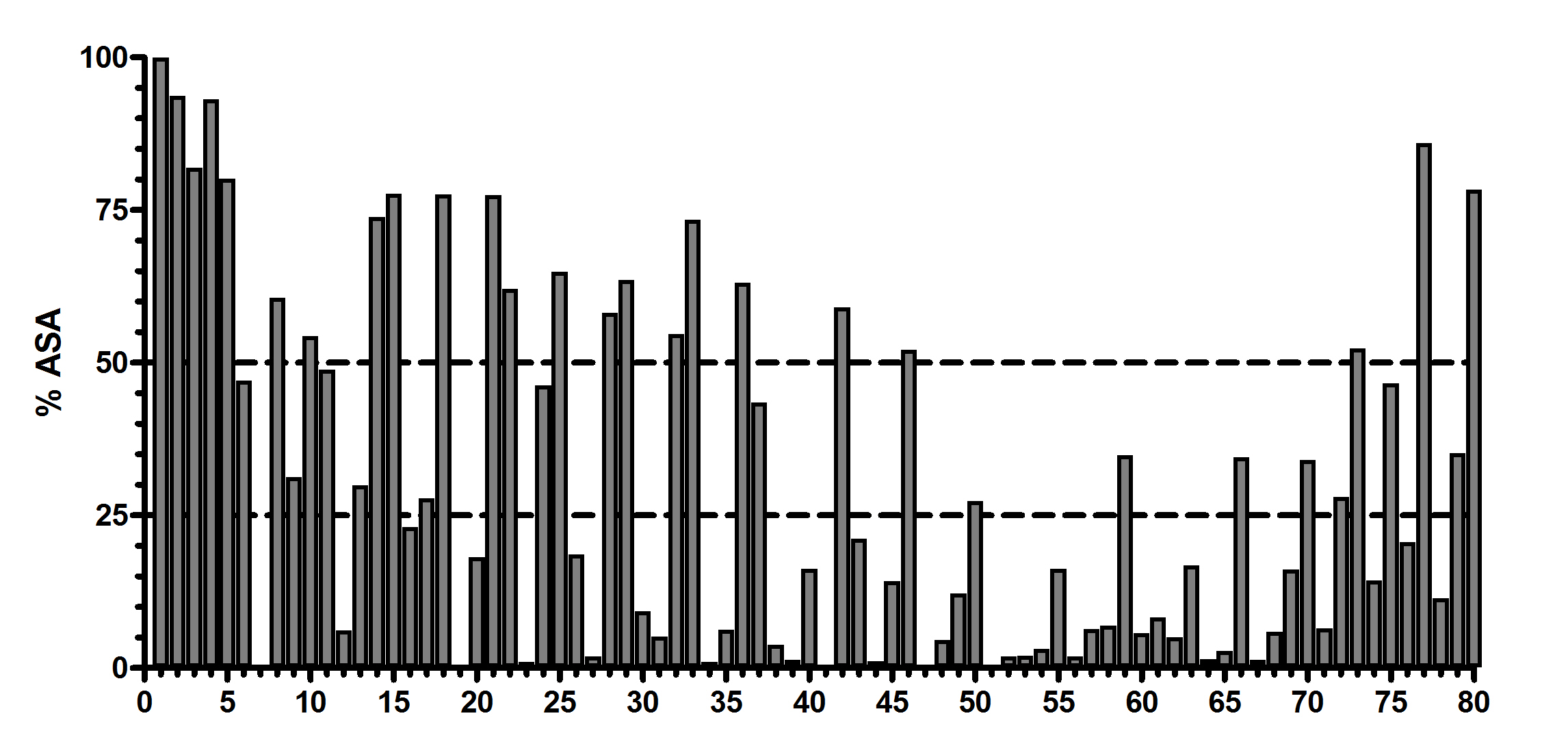

Supplement: S20 Fig — The percentage of solvent exposure is represented in the bar graph with respect to the residues of PrgI. The underlying data for this figure can be found in S24 Data. (TIF) [file pbio.3000351.s020.tif]

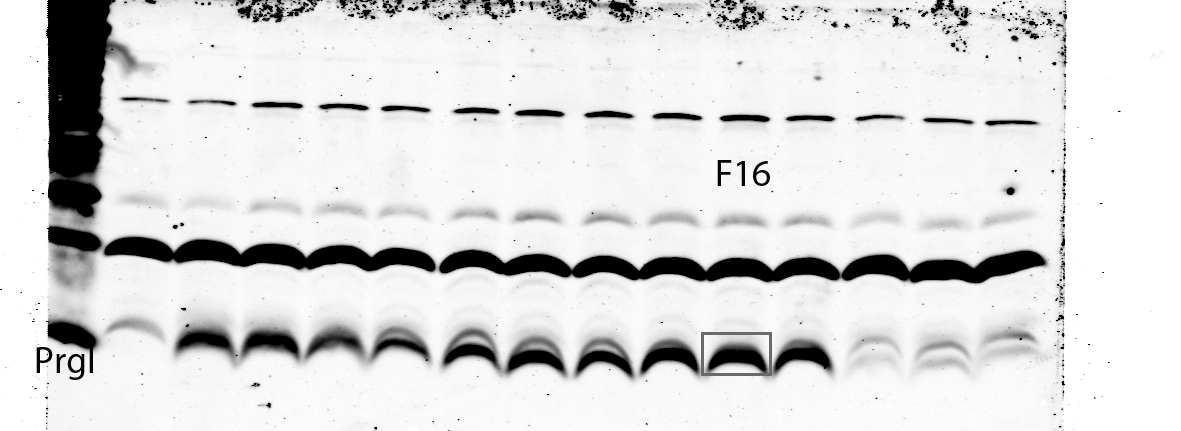

Supplement: S13 Data — (ZIP) [file pbio.3000351.s033.zip › S13-data/F16-anti-prgI.tif]

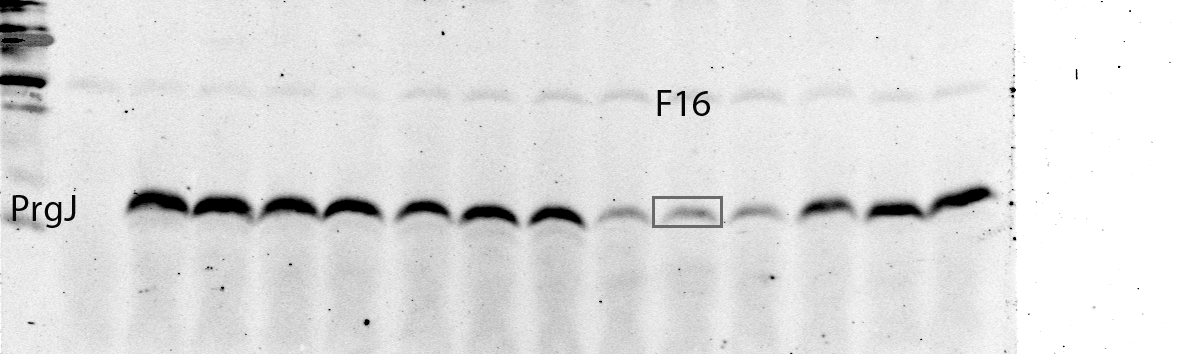

Supplement: S13 Data — (ZIP) [file pbio.3000351.s033.zip › S13-data/F16-anti-prgJ.tif]

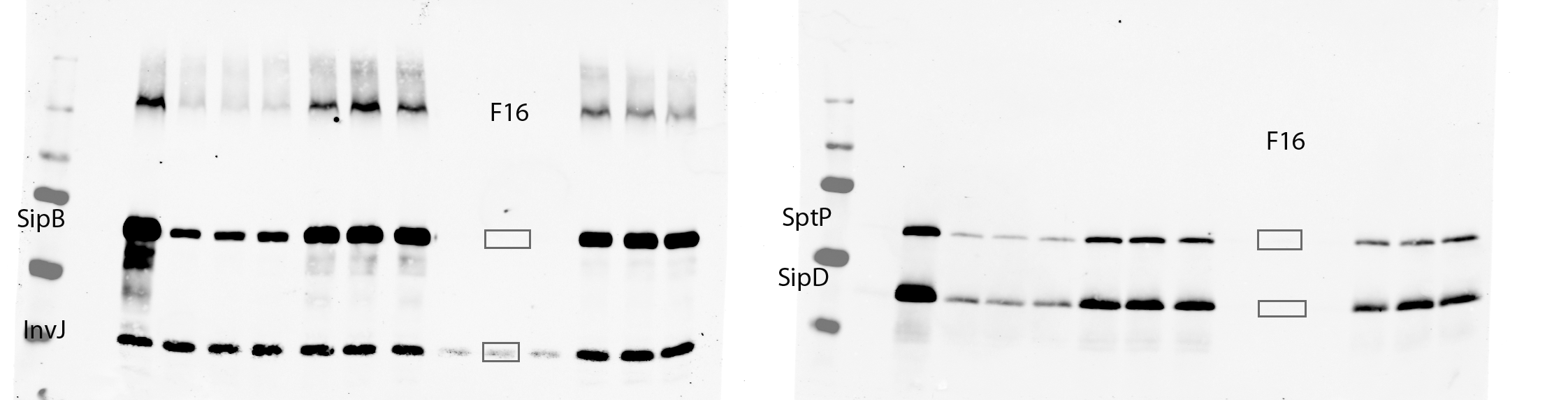

Supplement: S13 Data — (ZIP) [file pbio.3000351.s033.zip › S13-data/F16-anti-SipB-InvJ-SptP-SipD.tif]

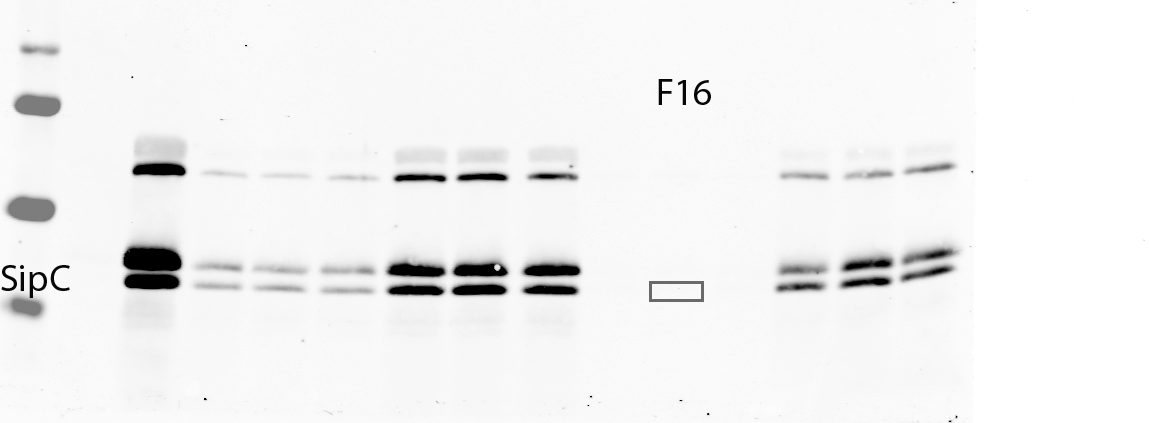

Supplement: S13 Data — (ZIP) [file pbio.3000351.s033.zip › S13-data/F16-anti-SipC.tif]

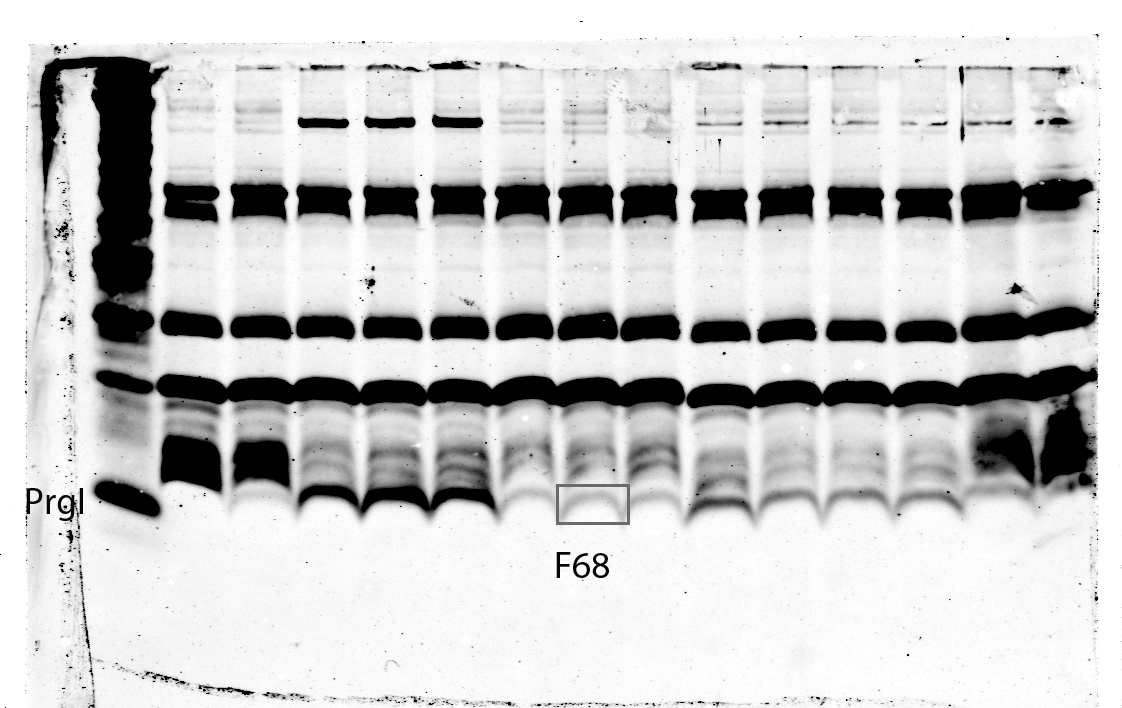

Supplement: S13 Data — (ZIP) [file pbio.3000351.s033.zip › S13-data/F68-anti-prgI.tif]

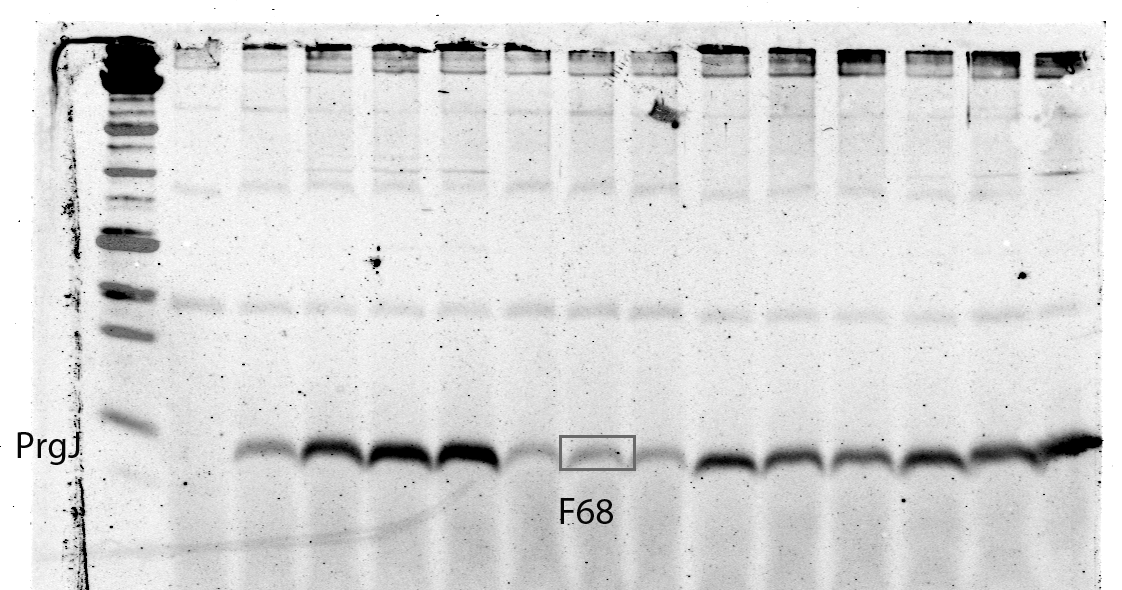

Supplement: S13 Data — (ZIP) [file pbio.3000351.s033.zip › S13-data/F68-anti-PrgJ.tif]

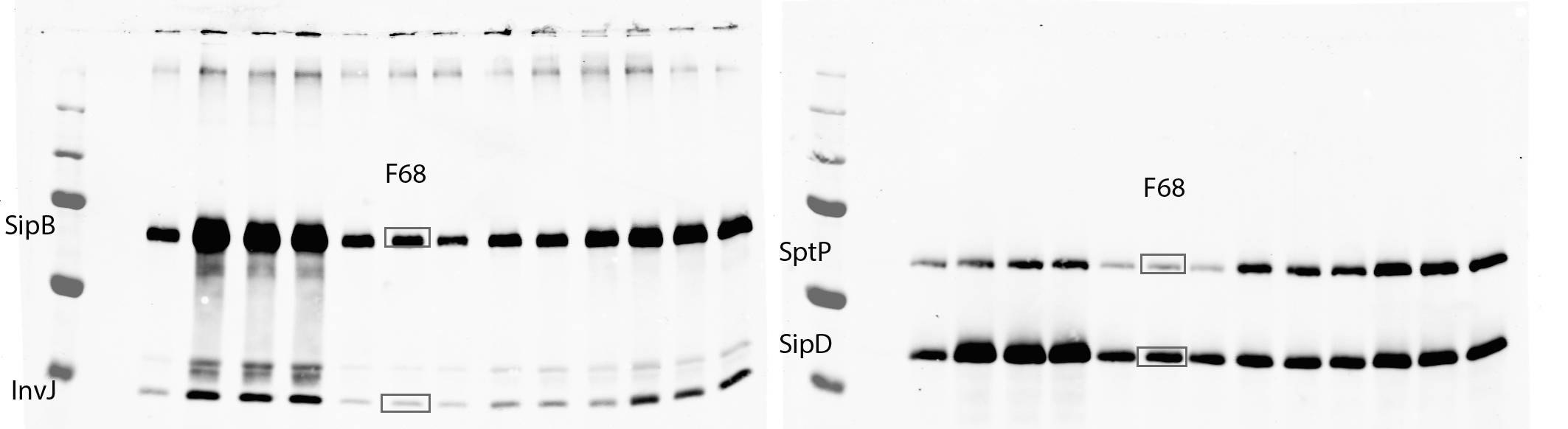

Supplement: S13 Data — (ZIP) [file pbio.3000351.s033.zip › S13-data/F68-anti-SipB-InvJ-SptP-SipD.tif]

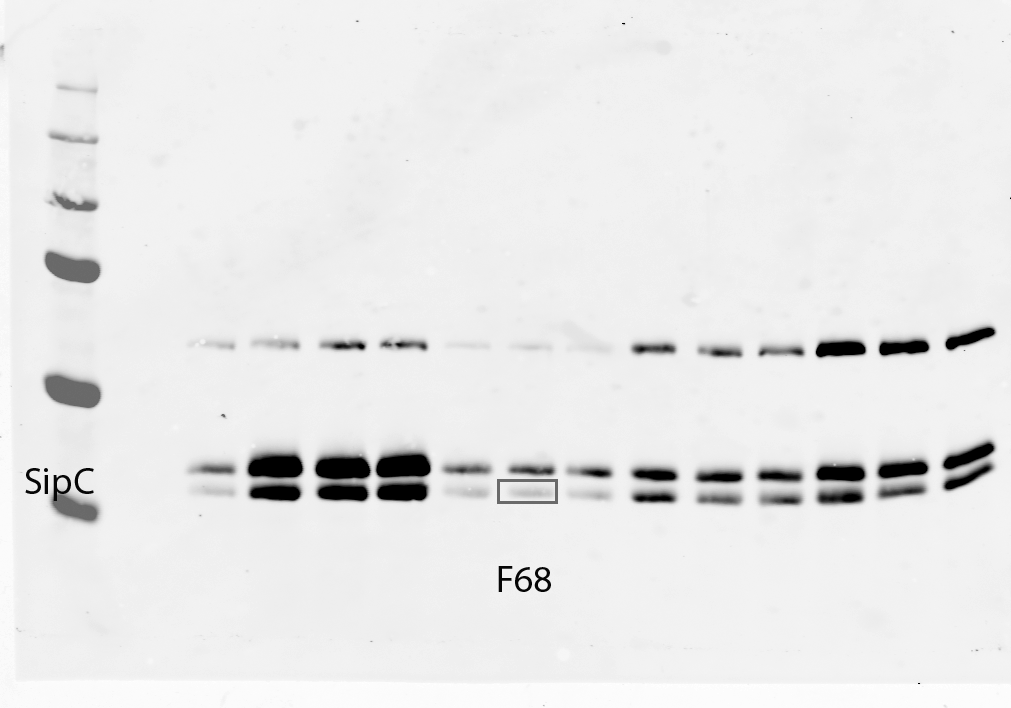

Supplement: S13 Data — (ZIP) [file pbio.3000351.s033.zip › S13-data/F68-anti-SipC.tif]

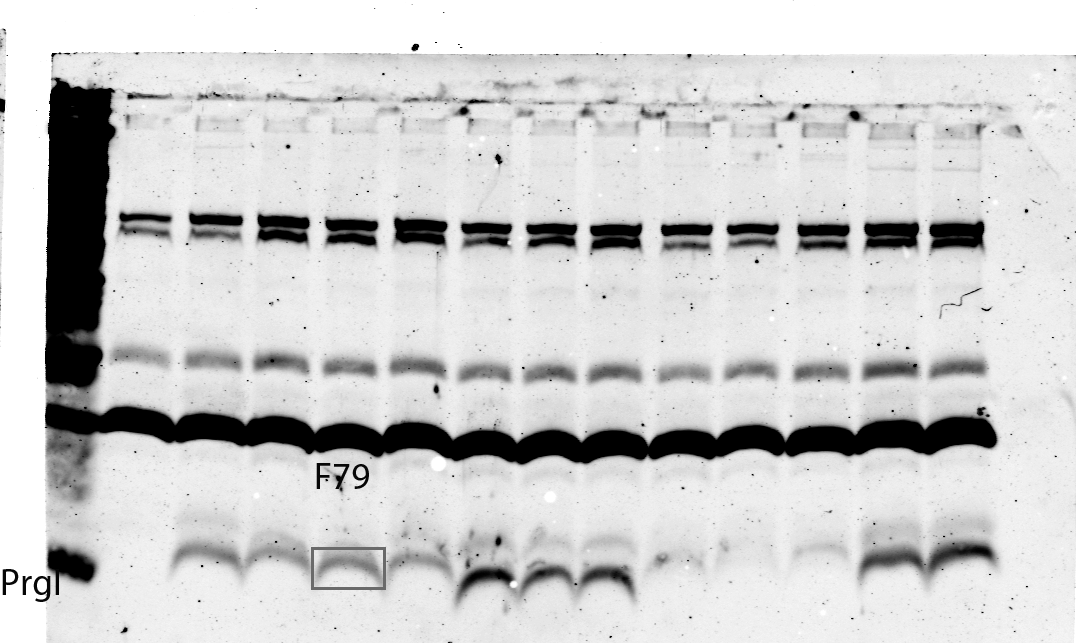

Supplement: S13 Data — (ZIP) [file pbio.3000351.s033.zip › S13-data/F79-anti-prgI.tif]

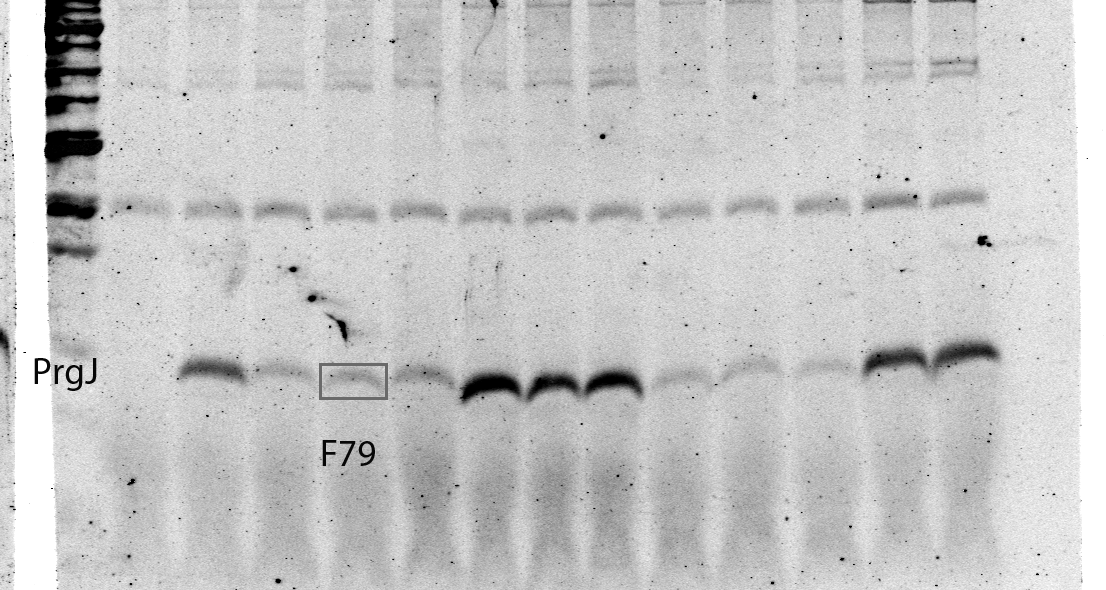

Supplement: S13 Data — (ZIP) [file pbio.3000351.s033.zip › S13-data/F79-anti-prgJ.tif]

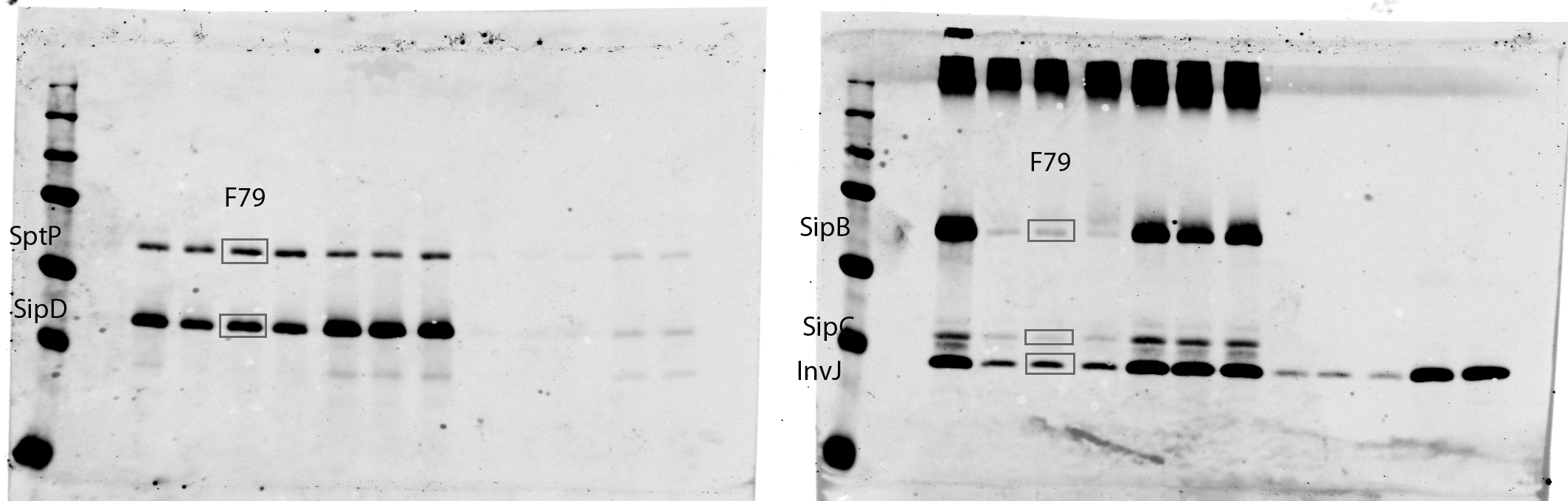

Supplement: S13 Data — (ZIP) [file pbio.3000351.s033.zip › S13-data/F79-anti-SptP-SipD-SipB-SipC-InvJ.tif]

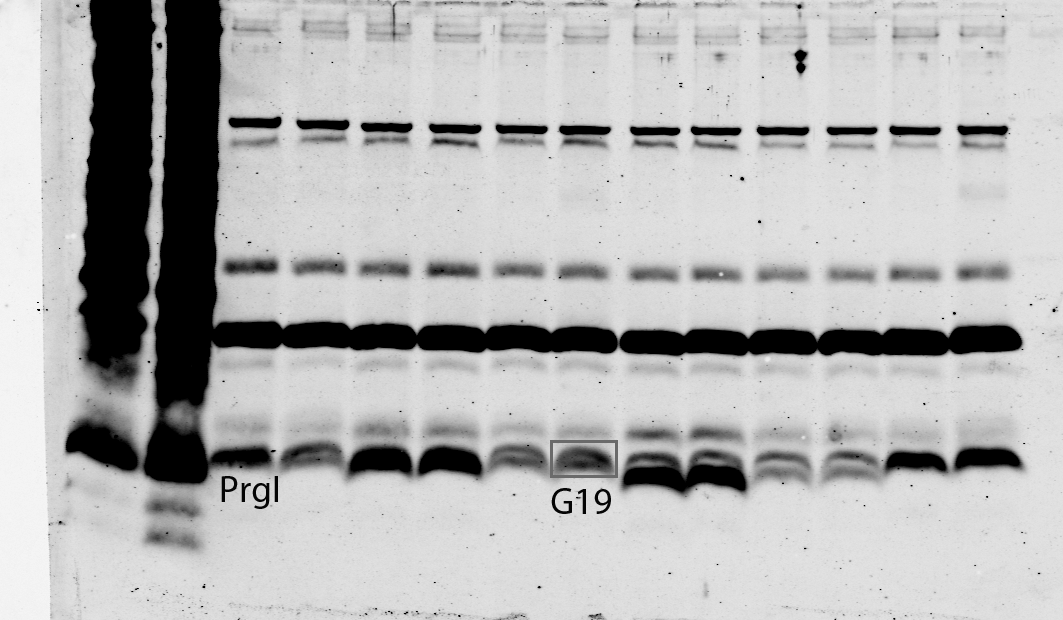

Supplement: S13 Data — (ZIP) [file pbio.3000351.s033.zip › S13-data/G19-anti-PrgI.tif]

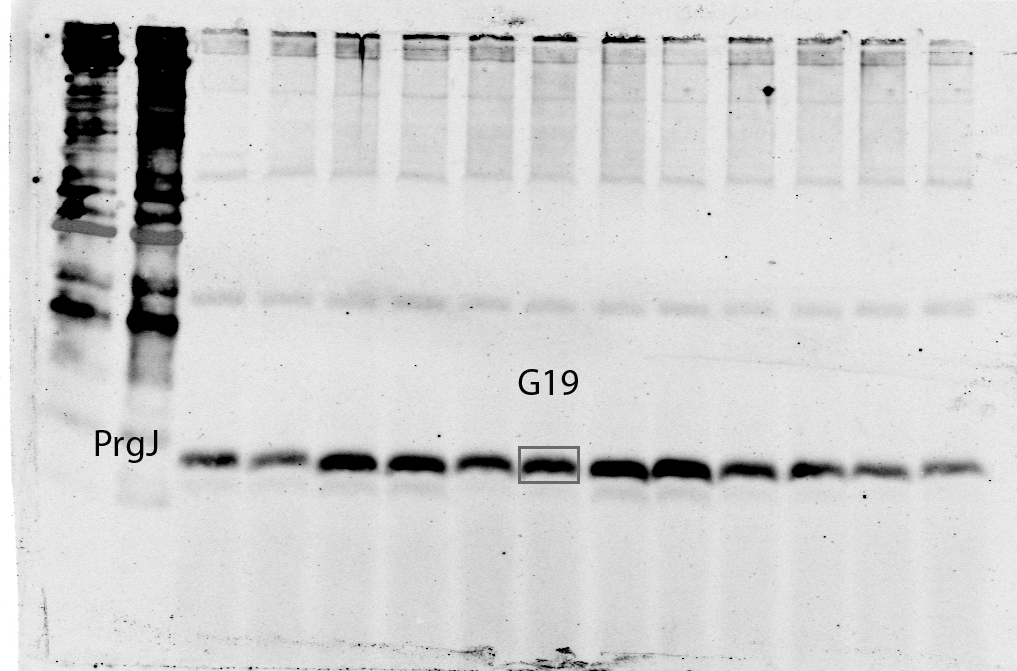

Supplement: S13 Data — (ZIP) [file pbio.3000351.s033.zip › S13-data/G19-anti-PrgJ.tif]

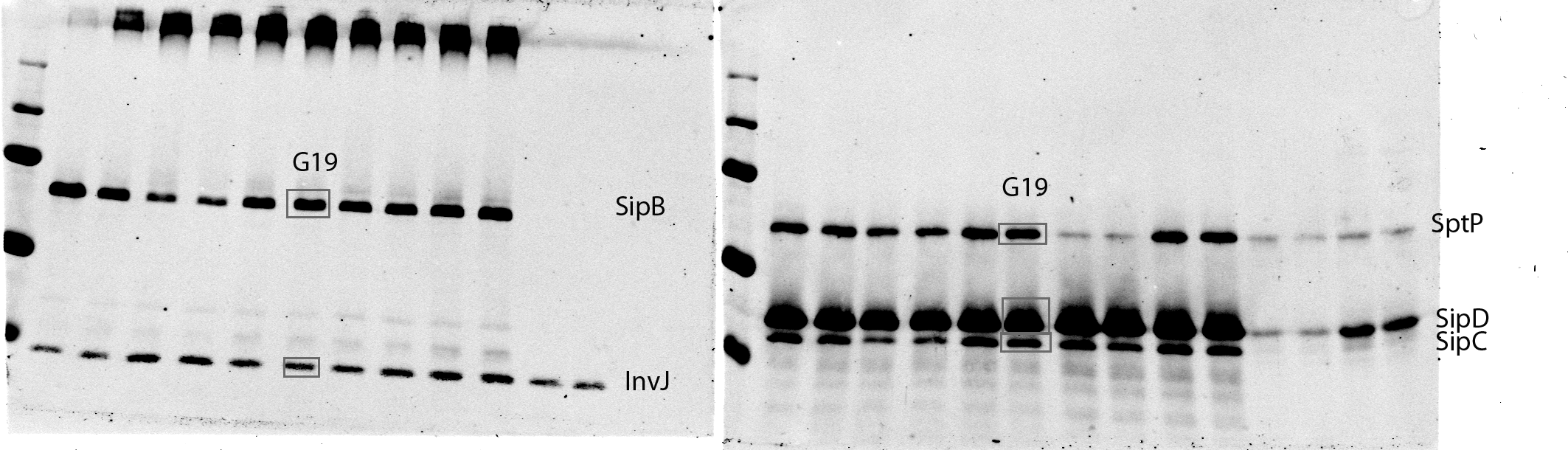

Supplement: S13 Data — (ZIP) [file pbio.3000351.s033.zip › S13-data/G19-anti-SipB-InvJ-SptP-SipD-SipC.tif]

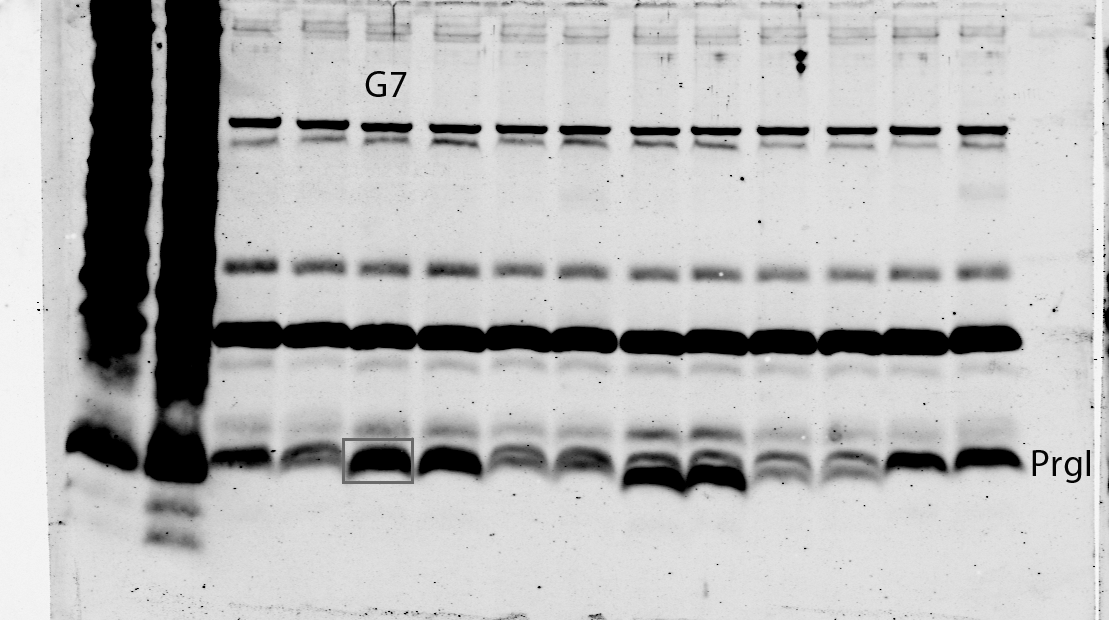

Supplement: S13 Data — (ZIP) [file pbio.3000351.s033.zip › S13-data/G7-anti-PrgI.tif]

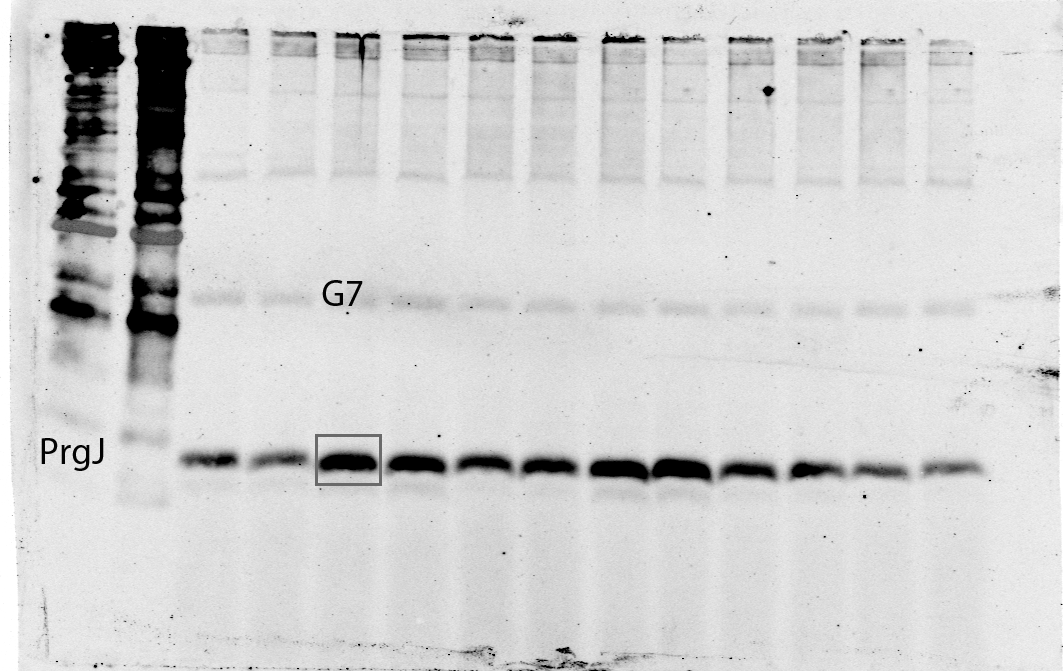

Supplement: S13 Data — (ZIP) [file pbio.3000351.s033.zip › S13-data/G7-anti-PrgJ.tif]

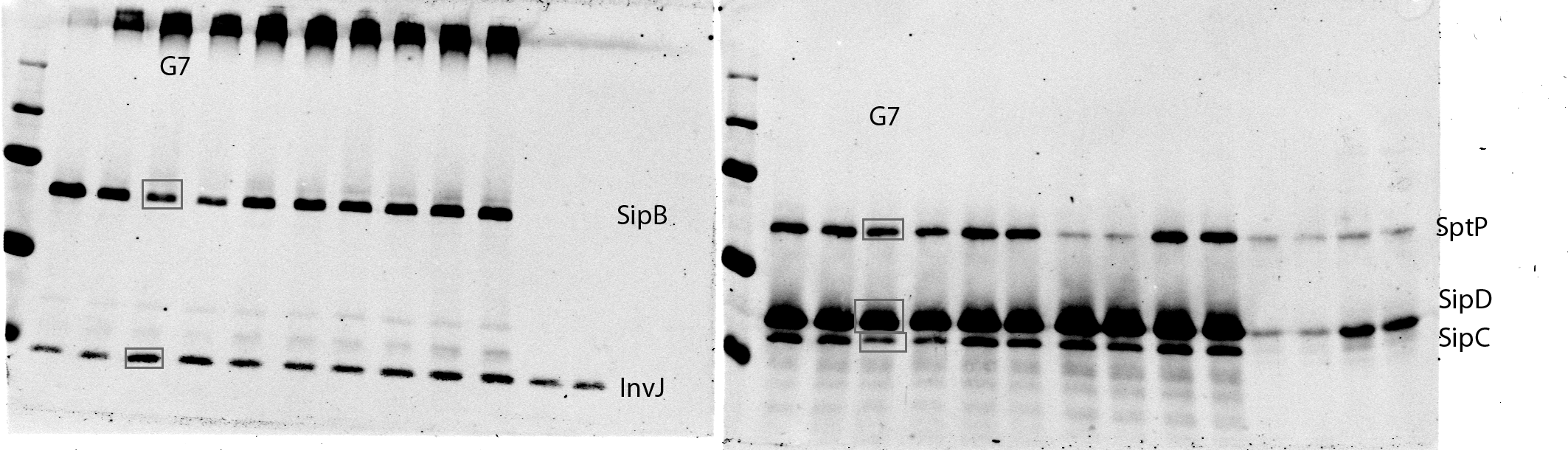

Supplement: S13 Data — (ZIP) [file pbio.3000351.s033.zip › S13-data/G7-anti-SipB-InvJ-SptP-SipD-SipC.tif]

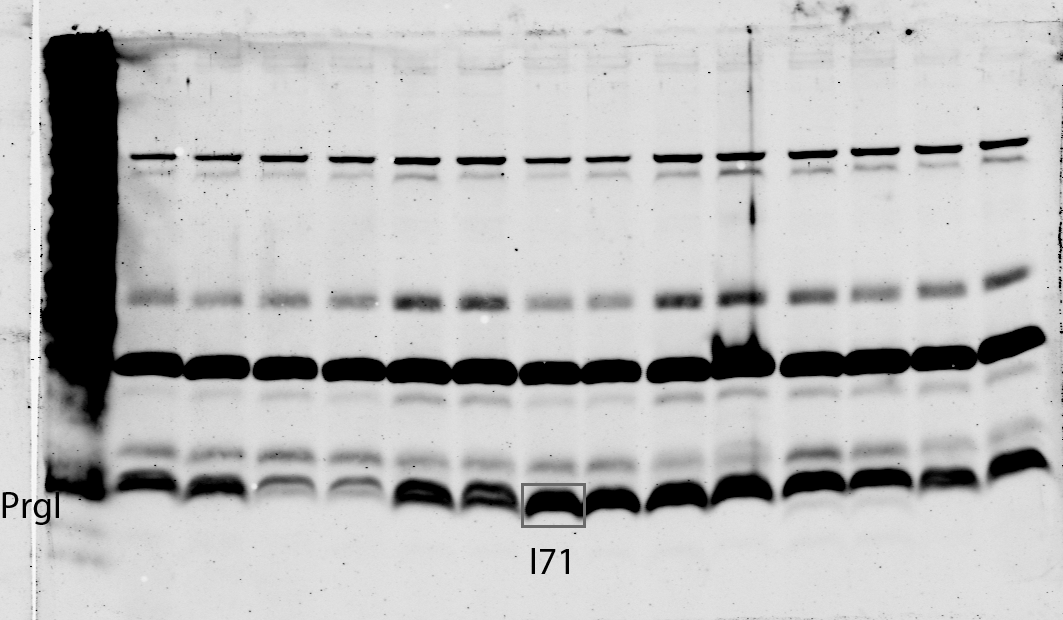

Supplement: S13 Data — (ZIP) [file pbio.3000351.s033.zip › S13-data/I71-anti-PrgI.tif]

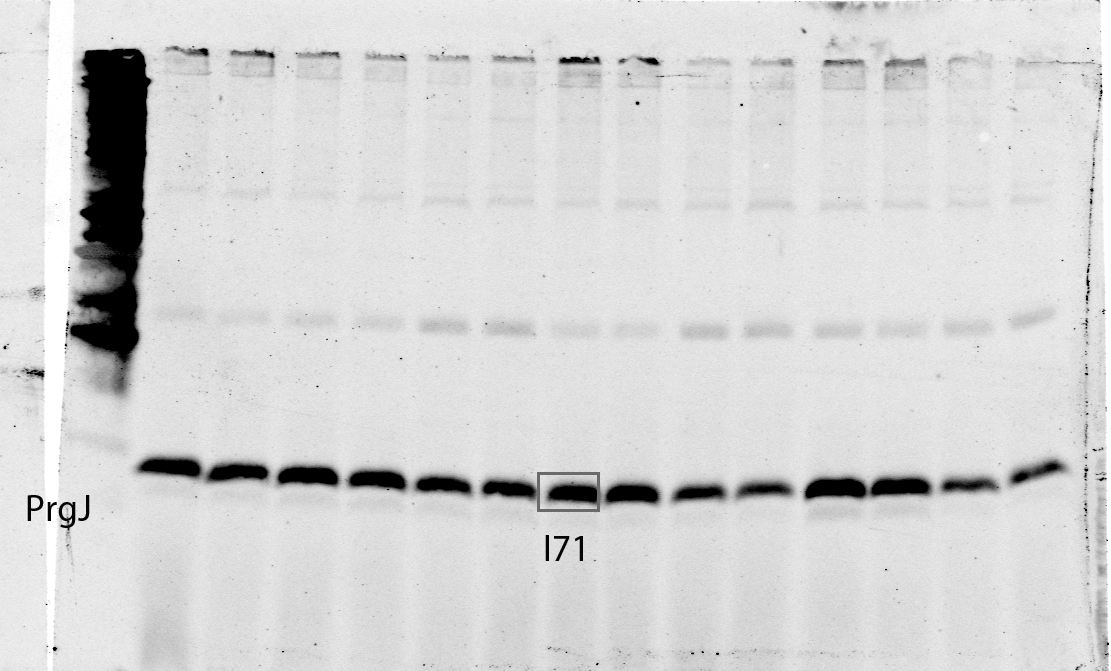

Supplement: S13 Data — (ZIP) [file pbio.3000351.s033.zip › S13-data/I71-anti-PrgJ.tif]

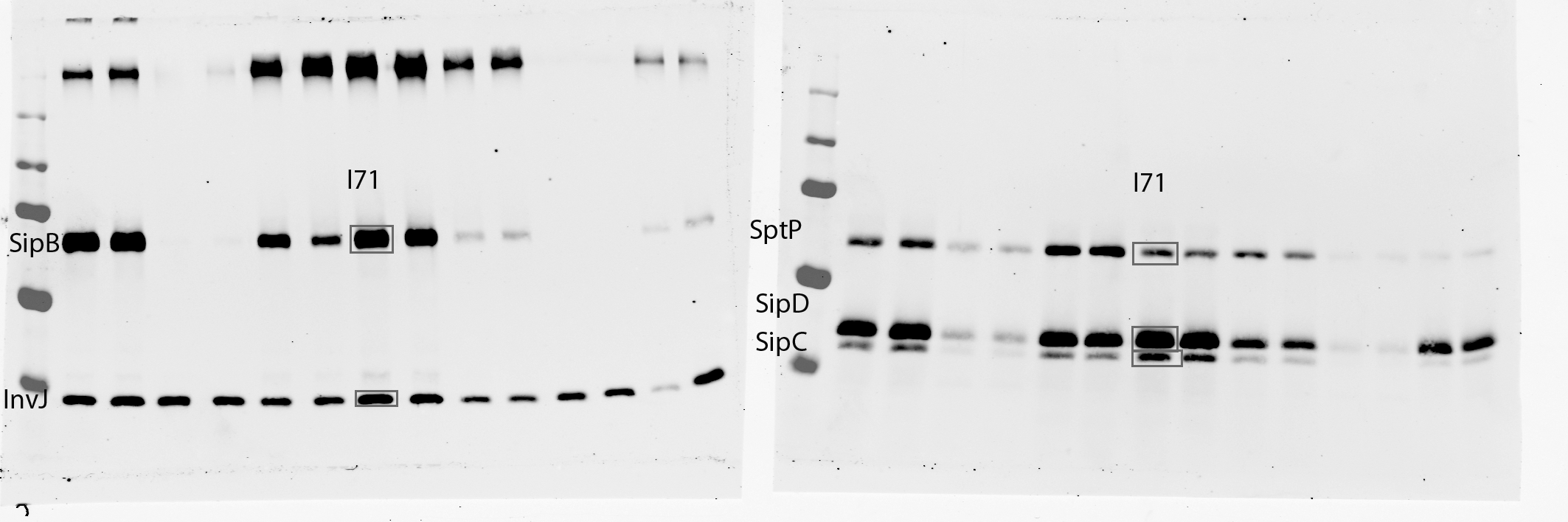

Supplement: S13 Data — (ZIP) [file pbio.3000351.s033.zip › S13-data/I71-anti-SipB-InvJ-SptP-SipD-SipC.tif]

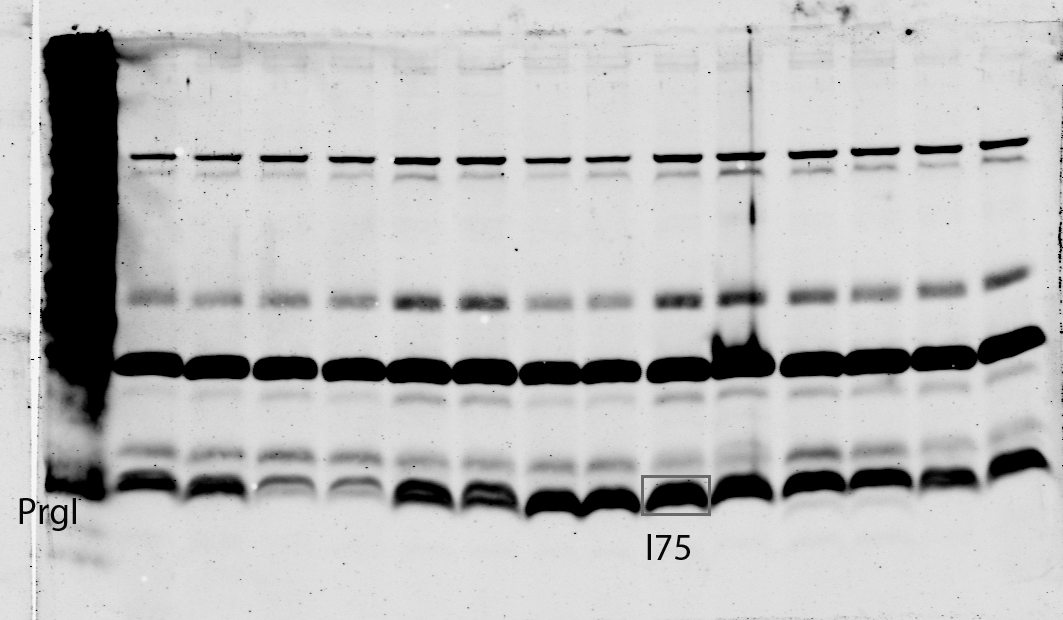

Supplement: S13 Data — (ZIP) [file pbio.3000351.s033.zip › S13-data/I75-anti-PrgI.tif]

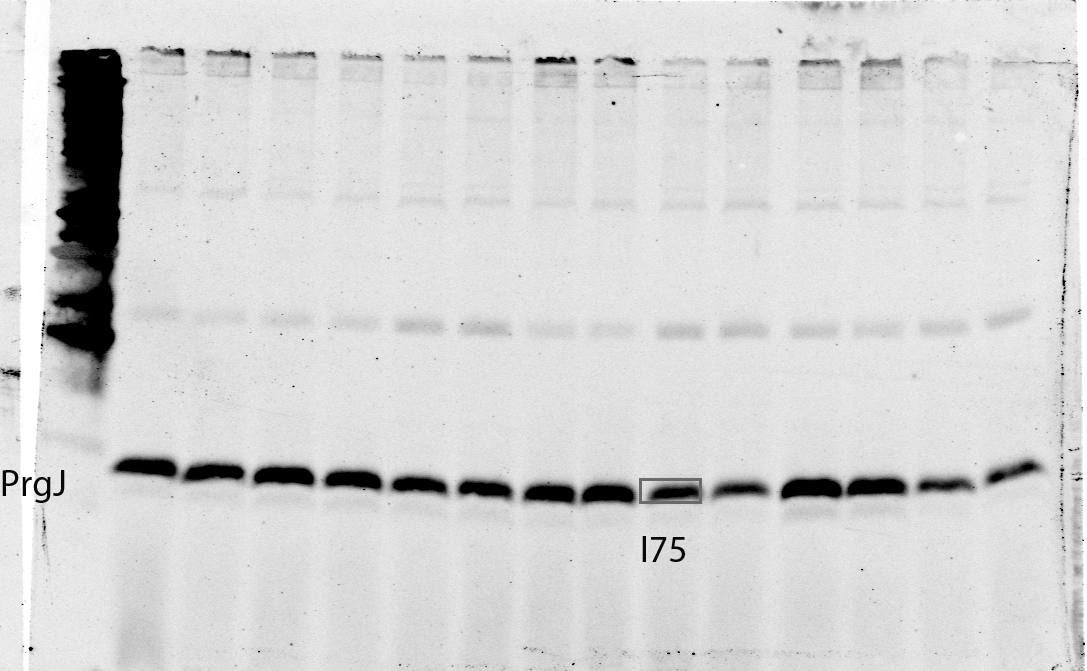

Supplement: S13 Data — (ZIP) [file pbio.3000351.s033.zip › S13-data/I75-anti-PrgJ.tif]

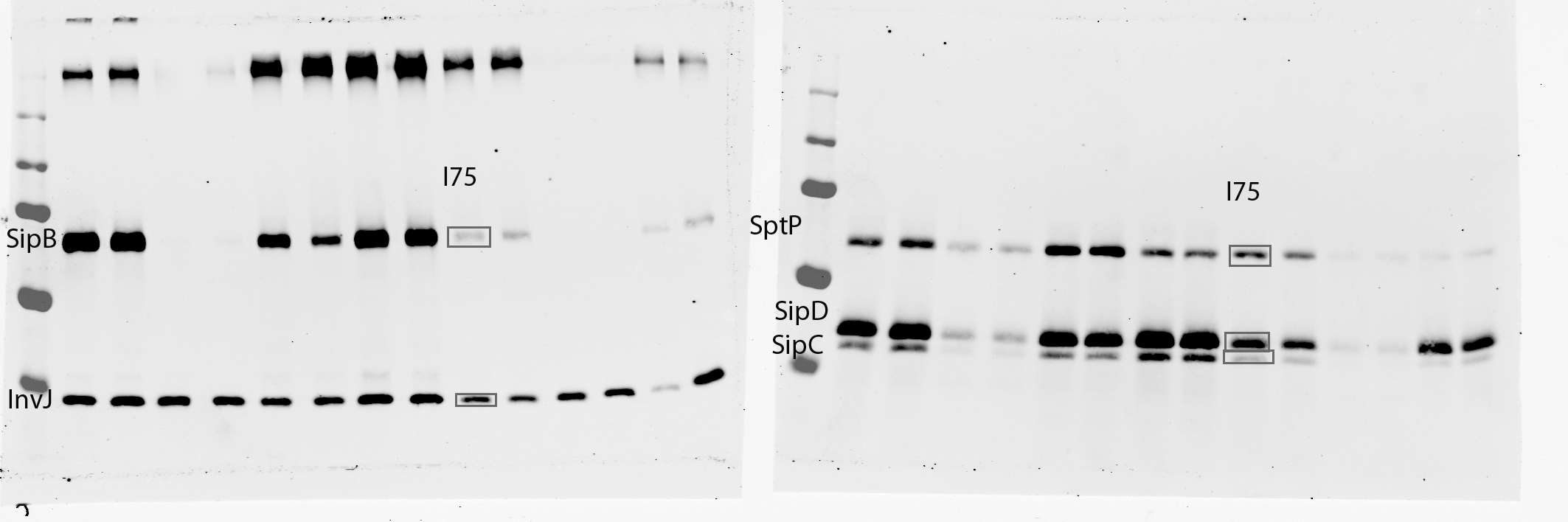

Supplement: S13 Data — (ZIP) [file pbio.3000351.s033.zip › S13-data/I75-anti-SipB-InvJ-SptP-SipD-SipC.tif]

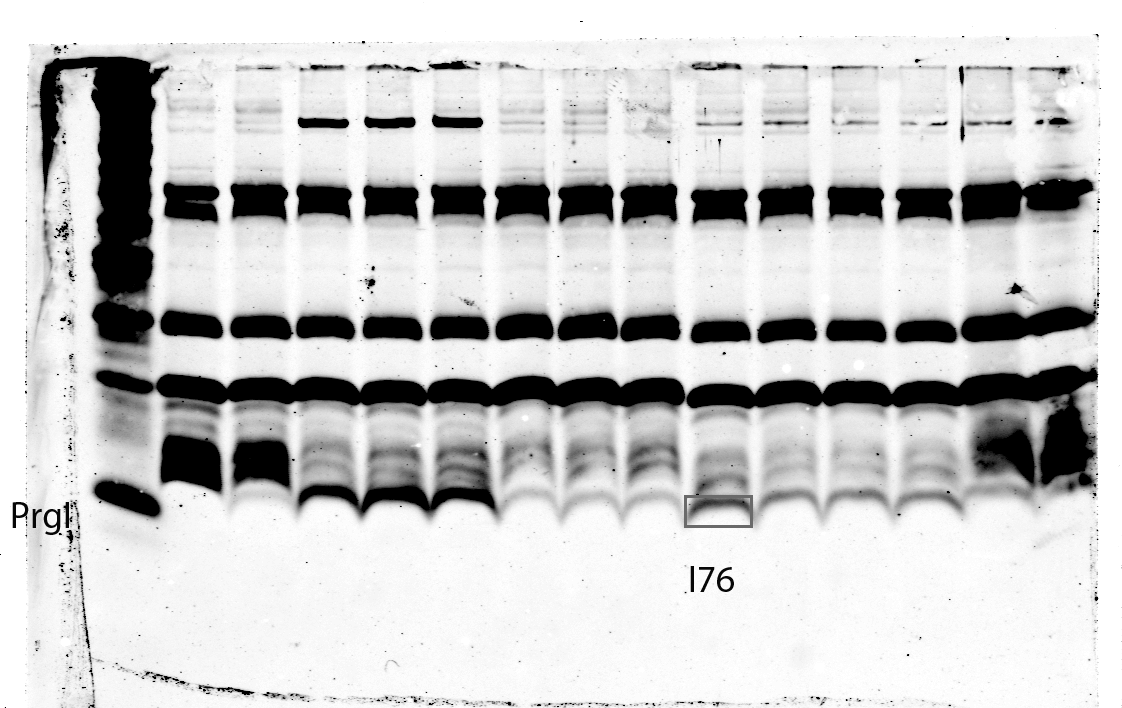

Supplement: S13 Data — (ZIP) [file pbio.3000351.s033.zip › S13-data/I76-anti-prgI.tif]

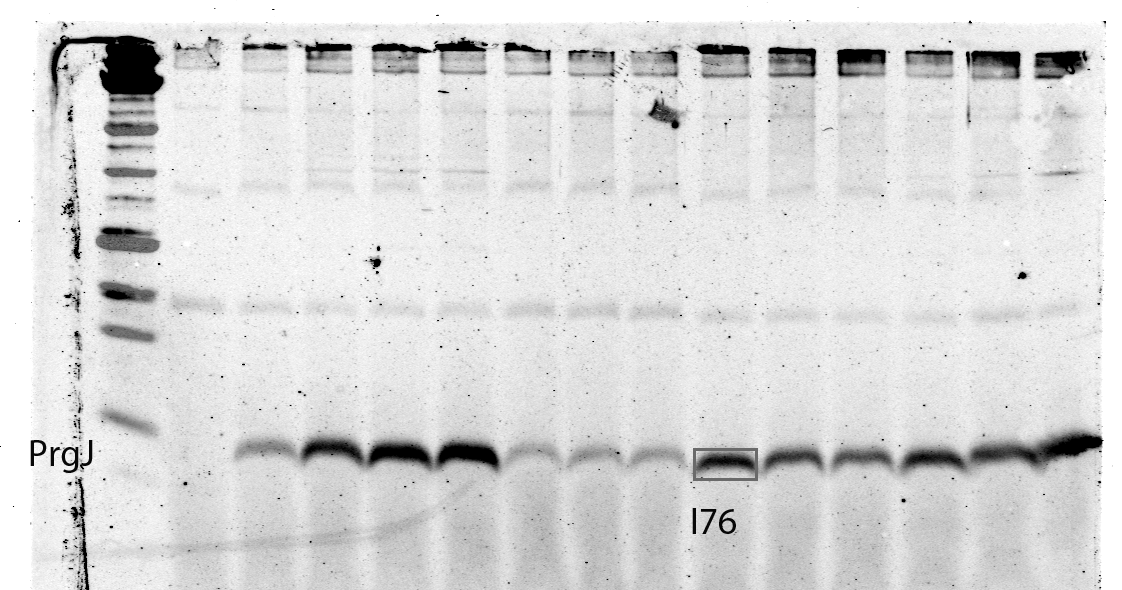

Supplement: S13 Data — (ZIP) [file pbio.3000351.s033.zip › S13-data/I76-anti-PrgJ.tif]

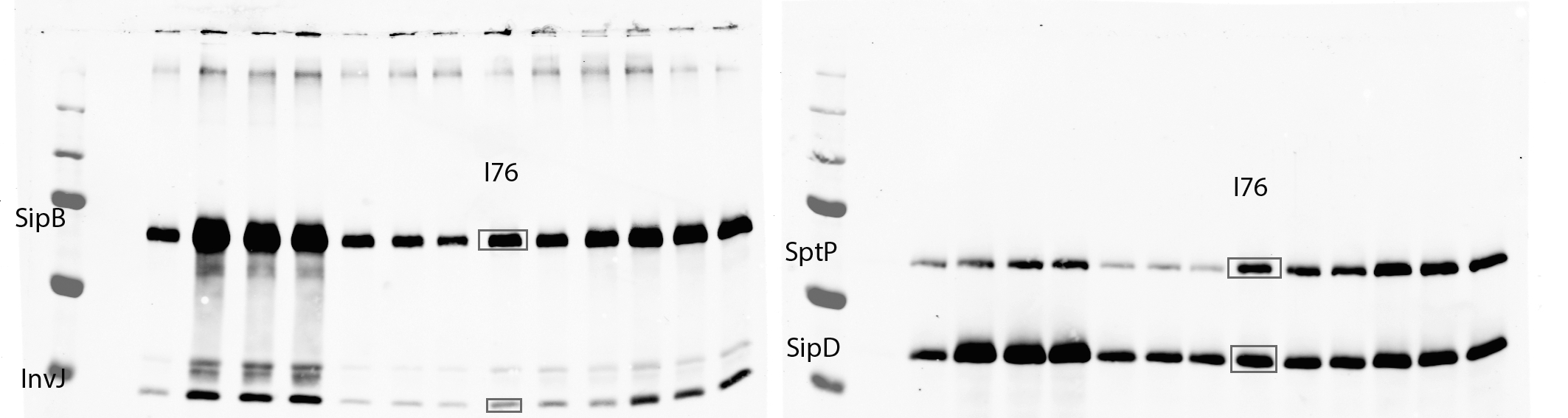

Supplement: S13 Data — (ZIP) [file pbio.3000351.s033.zip › S13-data/I76-anti-SipB-InvJ-SptP-SipD.tif]

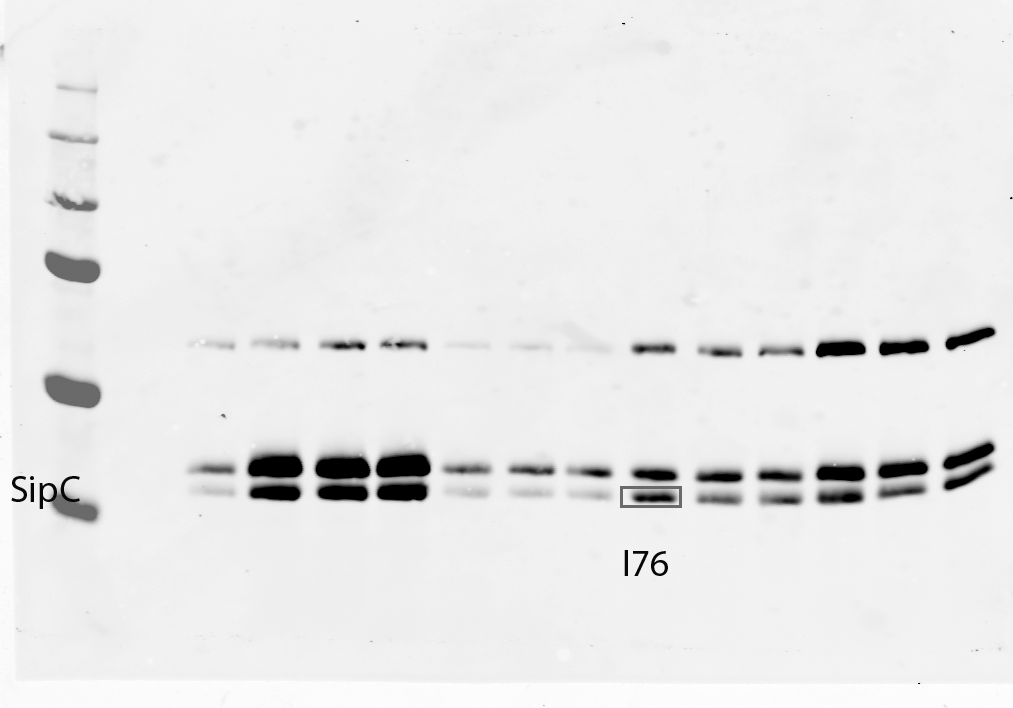

Supplement: S13 Data — (ZIP) [file pbio.3000351.s033.zip › S13-data/I76-anti-SipC.tif]

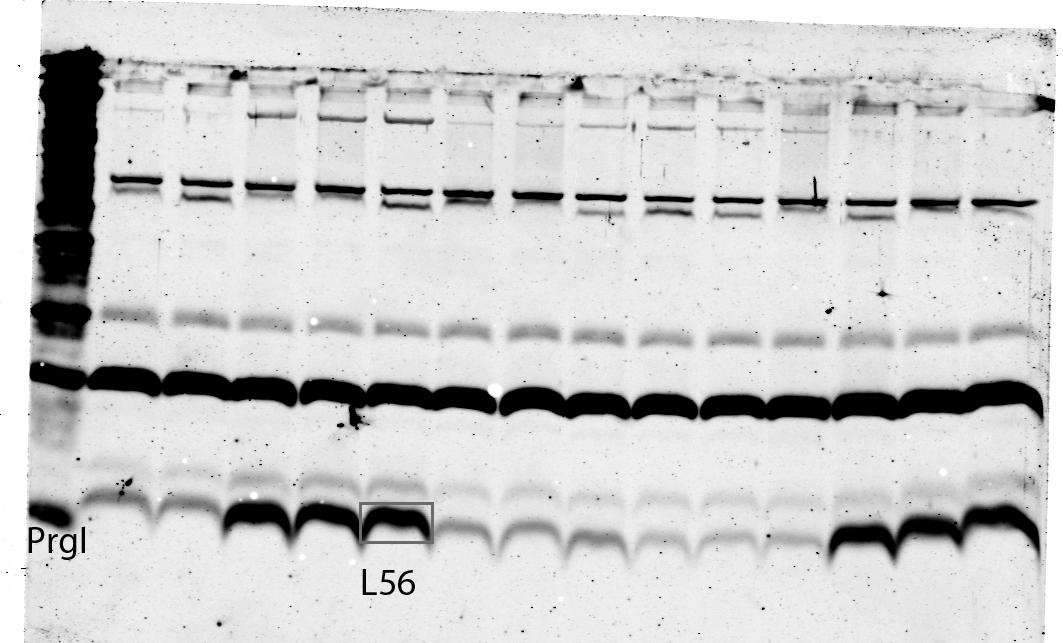

Supplement: S16 Data — (ZIP) [file pbio.3000351.s036.zip › S16-data/L56-anti-prgI.tif]

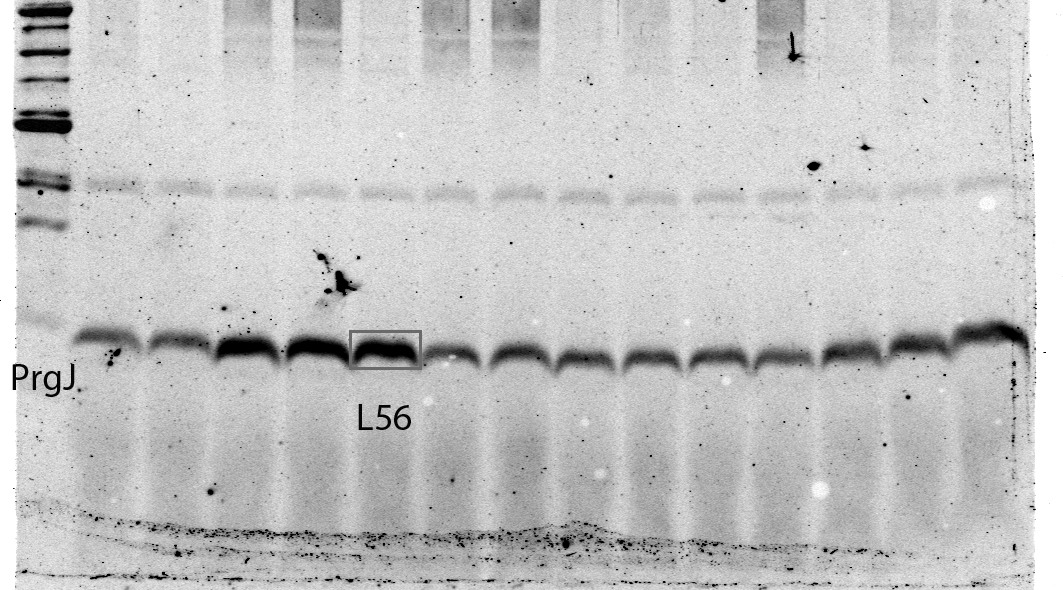

Supplement: S16 Data — (ZIP) [file pbio.3000351.s036.zip › S16-data/L56-anti-prgJ.tif]

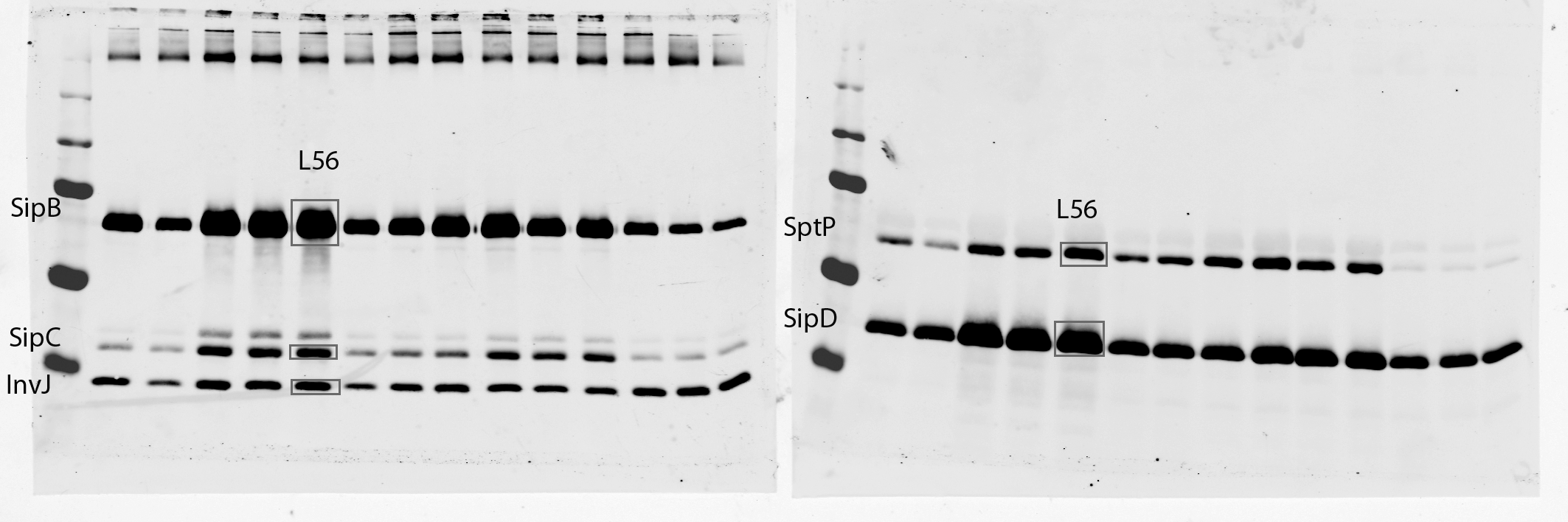

Supplement: S16 Data — (ZIP) [file pbio.3000351.s036.zip › S16-data/L56-anti-SipB-SipC-InvJ-SptP-SipD.tif]

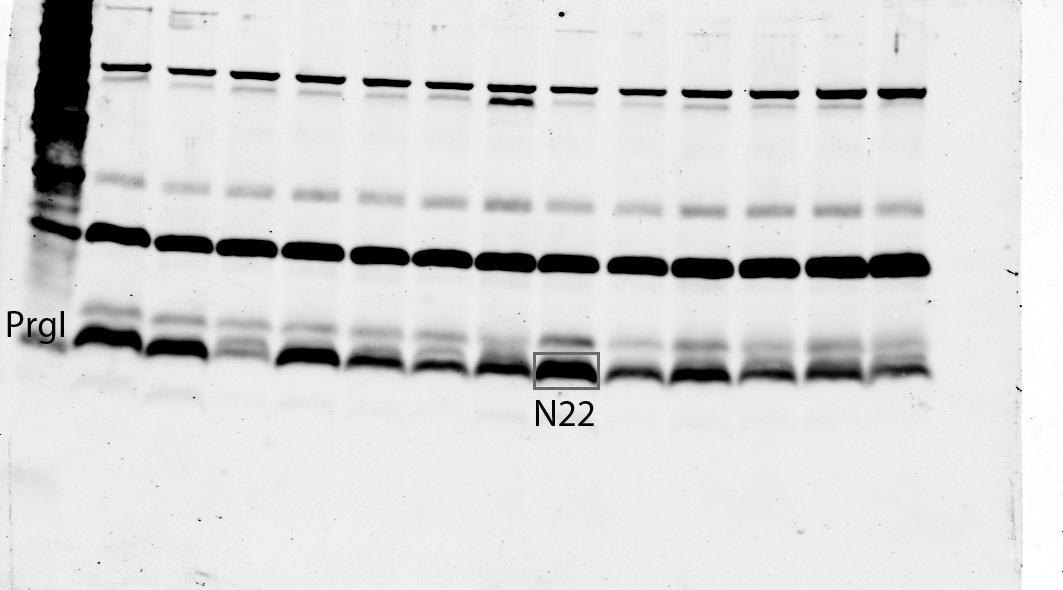

Supplement: S16 Data — (ZIP) [file pbio.3000351.s036.zip › S16-data/N22-anti-PrgI.tif]

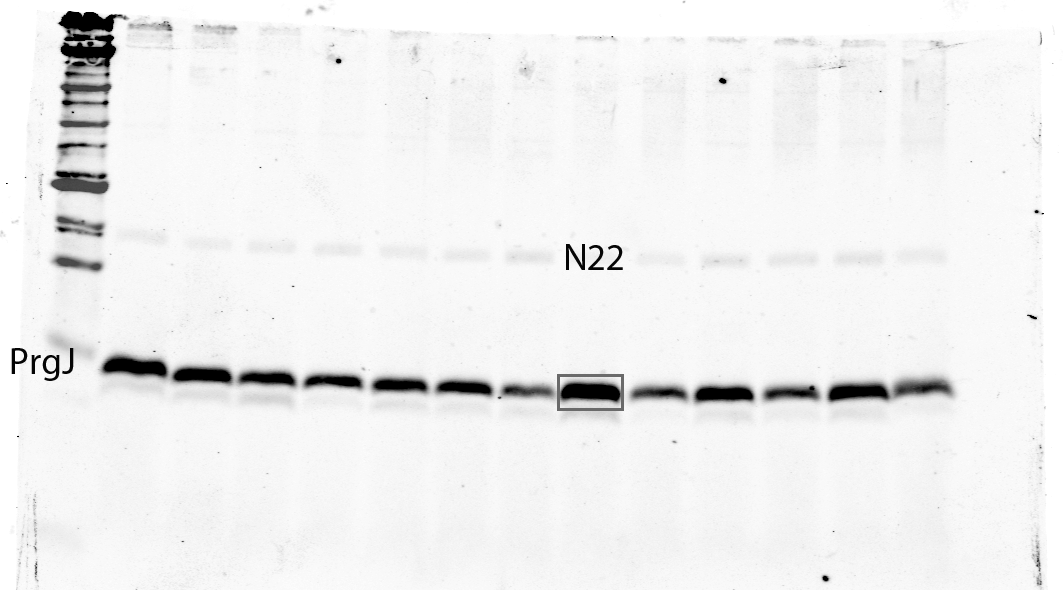

Supplement: S16 Data — (ZIP) [file pbio.3000351.s036.zip › S16-data/N22-anti-PrgJ.tif]

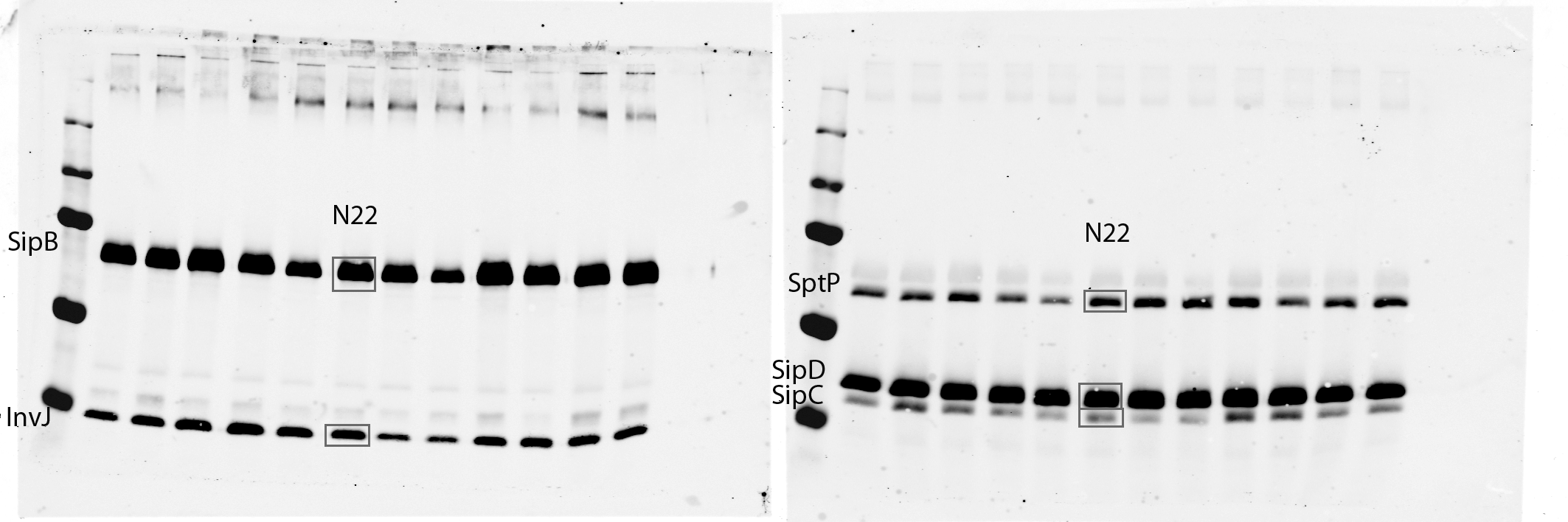

Supplement: S16 Data — (ZIP) [file pbio.3000351.s036.zip › S16-data/N22-anti-SipB-InvJ-SptP-SipD-SipC.tif]

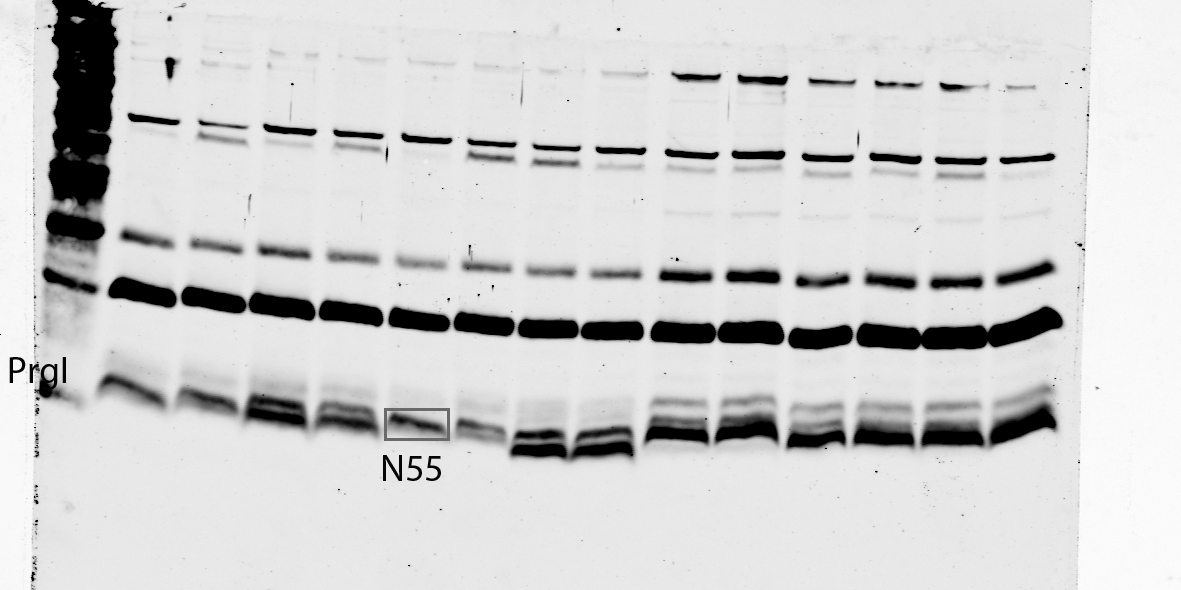

Supplement: S16 Data — (ZIP) [file pbio.3000351.s036.zip › S16-data/N55-anti-prgI.tif]

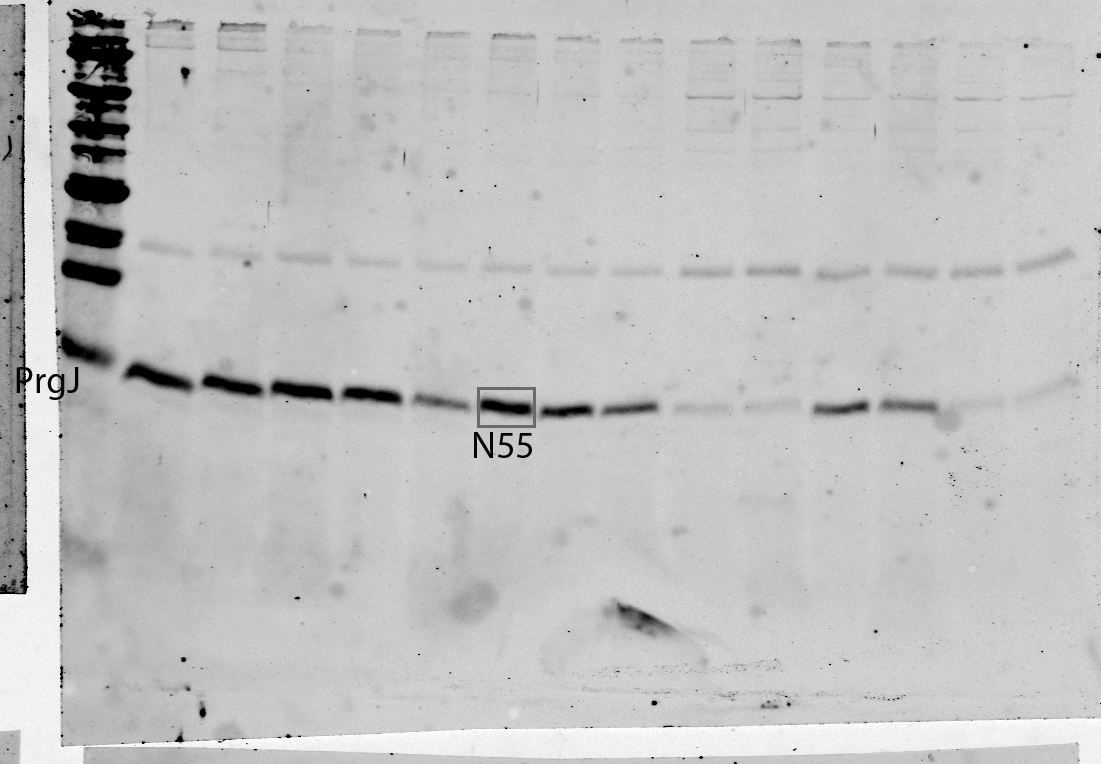

Supplement: S16 Data — (ZIP) [file pbio.3000351.s036.zip › S16-data/N55-anti-prgJ.tif]

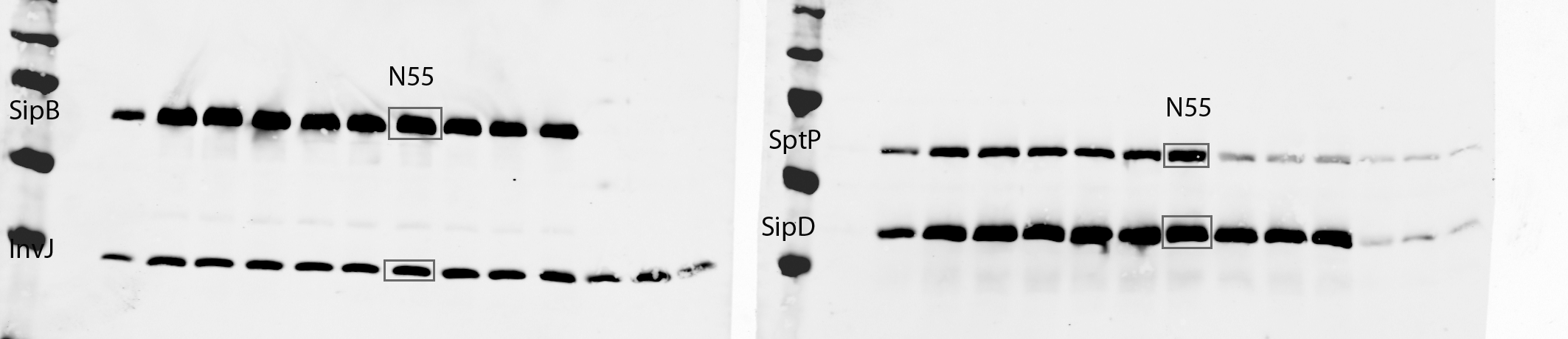

Supplement: S16 Data — (ZIP) [file pbio.3000351.s036.zip › S16-data/N55-anti-SipB-InvJ-SptP-SipD.tif]

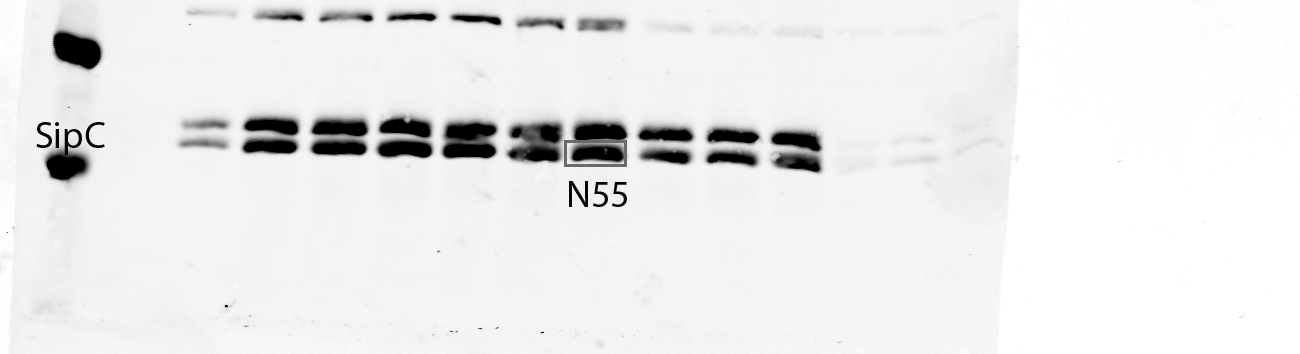

Supplement: S16 Data — (ZIP) [file pbio.3000351.s036.zip › S16-data/N55-anti-sipC.tif]

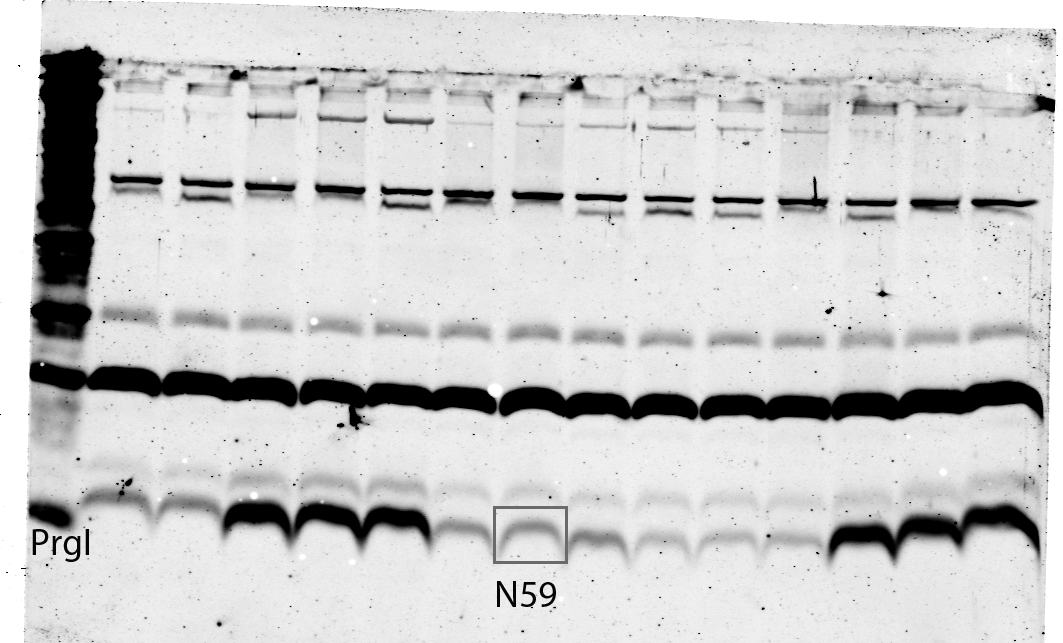

Supplement: S16 Data — (ZIP) [file pbio.3000351.s036.zip › S16-data/N59-anti-prgI.tif]

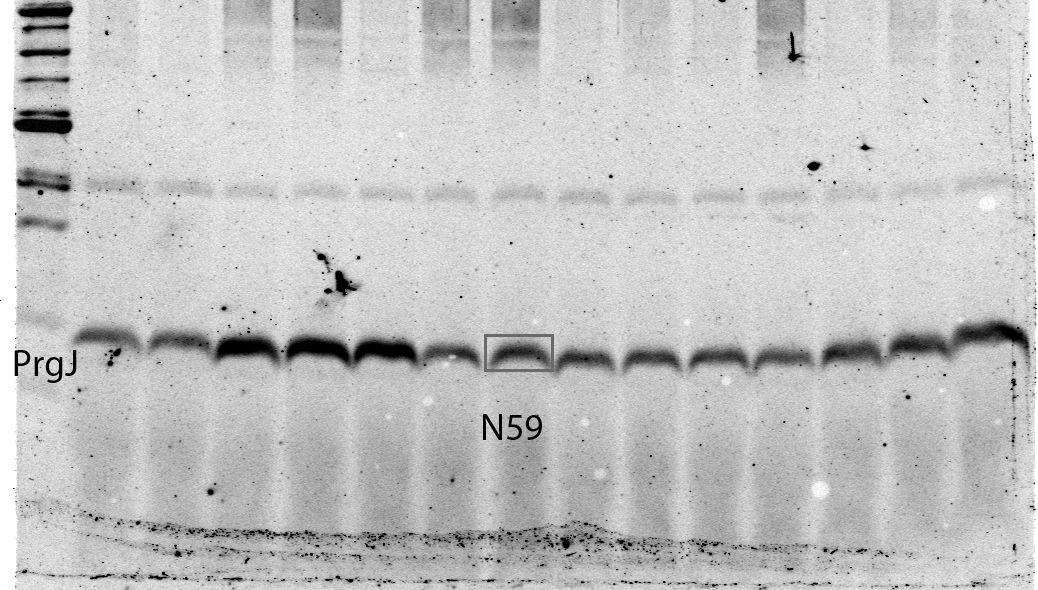

Supplement: S16 Data — (ZIP) [file pbio.3000351.s036.zip › S16-data/N59-anti-prgJ.tif]

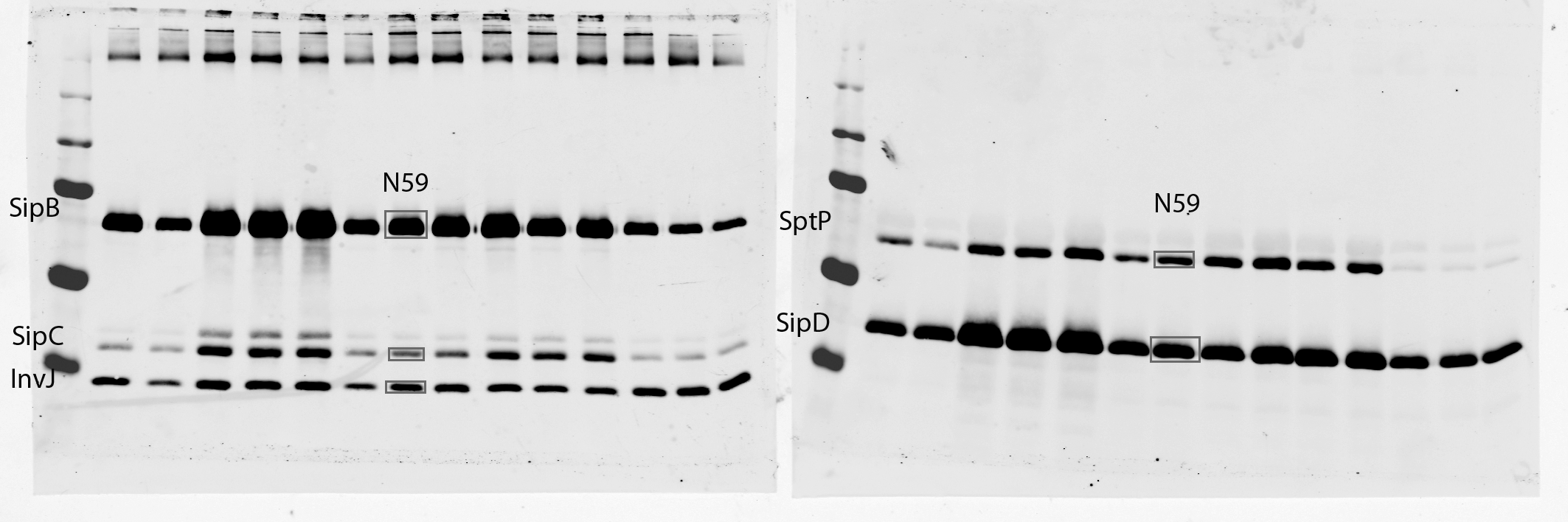

Supplement: S16 Data — (ZIP) [file pbio.3000351.s036.zip › S16-data/N59-anti-SipB-SipC-invJ-SptP-SipD.tif]

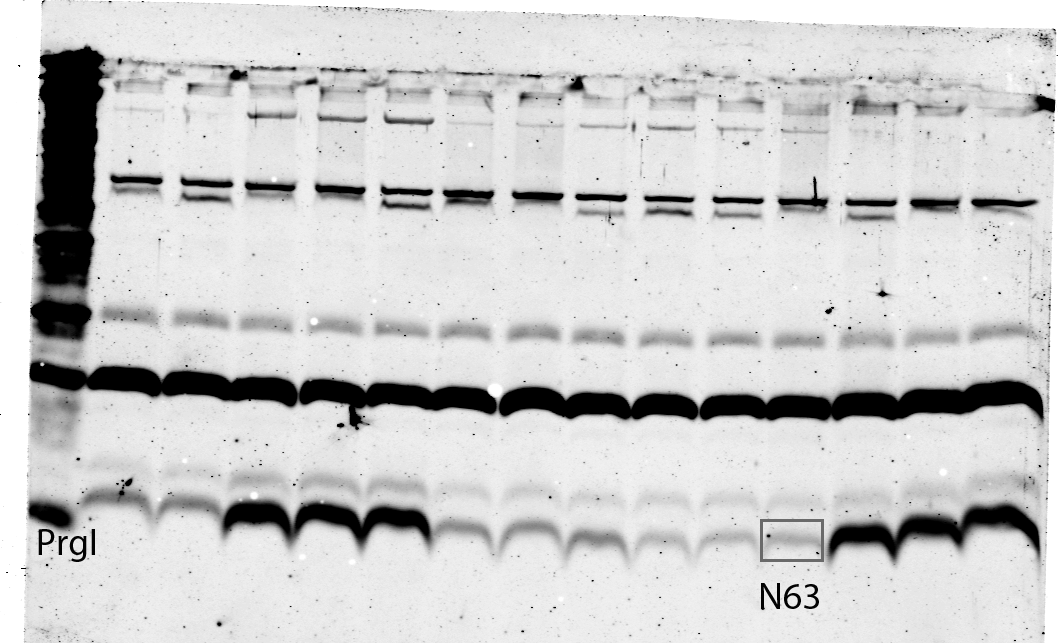

Supplement: S16 Data — (ZIP) [file pbio.3000351.s036.zip › S16-data/N63-anti-prgI.tif]

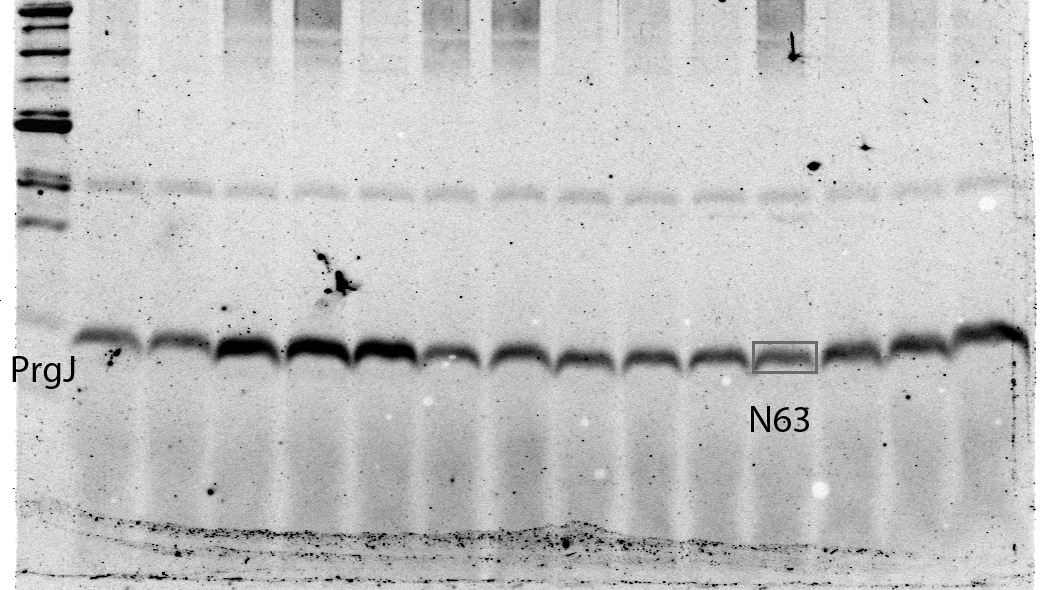

Supplement: S16 Data — (ZIP) [file pbio.3000351.s036.zip › S16-data/N63-anti-prgJ.tif]

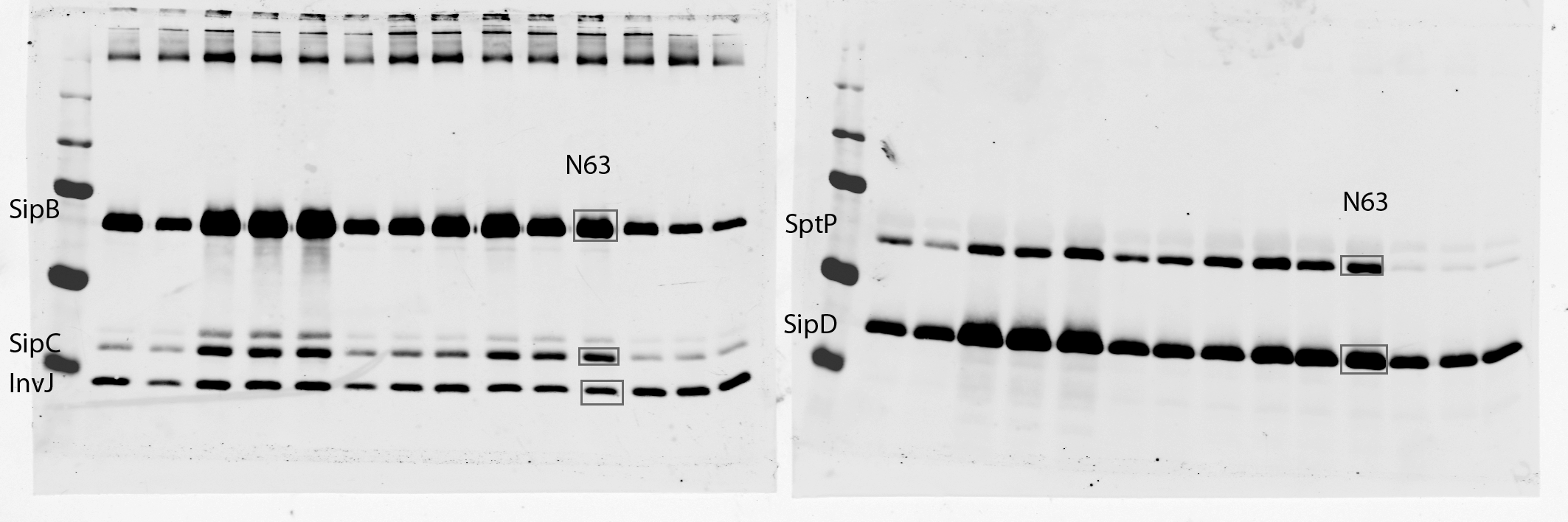

Supplement: S16 Data — (ZIP) [file pbio.3000351.s036.zip › S16-data/N63-anti-SipB-SipC-InvJ-SptP-SipD.tif]

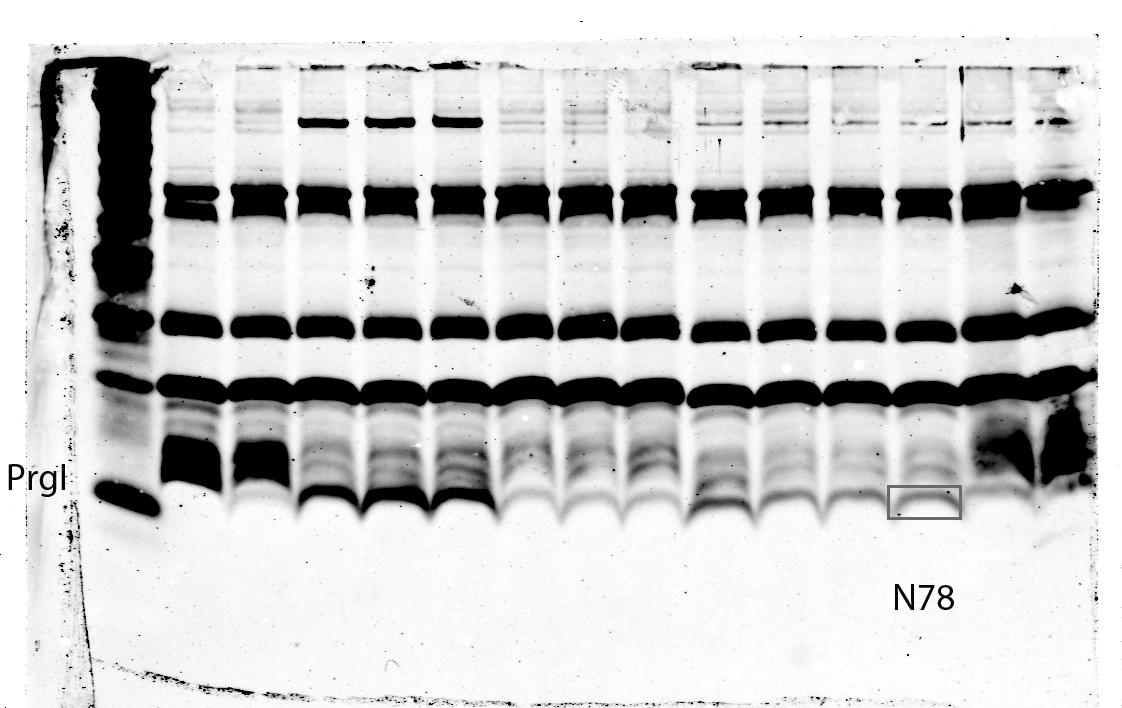

Supplement: S16 Data — (ZIP) [file pbio.3000351.s036.zip › S16-data/N78-anti-prgI.tif]

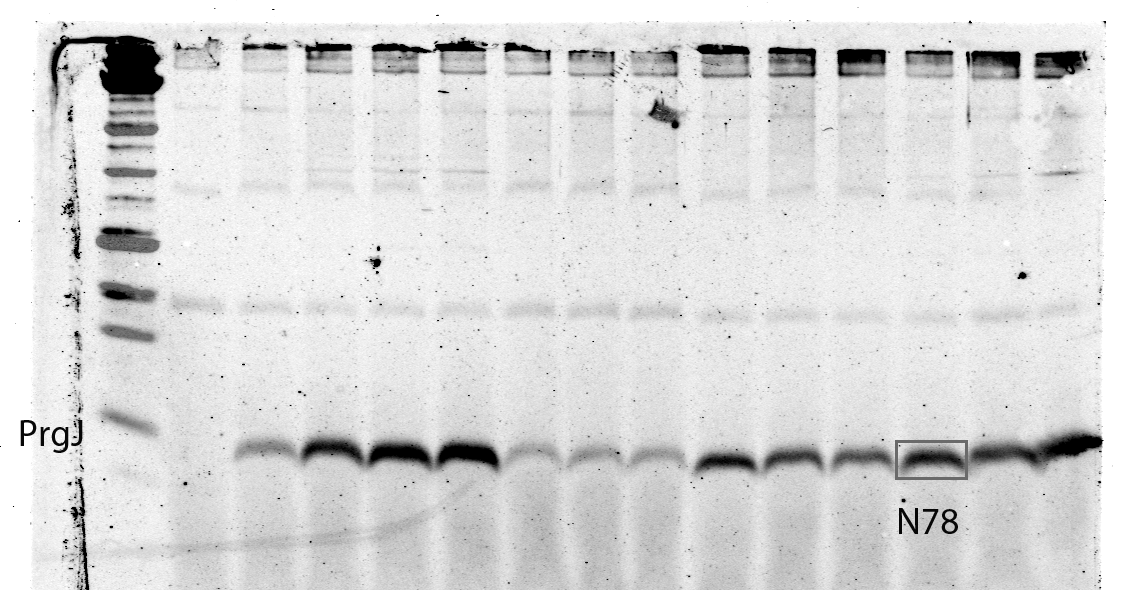

Supplement: S16 Data — (ZIP) [file pbio.3000351.s036.zip › S16-data/N78-anti-PrgJ.tif]

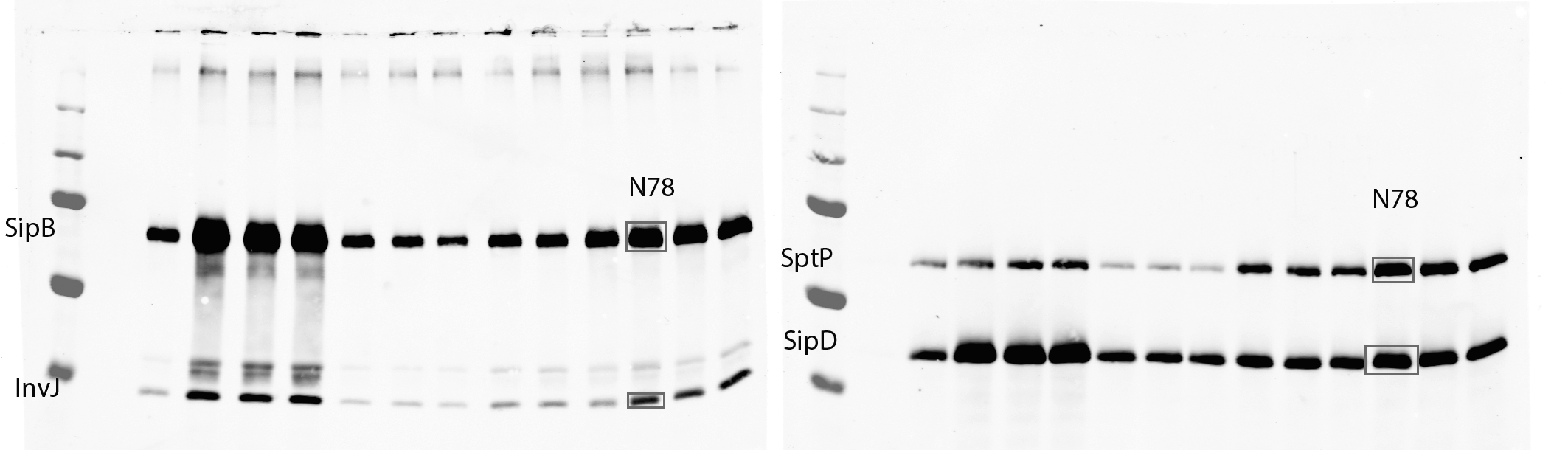

Supplement: S16 Data — (ZIP) [file pbio.3000351.s036.zip › S16-data/N78-anti-SipB-InvJ-SptP-SipD.tif]

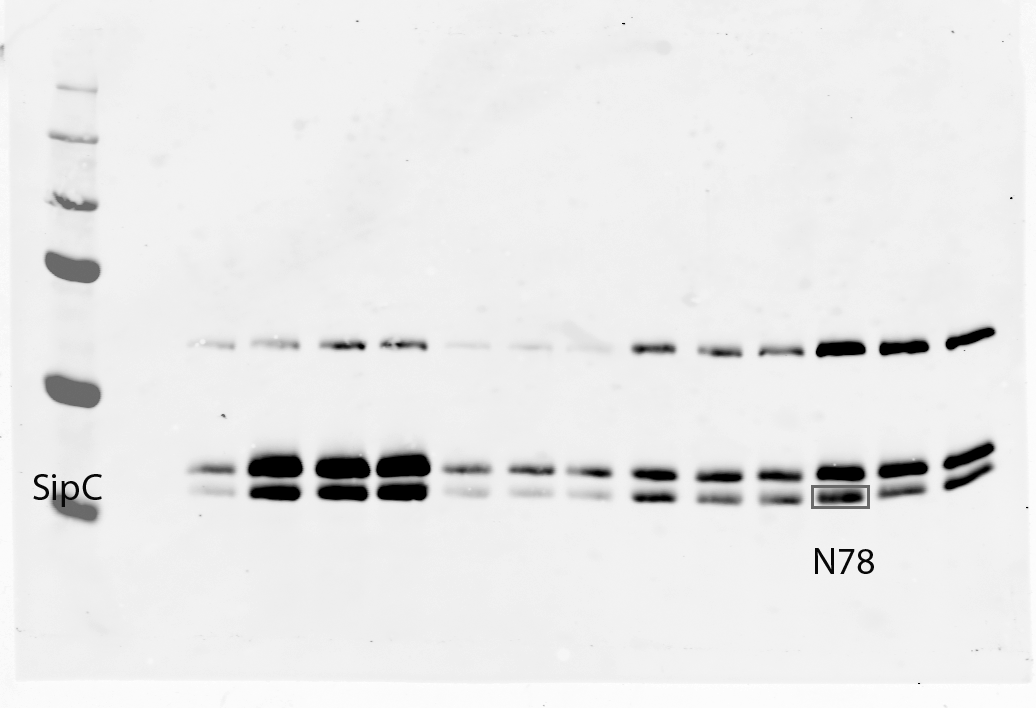

Supplement: S16 Data — (ZIP) [file pbio.3000351.s036.zip › S16-data/N78-anti-SipC.tif]

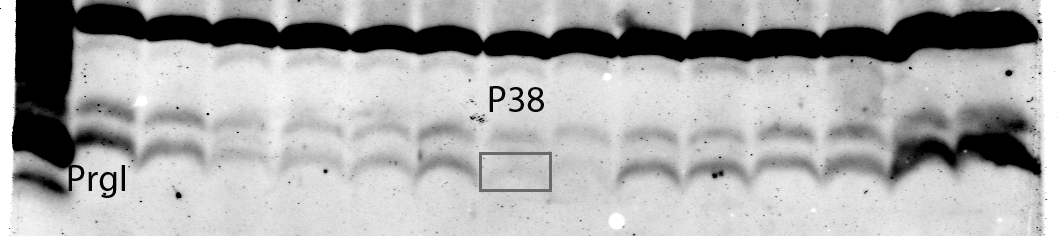

Supplement: S16 Data — (ZIP) [file pbio.3000351.s036.zip › S16-data/P38-anti-PrgI.tif]

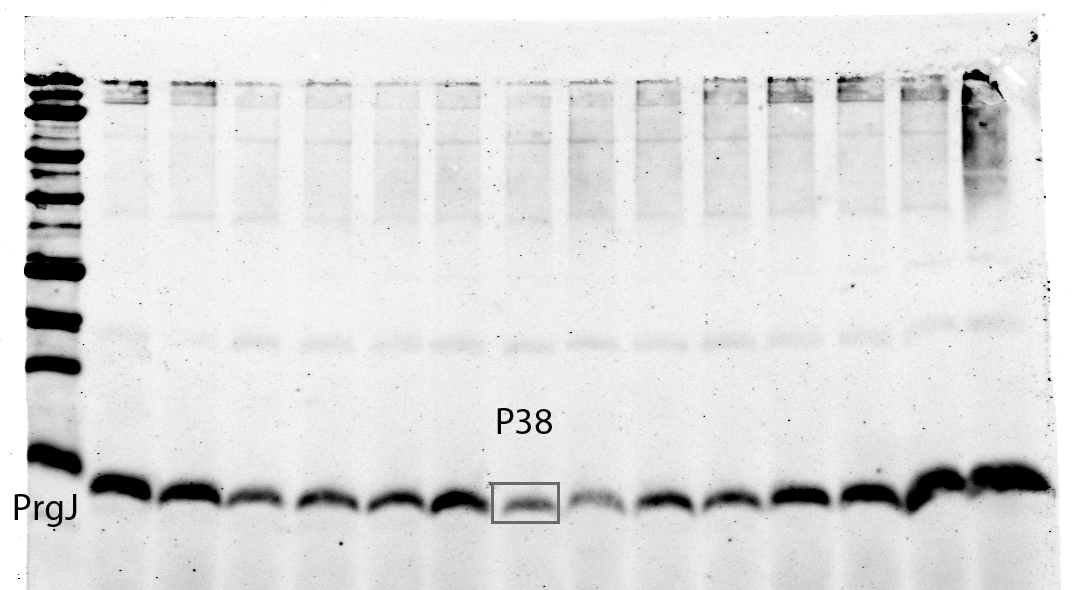

Supplement: S16 Data — (ZIP) [file pbio.3000351.s036.zip › S16-data/P38-anti-PrgJ.tif]

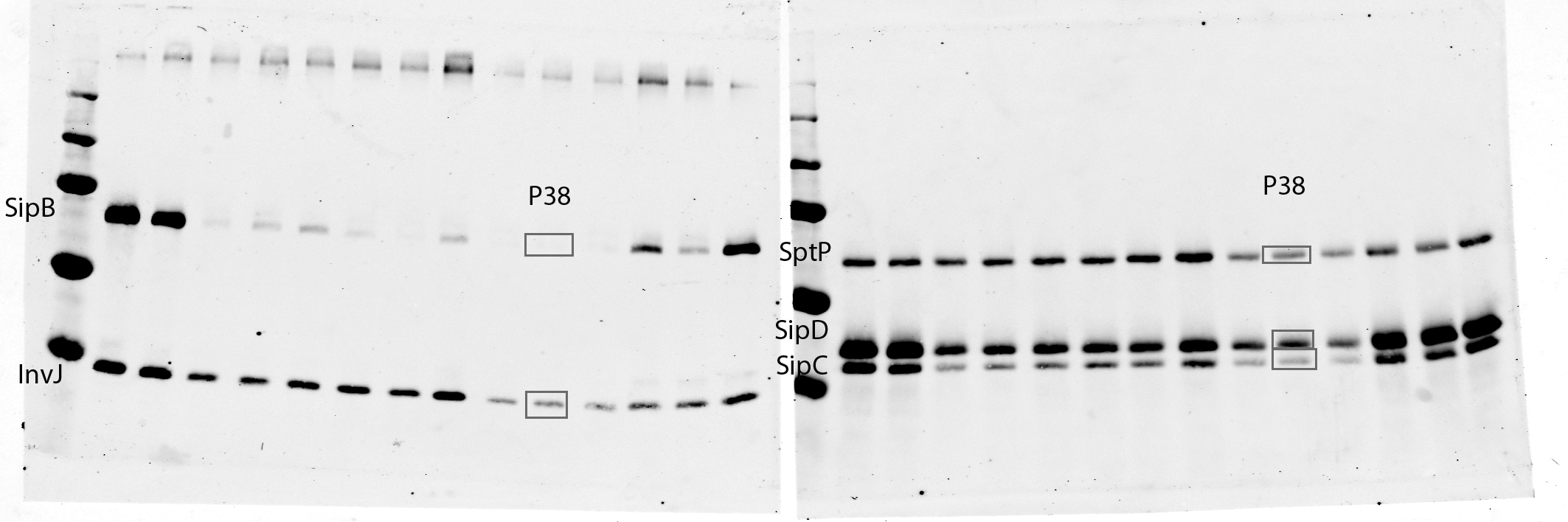

Supplement: S16 Data — (ZIP) [file pbio.3000351.s036.zip › S16-data/P38-anti-SipB-InvJ-SptP-SipD-SipC.tif]

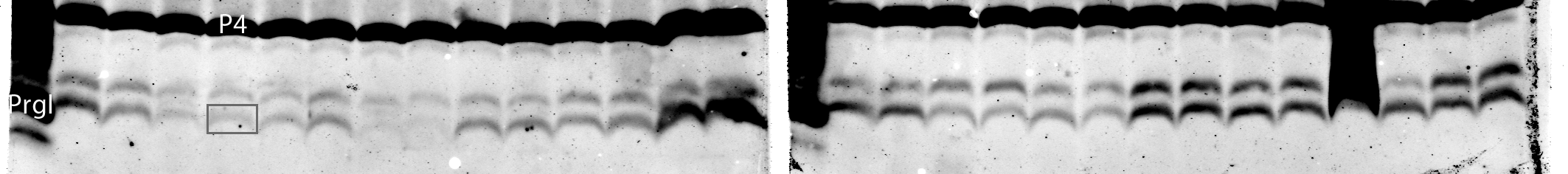

Supplement: S16 Data — (ZIP) [file pbio.3000351.s036.zip › S16-data/P4-anti-PrgI.tif]

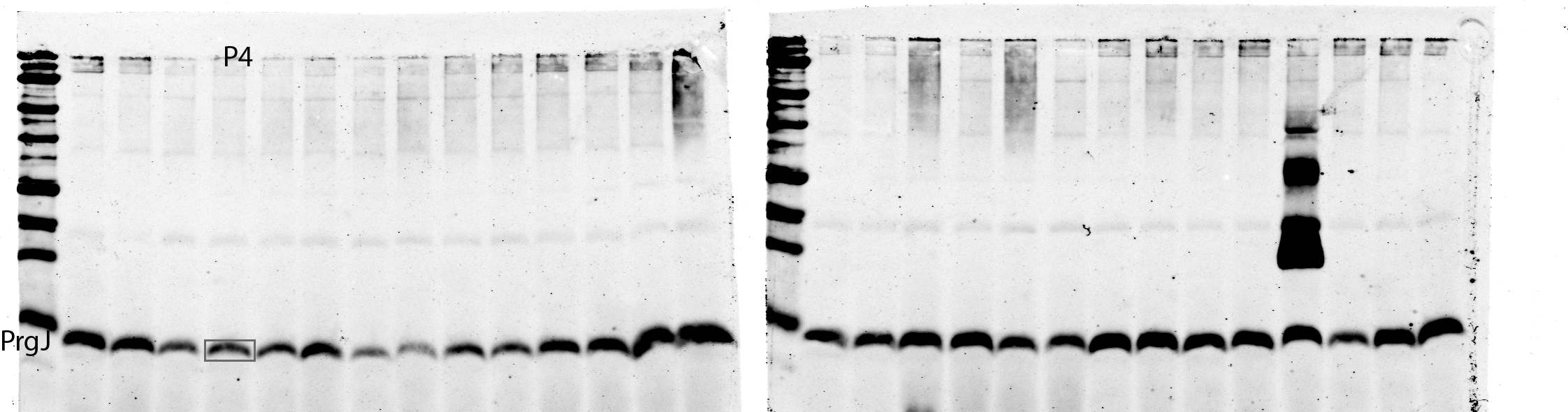

Supplement: S16 Data — (ZIP) [file pbio.3000351.s036.zip › S16-data/P4-anti-PrgJ.tif]

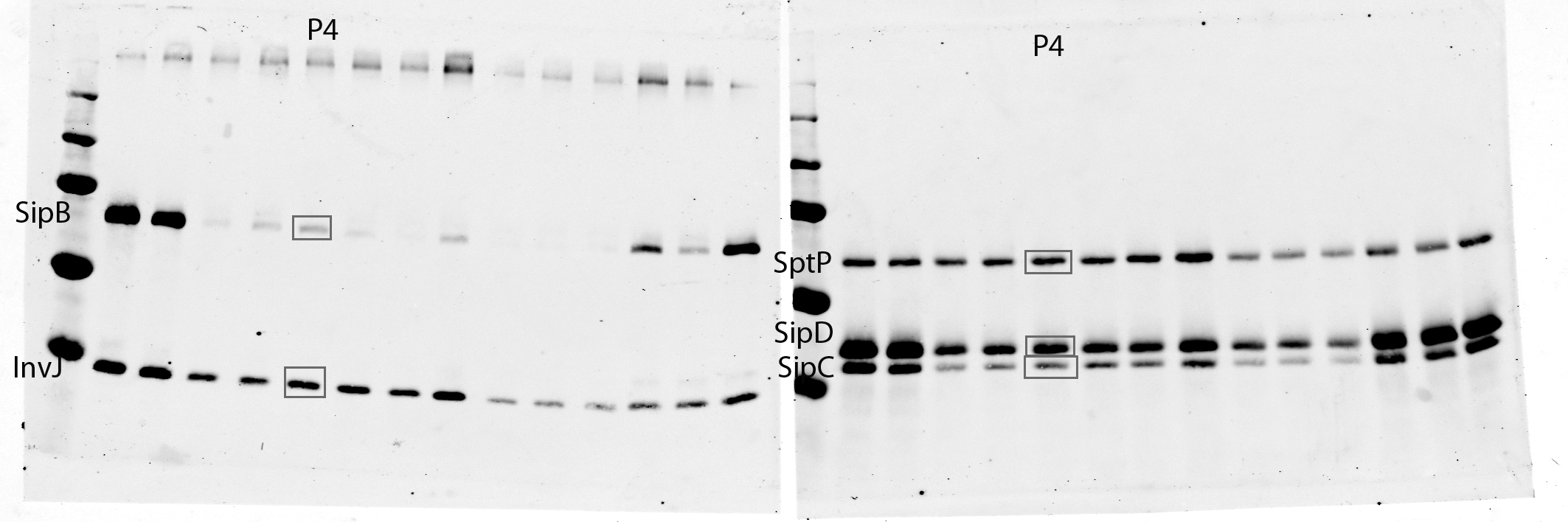

Supplement: S16 Data — (ZIP) [file pbio.3000351.s036.zip › S16-data/P4-anti-SipB-InvJ-SptP-SipD-SipC.tif]

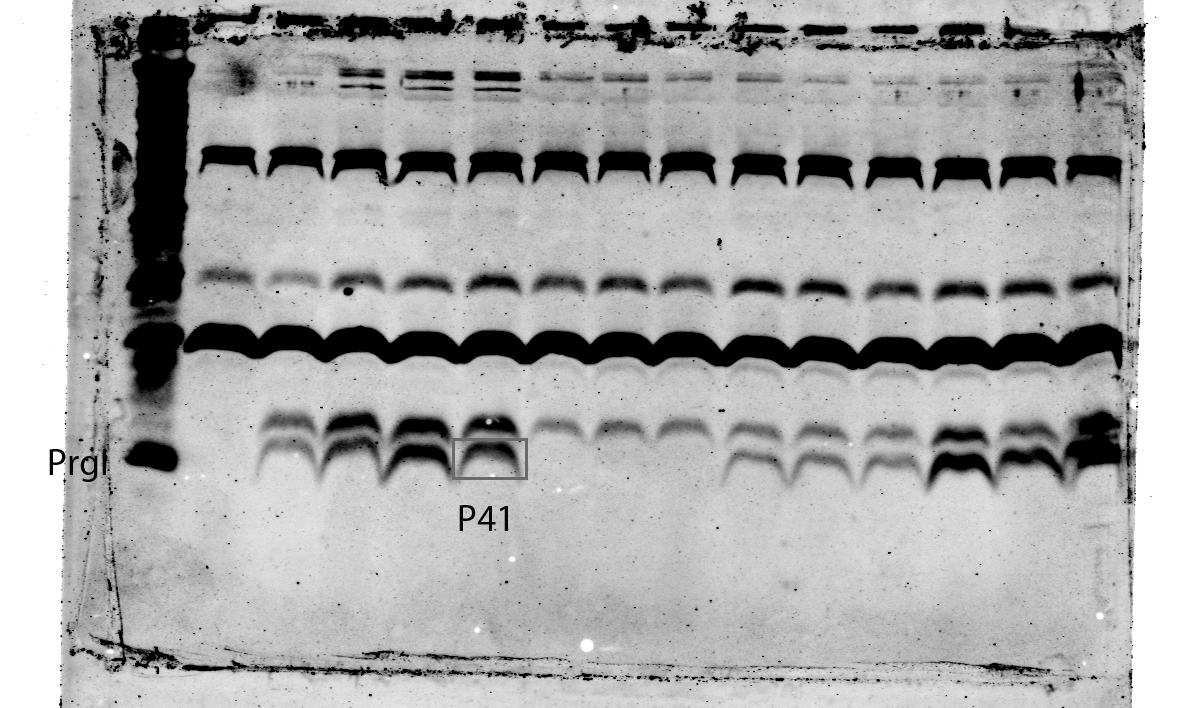

Supplement: S16 Data — (ZIP) [file pbio.3000351.s036.zip › S16-data/P41-anti-prgI.tif]

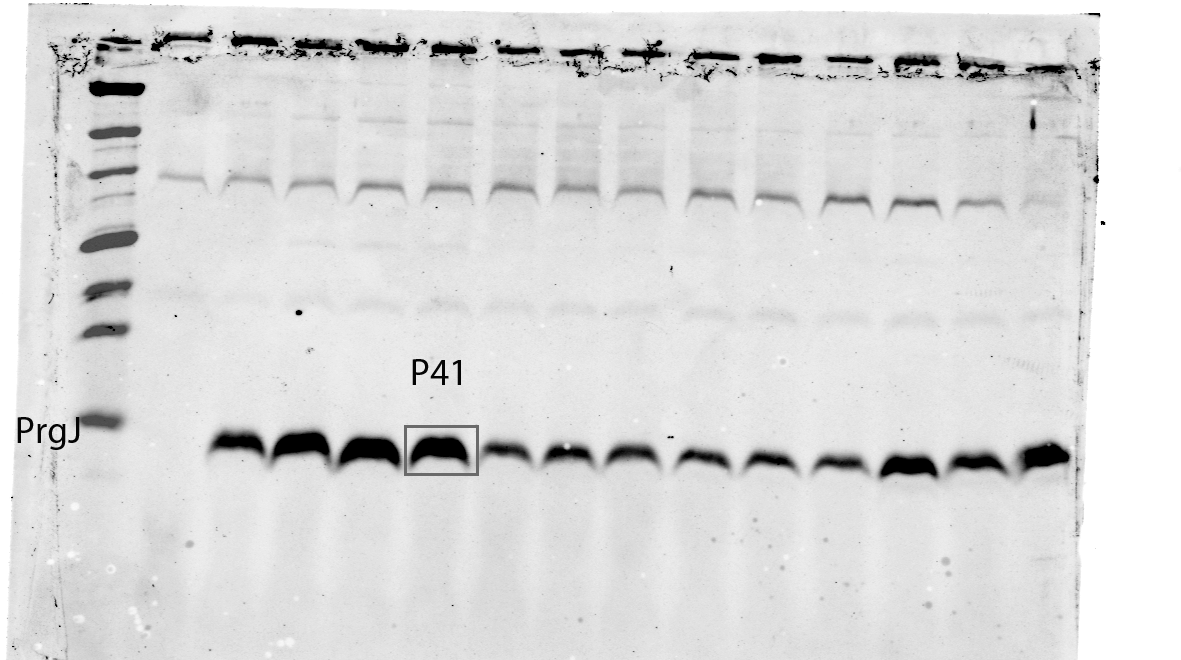

Supplement: S16 Data — (ZIP) [file pbio.3000351.s036.zip › S16-data/P41-anti-prgJ.tif]

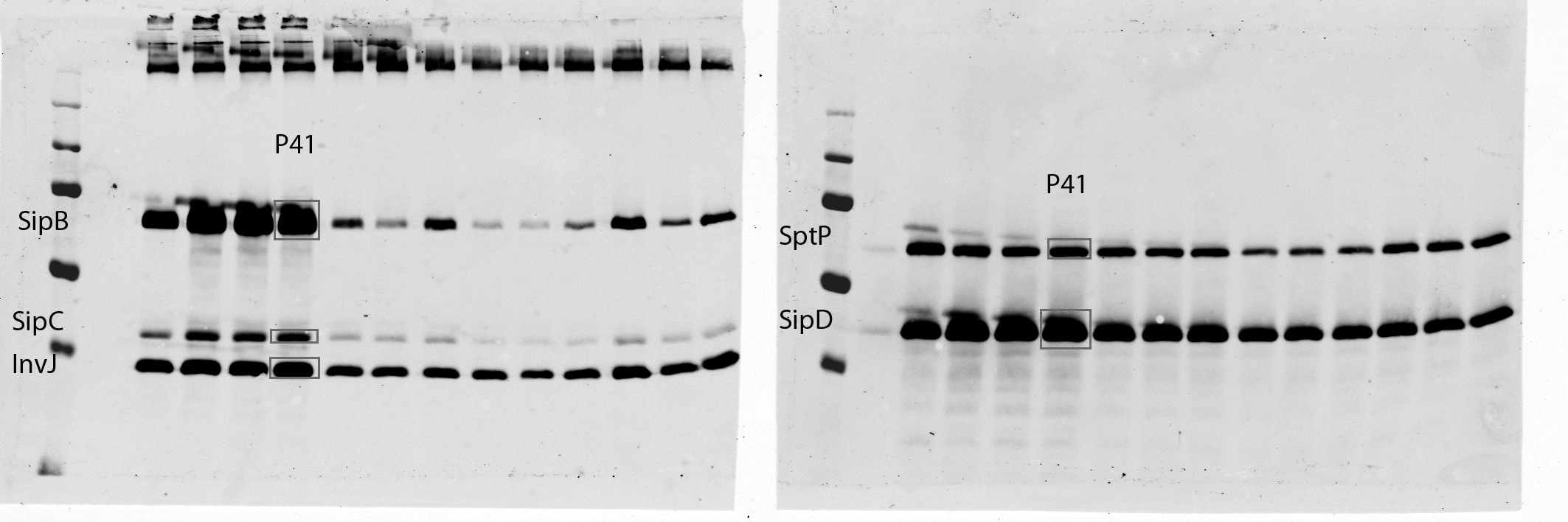

Supplement: S16 Data — (ZIP) [file pbio.3000351.s036.zip › S16-data/P41-anti-SipB-SipC-InvJ-SptP-SipD.tif]

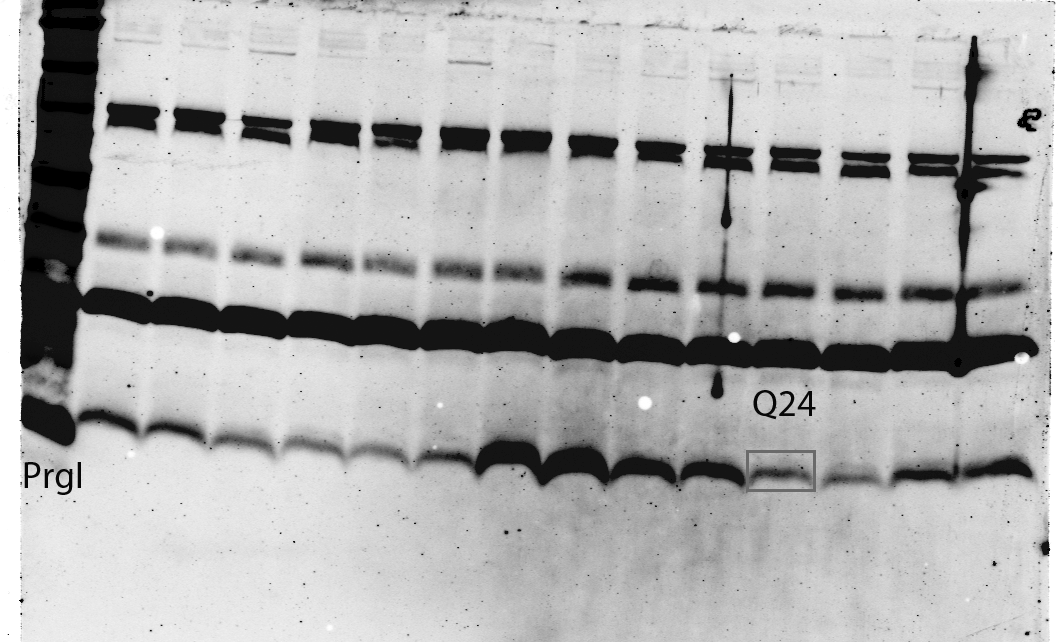

Supplement: S17 Data — (ZIP) [file pbio.3000351.s037.zip › S17-data/Q24-anti-prgI.tif]

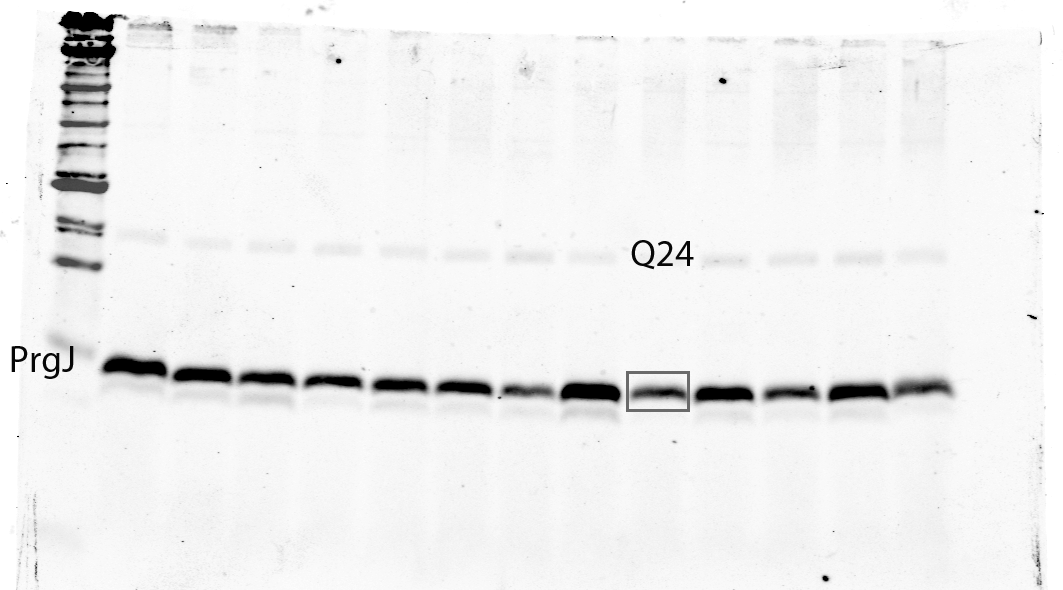

Supplement: S17 Data — (ZIP) [file pbio.3000351.s037.zip › S17-data/Q24-anti-PrgJ.tif]

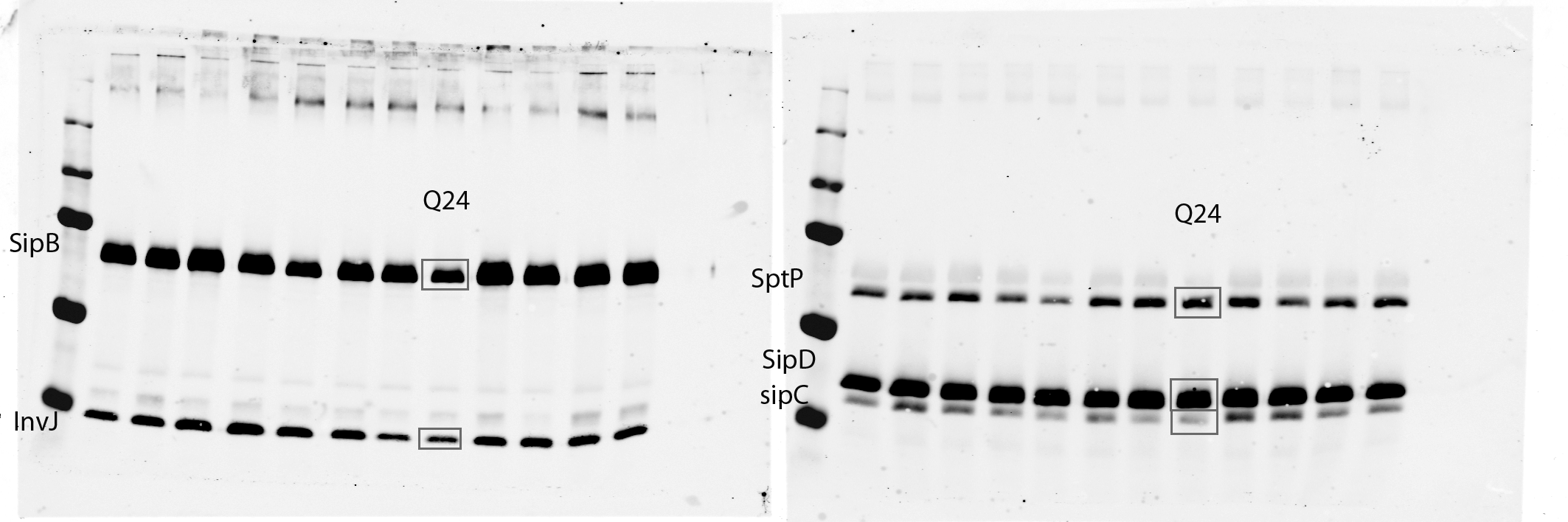

Supplement: S17 Data — (ZIP) [file pbio.3000351.s037.zip › S17-data/Q24-anti-SipB-InvJ-SptP-SipD-SipC.tif]

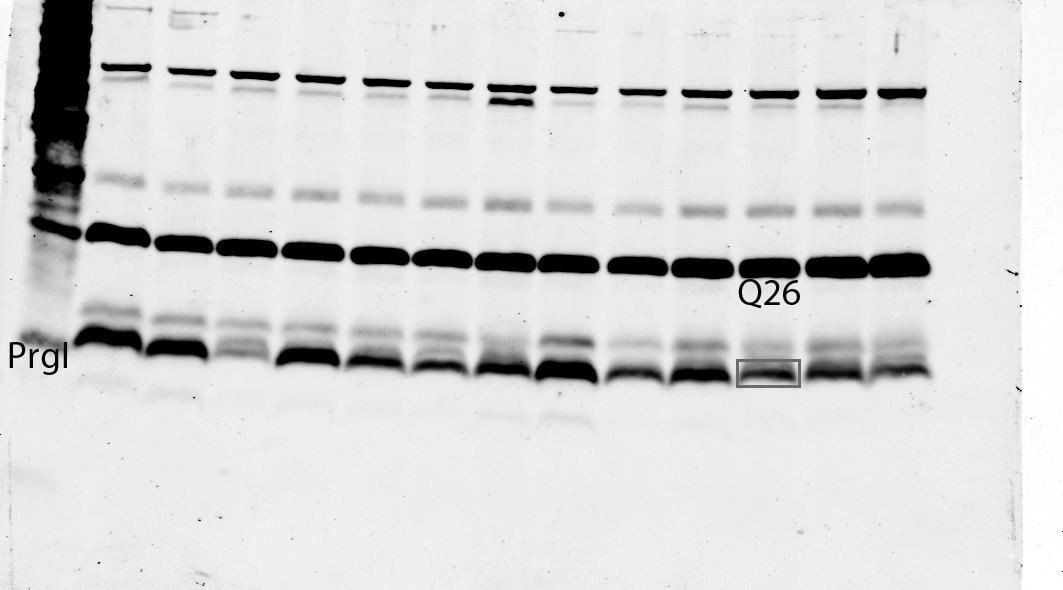

Supplement: S17 Data — (ZIP) [file pbio.3000351.s037.zip › S17-data/Q26-anti-PrgI.tif]

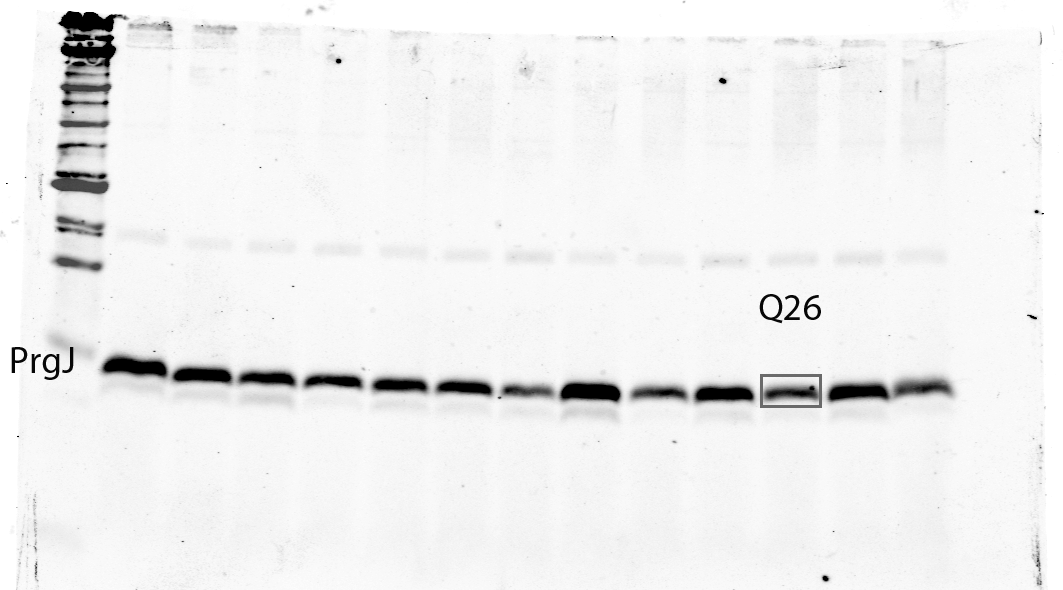

Supplement: S17 Data — (ZIP) [file pbio.3000351.s037.zip › S17-data/Q26-anti-PrgJ.tif]

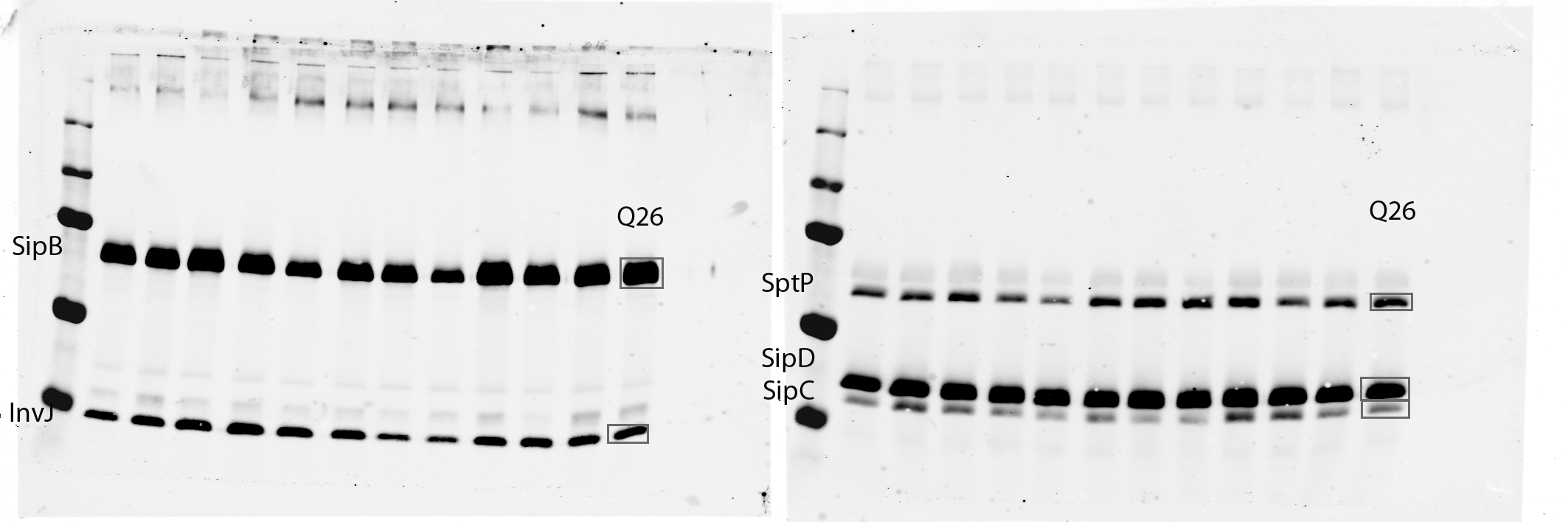

Supplement: S17 Data — (ZIP) [file pbio.3000351.s037.zip › S17-data/Q26-anti-SipB-InvJ-SptP-SipD-SipC.tif]

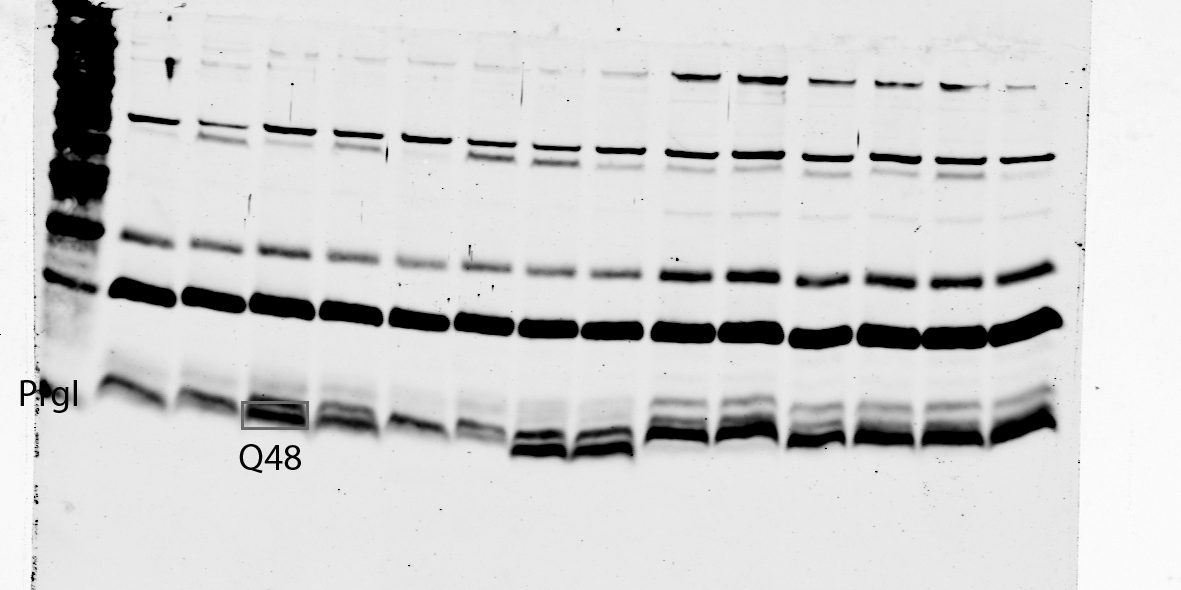

Supplement: S17 Data — (ZIP) [file pbio.3000351.s037.zip › S17-data/Q48-anti-prgI.tif]

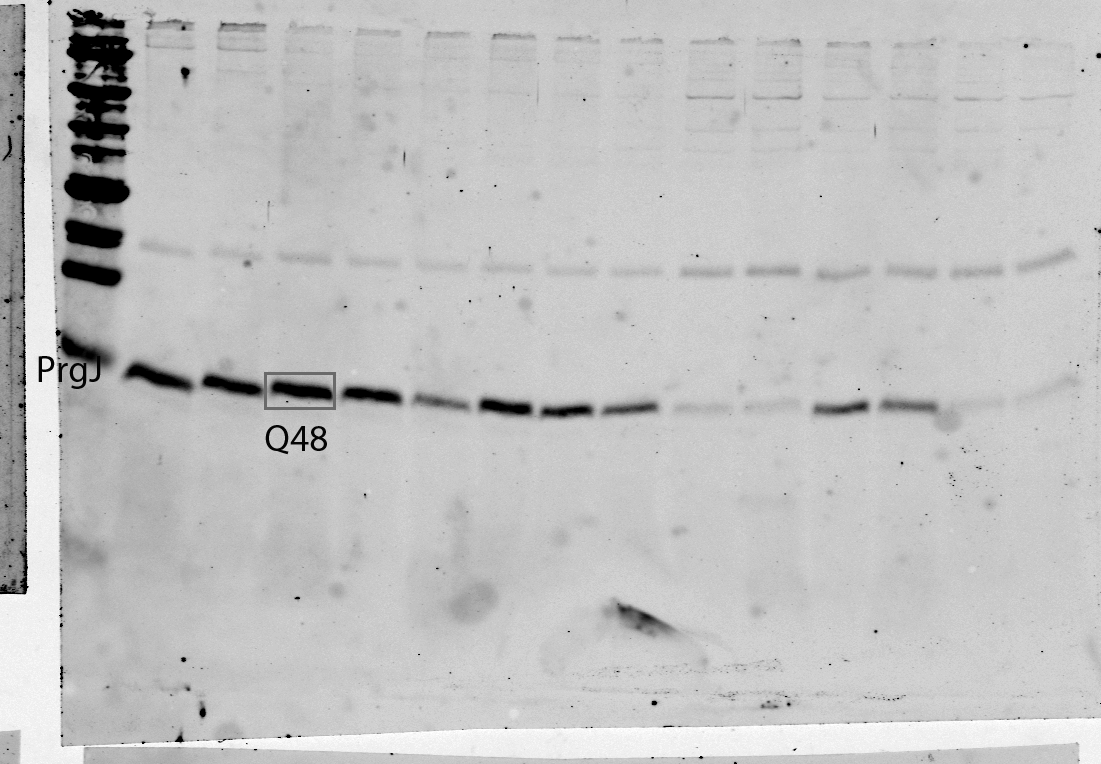

Supplement: S17 Data — (ZIP) [file pbio.3000351.s037.zip › S17-data/Q48-anti-prgJ.tif]

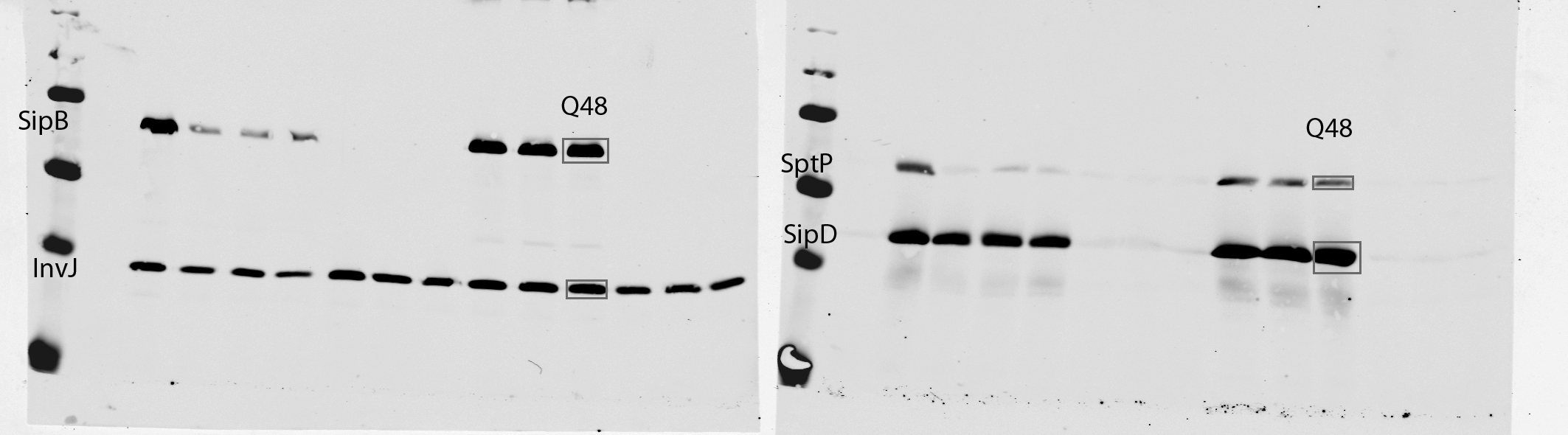

Supplement: S17 Data — (ZIP) [file pbio.3000351.s037.zip › S17-data/Q48-anti-SipB-InvJ-SptP-SipD.tif]

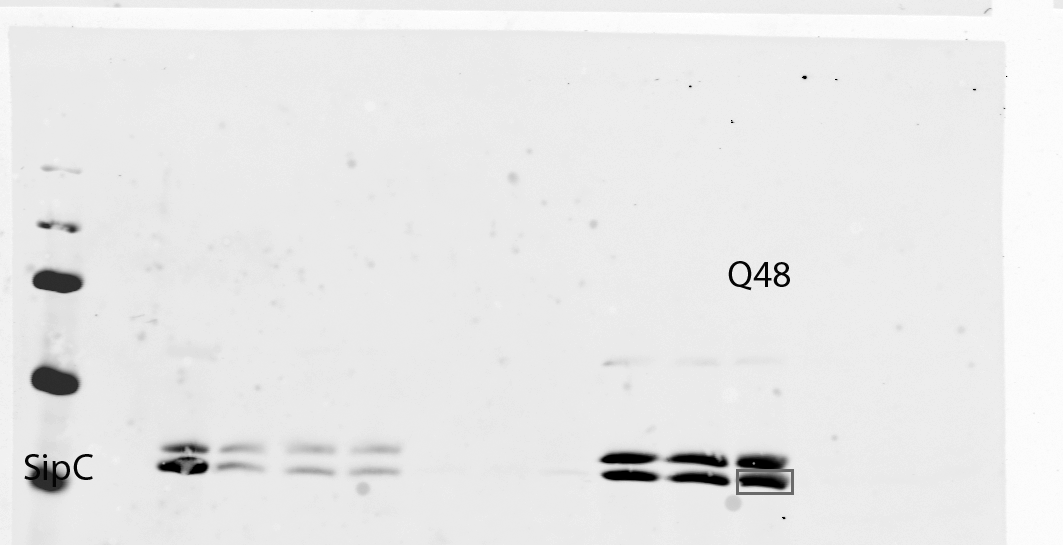

Supplement: S17 Data — (ZIP) [file pbio.3000351.s037.zip › S17-data/Q48-anti-sipC.tif]

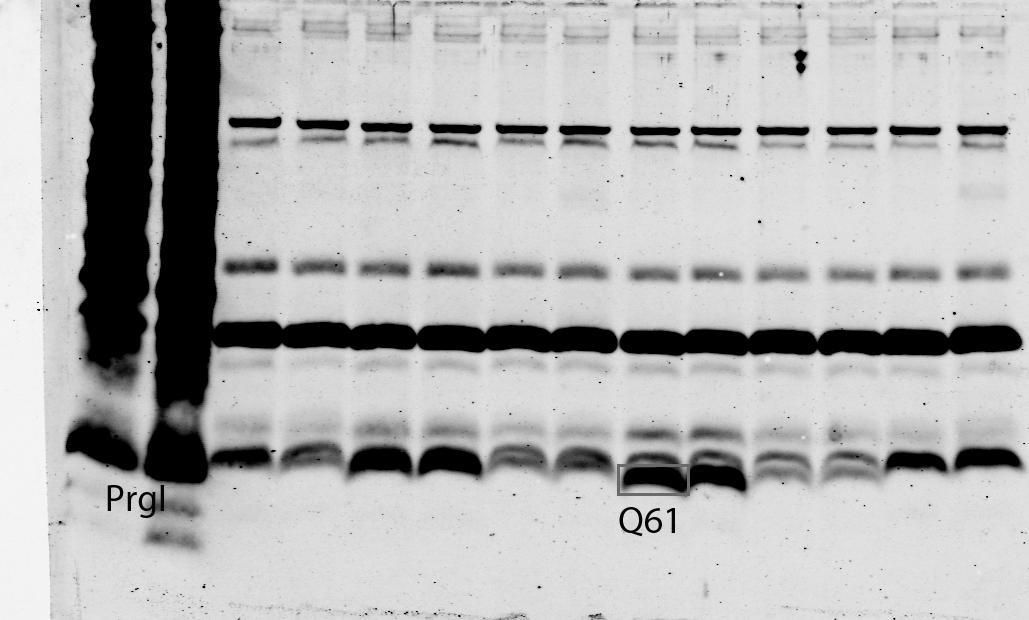

Supplement: S17 Data — (ZIP) [file pbio.3000351.s037.zip › S17-data/Q61-anti-PrgI.tif]

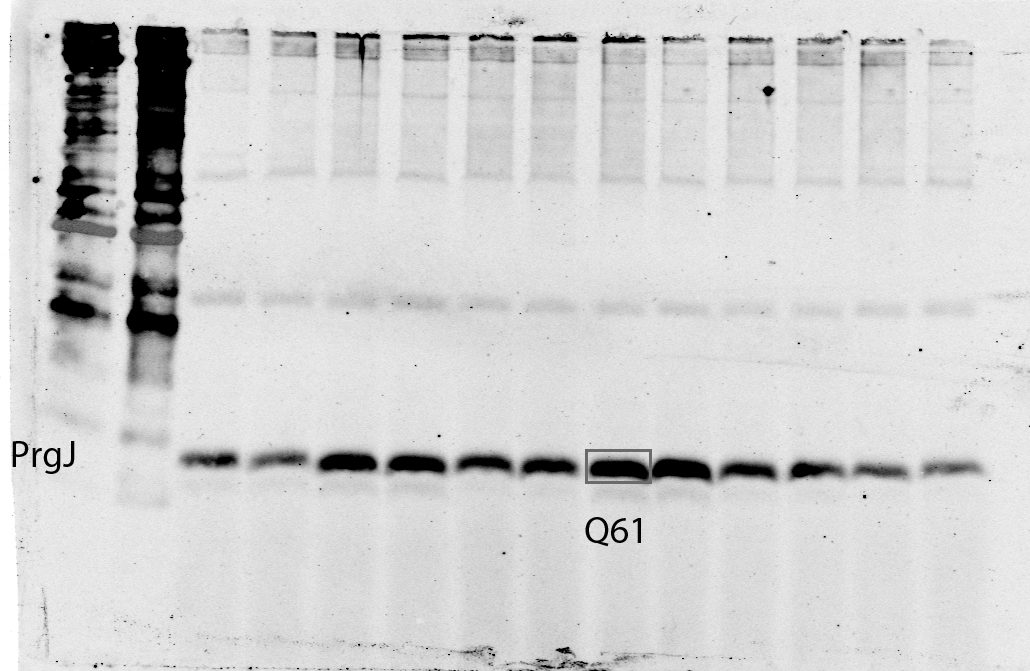

Supplement: S17 Data — (ZIP) [file pbio.3000351.s037.zip › S17-data/Q61-anti-PrgJ.tif]

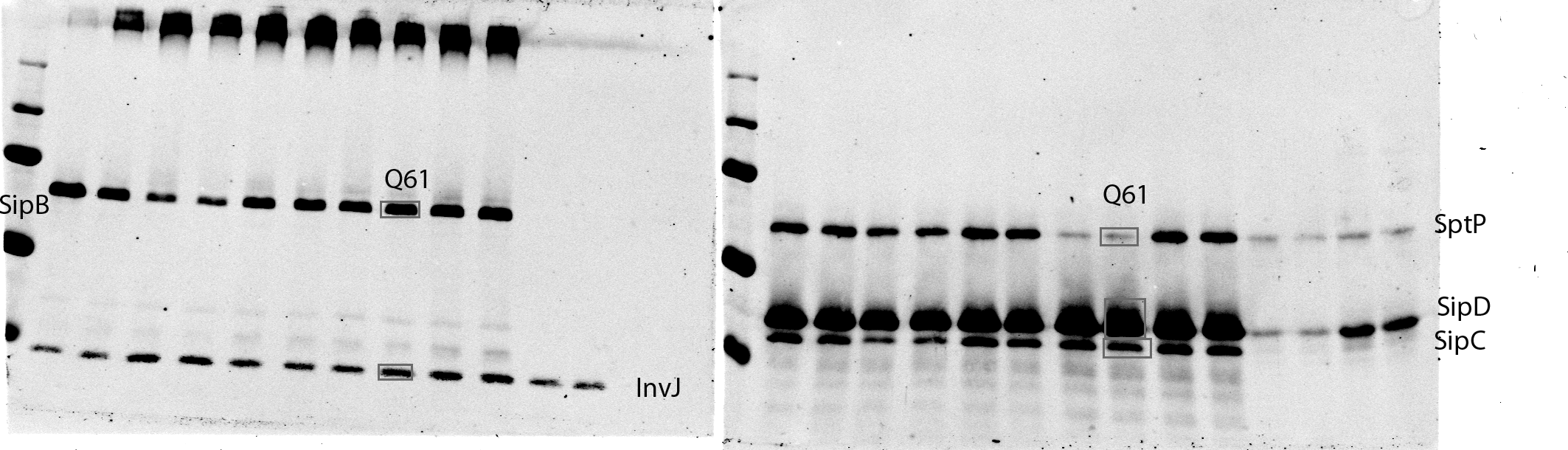

Supplement: S17 Data — (ZIP) [file pbio.3000351.s037.zip › S17-data/Q61-anti-SipB-InvJ-SptP-SipD-SipC.tif]

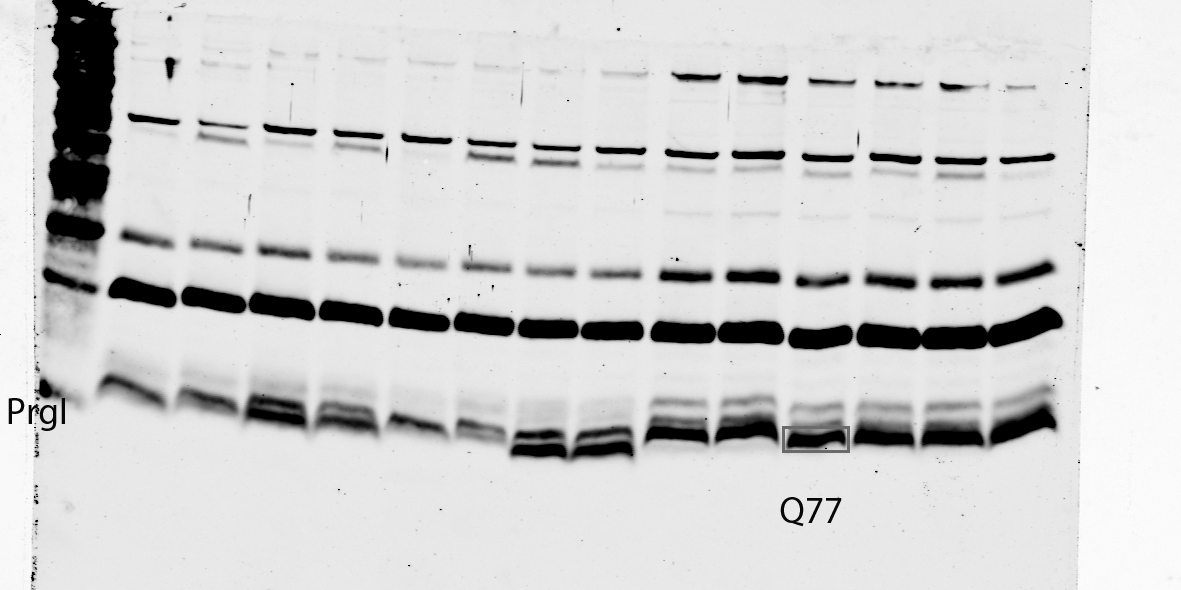

Supplement: S17 Data — (ZIP) [file pbio.3000351.s037.zip › S17-data/Q77-anti-prgI.tif]

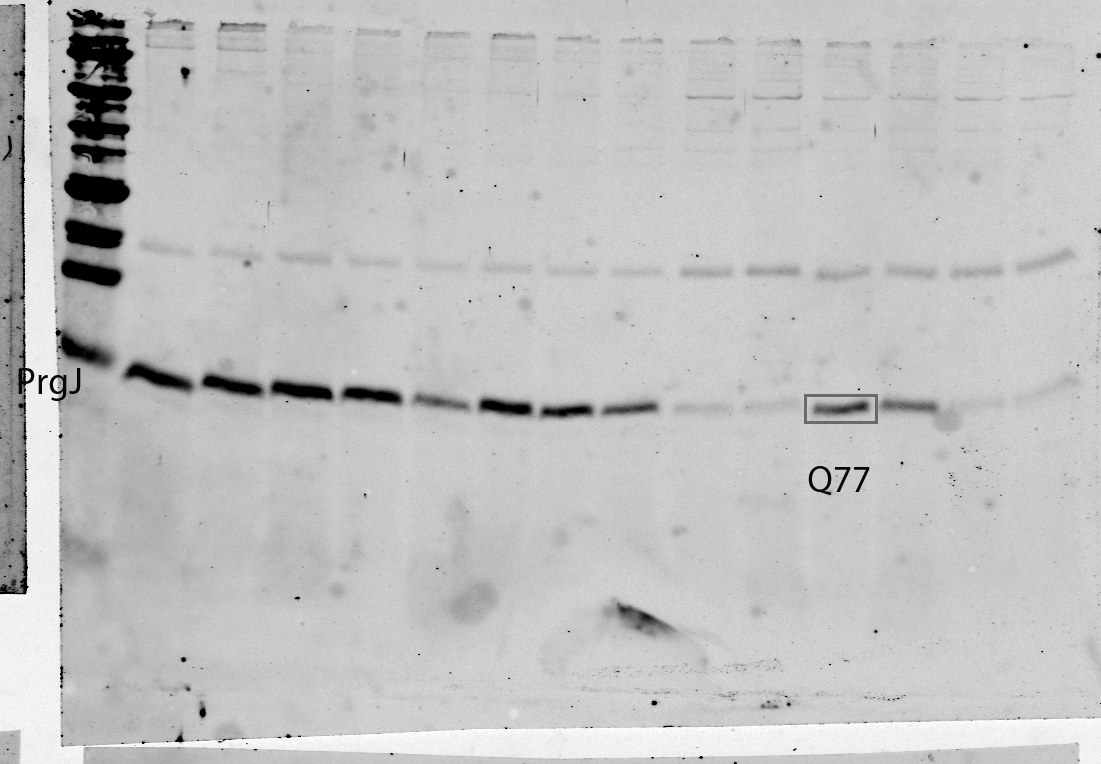

Supplement: S17 Data — (ZIP) [file pbio.3000351.s037.zip › S17-data/Q77-anti-prgJ.tif]

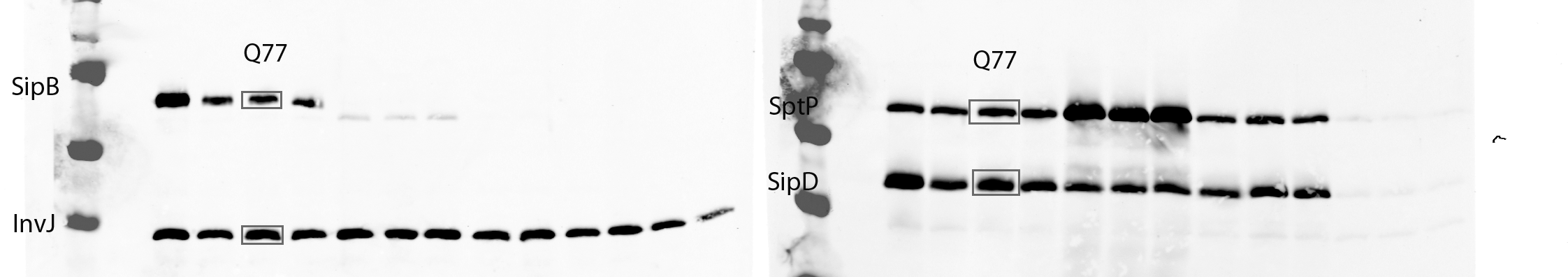

Supplement: S17 Data — (ZIP) [file pbio.3000351.s037.zip › S17-data/Q77-anti-SipB-InvJ-SptP-SipD.tif]

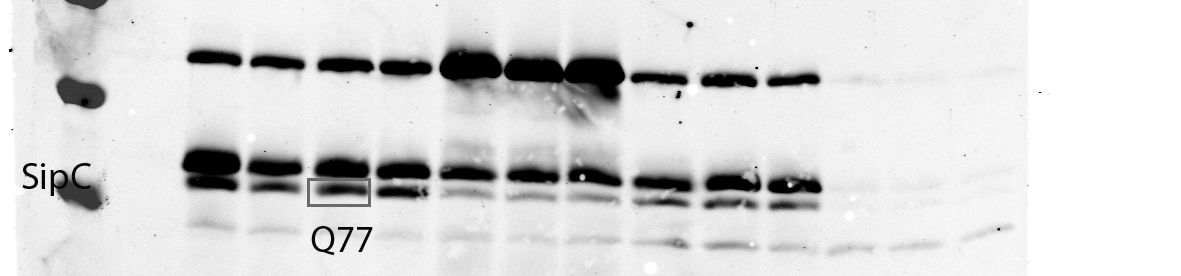

Supplement: S17 Data — (ZIP) [file pbio.3000351.s037.zip › S17-data/Q77-anti-sipC.tif]

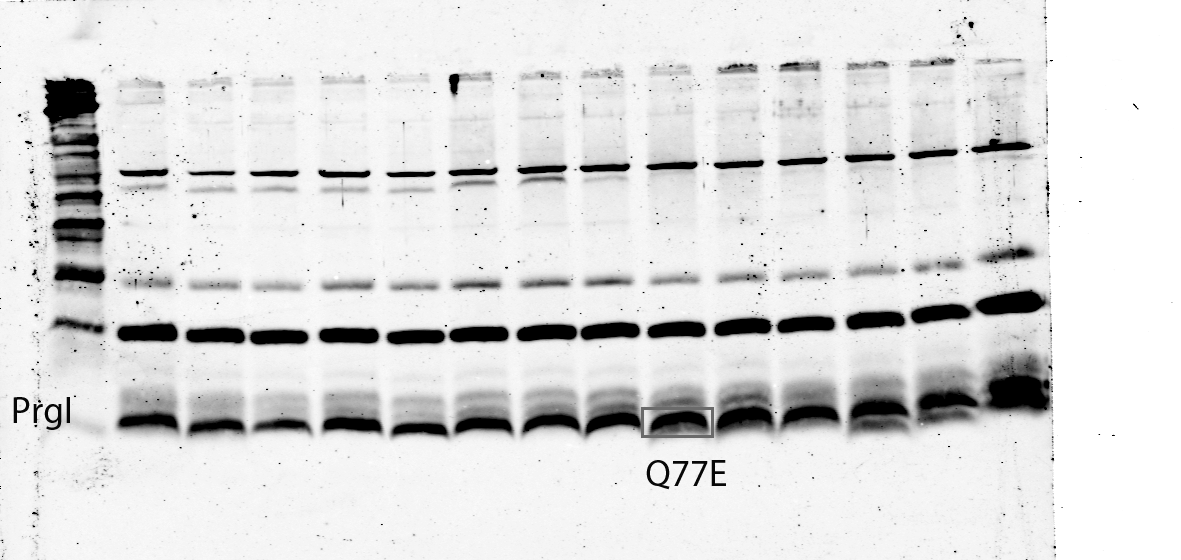

Supplement: S17 Data — (ZIP) [file pbio.3000351.s037.zip › S17-data/Q77E-anti-prgI.tif]

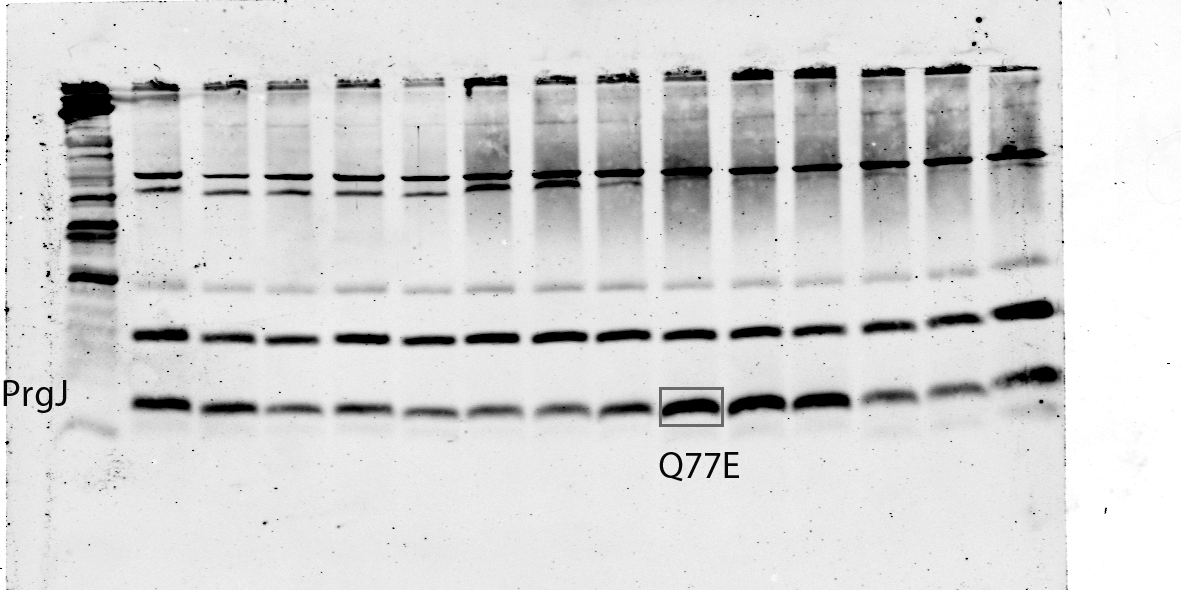

Supplement: S17 Data — (ZIP) [file pbio.3000351.s037.zip › S17-data/Q77E-anti-prgJ.tif]

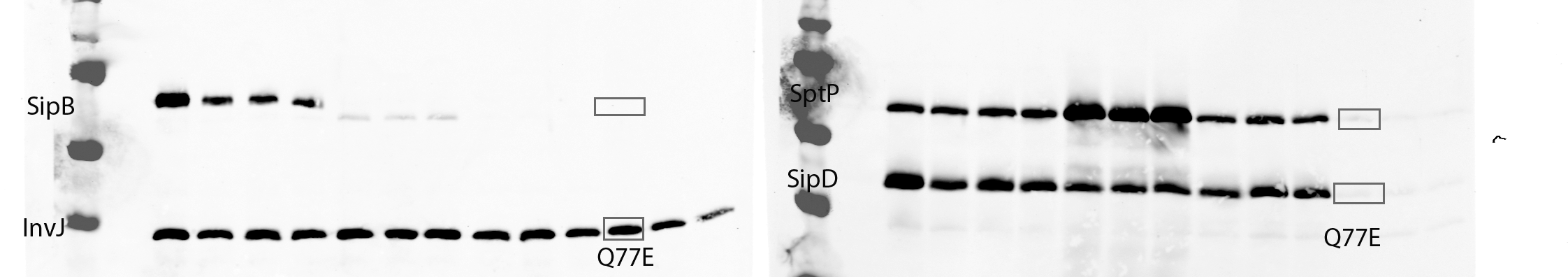

Supplement: S17 Data — (ZIP) [file pbio.3000351.s037.zip › S17-data/Q77E-anti-SipB-InvJ-SptP-SipD.tif]

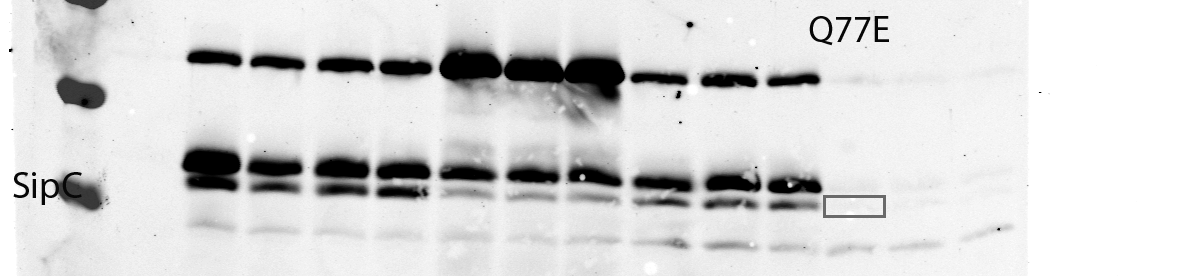

Supplement: S17 Data — (ZIP) [file pbio.3000351.s037.zip › S17-data/Q77E-anti-sipC.tif]

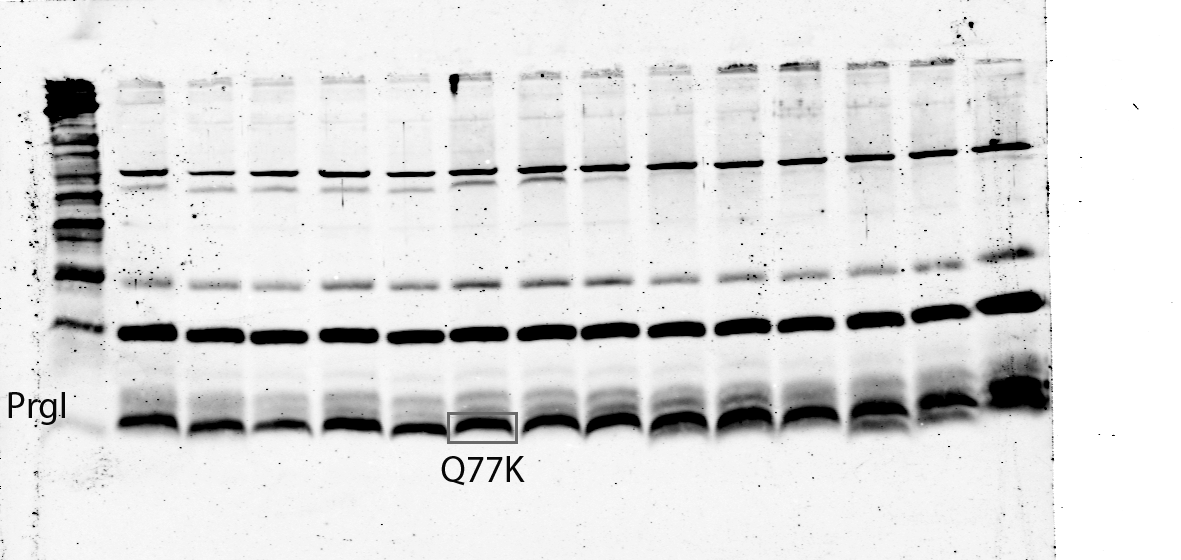

Supplement: S17 Data — (ZIP) [file pbio.3000351.s037.zip › S17-data/Q77K-anti-prgI.tif]

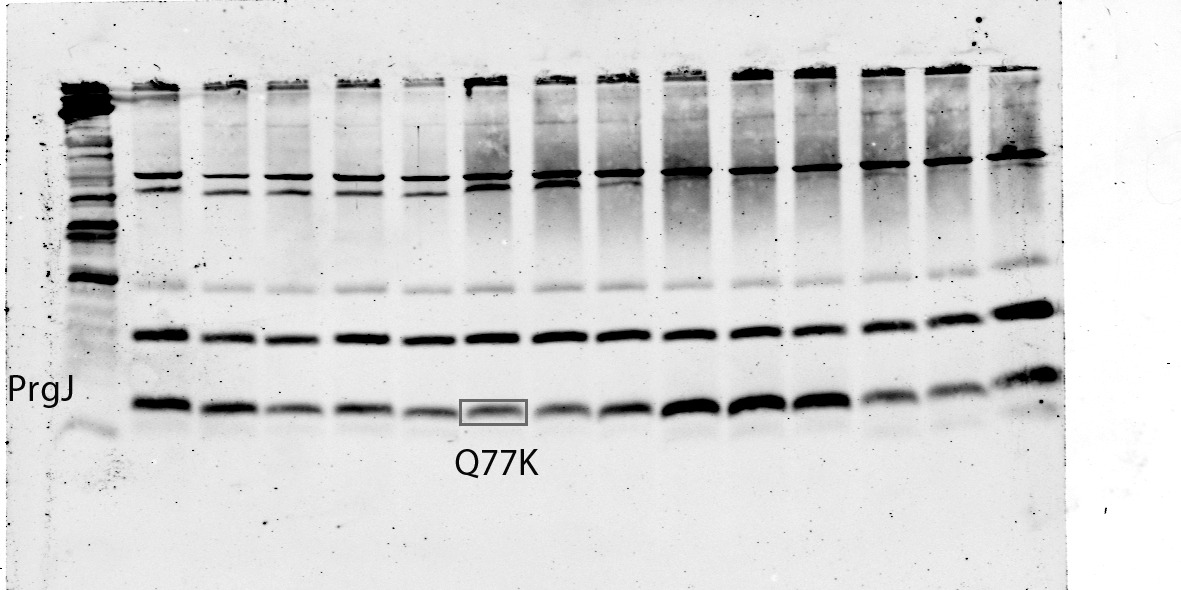

Supplement: S17 Data — (ZIP) [file pbio.3000351.s037.zip › S17-data/Q77K-anti-prgJ.tif]

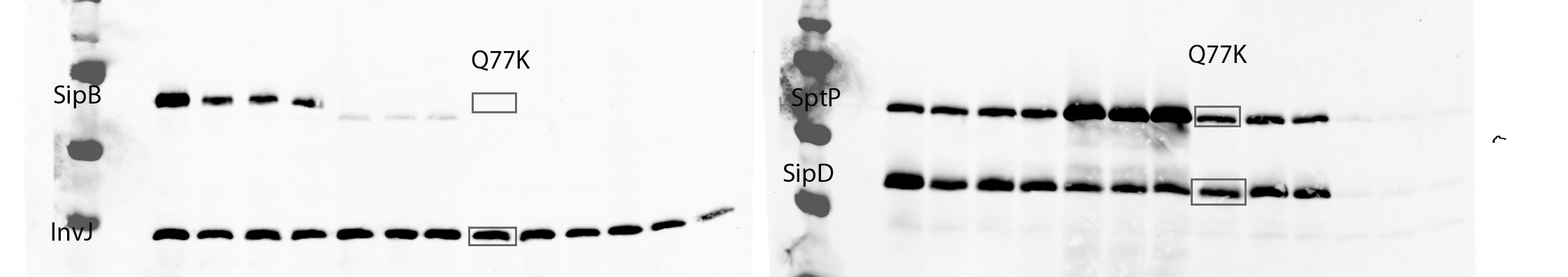

Supplement: S17 Data — (ZIP) [file pbio.3000351.s037.zip › S17-data/Q77K-anti-SipB-InvJ-SptP-SipD.tif]
