# Supplementary figures and images for: A polymorphic helix of a Salmonella needle protein relays signals defining distinct steps in type III secretion (part 2 of 3)
Source: PLoS Biol. 2019 Jul 1;17(7):e3000351. doi: 10.1371/journal.pbio.3000351 (PMC6625726; doi:10.1371/journal.pbio.3000351)

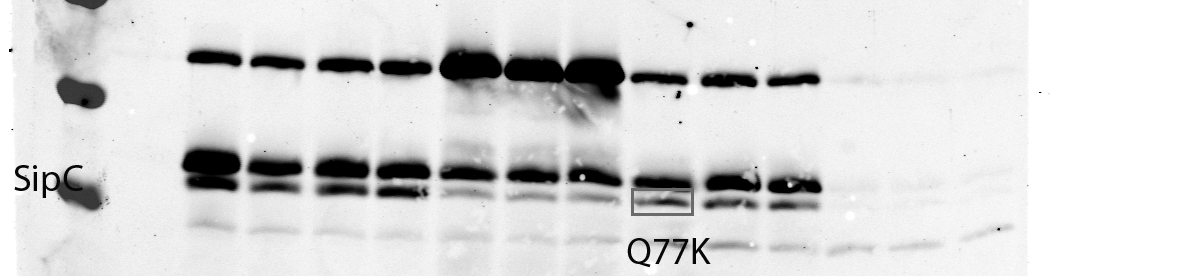

Supplement: S17 Data — (ZIP) [file pbio.3000351.s037.zip › S17-data/Q77K-anti-sipC.tif]

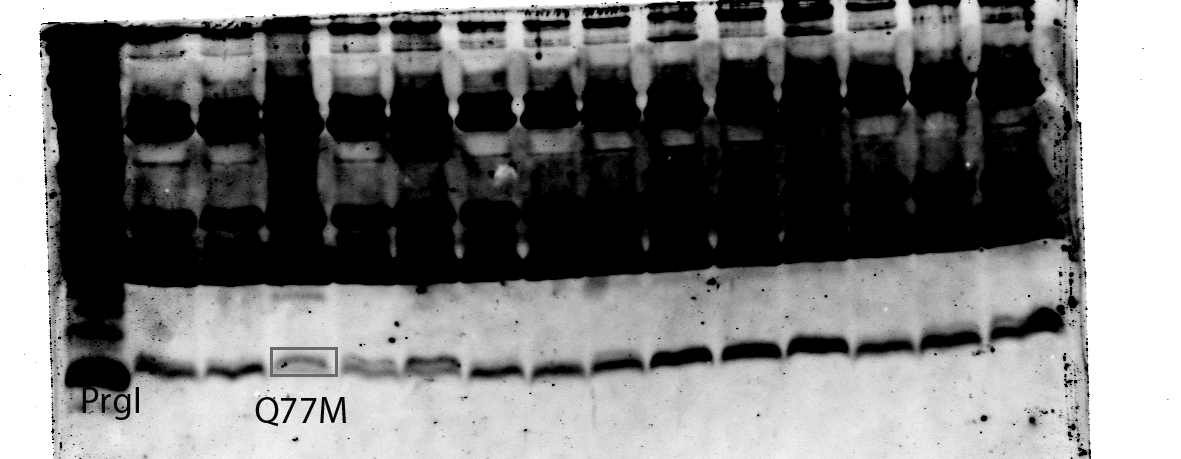

Supplement: S17 Data — (ZIP) [file pbio.3000351.s037.zip › S17-data/Q77M-anti-prgI.tif]

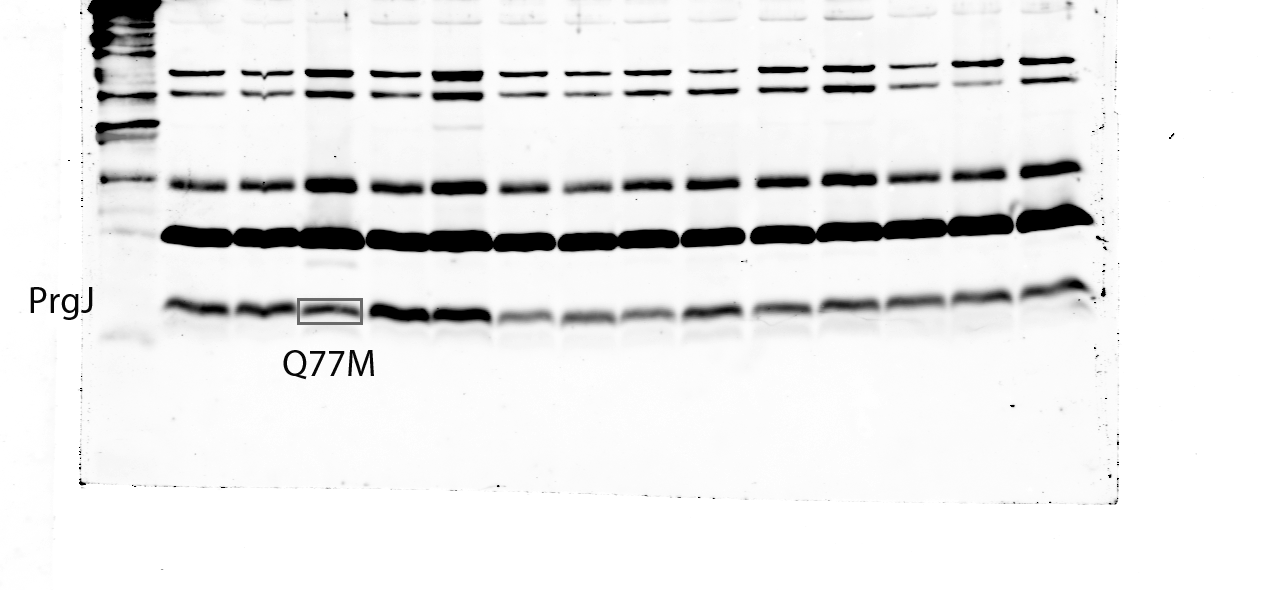

Supplement: S17 Data — (ZIP) [file pbio.3000351.s037.zip › S17-data/Q77M-anti-prgJ.tif]

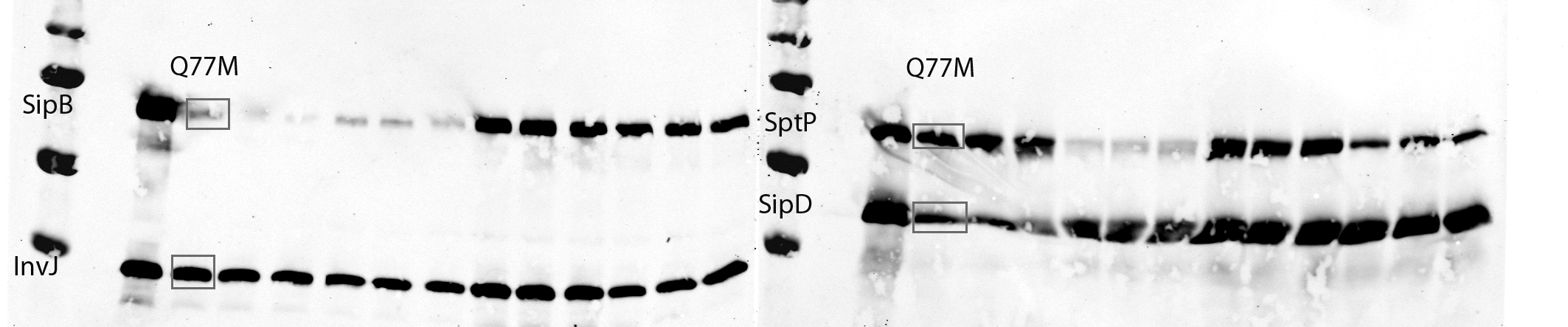

Supplement: S17 Data — (ZIP) [file pbio.3000351.s037.zip › S17-data/Q77M-anti-SipB-InvJ-SptP-SipD.tif]

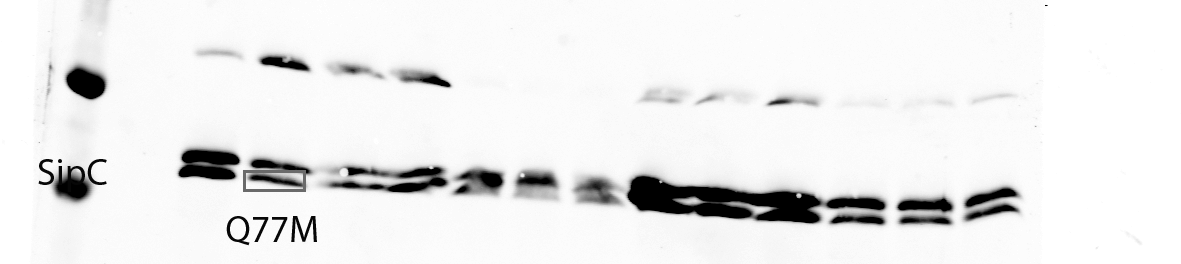

Supplement: S17 Data — (ZIP) [file pbio.3000351.s037.zip › S17-data/Q77M-anti-sipC.tif]

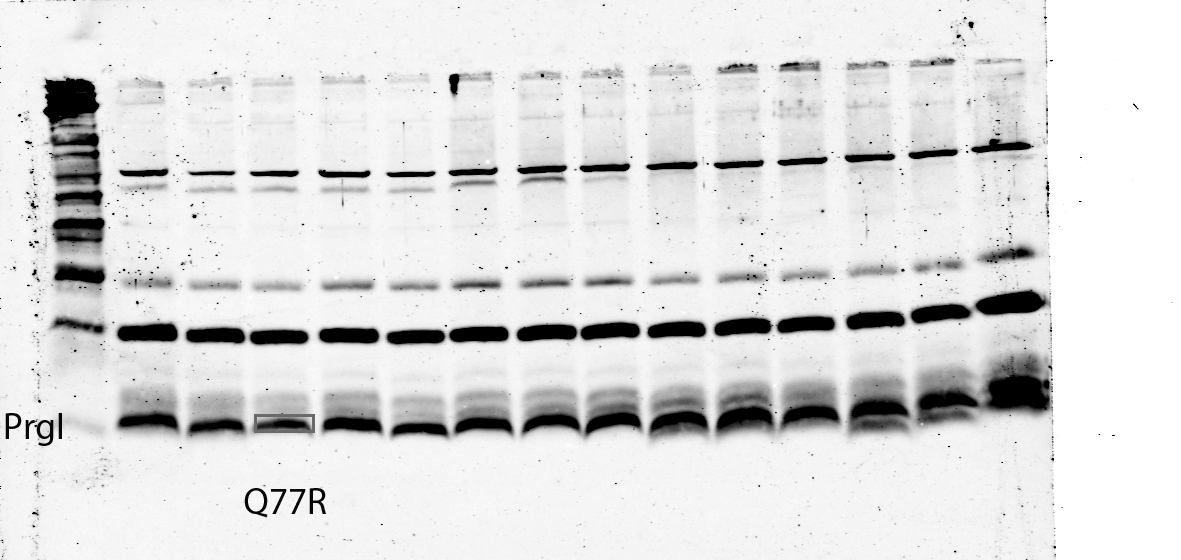

Supplement: S17 Data — (ZIP) [file pbio.3000351.s037.zip › S17-data/Q77R-anti-prgI.tif]

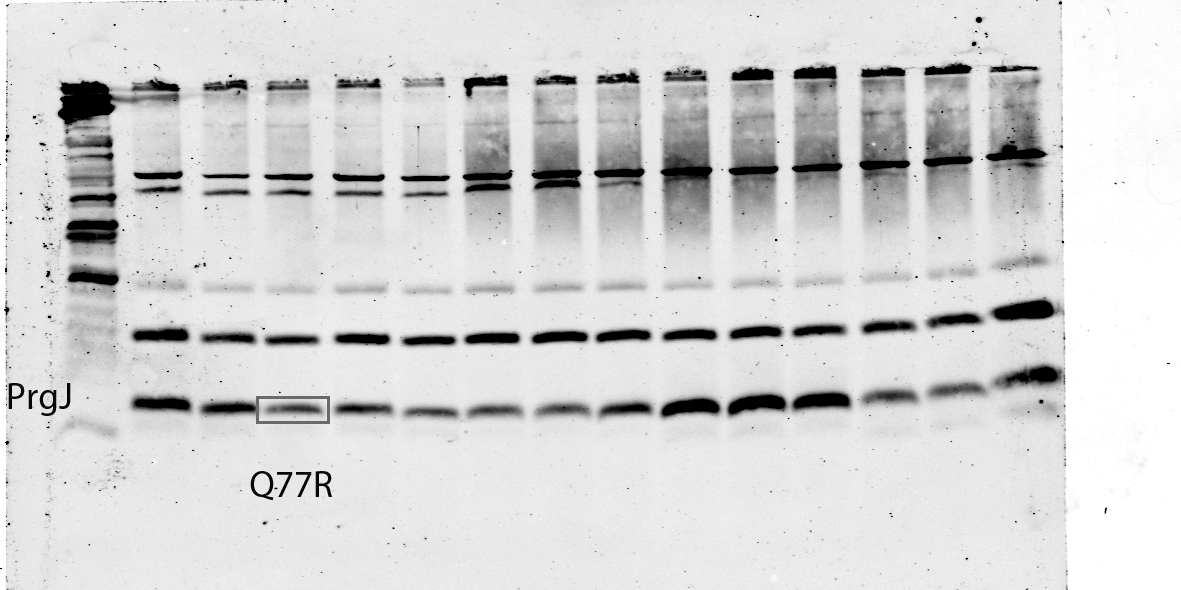

Supplement: S17 Data — (ZIP) [file pbio.3000351.s037.zip › S17-data/Q77R-anti-prgJ.tif]

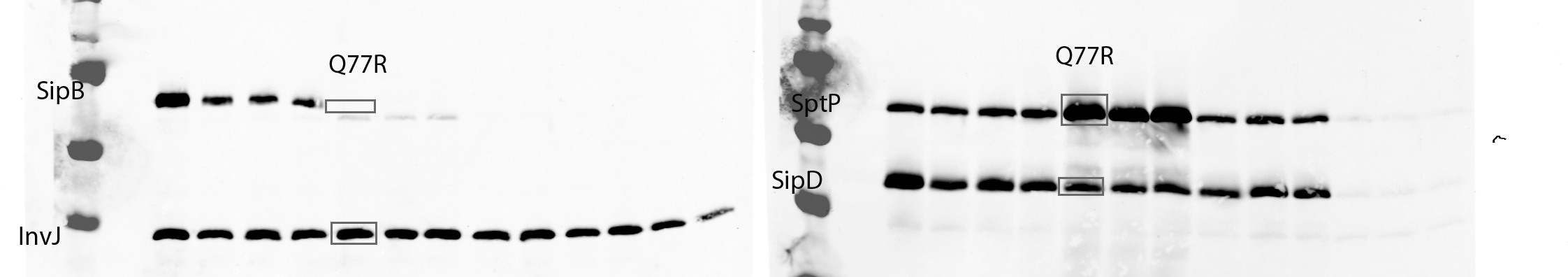

Supplement: S17 Data — (ZIP) [file pbio.3000351.s037.zip › S17-data/Q77R-anti-SipB-InvJ-SptP-SipD.tif]

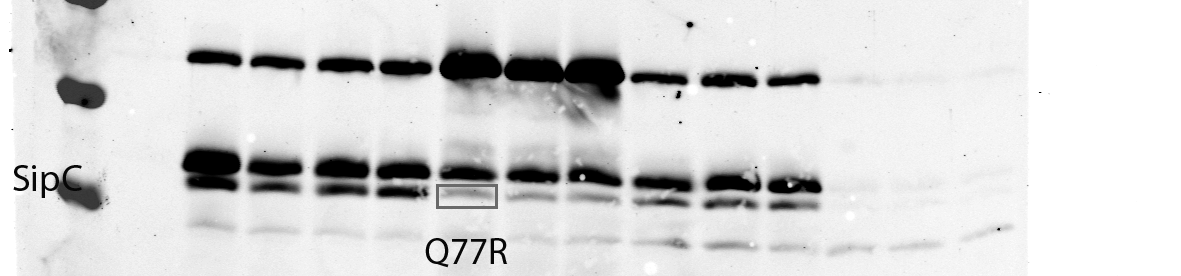

Supplement: S17 Data — (ZIP) [file pbio.3000351.s037.zip › S17-data/Q77R-anti-sipC.tif]

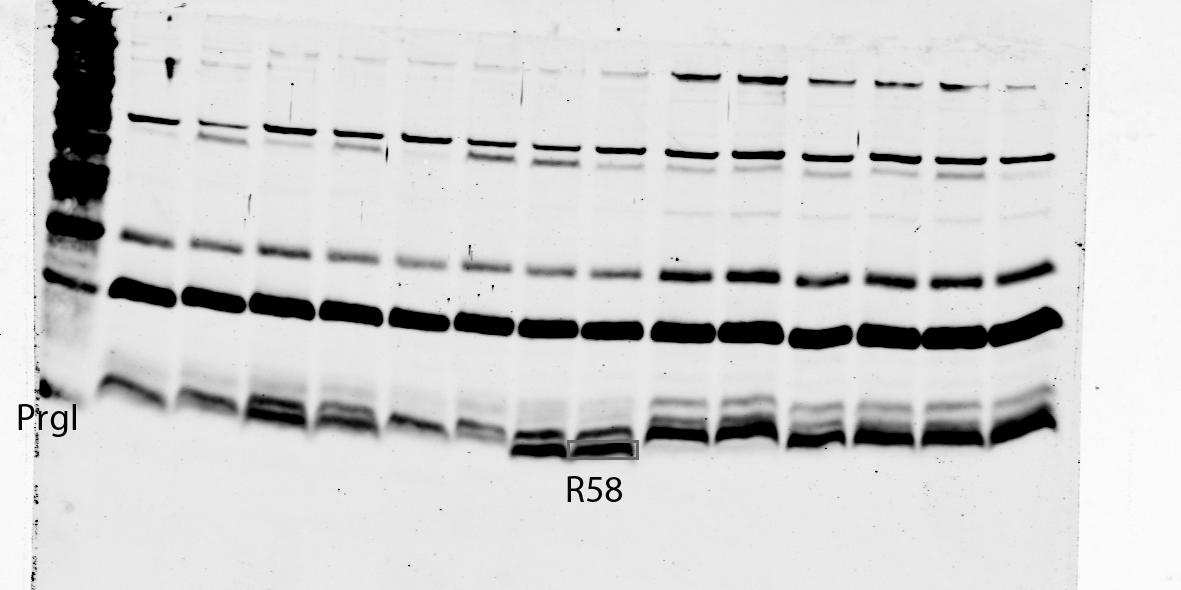

Supplement: S18 Data — (ZIP) [file pbio.3000351.s038.zip › S18-data/R58-anti-prgI.tif]

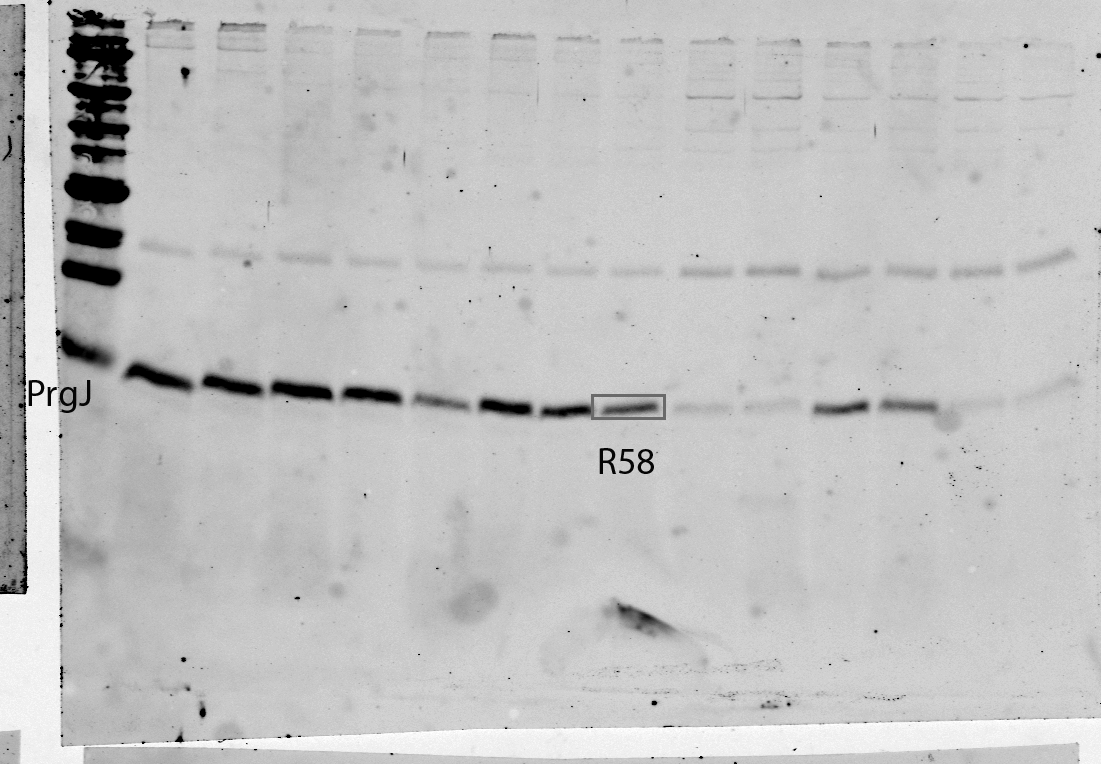

Supplement: S18 Data — (ZIP) [file pbio.3000351.s038.zip › S18-data/R58-anti-prgJ.tif]

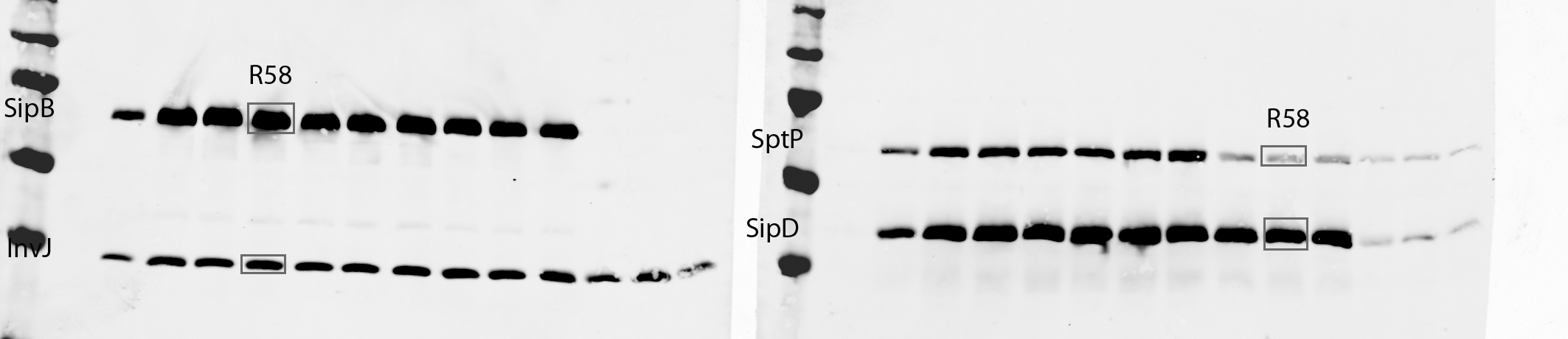

Supplement: S18 Data — (ZIP) [file pbio.3000351.s038.zip › S18-data/R58-anti-SipB-InvJ-SptP-SipD.tif]

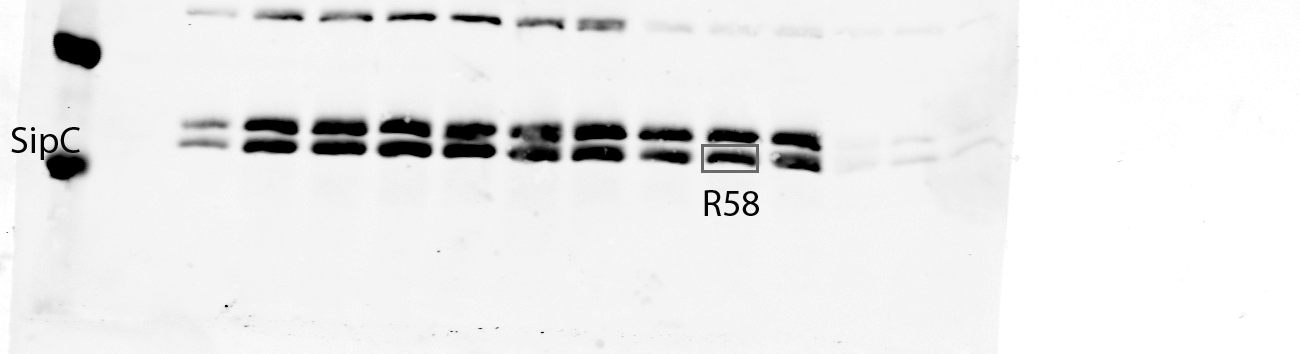

Supplement: S18 Data — (ZIP) [file pbio.3000351.s038.zip › S18-data/R58-anti-sipC.tif]

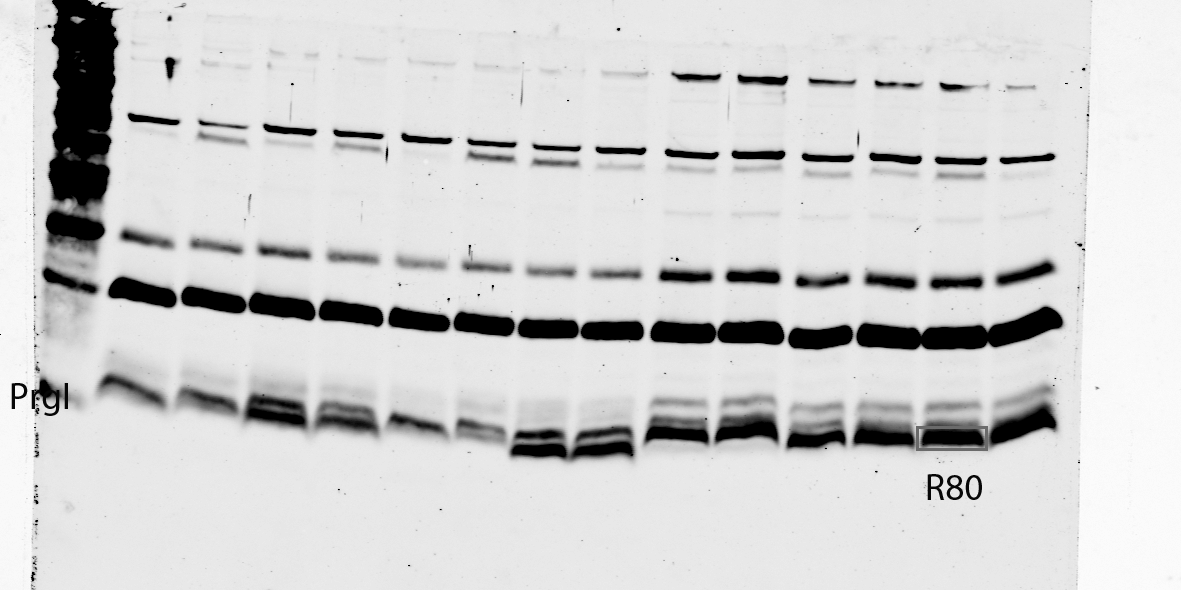

Supplement: S18 Data — (ZIP) [file pbio.3000351.s038.zip › S18-data/R80-anti-prgI.tif]

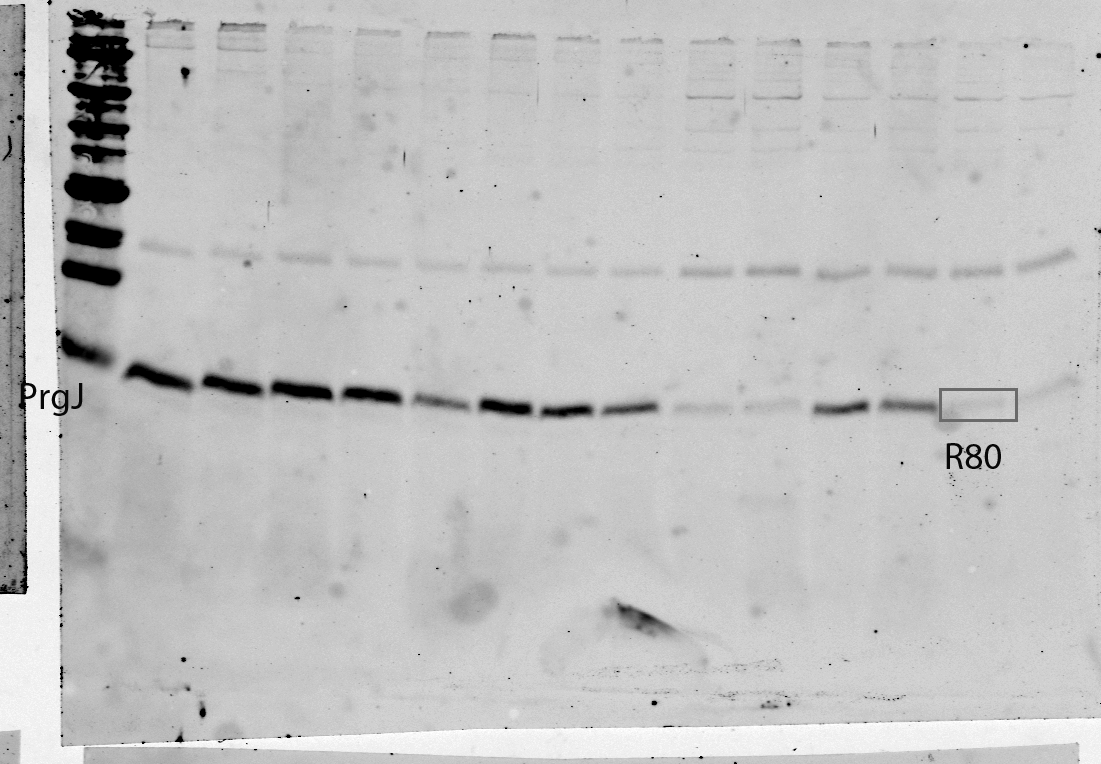

Supplement: S18 Data — (ZIP) [file pbio.3000351.s038.zip › S18-data/R80-anti-prgJ.tif]

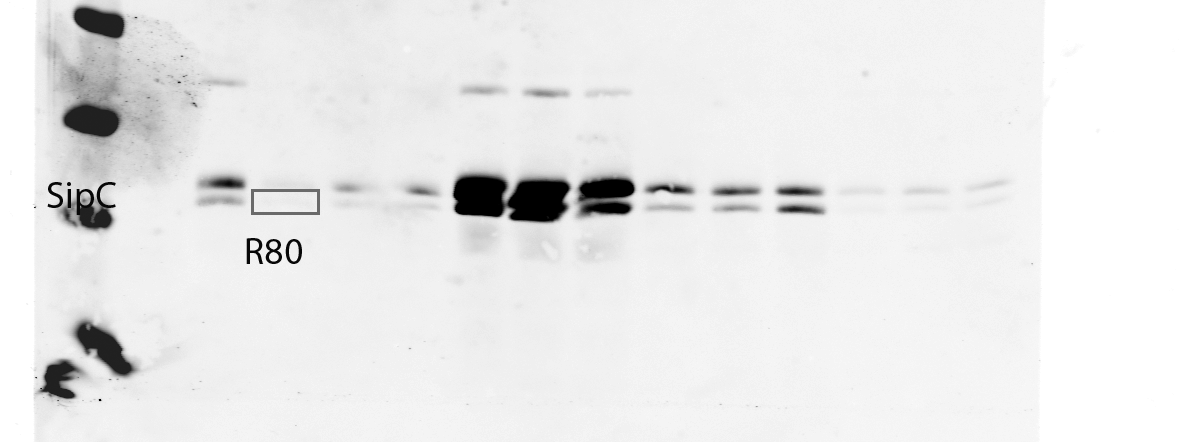

Supplement: S18 Data — (ZIP) [file pbio.3000351.s038.zip › S18-data/R80-anti-sipC.tif]

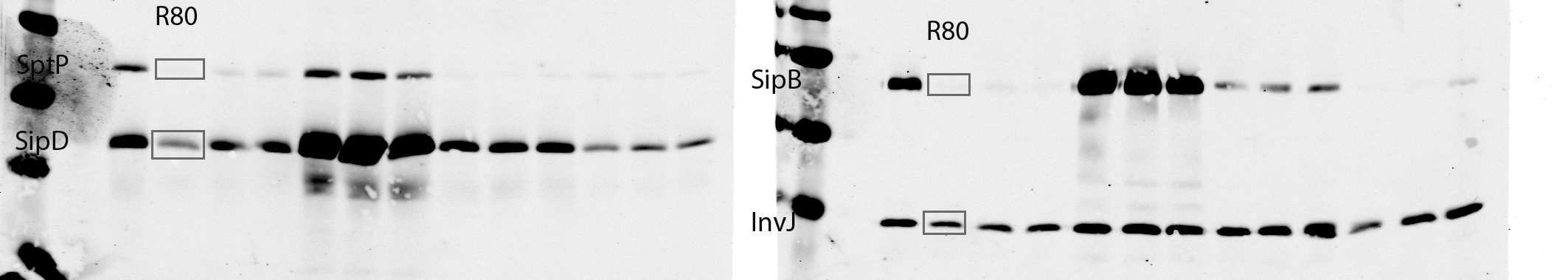

Supplement: S18 Data — (ZIP) [file pbio.3000351.s038.zip › S18-data/R80-anti-SptP-SipD-SipB-InvJ.tif]

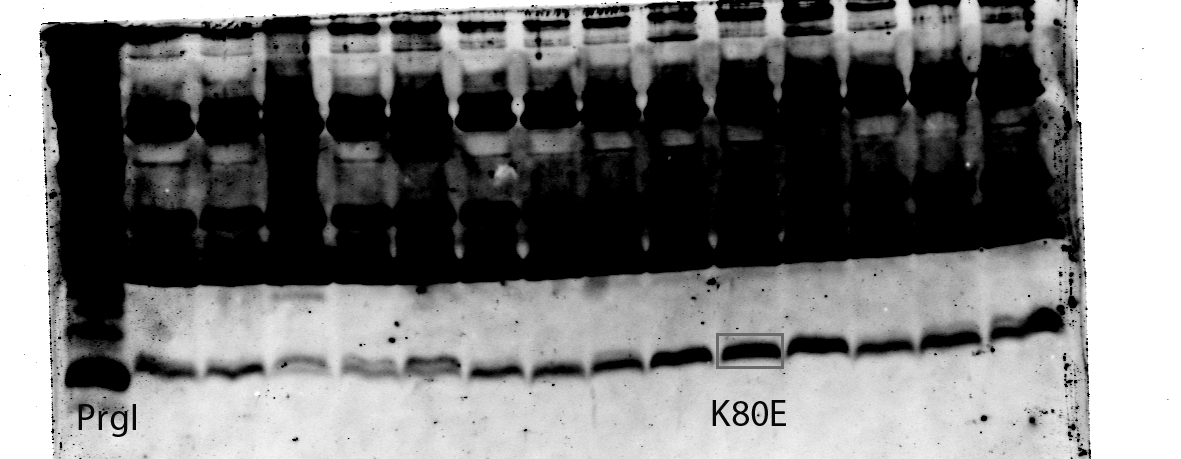

Supplement: S18 Data — (ZIP) [file pbio.3000351.s038.zip › S18-data/R80E-anti-prgI.tif]

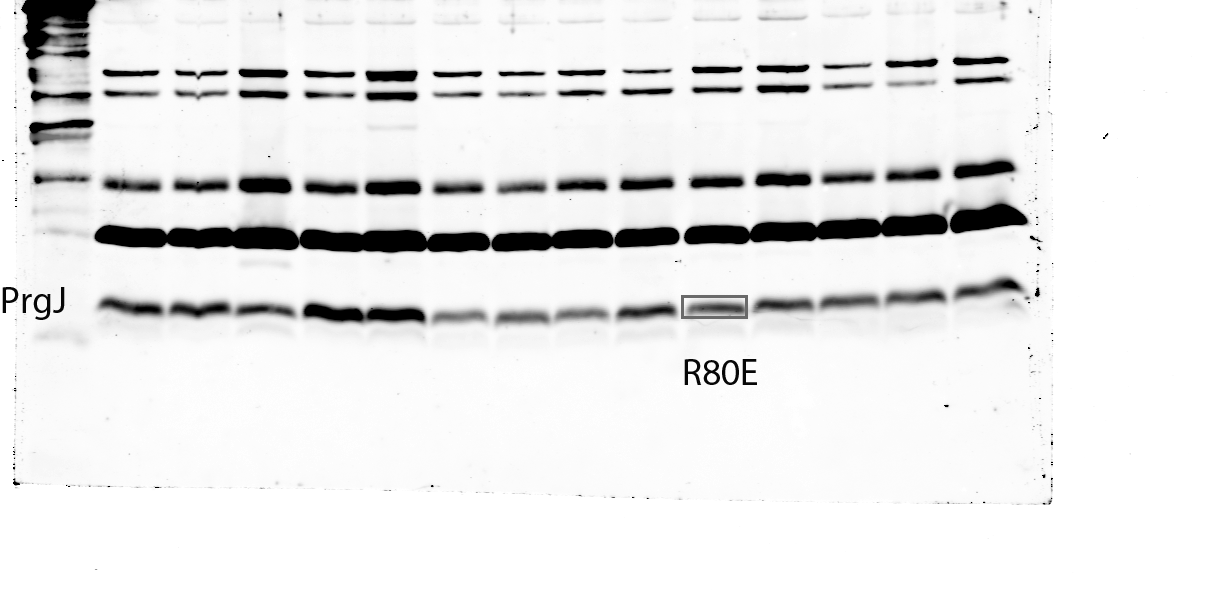

Supplement: S18 Data — (ZIP) [file pbio.3000351.s038.zip › S18-data/R80E-anti-prgJ.tif]

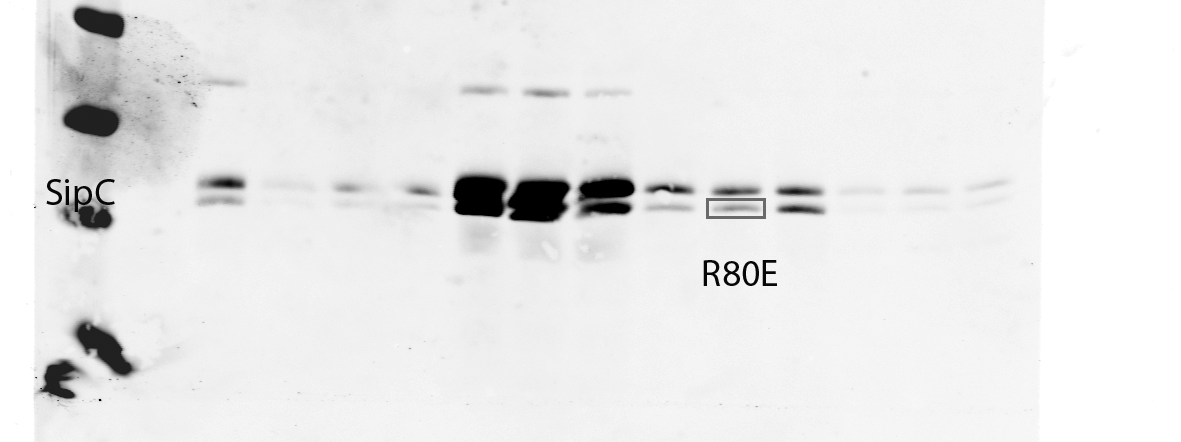

Supplement: S18 Data — (ZIP) [file pbio.3000351.s038.zip › S18-data/R80E-anti-sipC.tif]

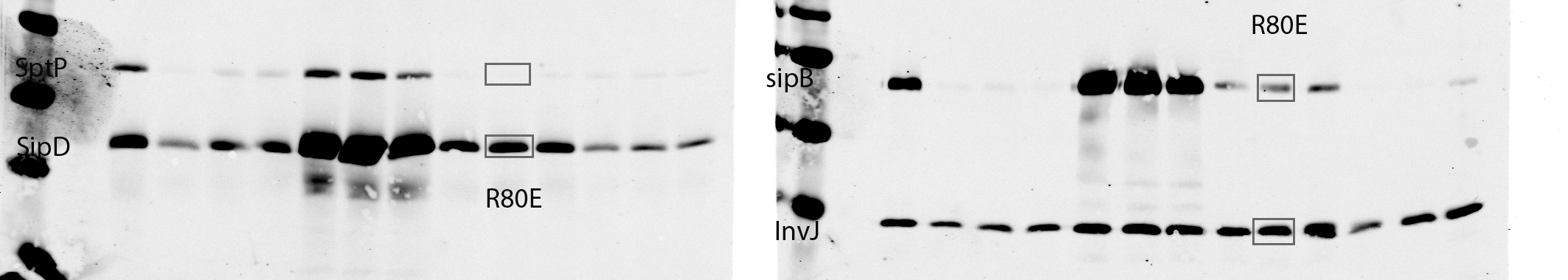

Supplement: S18 Data — (ZIP) [file pbio.3000351.s038.zip › S18-data/R80E-anti-SptP-SipD-SipB-InvJ.tif]

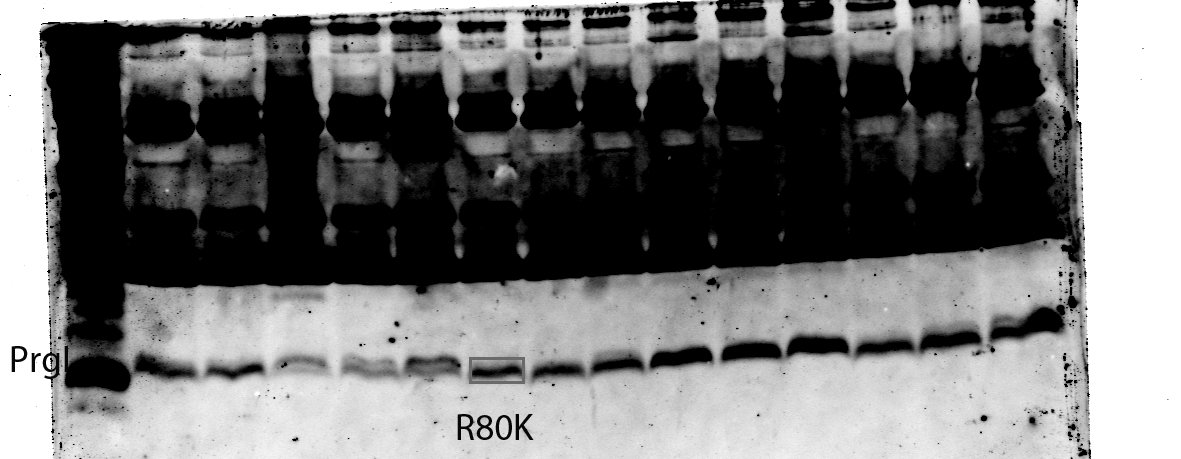

Supplement: S18 Data — (ZIP) [file pbio.3000351.s038.zip › S18-data/R80K-anti-prgI.tif]

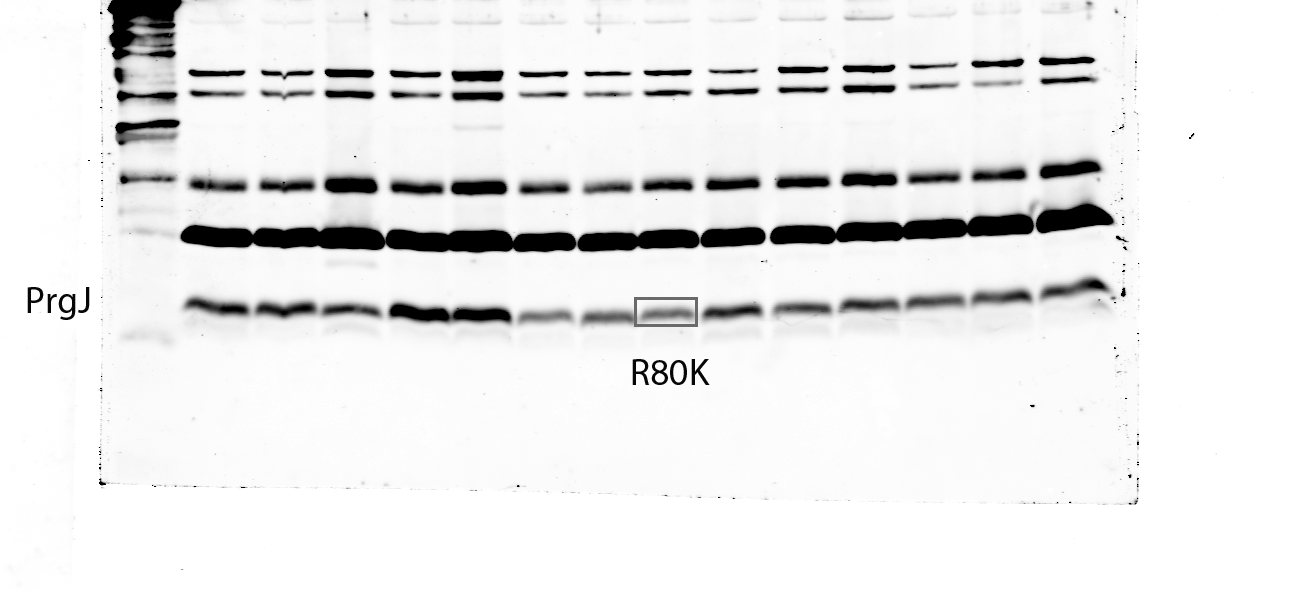

Supplement: S18 Data — (ZIP) [file pbio.3000351.s038.zip › S18-data/R80K-anti-prgJ.tif]

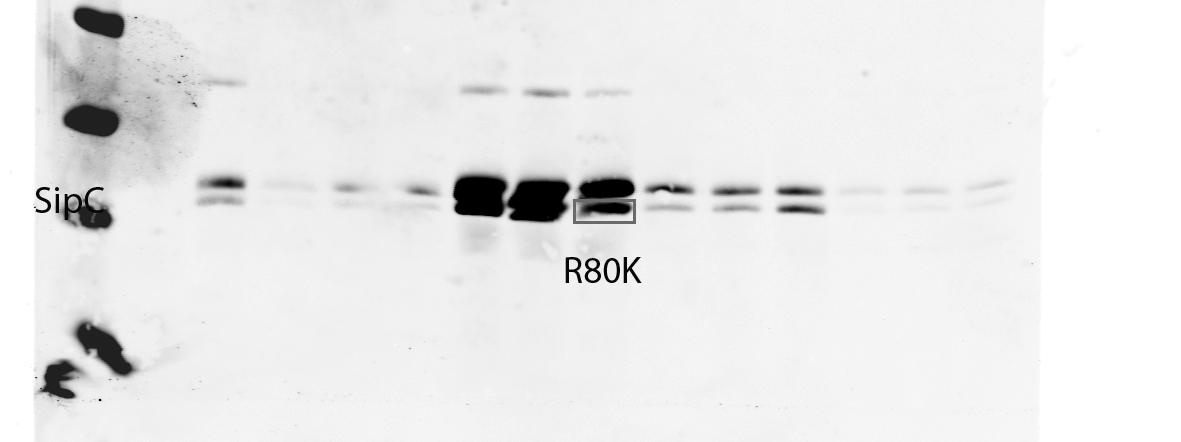

Supplement: S18 Data — (ZIP) [file pbio.3000351.s038.zip › S18-data/R80K-anti-sipC.tif]

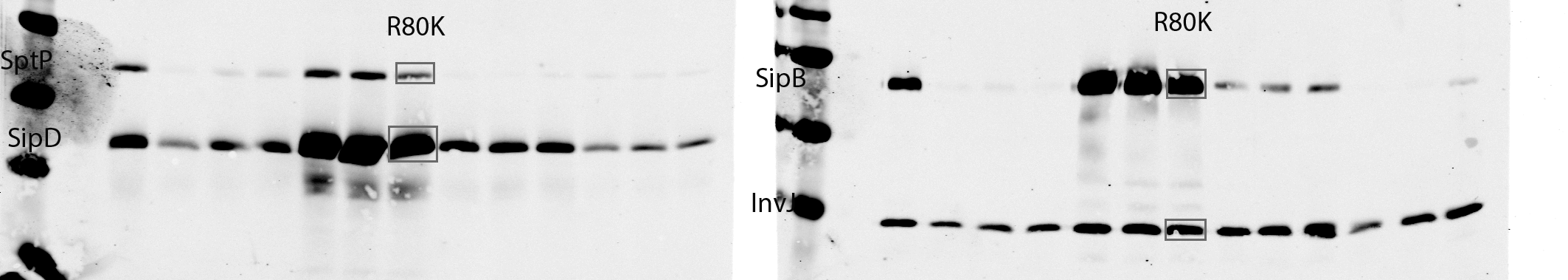

Supplement: S18 Data — (ZIP) [file pbio.3000351.s038.zip › S18-data/R80K-anti-SptP-SipD-SipB-InvJ.tif]

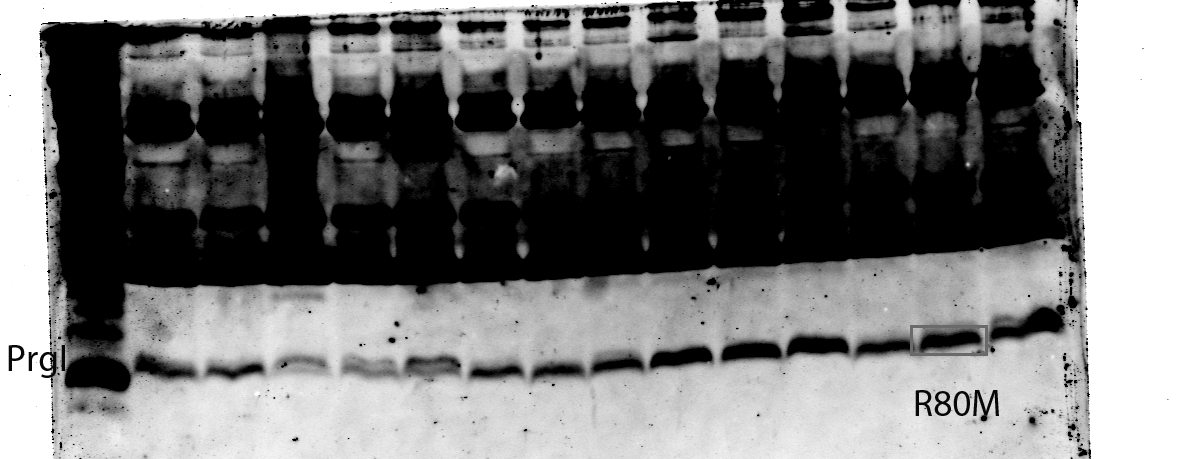

Supplement: S18 Data — (ZIP) [file pbio.3000351.s038.zip › S18-data/R80M-anti-prgI.tif]

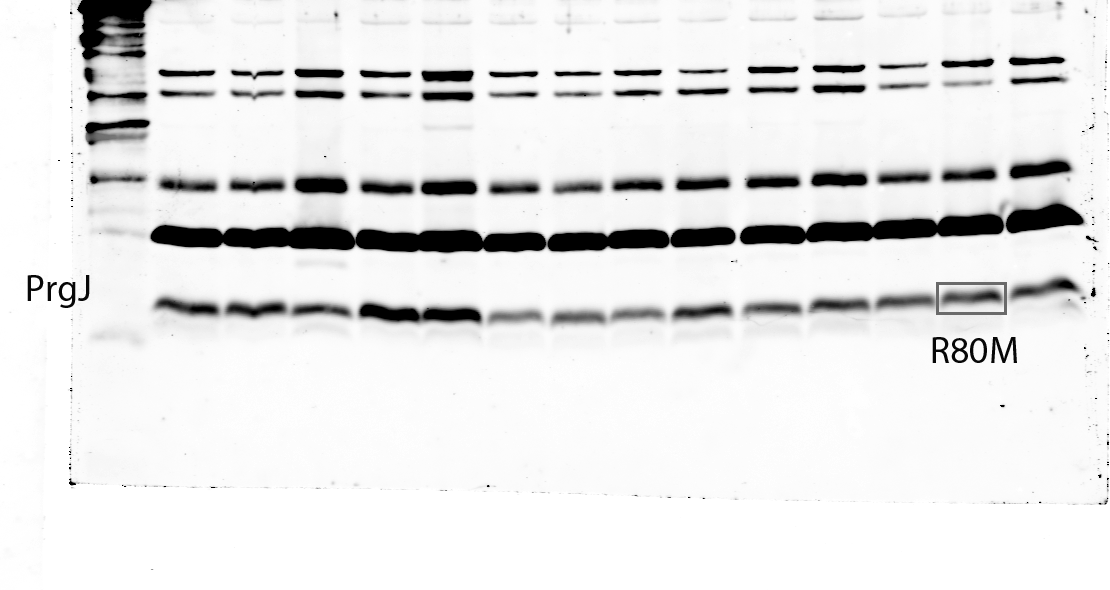

Supplement: S18 Data — (ZIP) [file pbio.3000351.s038.zip › S18-data/R80M-anti-prgJ.tif]

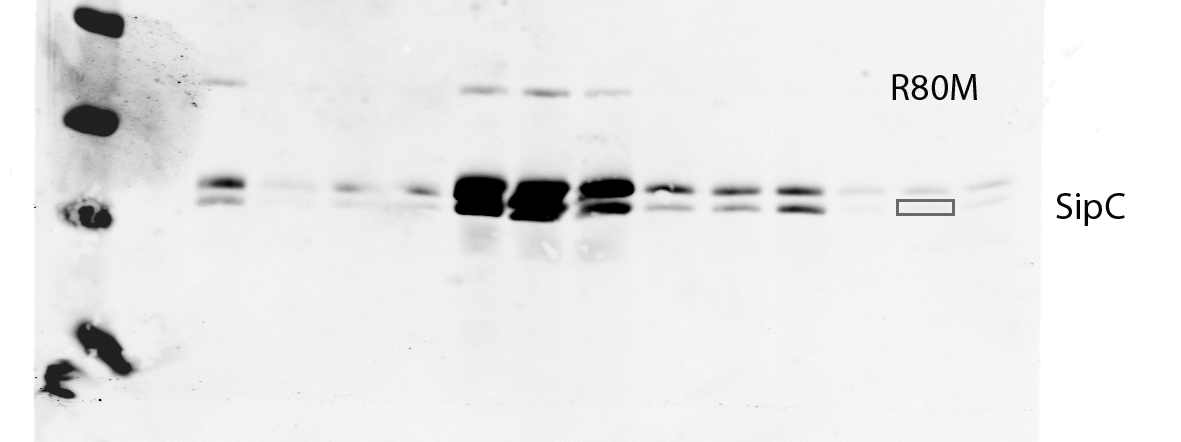

Supplement: S18 Data — (ZIP) [file pbio.3000351.s038.zip › S18-data/R80M-anti-sipC.tif]

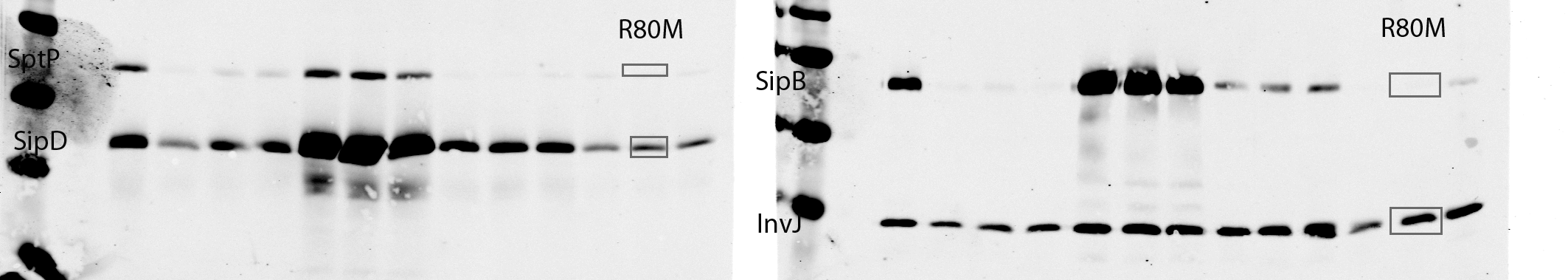

Supplement: S18 Data — (ZIP) [file pbio.3000351.s038.zip › S18-data/R80M-anti-SptP-SipD-SipB-InvJ.tif]

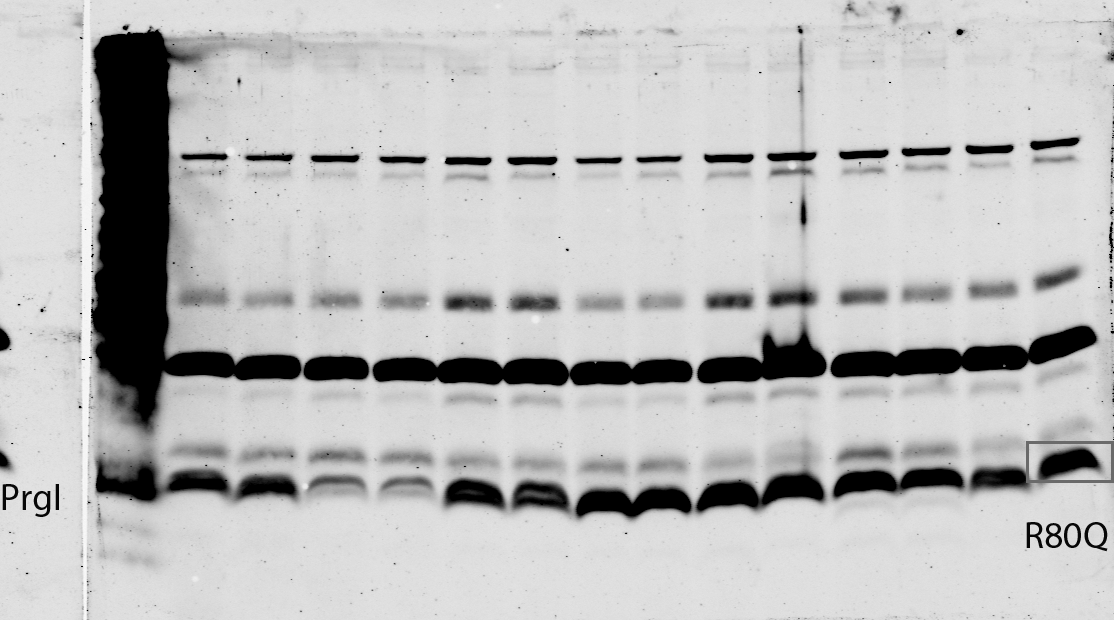

Supplement: S18 Data — (ZIP) [file pbio.3000351.s038.zip › S18-data/R80Q-anti-PrgI.tif]

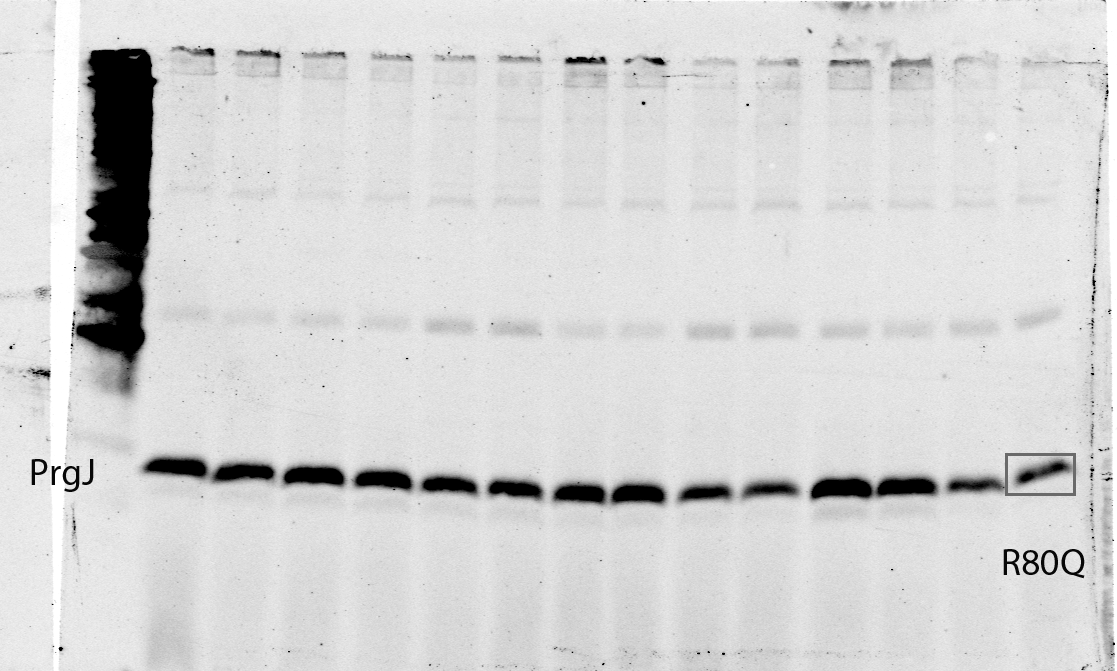

Supplement: S18 Data — (ZIP) [file pbio.3000351.s038.zip › S18-data/R80Q-anti-PrgJ.tif]

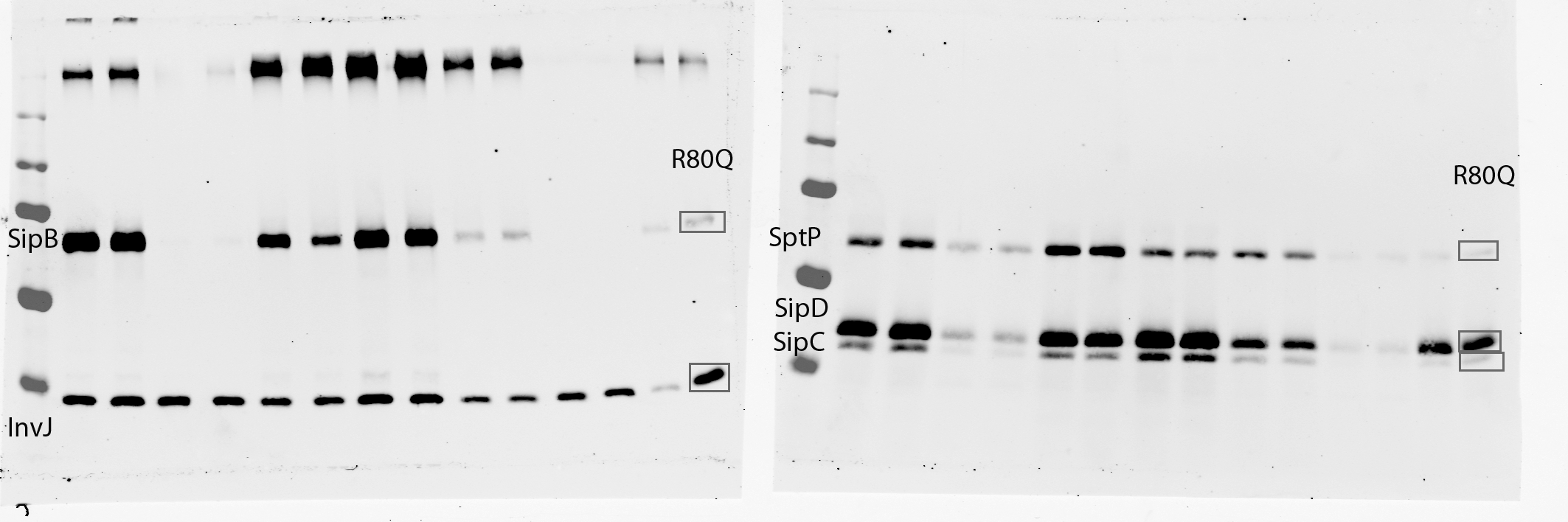

Supplement: S18 Data — (ZIP) [file pbio.3000351.s038.zip › S18-data/R80Q-anti-SipB-InvJ-SptP-SipD-SipC.tif]

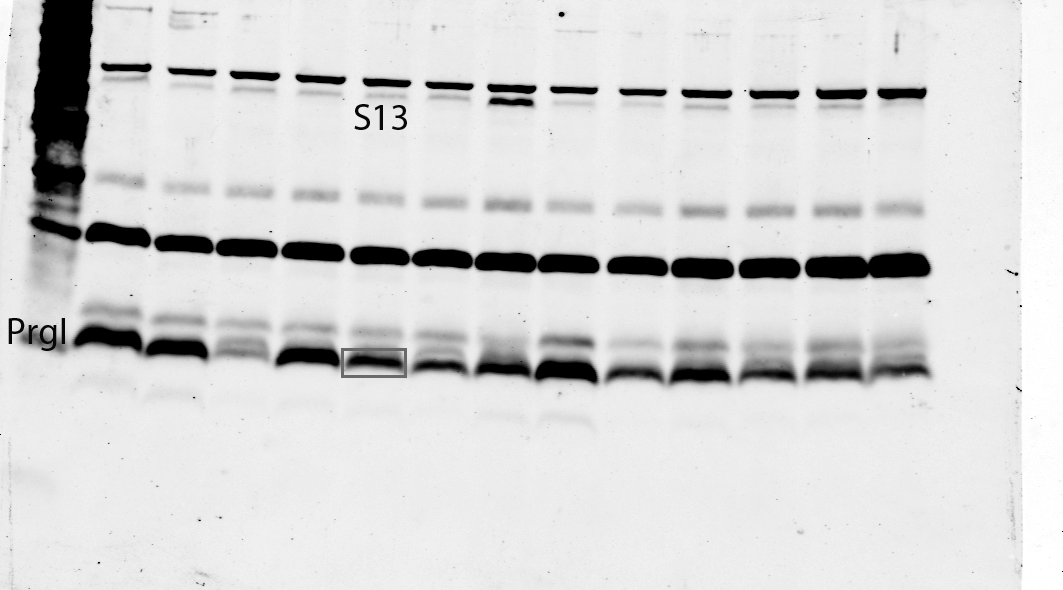

Supplement: S18 Data — (ZIP) [file pbio.3000351.s038.zip › S18-data/S13-anti-PrgI.tif]

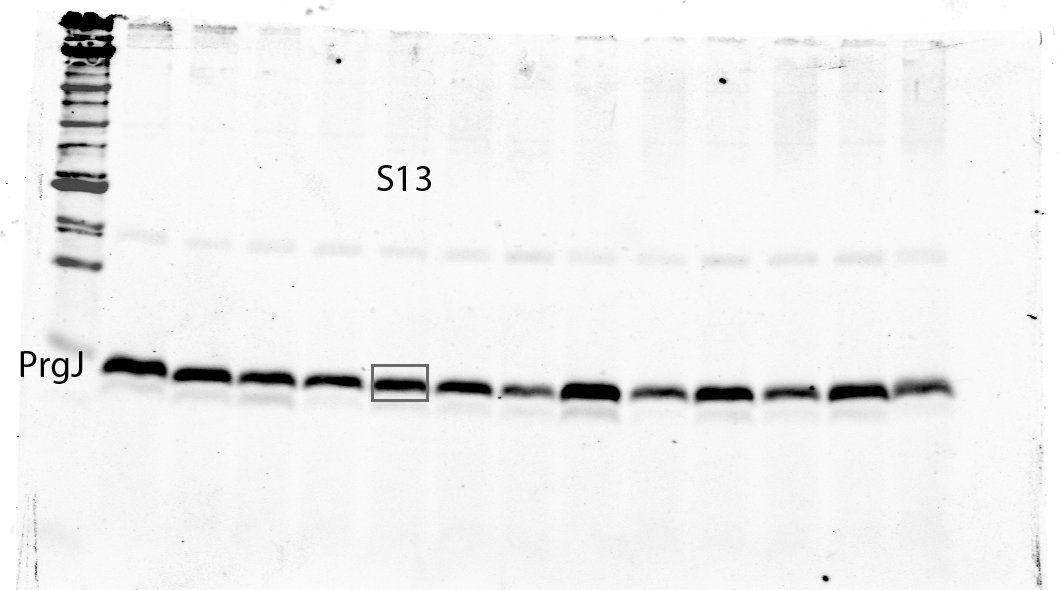

Supplement: S18 Data — (ZIP) [file pbio.3000351.s038.zip › S18-data/S13-anti-PrgJ.tif]

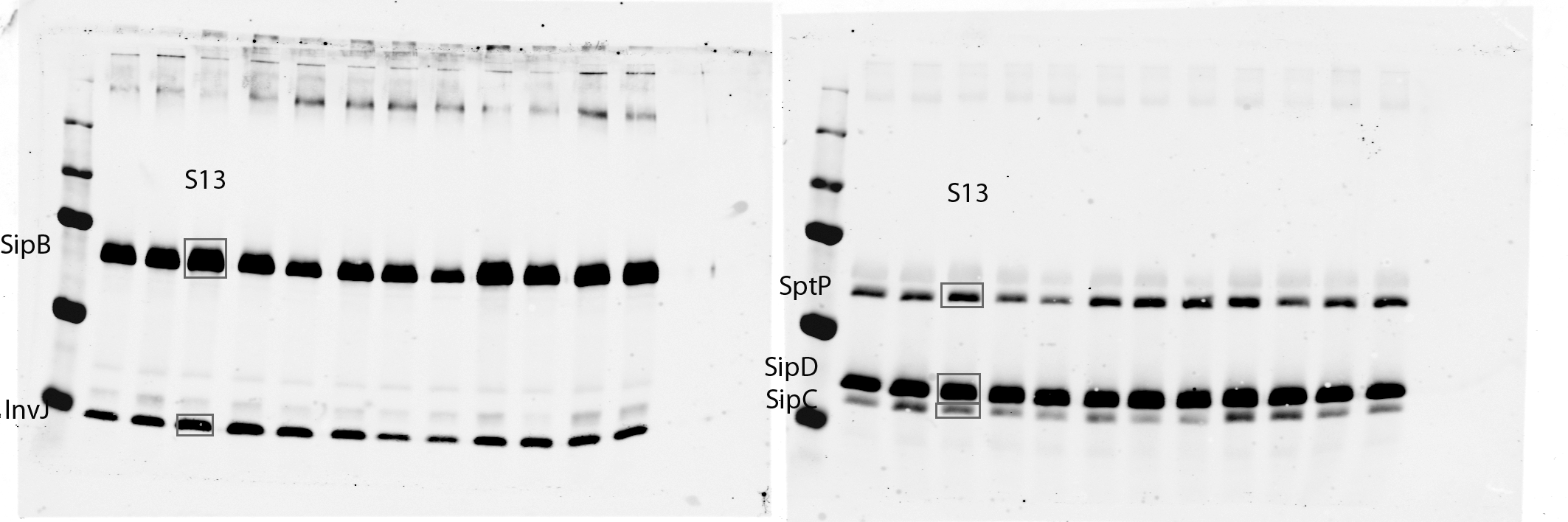

Supplement: S18 Data — (ZIP) [file pbio.3000351.s038.zip › S18-data/S13-anti-SipB-InvJ-SptP-SipD-SipC.tif]

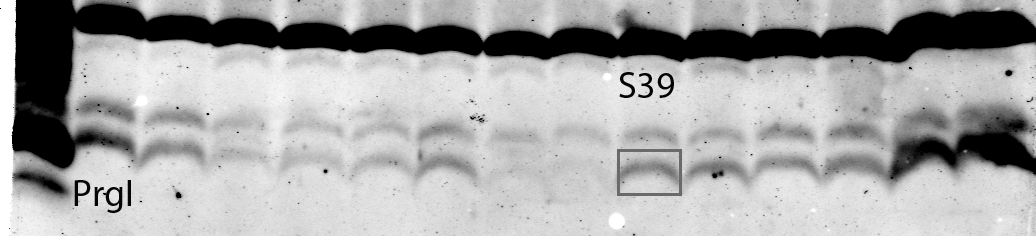

Supplement: S18 Data — (ZIP) [file pbio.3000351.s038.zip › S18-data/S39-anti-PrgI.tif]

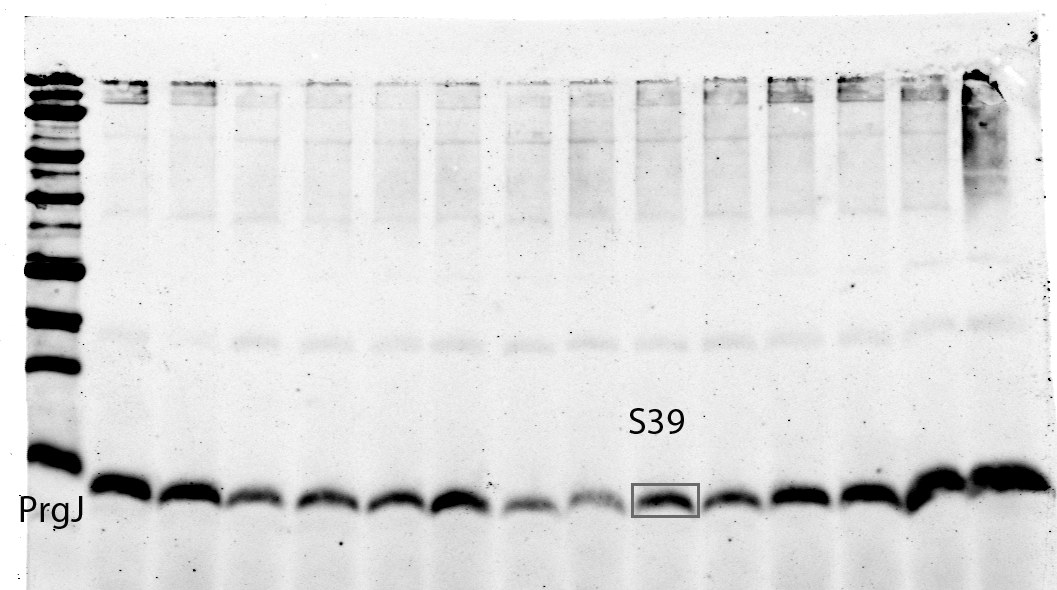

Supplement: S18 Data — (ZIP) [file pbio.3000351.s038.zip › S18-data/S39-anti-PrgJ.tif]

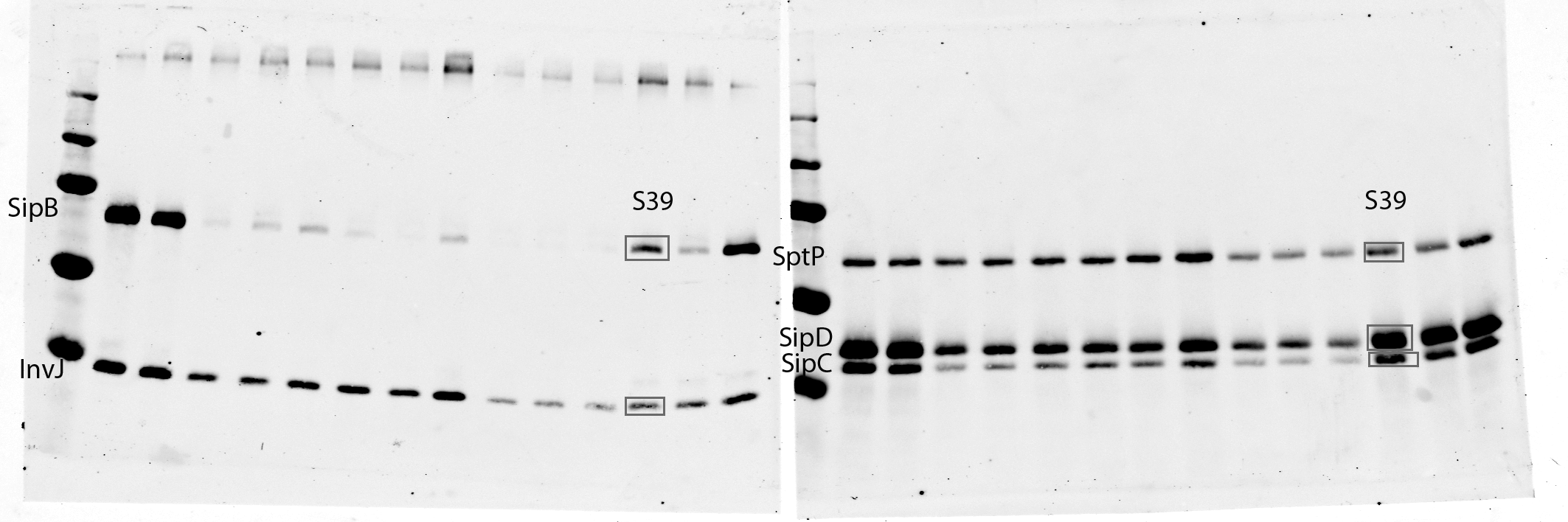

Supplement: S18 Data — (ZIP) [file pbio.3000351.s038.zip › S18-data/S39-anti-SipB-InvJ-SptP-SipD-SipC.tif]

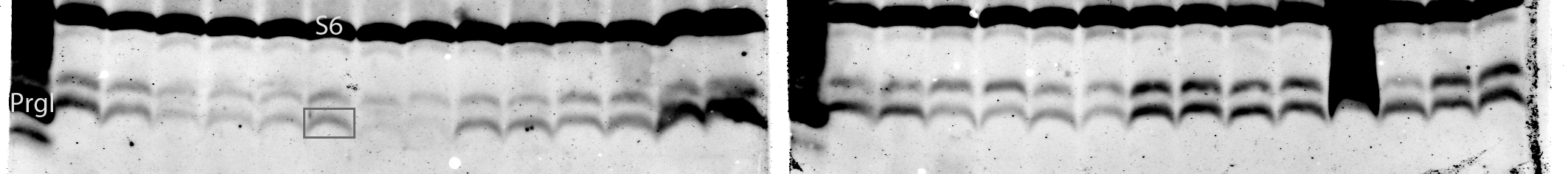

Supplement: S18 Data — (ZIP) [file pbio.3000351.s038.zip › S18-data/S6-anti-PrgI.tif]

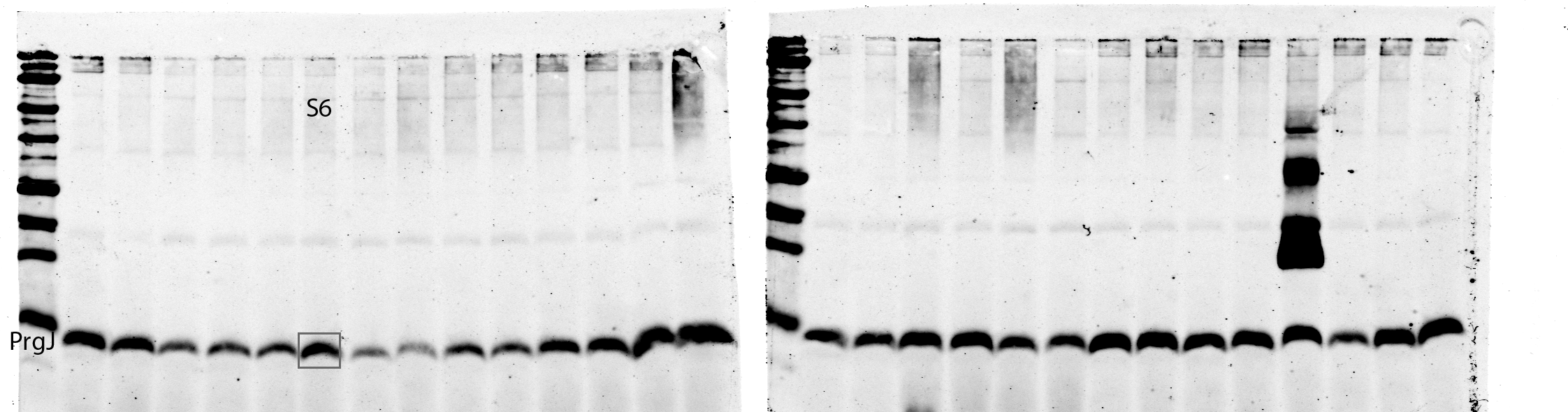

Supplement: S18 Data — (ZIP) [file pbio.3000351.s038.zip › S18-data/S6-anti-PrgJ.tif]

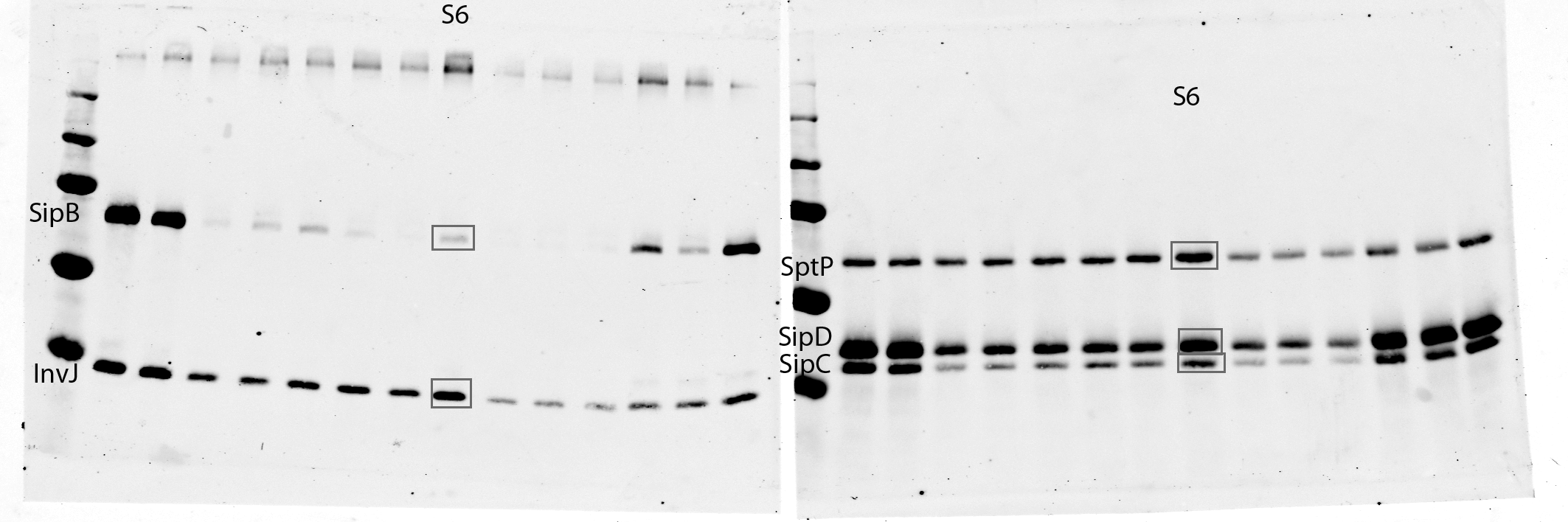

Supplement: S18 Data — (ZIP) [file pbio.3000351.s038.zip › S18-data/S6-anti-SipB-InvJ-SptP-SipD-SipC.tif]

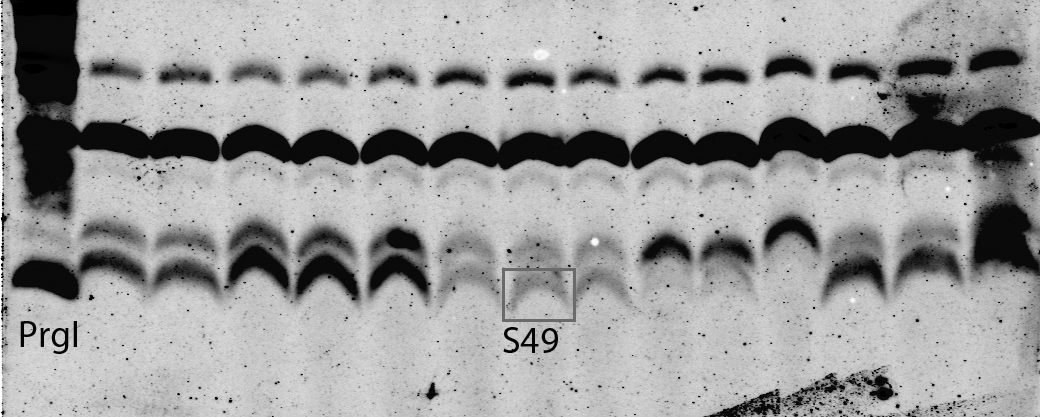

Supplement: S19 Data — (ZIP) [file pbio.3000351.s039.zip › S19-data/S49-anti-prgI.tif]

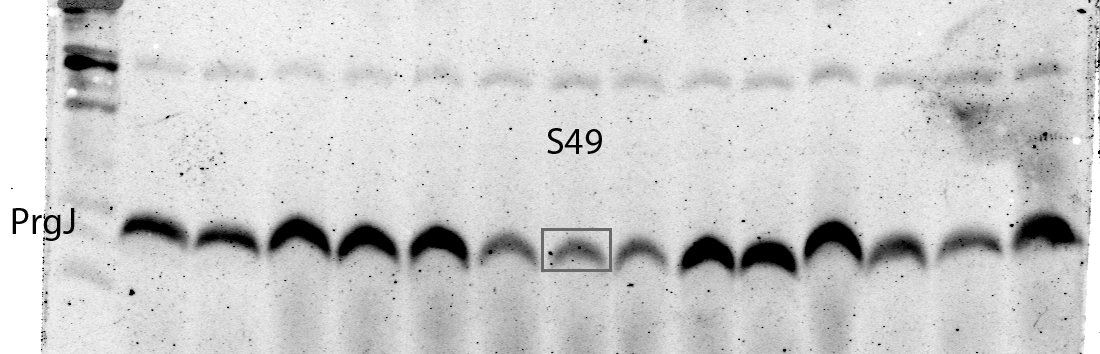

Supplement: S19 Data — (ZIP) [file pbio.3000351.s039.zip › S19-data/S49-anti-prgJ.tif]

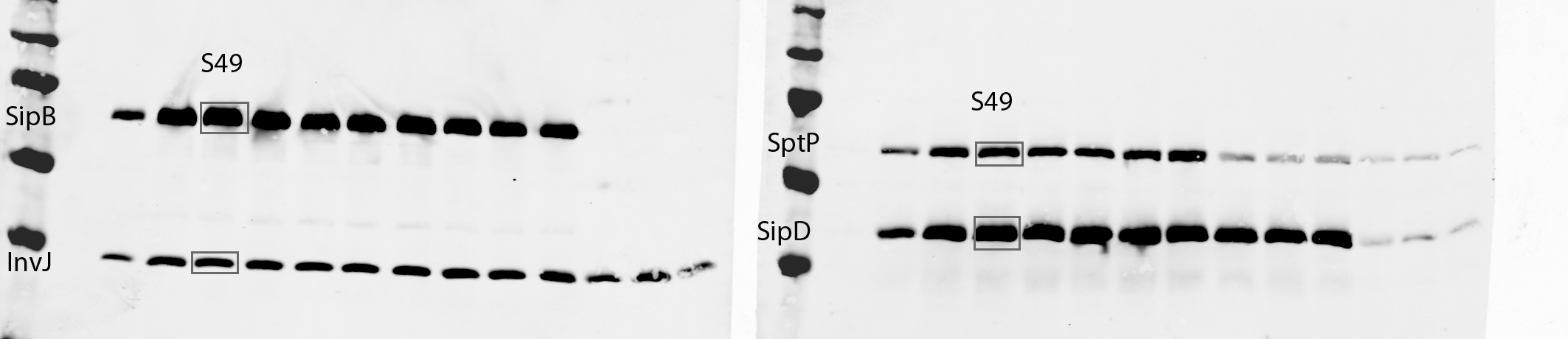

Supplement: S19 Data — (ZIP) [file pbio.3000351.s039.zip › S19-data/S49-anti-SipB-InvJ-SptP-SipD.tif]

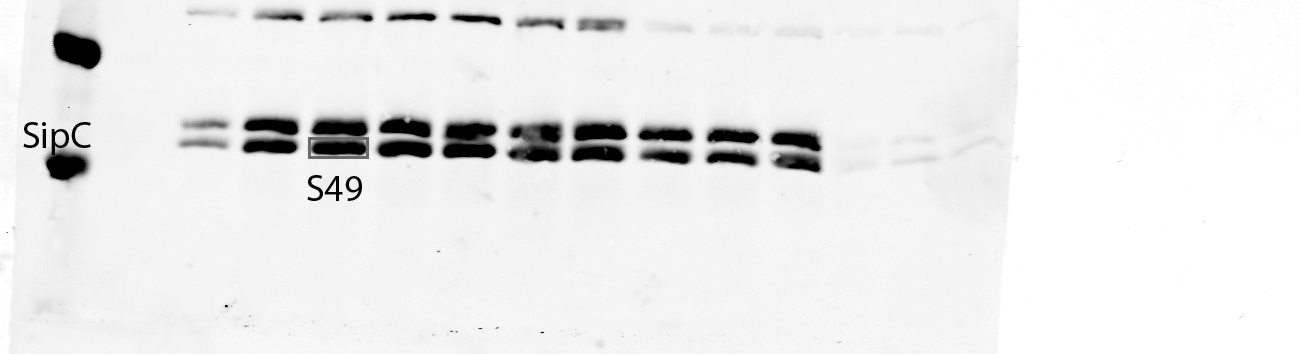

Supplement: S19 Data — (ZIP) [file pbio.3000351.s039.zip › S19-data/S49-anti-sipC.tif]

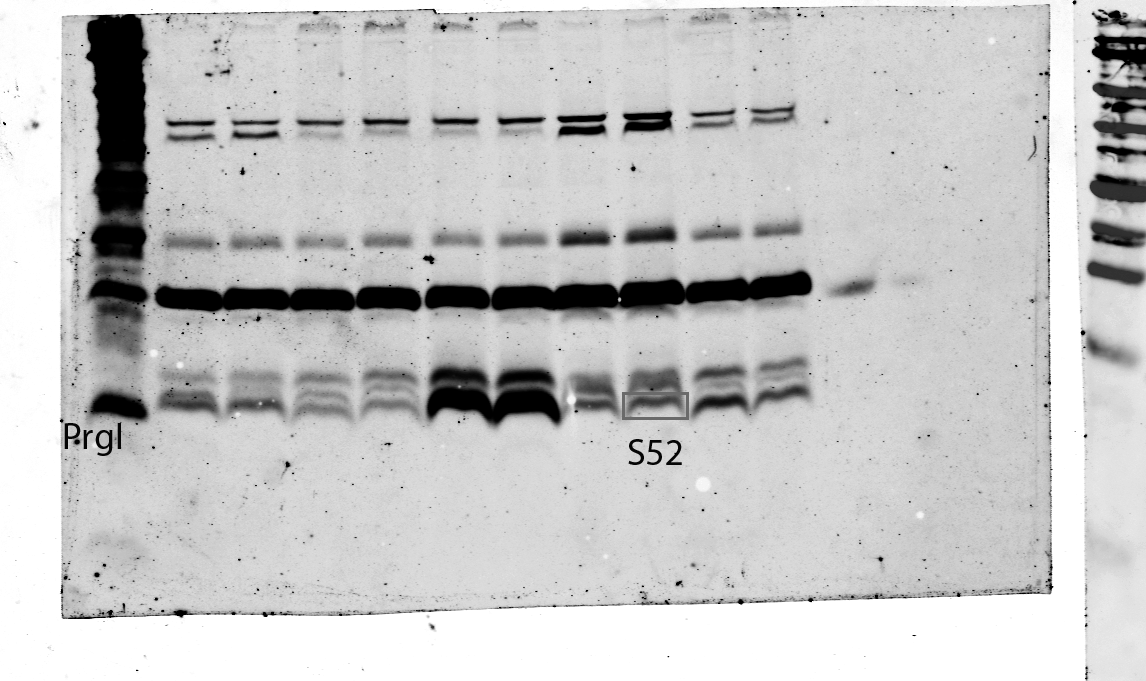

Supplement: S19 Data — (ZIP) [file pbio.3000351.s039.zip › S19-data/S52-anti-prgI.tif]

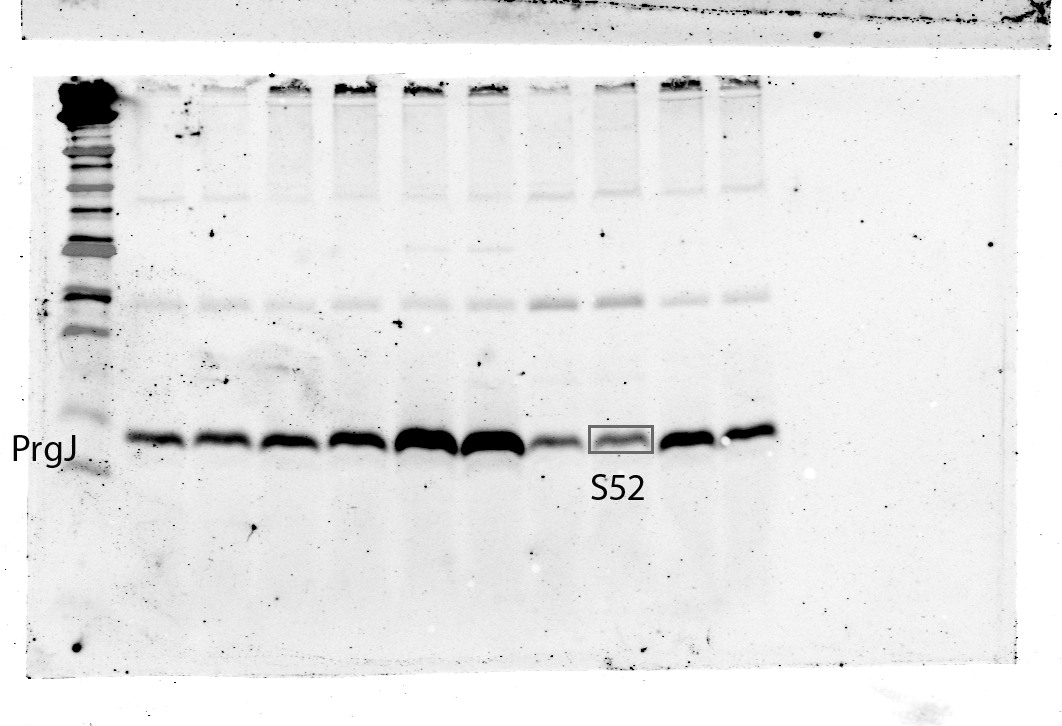

Supplement: S19 Data — (ZIP) [file pbio.3000351.s039.zip › S19-data/S52-anti-prgJ.tif]

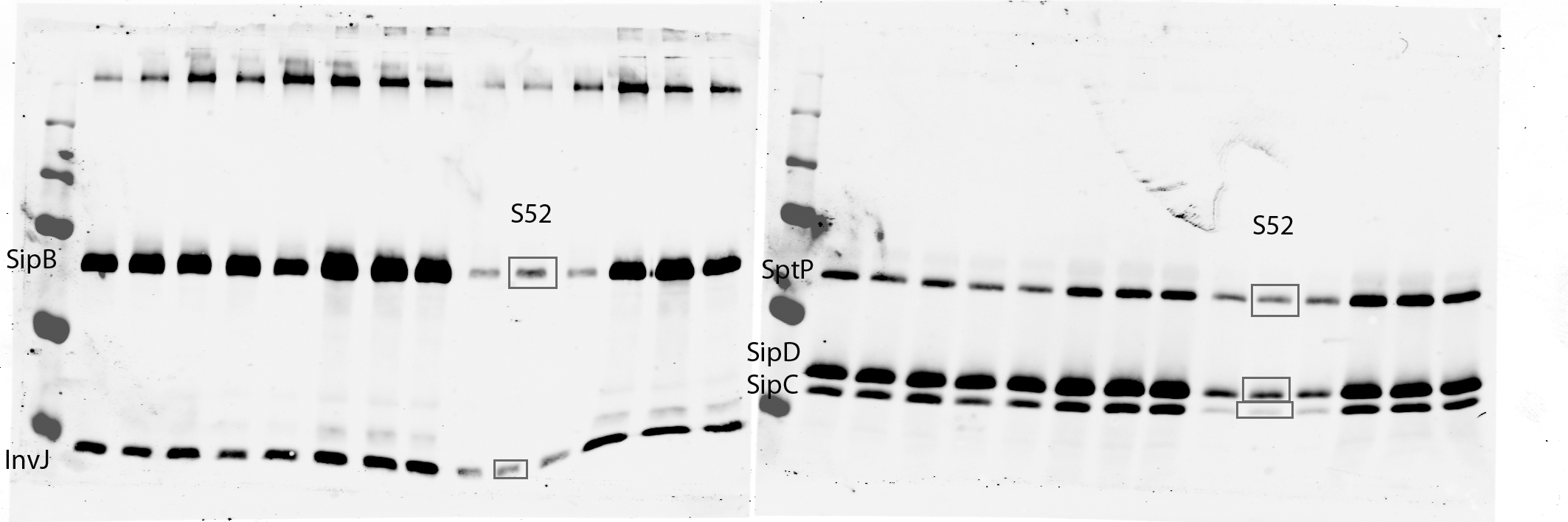

Supplement: S19 Data — (ZIP) [file pbio.3000351.s039.zip › S19-data/S52-anti-SipB-InvJ-SptP-SipD-SipC.tif]

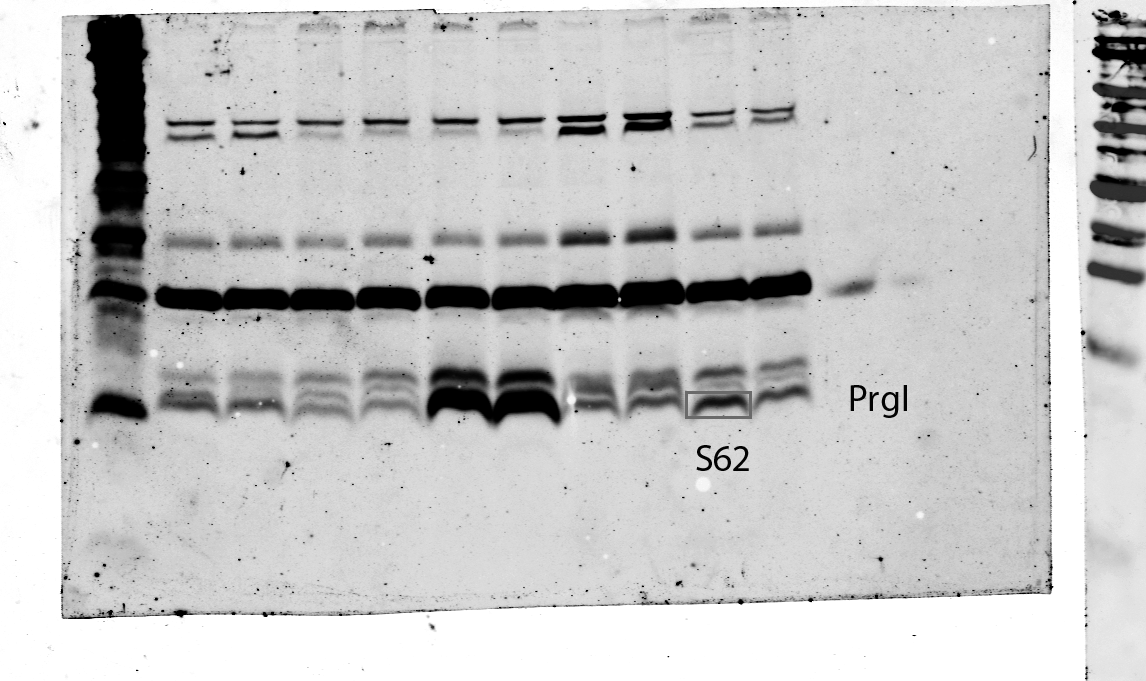

Supplement: S19 Data — (ZIP) [file pbio.3000351.s039.zip › S19-data/S62-anti-prgI.tif]

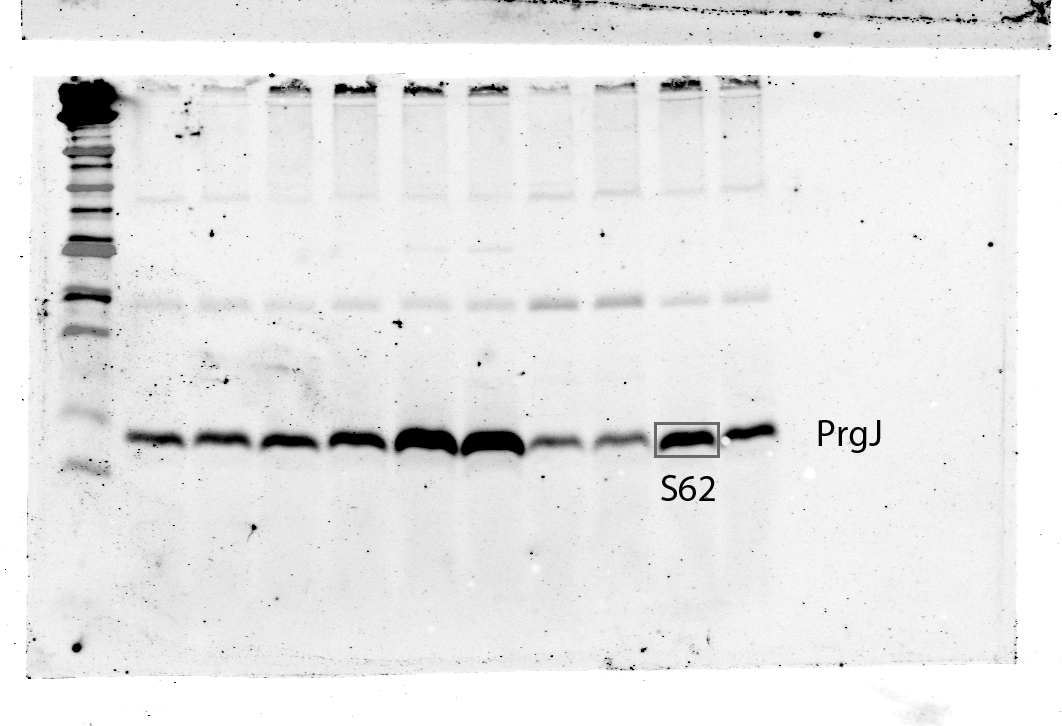

Supplement: S19 Data — (ZIP) [file pbio.3000351.s039.zip › S19-data/S62-anti-prgJ.tif]

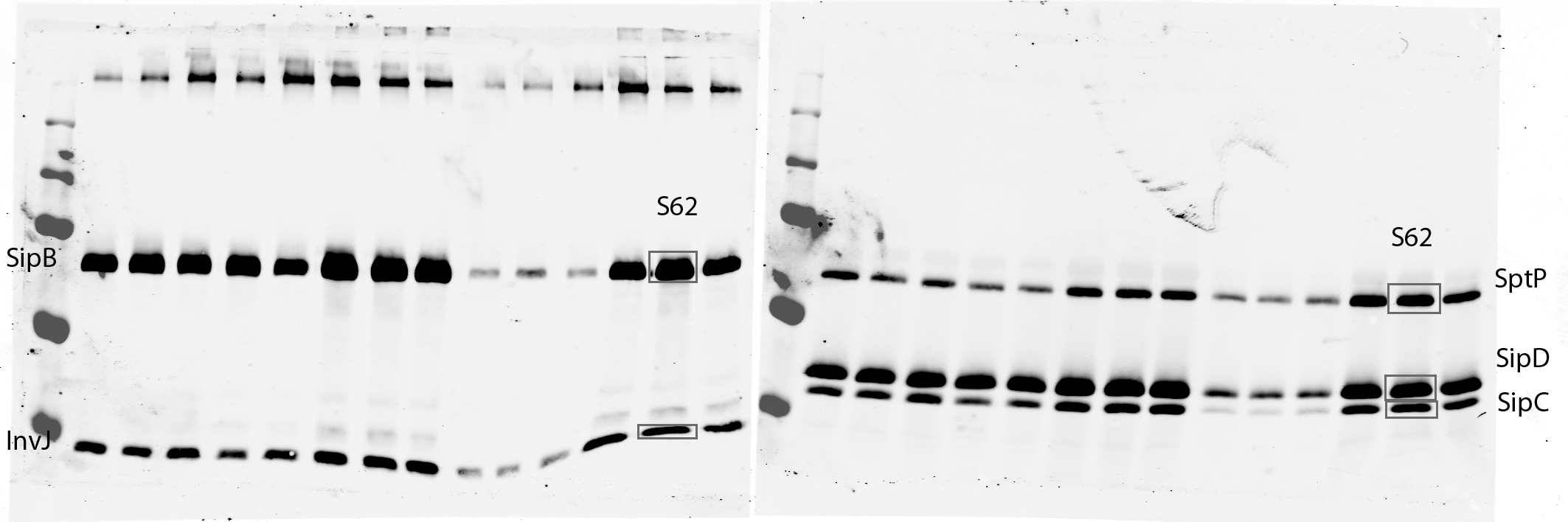

Supplement: S19 Data — (ZIP) [file pbio.3000351.s039.zip › S19-data/S62-anti-SipB-InvJ-SptP-SipD-SipC.tif]

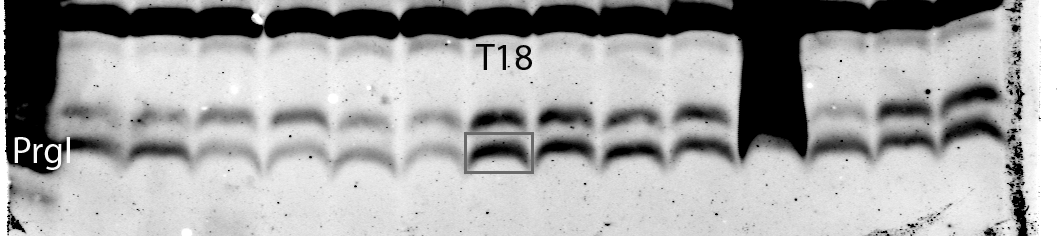

Supplement: S19 Data — (ZIP) [file pbio.3000351.s039.zip › S19-data/T18-anti-PrgI.tif]

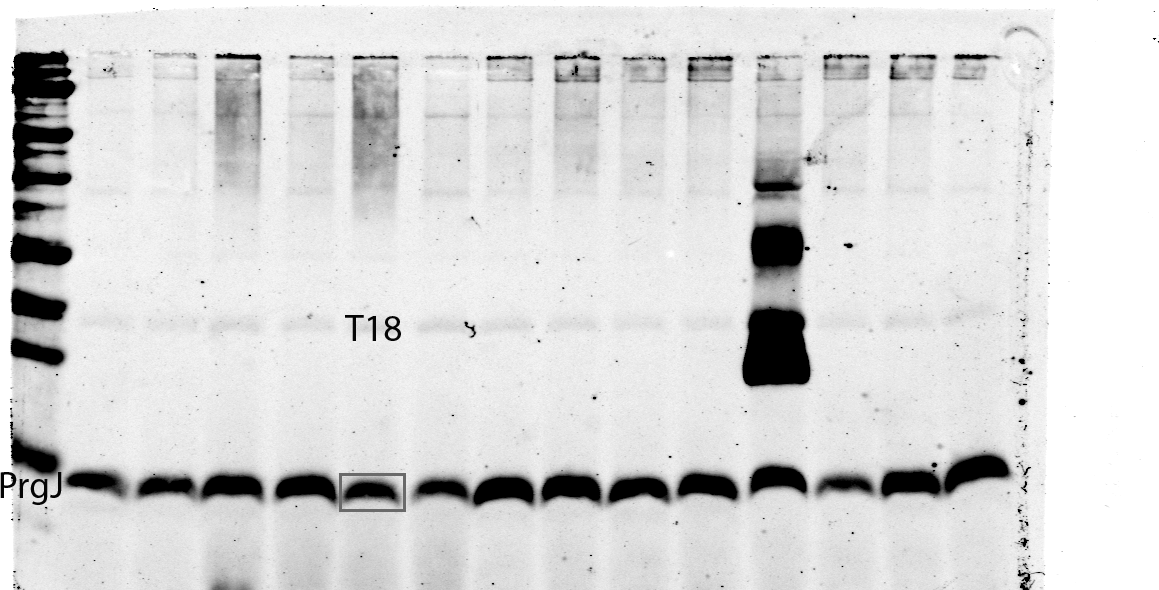

Supplement: S19 Data — (ZIP) [file pbio.3000351.s039.zip › S19-data/T18-anti-PrgJ.tif]

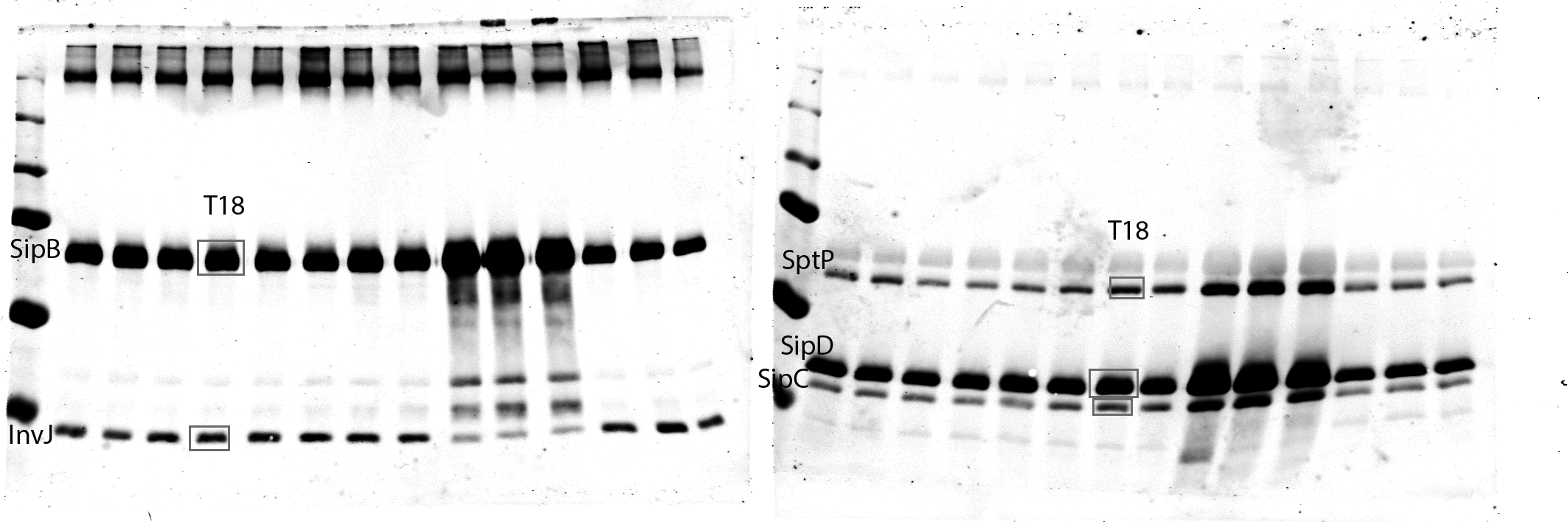

Supplement: S19 Data — (ZIP) [file pbio.3000351.s039.zip › S19-data/T18-anti-SipB-InvJ-SptP-SipD-SipC.tif]

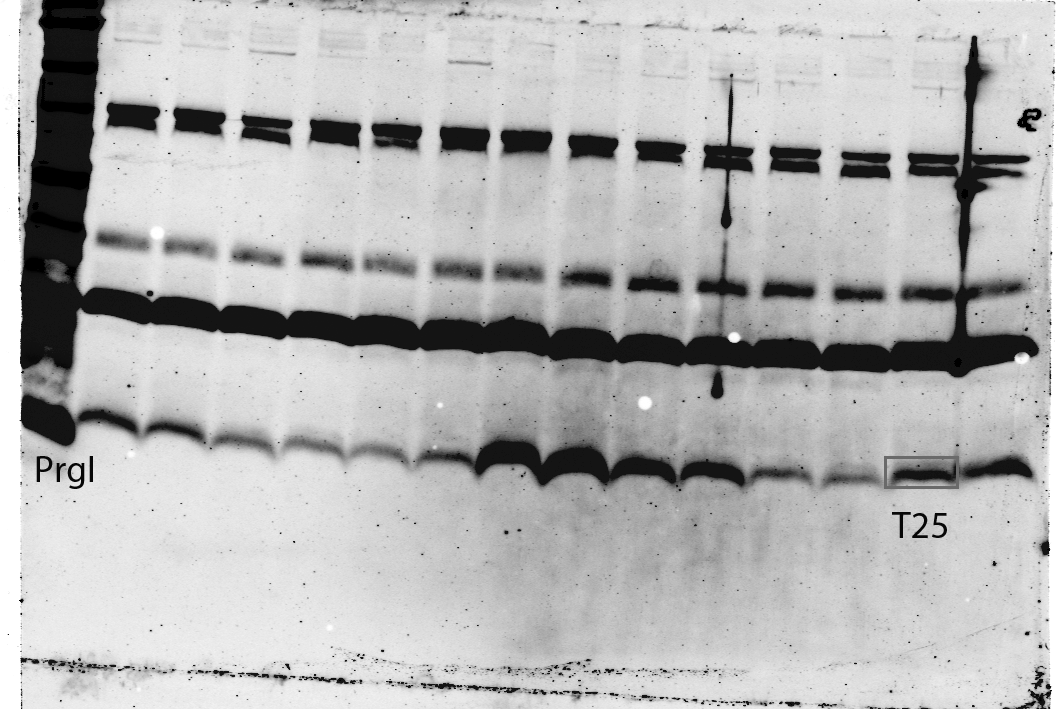

Supplement: S19 Data — (ZIP) [file pbio.3000351.s039.zip › S19-data/T25-anti-prgI.tif]

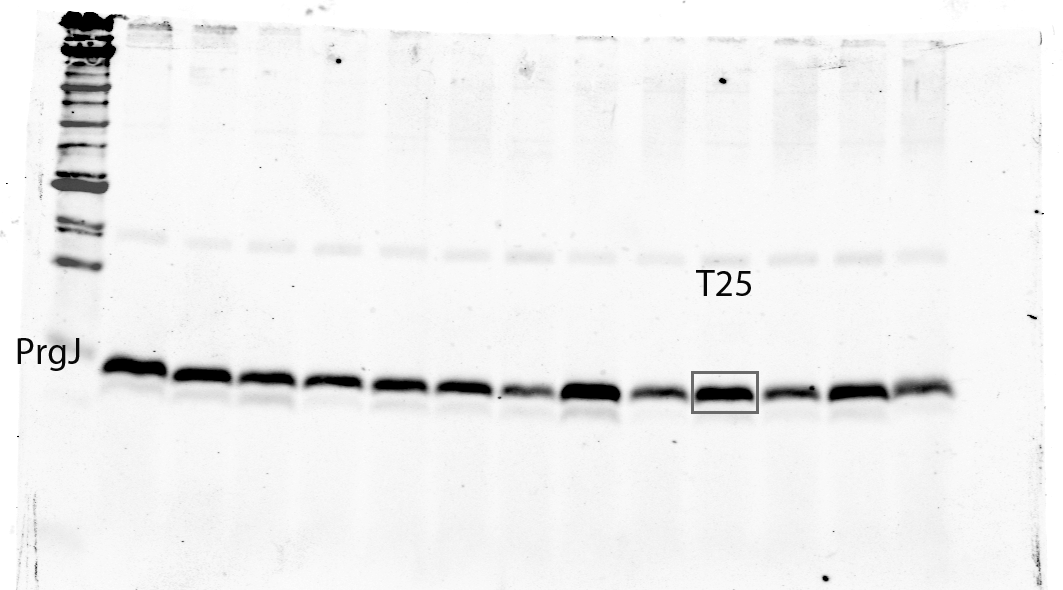

Supplement: S19 Data — (ZIP) [file pbio.3000351.s039.zip › S19-data/T25-anti-PrgJ.tif]

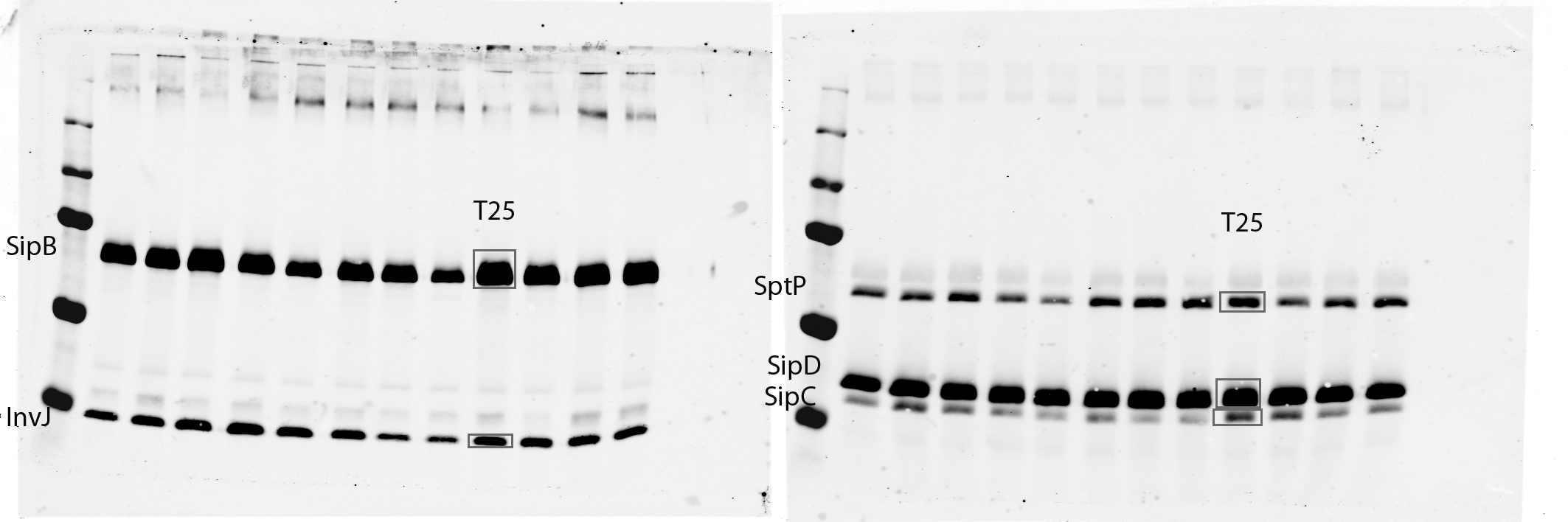

Supplement: S19 Data — (ZIP) [file pbio.3000351.s039.zip › S19-data/T25-anti-SipB-InvJ-SptP-SipD-SipC.tif]

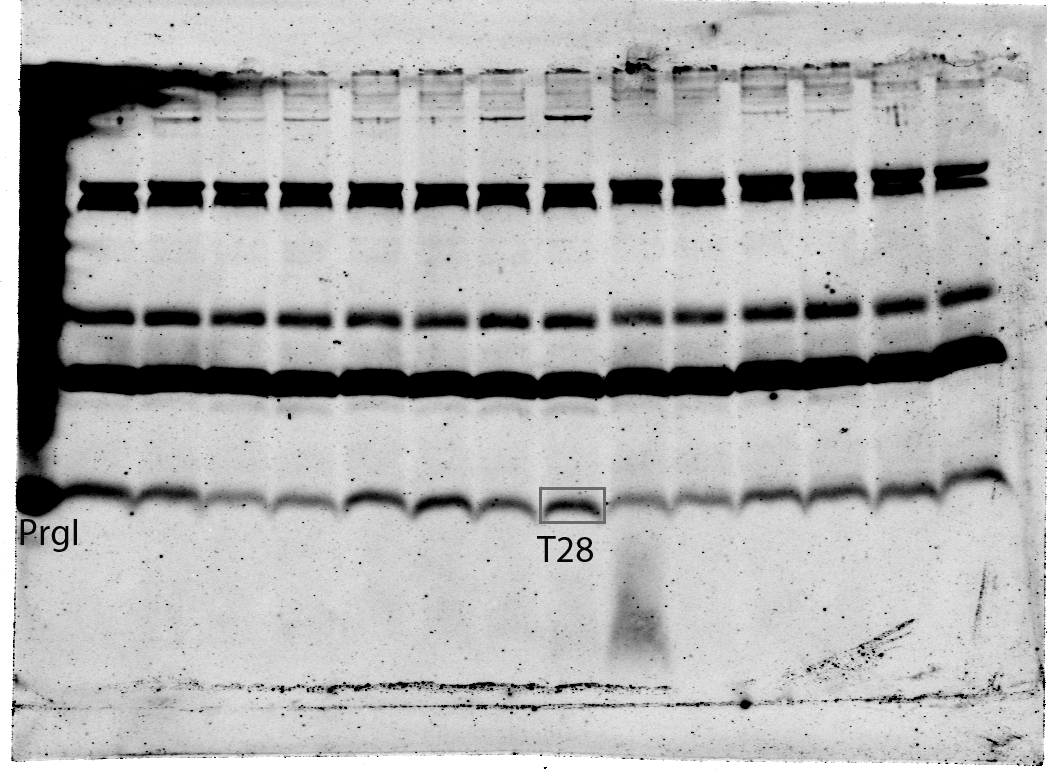

Supplement: S19 Data — (ZIP) [file pbio.3000351.s039.zip › S19-data/T28-anti-prgI.tif]

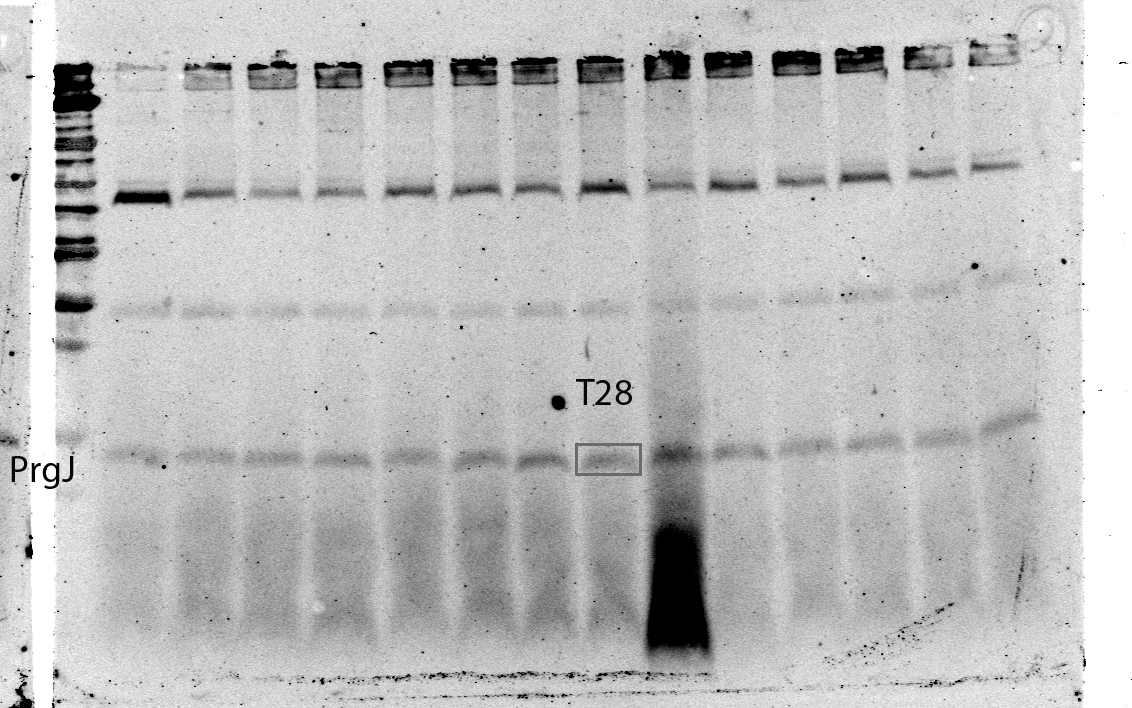

Supplement: S19 Data — (ZIP) [file pbio.3000351.s039.zip › S19-data/T28-anti-prgJ.tif]

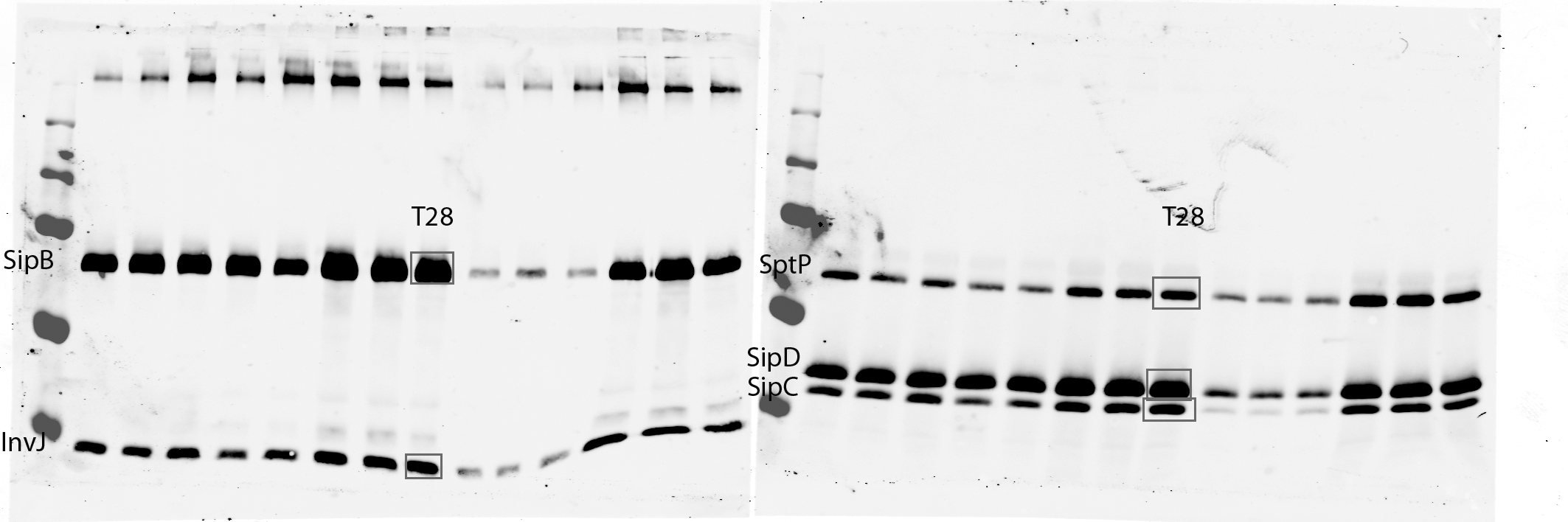

Supplement: S19 Data — (ZIP) [file pbio.3000351.s039.zip › S19-data/T28-anti-SipB-InvJ-SptP-SipD-SipC.tif]

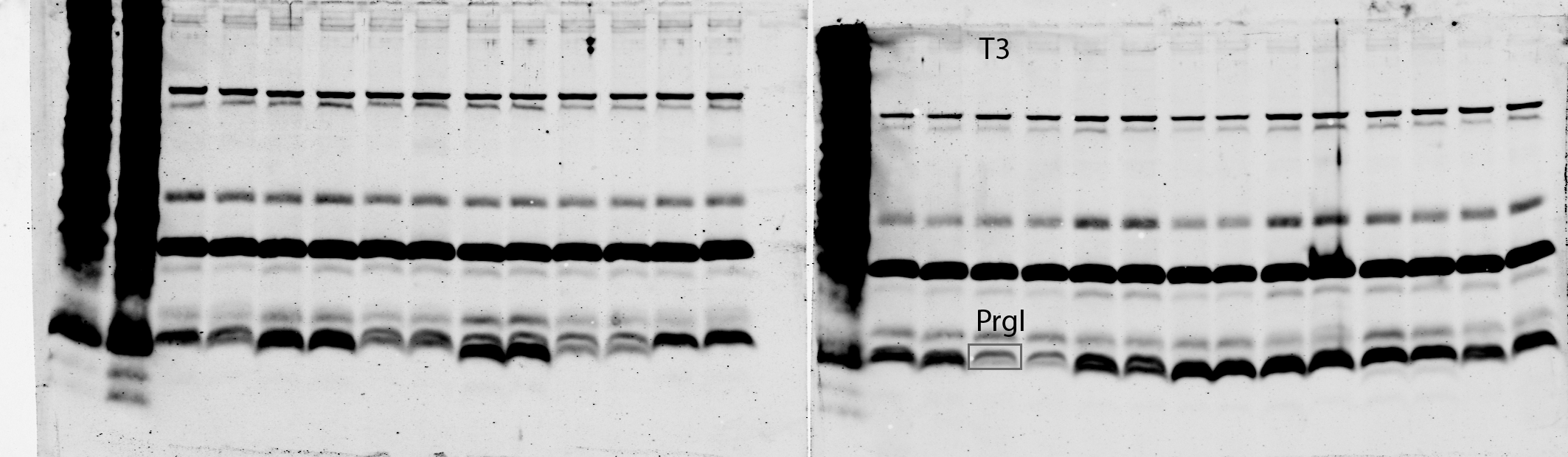

Supplement: S19 Data — (ZIP) [file pbio.3000351.s039.zip › S19-data/T3-anti-PrgI.tif]

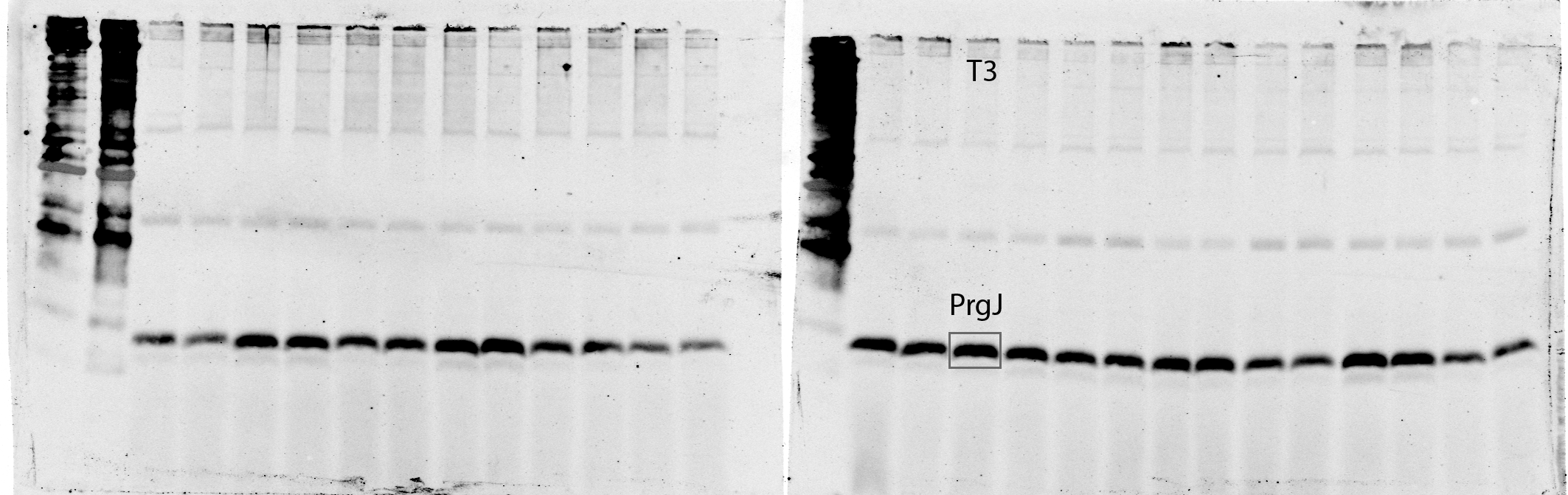

Supplement: S19 Data — (ZIP) [file pbio.3000351.s039.zip › S19-data/T3-anti-PrgJ.tif]

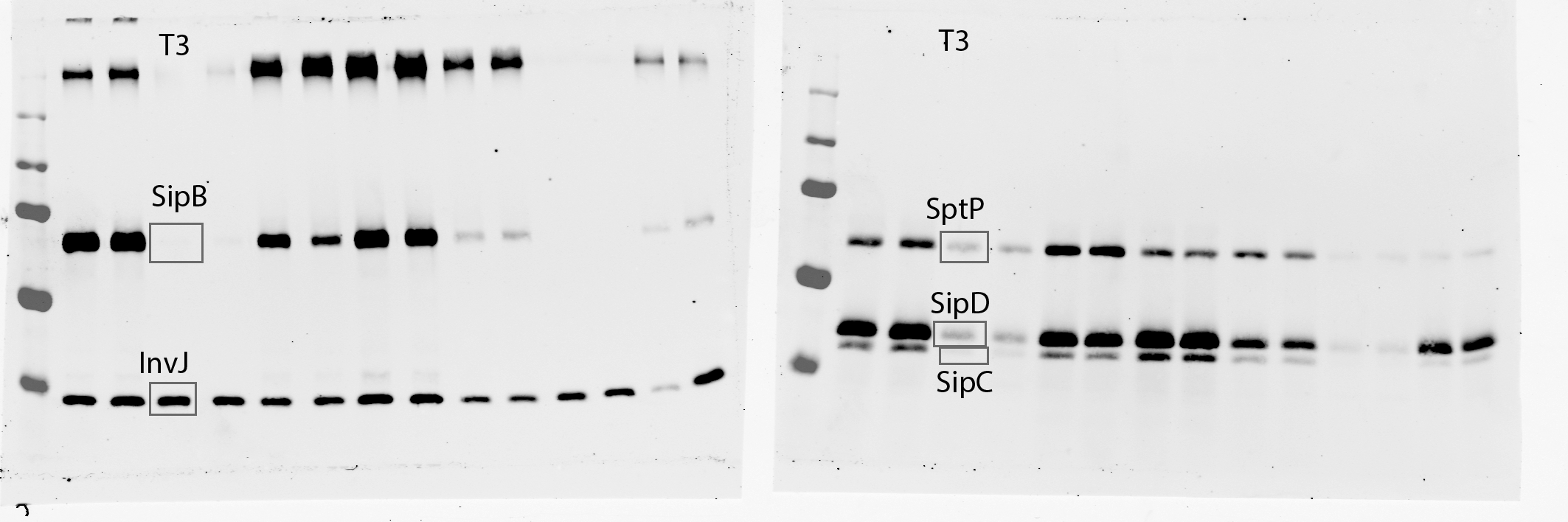

Supplement: S19 Data — (ZIP) [file pbio.3000351.s039.zip › S19-data/T3-anti-SipB-InvJ-SptP-SipD-SipC.tif]

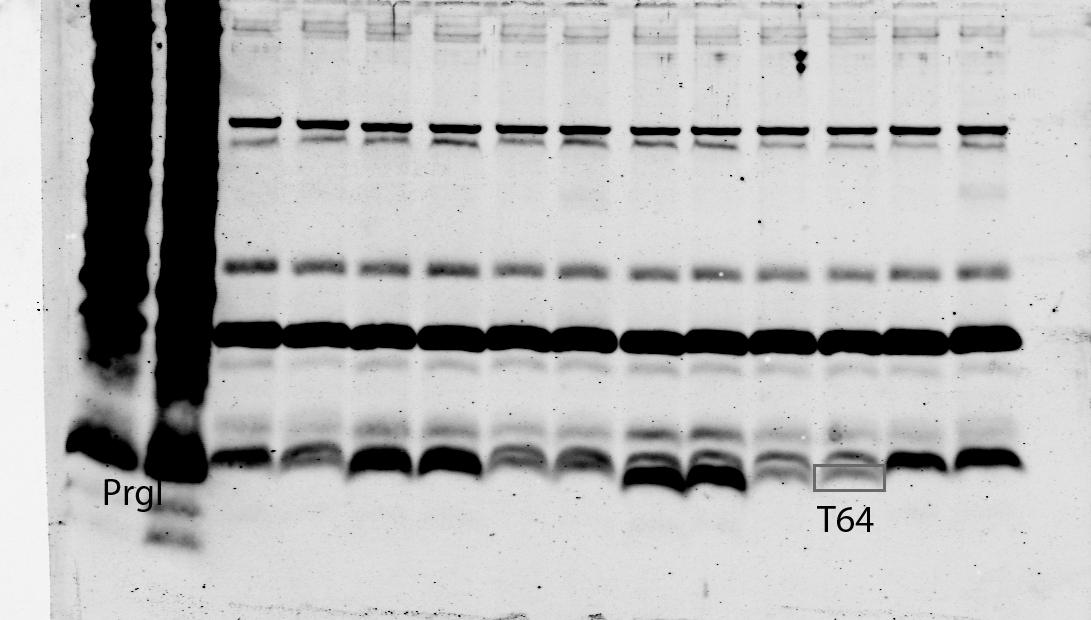

Supplement: S19 Data — (ZIP) [file pbio.3000351.s039.zip › S19-data/T64-anti-PrgI.tif]

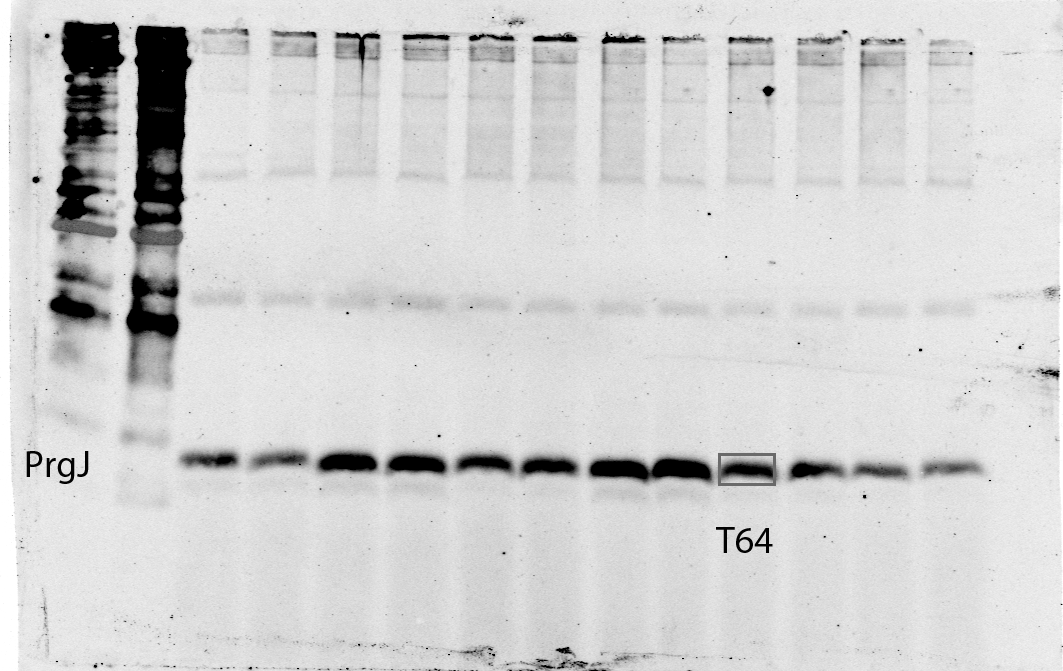

Supplement: S19 Data — (ZIP) [file pbio.3000351.s039.zip › S19-data/T64-anti-PrgJ.tif]

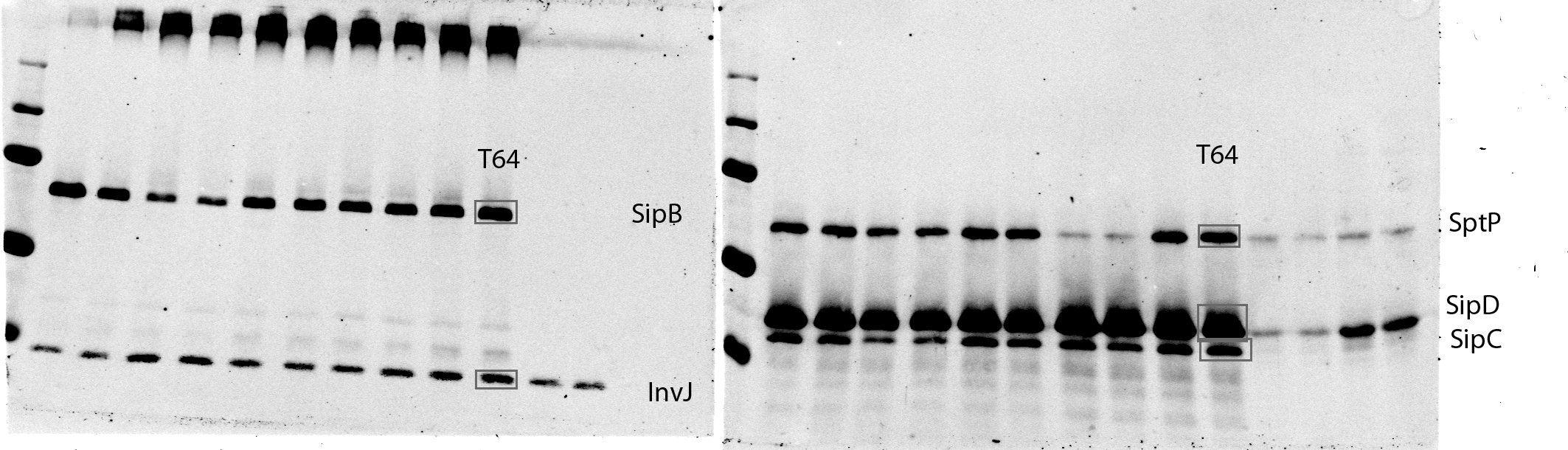

Supplement: S19 Data — (ZIP) [file pbio.3000351.s039.zip › S19-data/T64-anti-SipB-InvJ-SptP-SipD-SipC.tif]

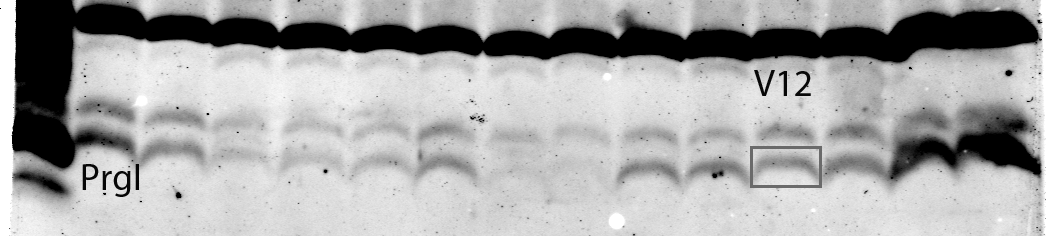

Supplement: S20 Data — (ZIP) [file pbio.3000351.s040.zip › V12-anti-PrgI.tif]

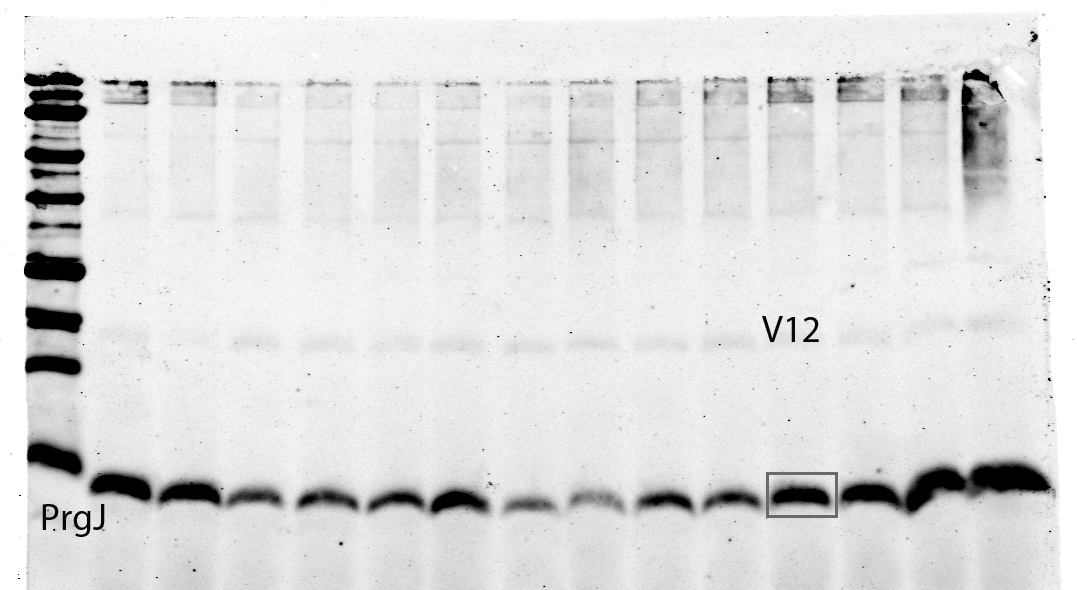

Supplement: S20 Data — (ZIP) [file pbio.3000351.s040.zip › V12-anti-PrgJ.tif]

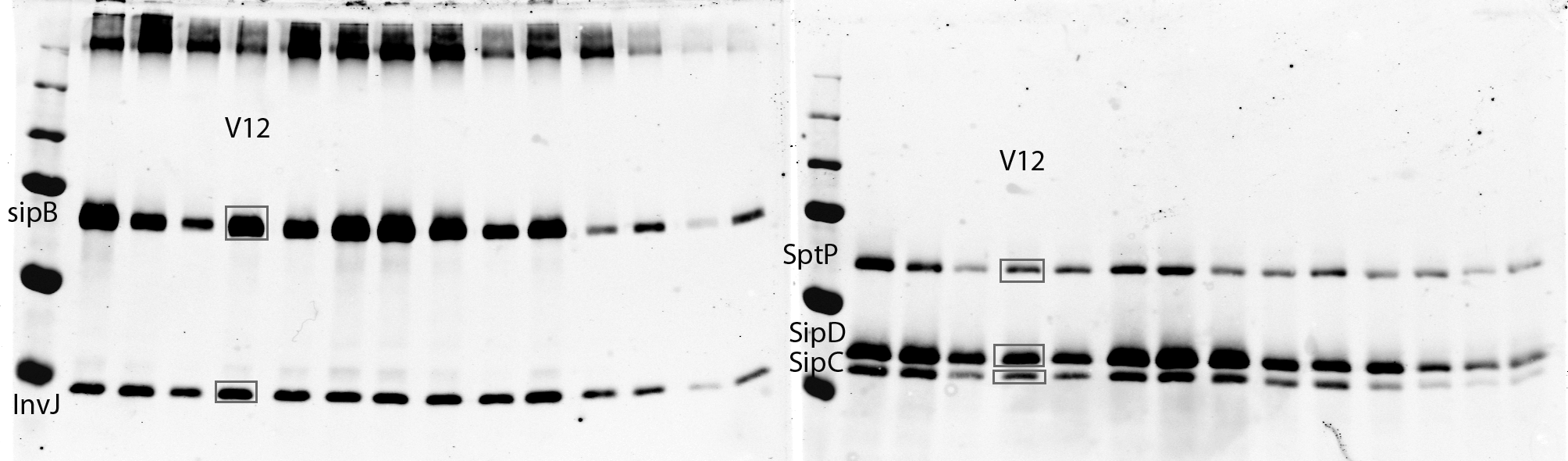

Supplement: S20 Data — (ZIP) [file pbio.3000351.s040.zip › V12-anti-SipB-InvJ-SptP-SipD-SipC.tif]

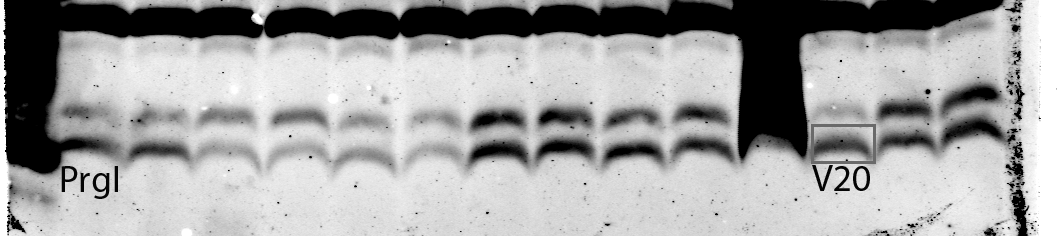

Supplement: S20 Data — (ZIP) [file pbio.3000351.s040.zip › V20-anti-PrgI.tif]

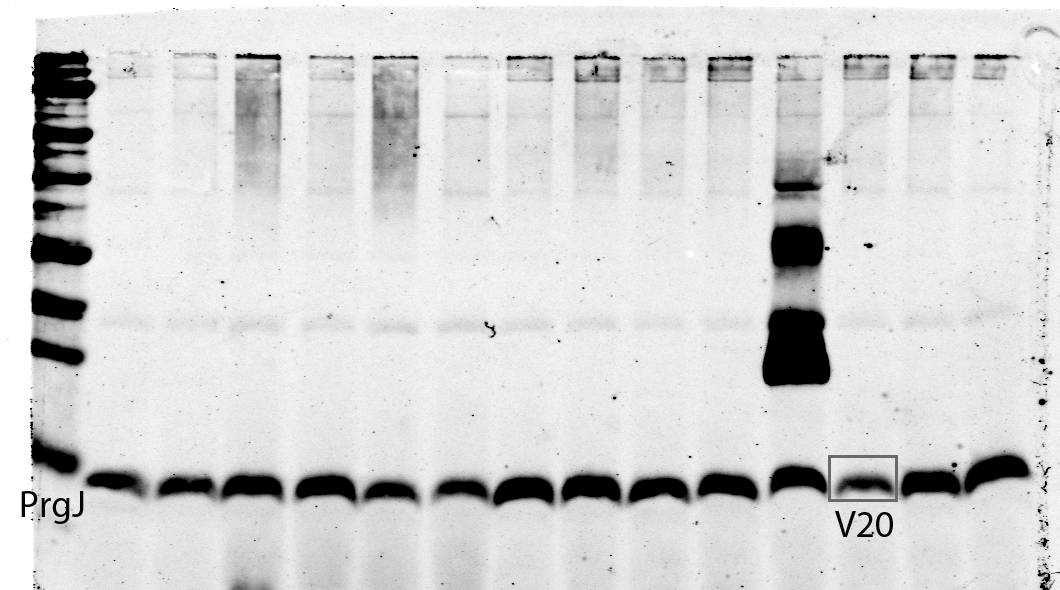

Supplement: S20 Data — (ZIP) [file pbio.3000351.s040.zip › V20-anti-PrgJ.tif]

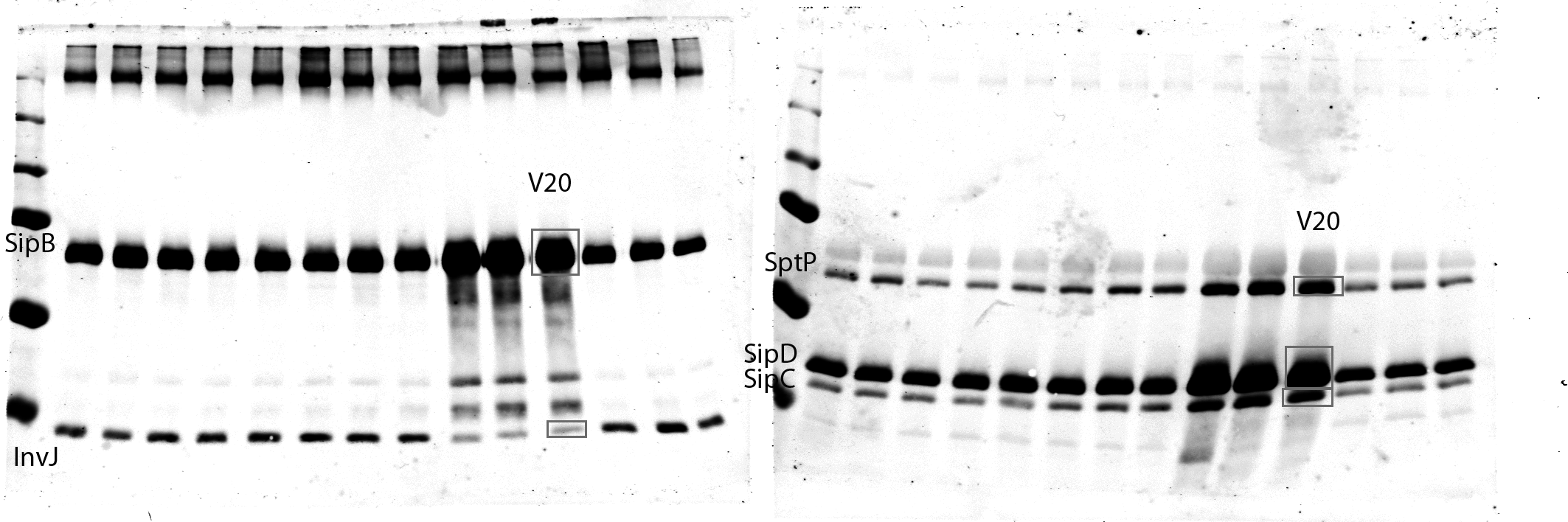

Supplement: S20 Data — (ZIP) [file pbio.3000351.s040.zip › V20-anti-SipB-InvJ-SptP-SipD-SipC.tif]

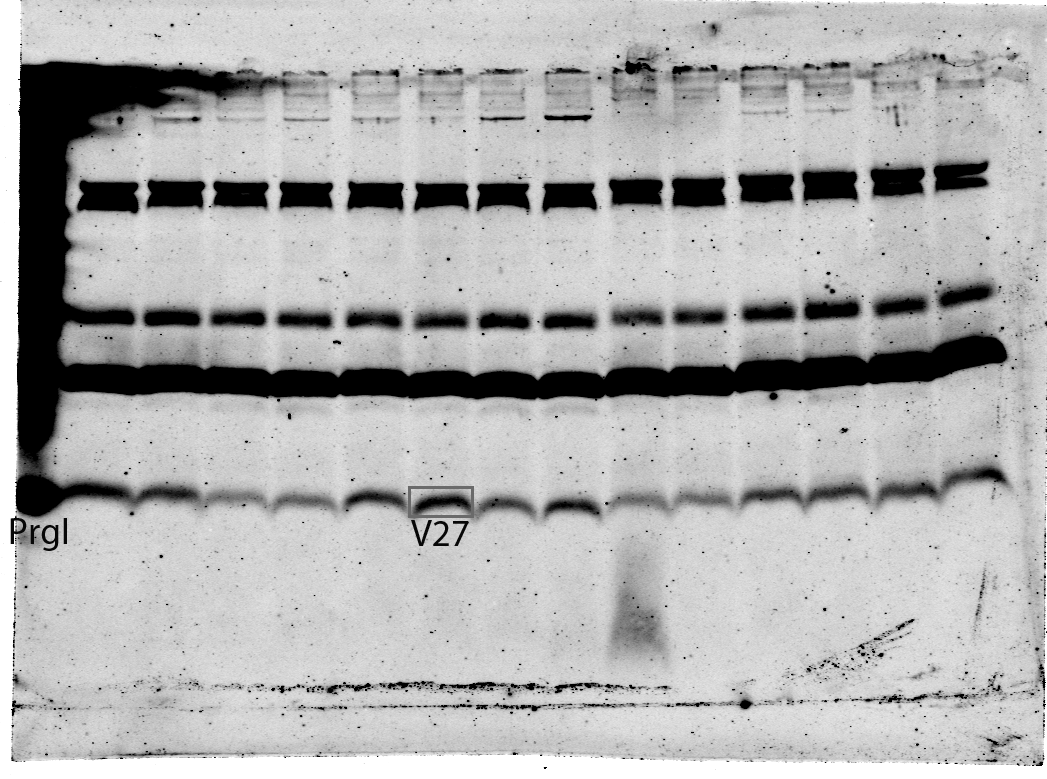

Supplement: S20 Data — (ZIP) [file pbio.3000351.s040.zip › V27-anti-prgI.tif]

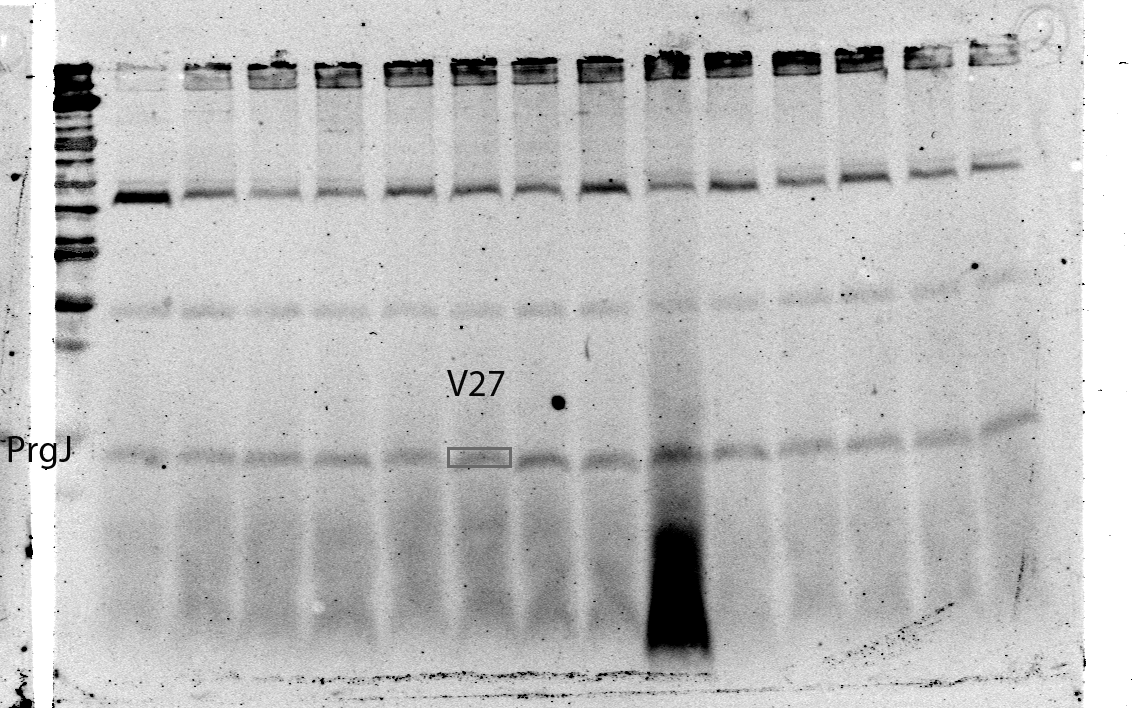

Supplement: S20 Data — (ZIP) [file pbio.3000351.s040.zip › V27-anti-prgJ.tif]

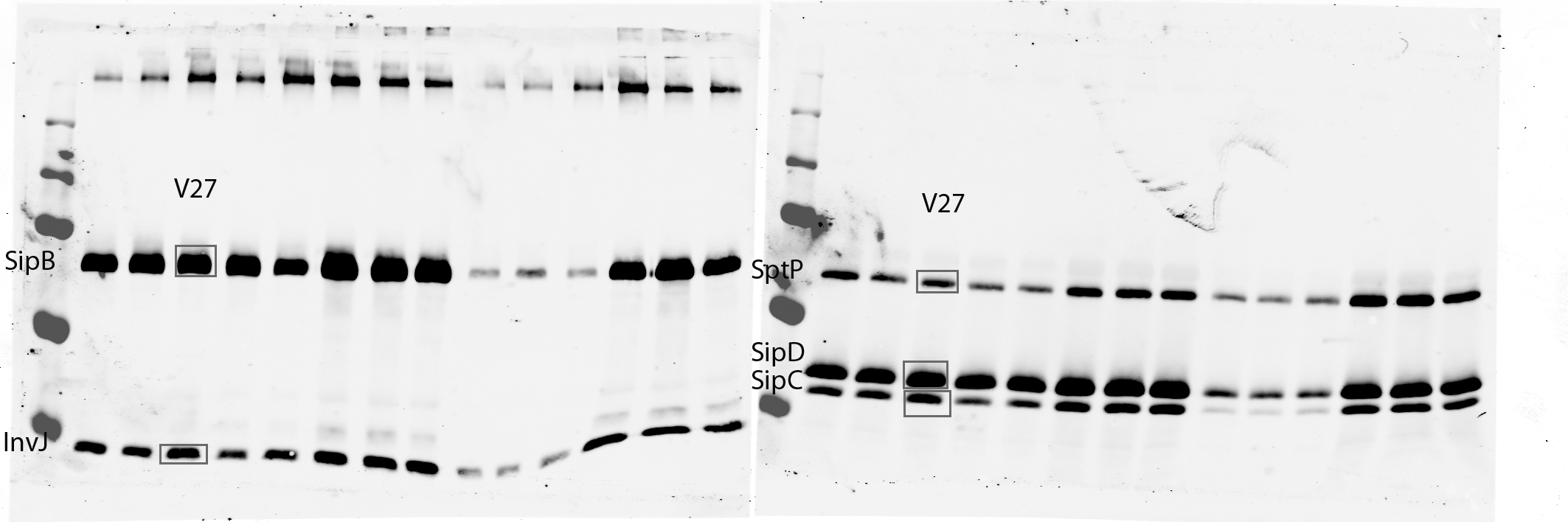

Supplement: S20 Data — (ZIP) [file pbio.3000351.s040.zip › V27-anti-SipB-InvJ-SptP-SipD-SipC.tif]

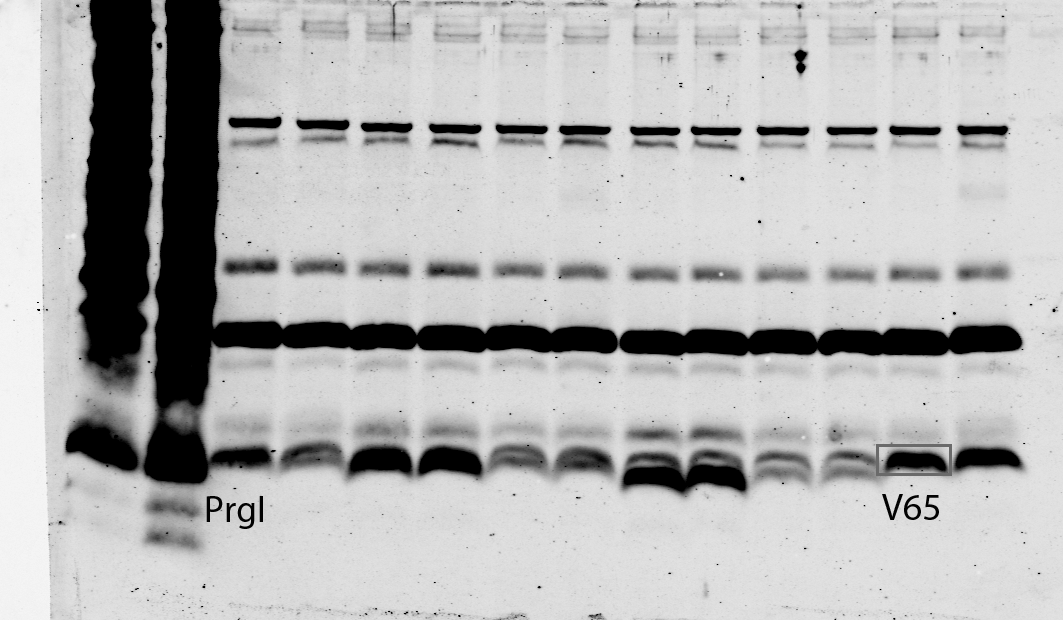

Supplement: S20 Data — (ZIP) [file pbio.3000351.s040.zip › V65-anti-PrgI.tif]

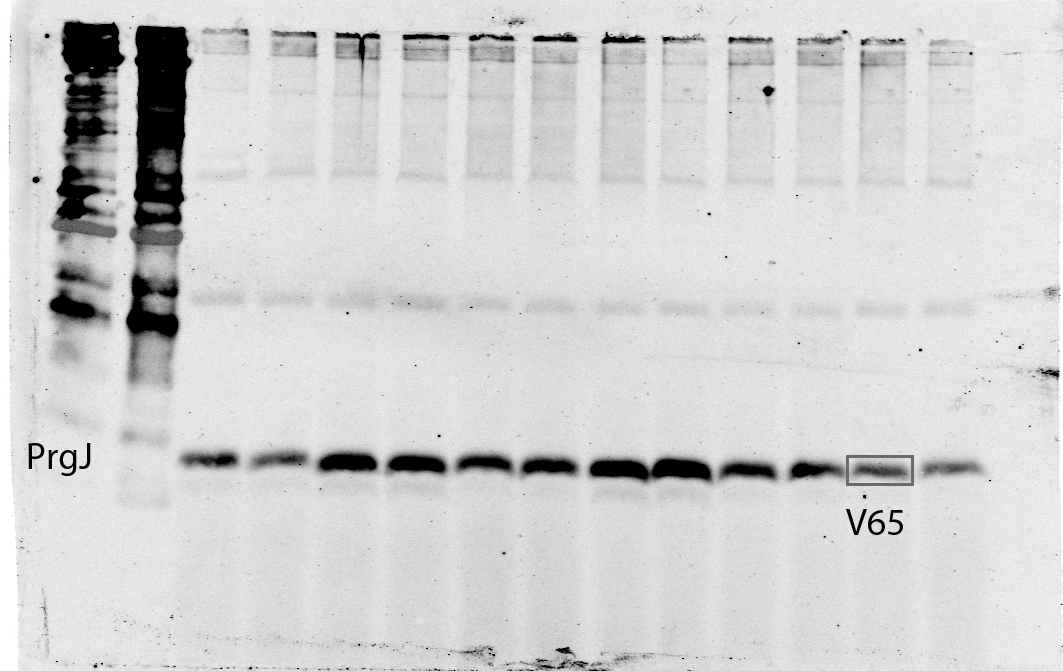

Supplement: S20 Data — (ZIP) [file pbio.3000351.s040.zip › V65-anti-PrgJ.tif]

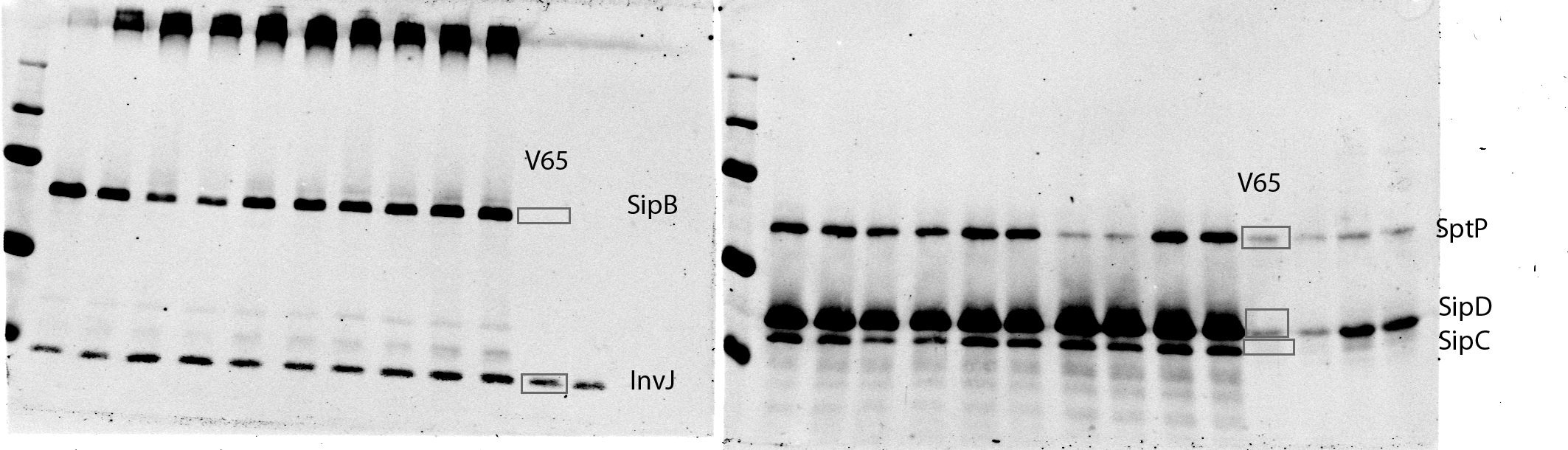

Supplement: S20 Data — (ZIP) [file pbio.3000351.s040.zip › V65-anti-SipB-InvJ-SptP-SipD-SipC.tif]

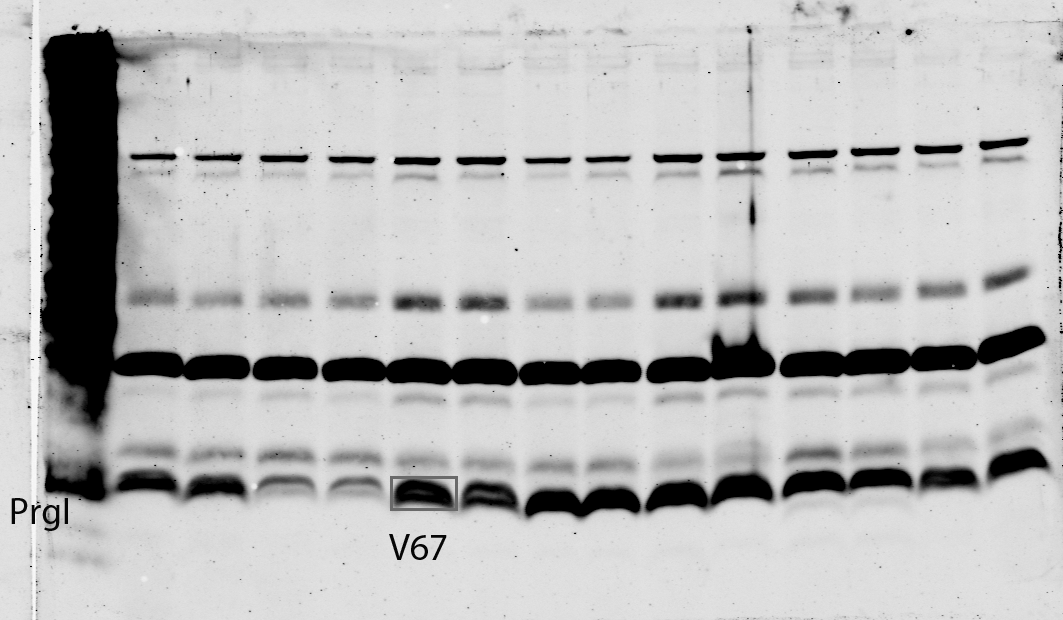

Supplement: S20 Data — (ZIP) [file pbio.3000351.s040.zip › V67-anti-PrgI.tif]

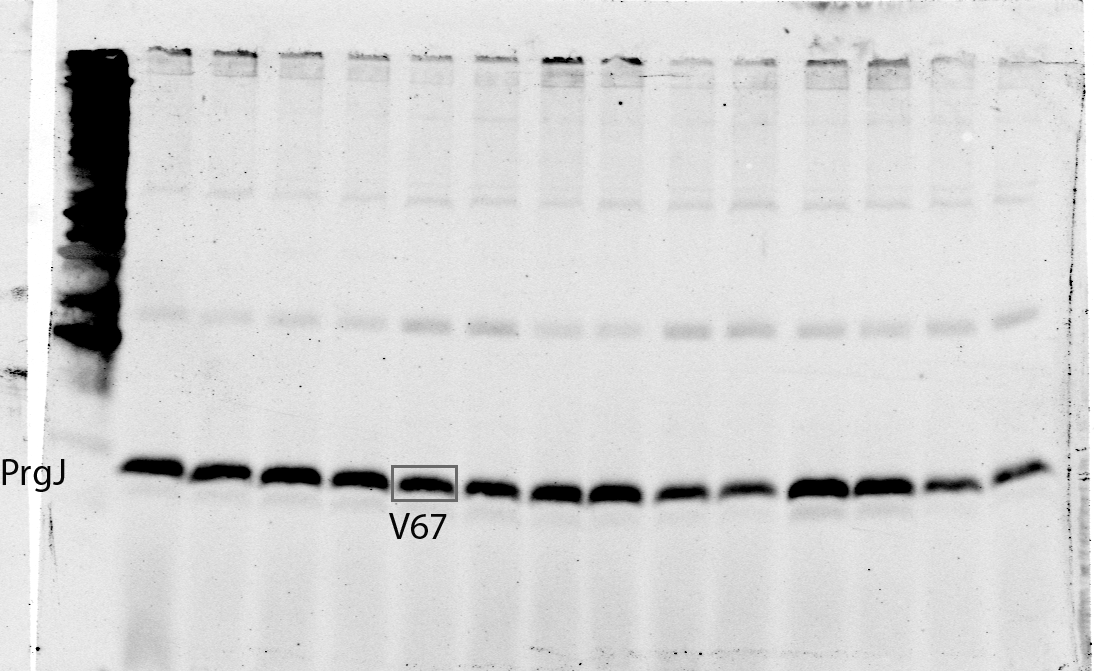

Supplement: S20 Data — (ZIP) [file pbio.3000351.s040.zip › V67-anti-PrgJ.tif]

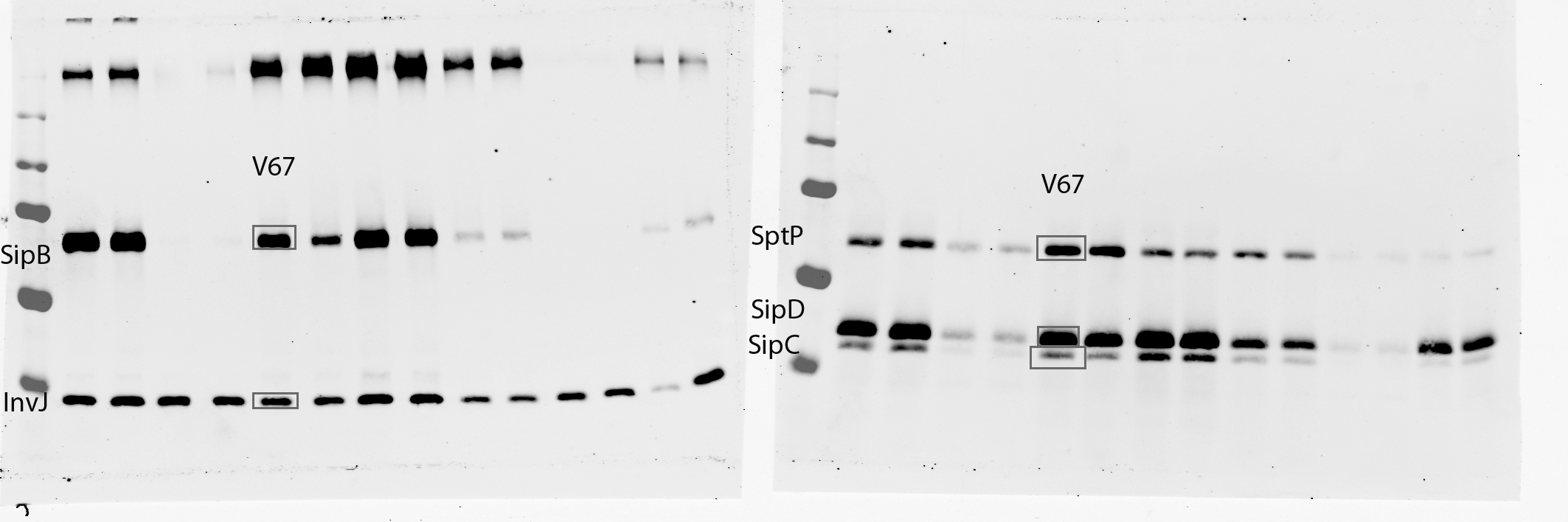

Supplement: S20 Data — (ZIP) [file pbio.3000351.s040.zip › V67-anti-SipB-InvJ-SptP-SipD-SipC.tif]

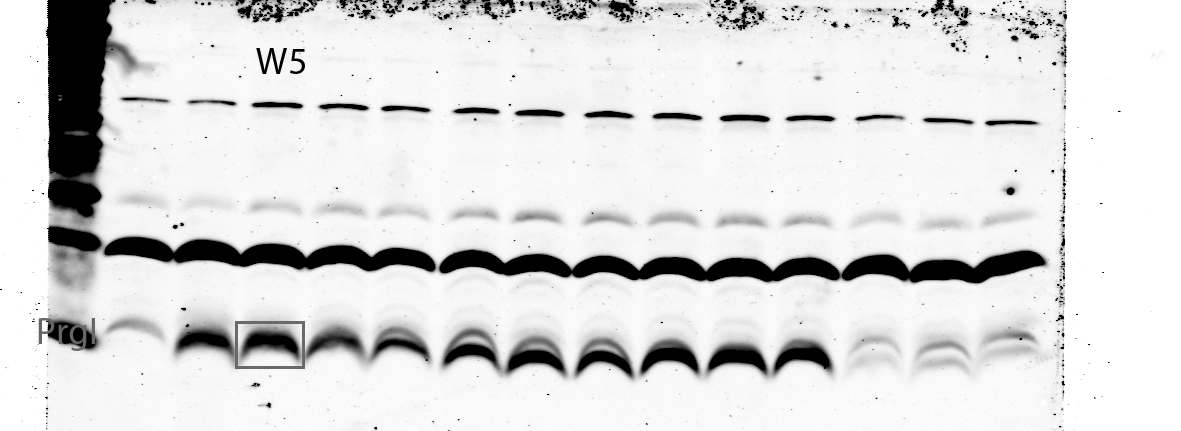

Supplement: S20 Data — (ZIP) [file pbio.3000351.s040.zip › W5-anti-prgI.tif]

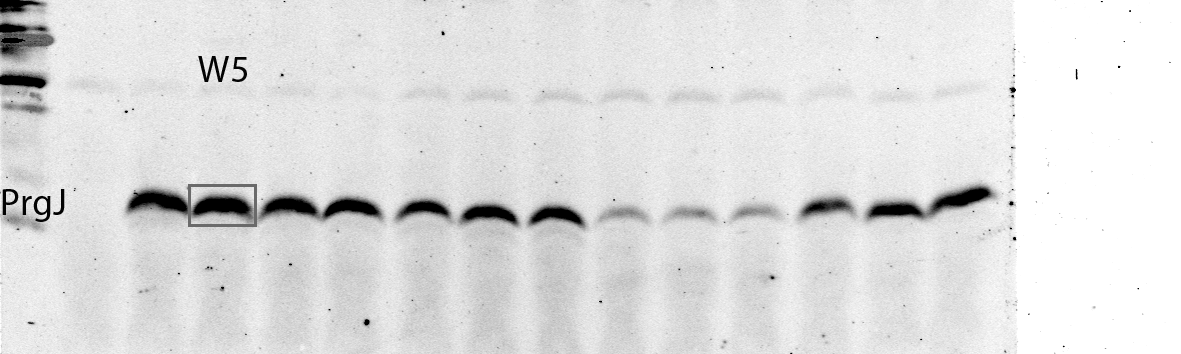

Supplement: S20 Data — (ZIP) [file pbio.3000351.s040.zip › W5-anti-prgJ.tif]

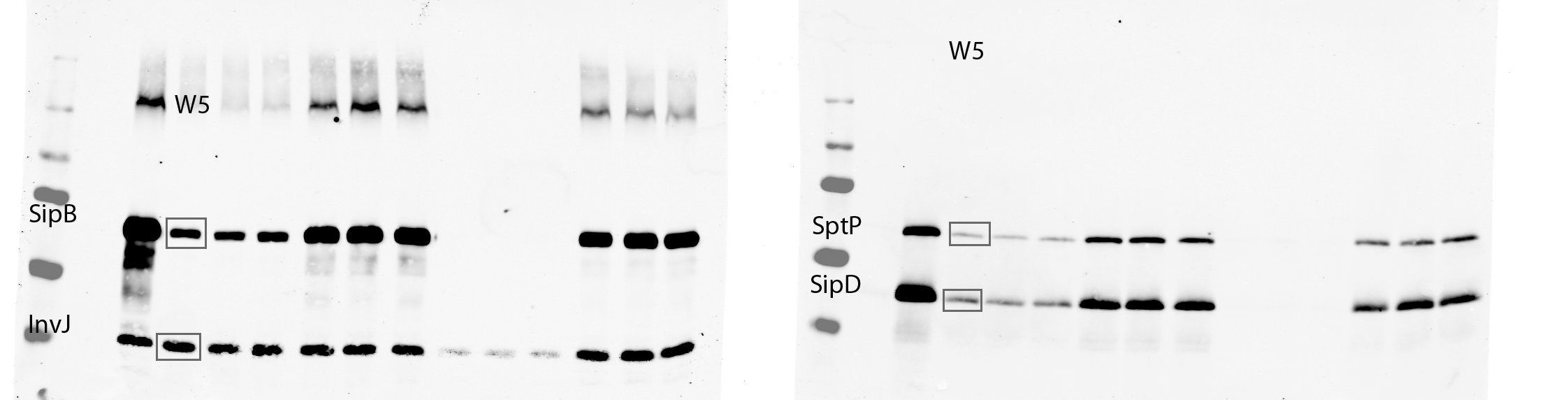

Supplement: S20 Data — (ZIP) [file pbio.3000351.s040.zip › W5-anti-SipB-InvJ-SptP-SipD.tif]

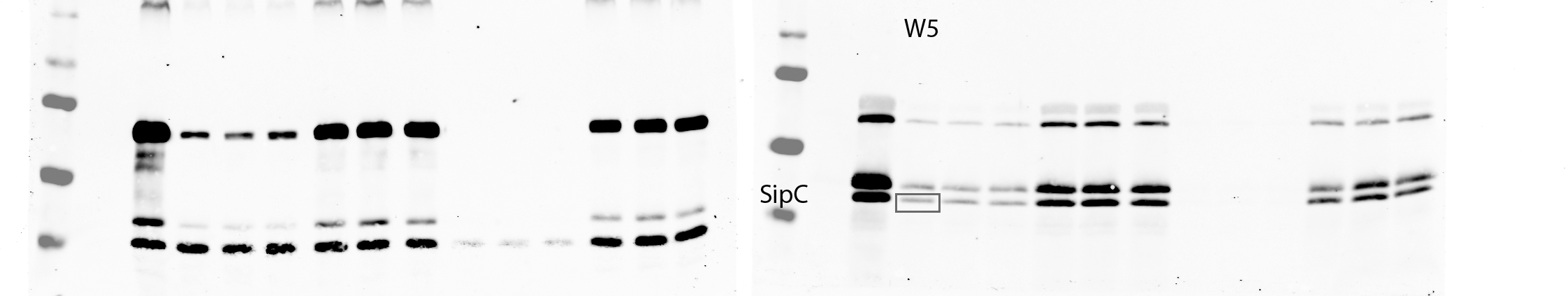

Supplement: S20 Data — (ZIP) [file pbio.3000351.s040.zip › W5-anti-SipC.tif]

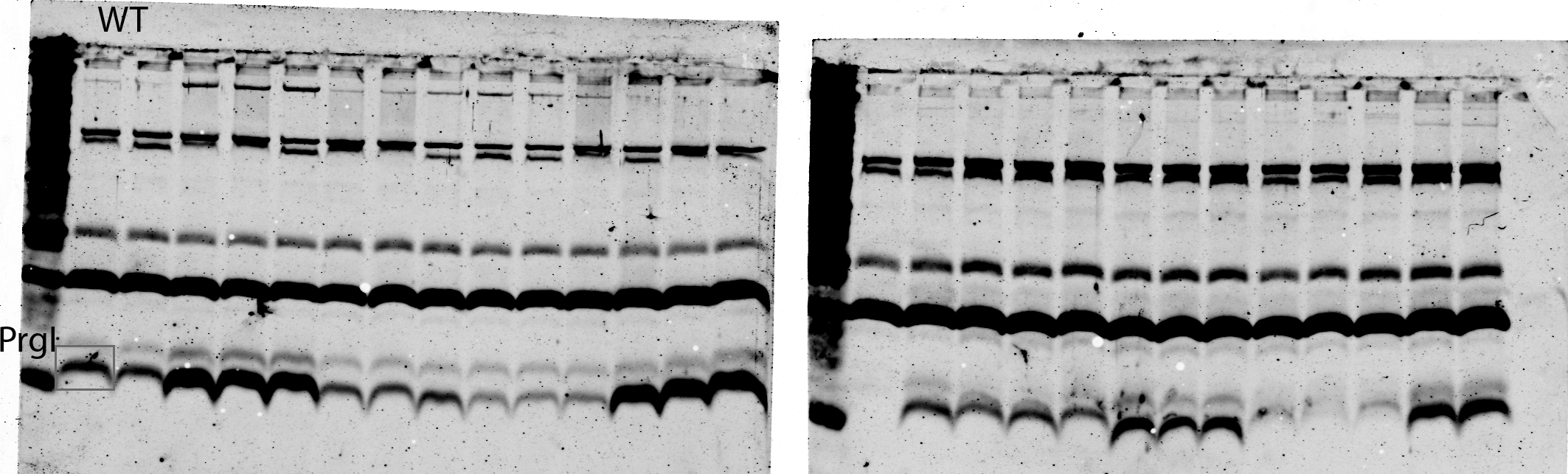

Supplement: S21 Data — (ZIP) [file pbio.3000351.s041.zip › S21-data/WT-anti-prgI.tif]

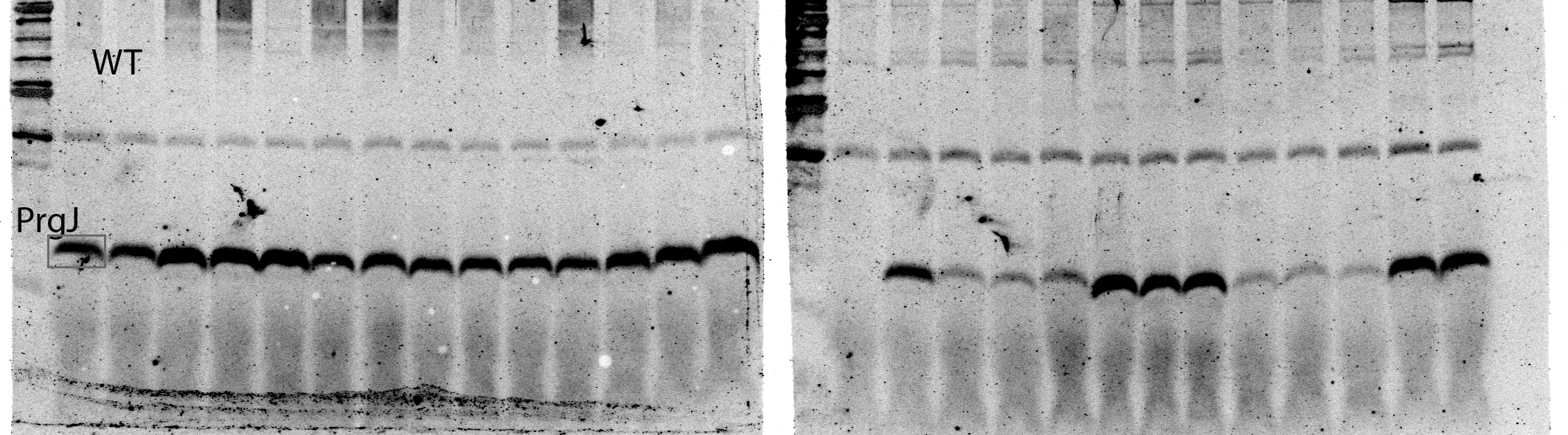

Supplement: S21 Data — (ZIP) [file pbio.3000351.s041.zip › S21-data/WT-anti-prgJ.tif]

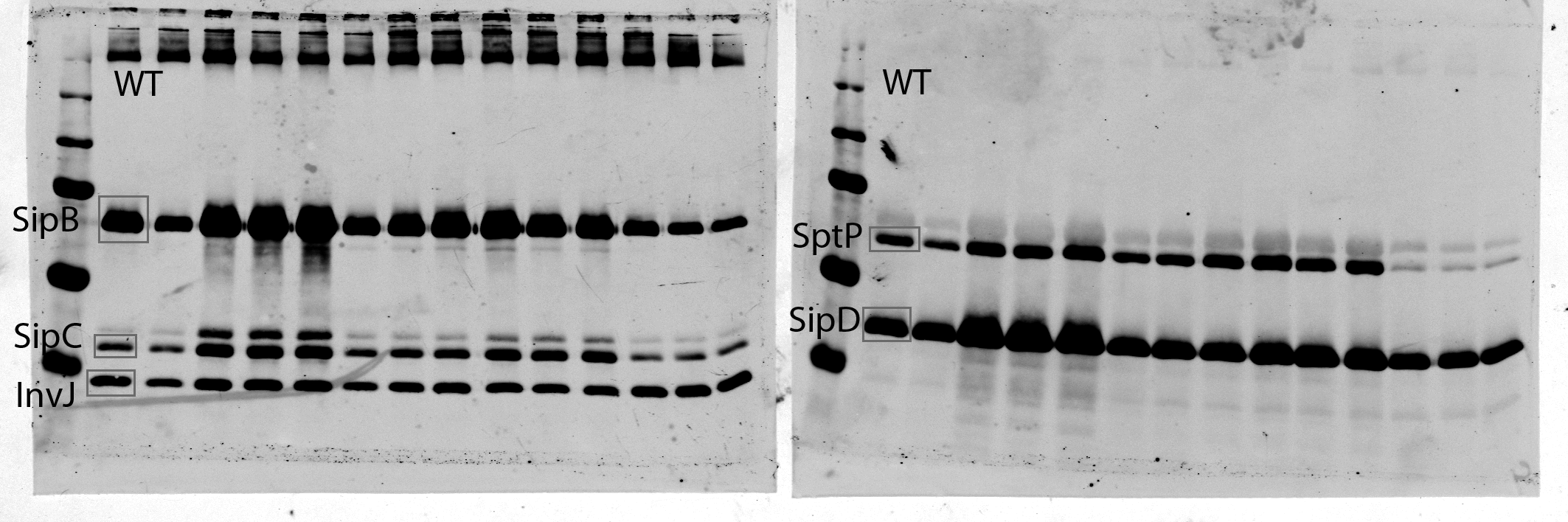

Supplement: S21 Data — (ZIP) [file pbio.3000351.s041.zip › S21-data/WT-anti-SipB-SipC-InvJ-SptP-SipD.tif]

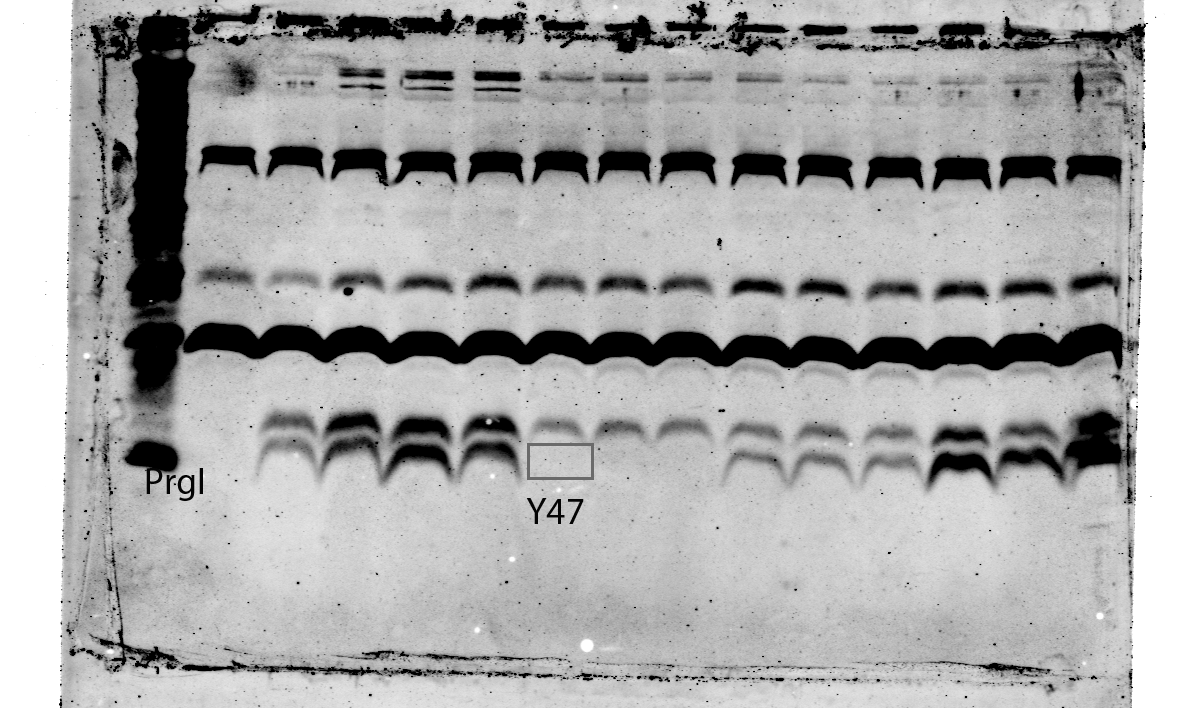

Supplement: S21 Data — (ZIP) [file pbio.3000351.s041.zip › S21-data/Y47-anti-prgI.tif]

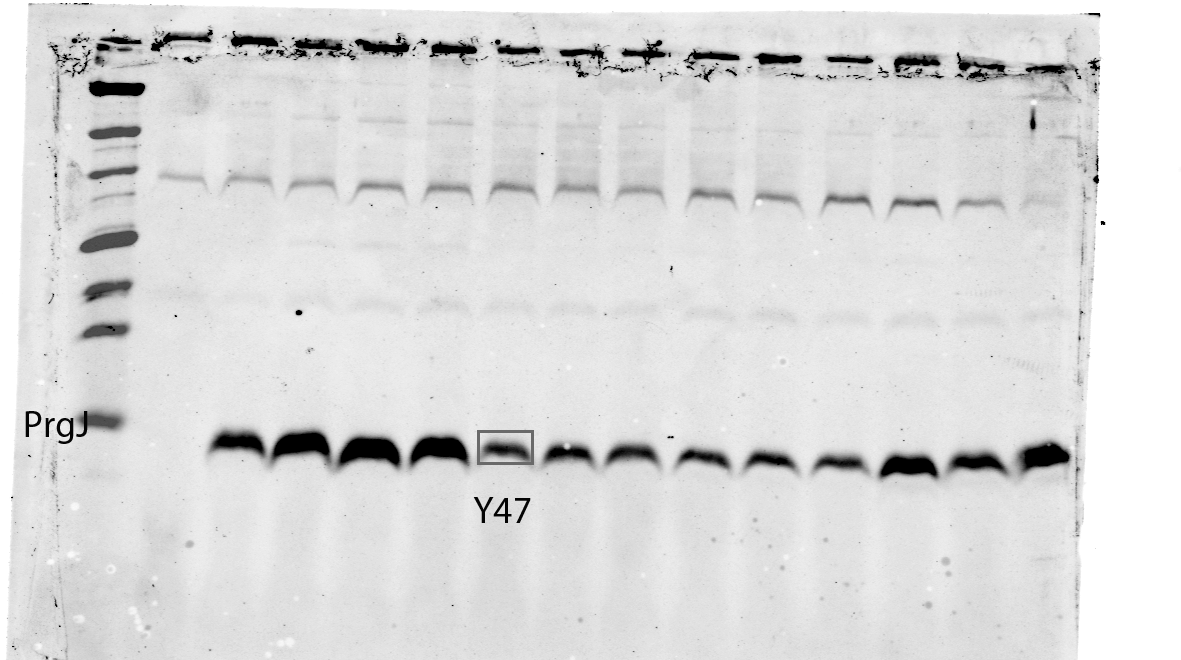

Supplement: S21 Data — (ZIP) [file pbio.3000351.s041.zip › S21-data/Y47-anti-prgJ.tif]

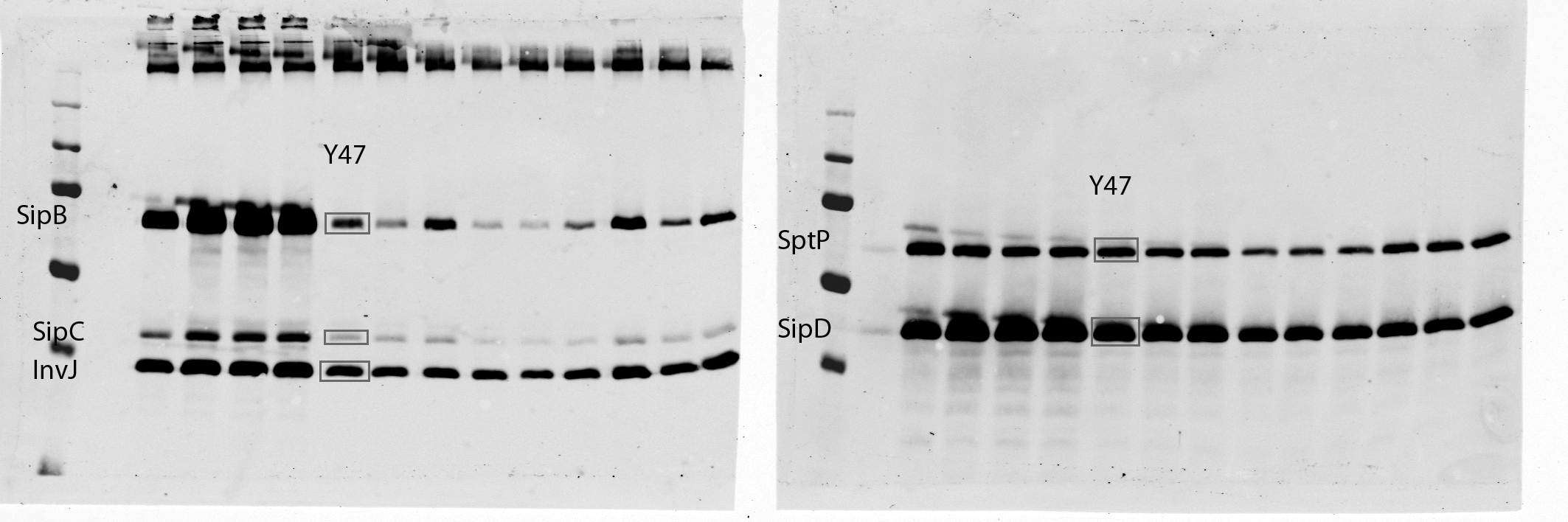

Supplement: S21 Data — (ZIP) [file pbio.3000351.s041.zip › S21-data/Y47-anti-SipB-SipC-InvJ-SptP-SipD.tif]

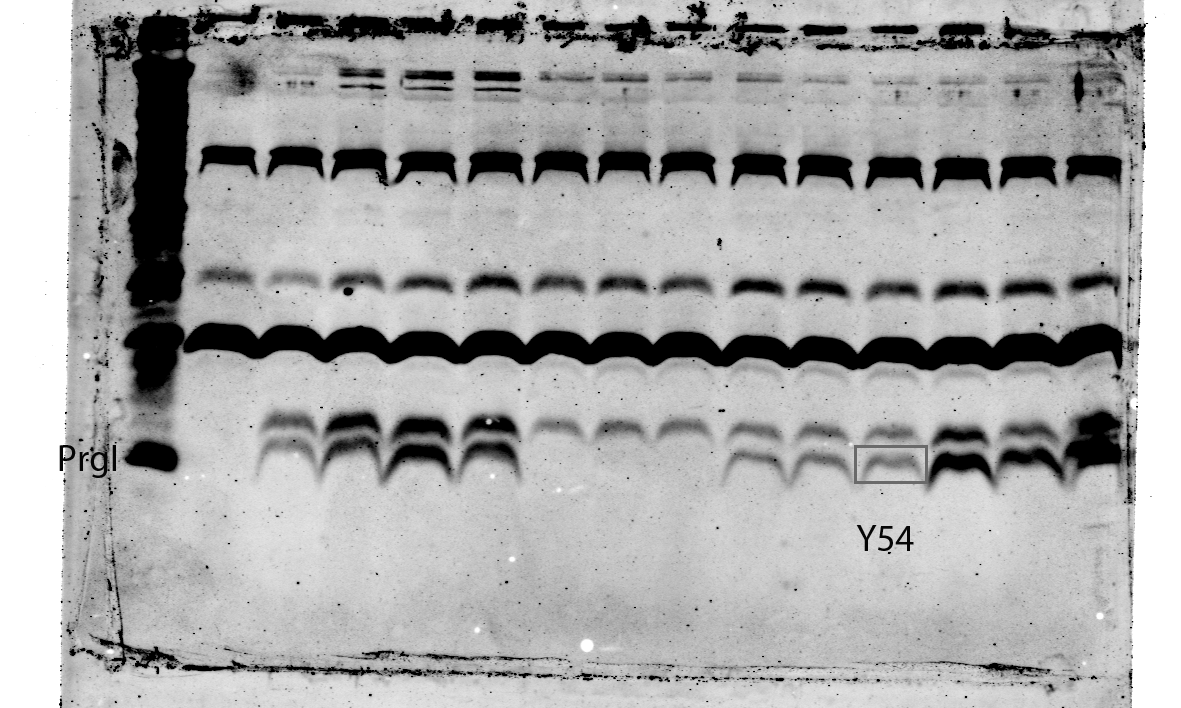

Supplement: S21 Data — (ZIP) [file pbio.3000351.s041.zip › S21-data/Y54-anti-prgI.tif]

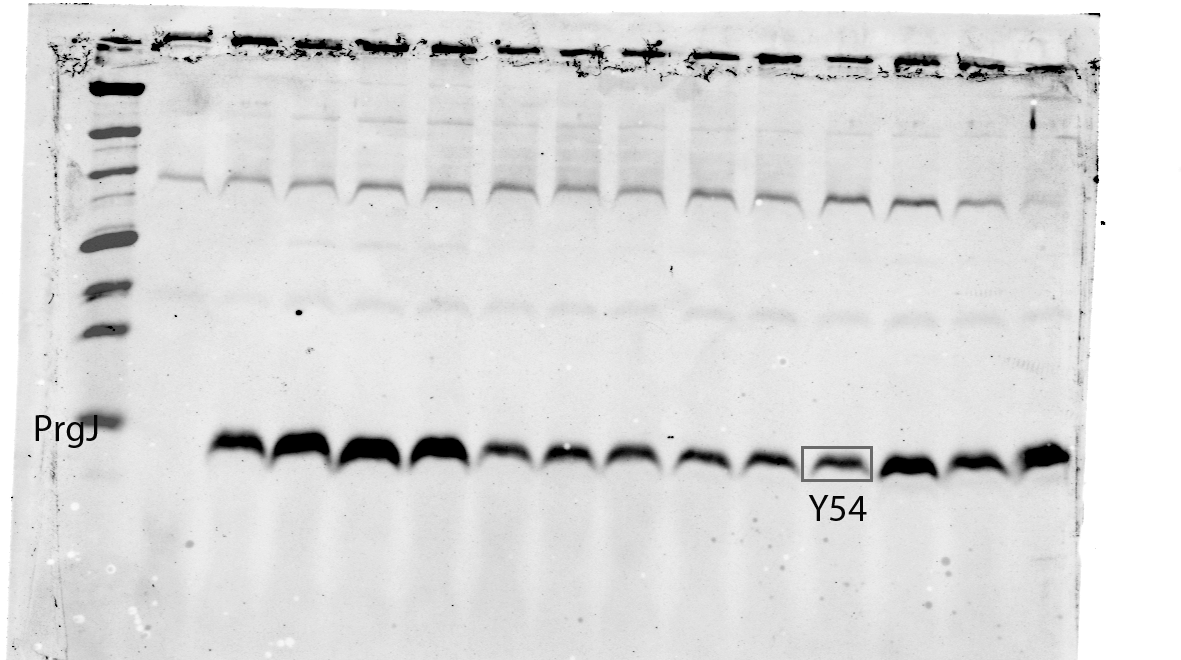

Supplement: S21 Data — (ZIP) [file pbio.3000351.s041.zip › S21-data/Y54-anti-prgJ.tif]

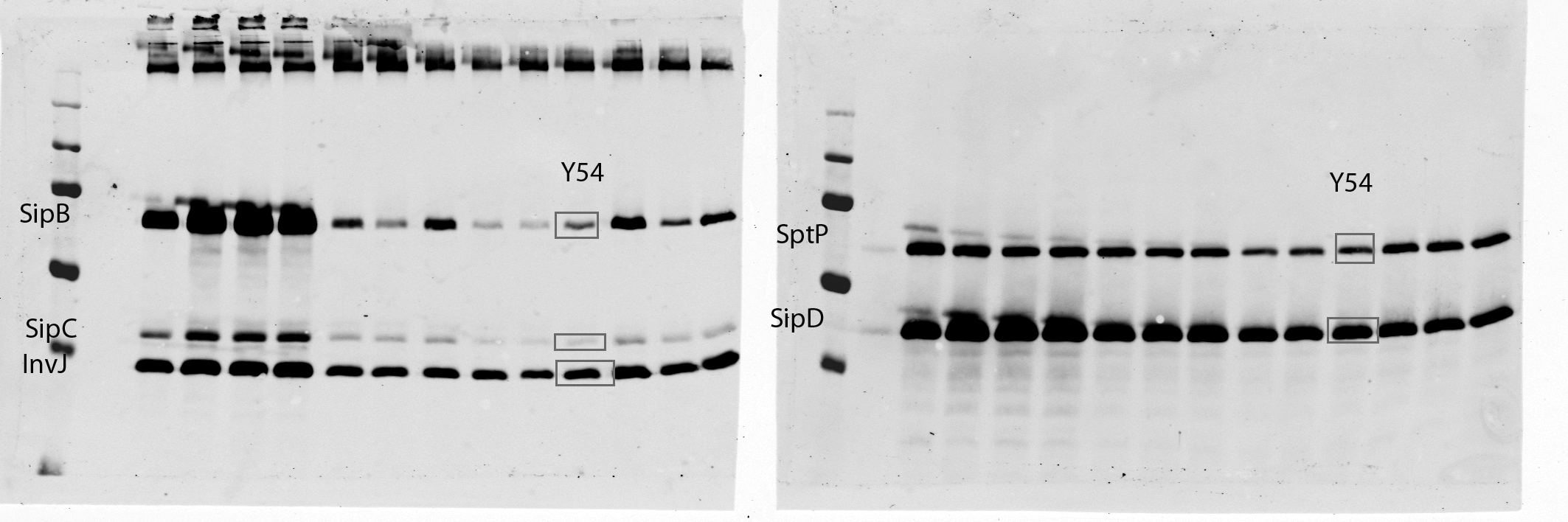

Supplement: S21 Data — (ZIP) [file pbio.3000351.s041.zip › S21-data/Y54-anti-SipB-SipC-InvJ-SptP-SipD.tif]

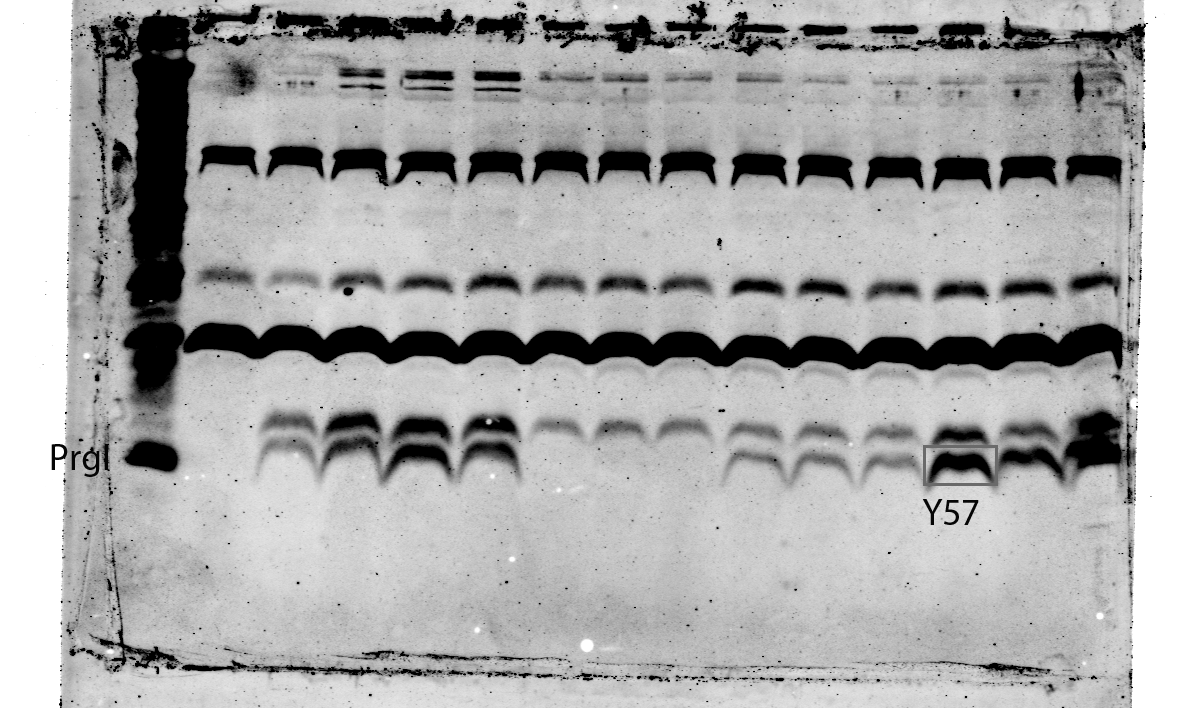

Supplement: S21 Data — (ZIP) [file pbio.3000351.s041.zip › S21-data/Y57-anti-prgI.tif]

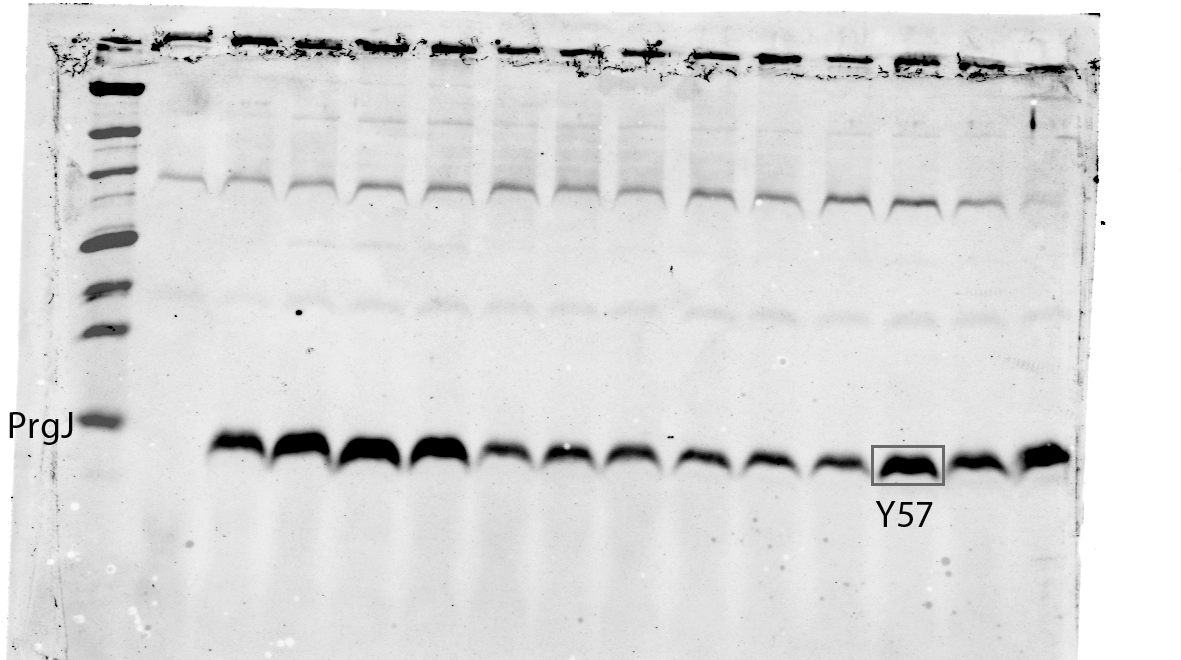

Supplement: S21 Data — (ZIP) [file pbio.3000351.s041.zip › S21-data/Y57-anti-prgJ.tif]

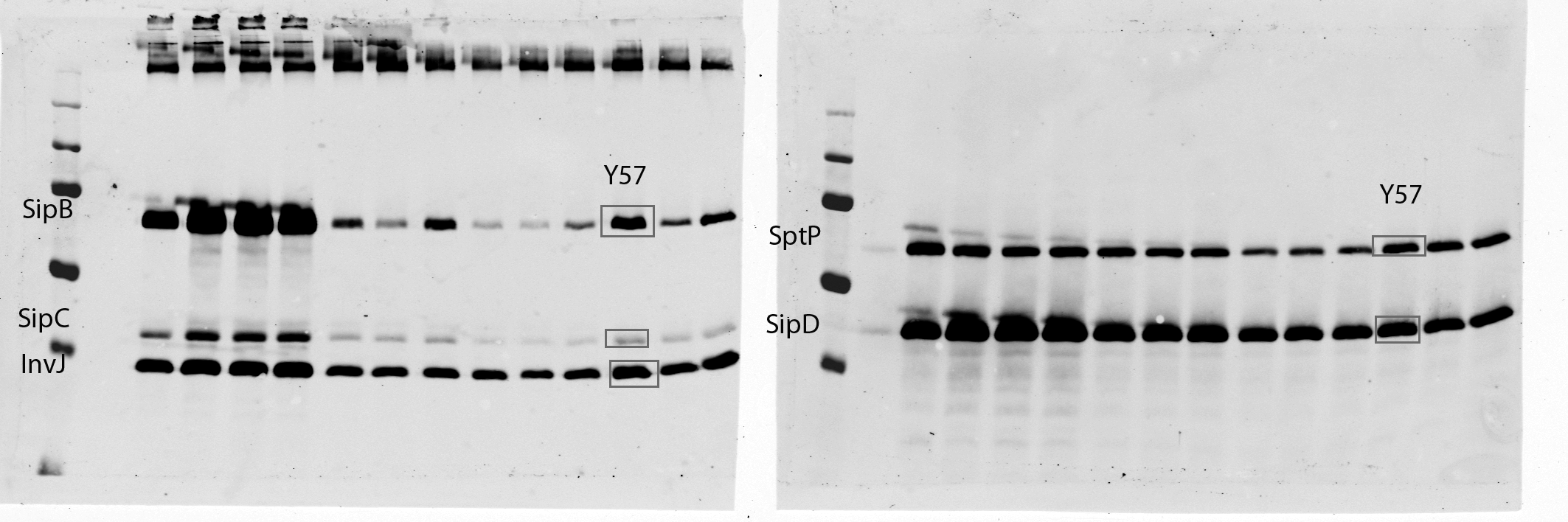

Supplement: S21 Data — (ZIP) [file pbio.3000351.s041.zip › S21-data/Y57-anti-SipB-SipC-InvJ-SptP-SipD.tif]

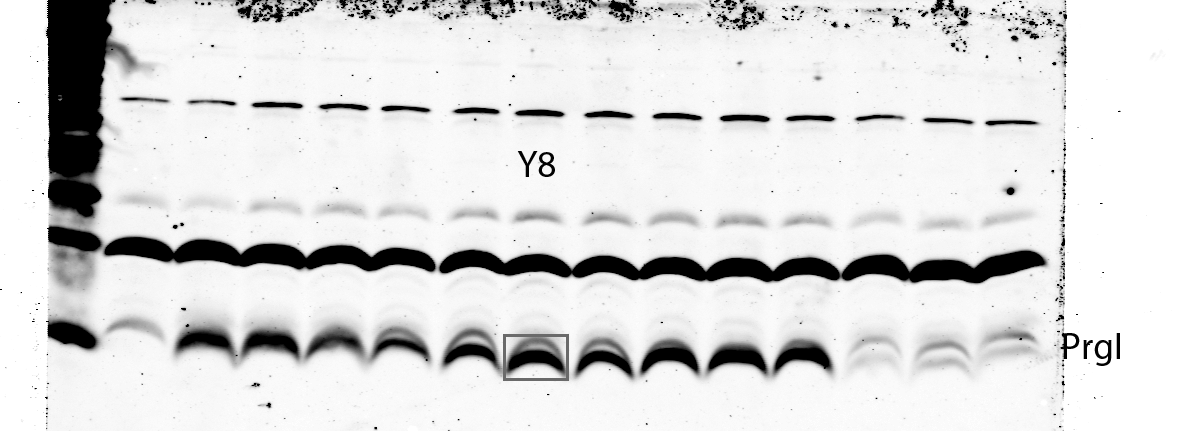

Supplement: S21 Data — (ZIP) [file pbio.3000351.s041.zip › S21-data/Y8-anti-prgI.tif]

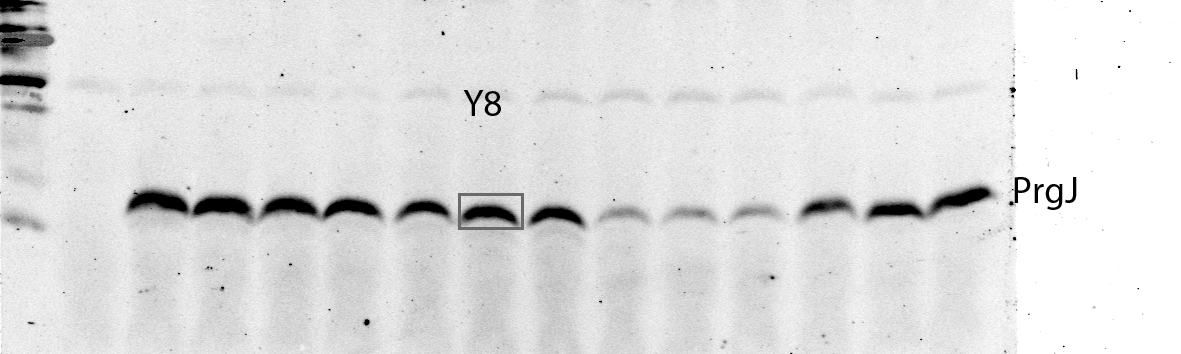

Supplement: S21 Data — (ZIP) [file pbio.3000351.s041.zip › S21-data/Y8-anti-prgJ.tif]

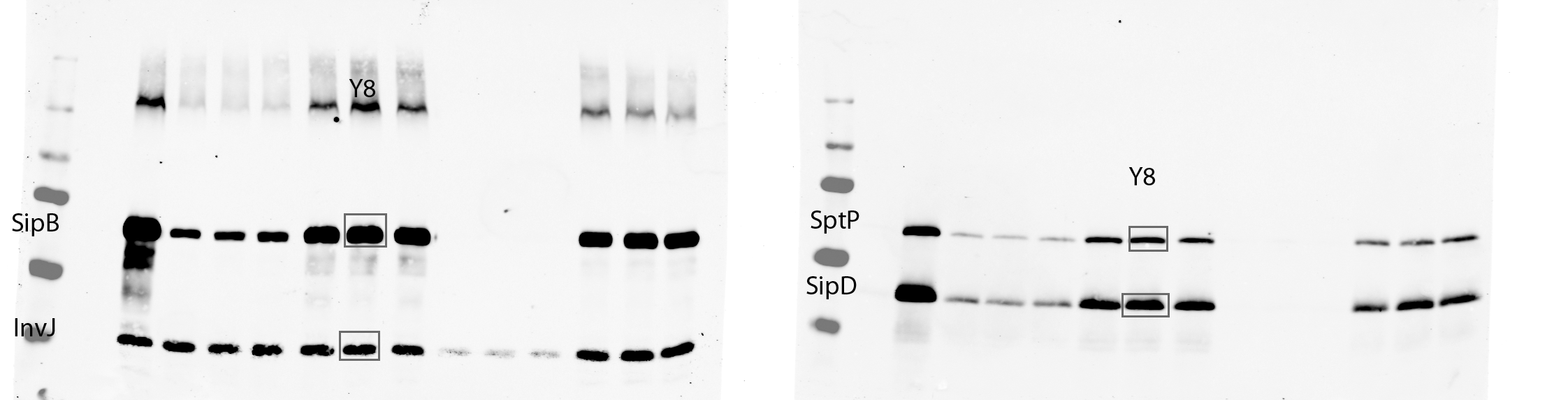

Supplement: S21 Data — (ZIP) [file pbio.3000351.s041.zip › S21-data/Y8-anti-SipB-InvJ-SptP-SipD.tif]
