# Supplementary figures and images for: A polymorphic helix of a Salmonella needle protein relays signals defining distinct steps in type III secretion (part 3 of 3)
Source: PLoS Biol. 2019 Jul 1;17(7):e3000351. doi: 10.1371/journal.pbio.3000351 (PMC6625726; doi:10.1371/journal.pbio.3000351)

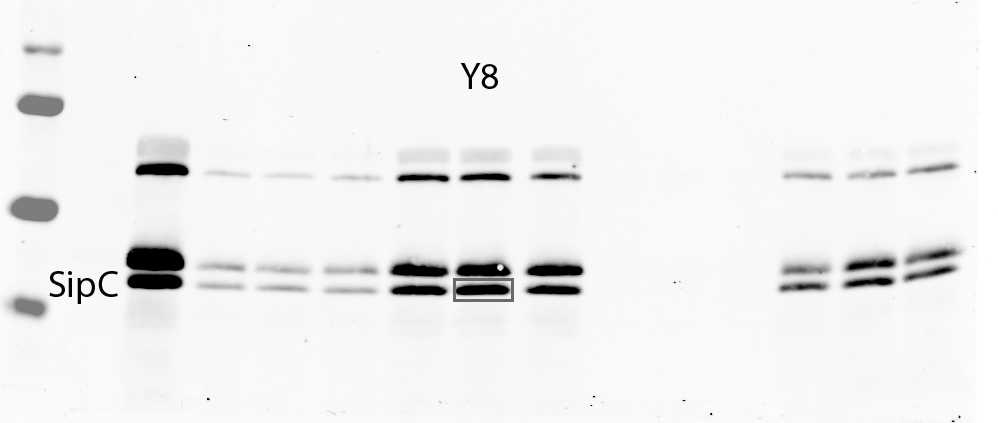

Supplement: S21 Data — (ZIP) [file pbio.3000351.s041.zip › S21-data/Y8-anti-SipC.tif]
