# Supplementary material for: A polymorphic helix of a Salmonella needle protein relays signals defining distinct steps in type III secretion
Source: PLoS Biol. 2019 Jul 1;17(7):e3000351. doi: 10.1371/journal.pbio.3000351 (PMC6625726; doi:10.1371/journal.pbio.3000351)
Supplement: S1 Table — (PDF) [file pbio.3000351.s045.pdf]

Table S1. Strains		
**Strain**	**Genotype**	**Source/Reference**
SB3372	*flhD*::Tn*10 sopBM45 sipD3×F sptP3×F* Δ*prgI* Δ*prgJ*	This study.
SB3373	*sopBM45 sipD3×F sptP3×F* Δ*prgI* Δ*prgJ*	This study.
SB3374	*flhD*::Tn*10 sopBM45 sipD3×F* Δ*prgI* Δ*prgJ*	This study.
SB3411	*flhD*::Tn*10* Δ*gifsy1* Δ*gifsy2* Δ*prgI* Δ*prgJ*	This study.
SB3485	*flhD*::Tn*10 sopBM45* Δ*sipD*	This study.
SB3494	*flhD*::Tn*10 mbp-prgH* Δ*gifsy1* Δ*gifsy2* Δ*prgI* Δ*prgJ*	This study.
