# Supplementary material for: A polymorphic helix of a Salmonella needle protein relays signals defining distinct steps in type III secretion
Source: PLoS Biol. 2019 Jul 1;17(7):e3000351. doi: 10.1371/journal.pbio.3000351 (PMC6625726; doi:10.1371/journal.pbio.3000351)
Supplement: S2 Table — (PDF) [file pbio.3000351.s046.pdf]

Table S2. Plasmids		
**Plasmid Designation**	**Description**	**Source/Reference**
pSB890	R6K suicide plasmid	Constructed by K. Kaniga
pSB3504	Plasmid to express *hilA* in a rhamnose-inducible manner	Wagner et al., and Carleton et al.
pSB3910	pET15b::*prgI* expression vector	Constructed by J. Kato
pSB4053	Suicide plasmid to insert 3×Flag tag at the C-terminus of *sptP*	Button and Galan
pSB4578	pWSK129::*sipD3×F* complementation vector for SB3485	Rathinavelan et al.
pSB4579	pWSK129::*sipD3×F* triple mutant (D320R, V323K, S327R) complementation vector for SB3485	Rathinavelan et al.
pSB4886	R6K suicide plasmid to delete *sipD* and generate SB3485	Constructed by M. Lara-Tejero
pSB5806	pET15b::*prgI* (V65A) expression vector	This Study
pSB5809	pET15b::*prgI* (T3A) expression vector	This Study
pSB5810	pET15b::*prgI* (L9A) expression vector	This Study
pSB5811	pET15b::*prgI* (F16A) expression vector	This Study
pSB5812	pET15b::*prgI* (L31A) expression vector	This Study
pSB5813	pET15b::*prgI* (L56A) expression vector	This Study
pSB5814	pET15b::*prgI* (E53A) expression vector	This Study
pSB5815	pET15b::*prgI* (D70K) expression vector	This Study
pSB5816	pET15b::*prgI* (K66E) expression vector	This Study
pSB5817	pET15b::*prgI* (K66A/R80A) expression vector	This Study
pSB5818	R6K suicide plasmid to insert a MBP tag at the N-terminus of *prgH* to generate SB3494	This Study
pSB5819	R6K suicide plasmid to delete *prgI prgJ*	This Study
pSB5721	pWSK29::*prgI prgJ* (WT) used to complement SB3372	This Study
pSB5722	pWSK29::*prgI prgJ* (S49A)	This Study
pSB5723	pWSK29::*prgI prgJ* (N55A)	This Study
pSB5724	pWSK29::*prgI prgJ* (R58A)	This Study
pSB5725	pWSK29::*prgI prgJ* (D70A/D72A)	This Study
pSB5726	pWSK29::*prgI prgJ* (Q77A)	This Study
pSB5727	pWSK29::*prgI prgJ* (Q77R)	This Study
pSB5728	pWSK29::*prgI prgJ* (Q77K)	This Study
pSB5729	pWSK29::*prgI prgJ* (Q77E)	This Study
pSB5730	pWSK29::*prgI prgJ* (Q77M)	This Study
pSB5731	pWSK29::*prgI prgJ* (P41A)	This Study
pSB5732	pWSK29::*prgI prgJ* (Q48A)	This Study
pSB5733	pWSK29::*prgI prgJ* (D70A)	This Study
pSB5734	pWSK29::*prgI prgJ* (R80K)	This Study
pSB5735	pWSK29::*prgI prgJ* (R80E)	This Study
pSB5736	pWSK29::*prgI prgJ* (R80M)	This Study
pSB5737	pWSK29::*prgI prgJ* (K66A/R80A)	This Study
pSB5738	pWSK29::*prgI prgJ* (K66A)	This Study
pSB5740	pWSK29::*prgI prgJ* (W5A)	This Study
pSB5741	pWSK29::*prgI prgJ* (Y8A)	This Study
pSB5742	pWSK29::*prgI prgJ* (F16A)	This Study
pSB5743	pWSK29::*prgI prgJ* (Y47A)	This Study
pSB5744	pWSK29::*prgI prgJ* (Y54A)	This Study
pSB5745	pWSK29::*prgI prgJ* (Y57A)	This Study
pSB5746	pWSK29::*prgI prgJ* (D72A)	This Study

pSB5747	pWSK29::*prgI prgJ* (F79A)	This Study
pSB5748	pWSK29::*prgI prgJ* (E53A)	This Study
pSB5749	pWSK29::*prgI prgJ* (F68A)	This Study
pSB5750	pWSK29::*prgI prgJ* (I76A)	This Study
pSB5751	pWSK29::*prgI prgJ* (N78A)	This Study
pSB5753	pWSK29::*prgI prgJ* (L31A)	This Study
pSB5754	pWSK29::*prgI prgJ* (D40A)	This Study
pSB5755	pWSK29::*prgI prgJ* (L23A)	This Study
pSB5756	pWSK29::*prgI prgJ* (D32A)	This Study
pSB5757		
pSB5758	pWSK29::*prgI prgJ* (N59A)	This Study
pSB5759	pWSK29::*prgI prgJ* (N63A)	This Study
pSB5760	pWSK29::*prgI prgJ* (K69A)	This Study
pSB5761	pWSK29::*prgI prgJ* (L9A)	This Study
pSB5762	pWSK29::*prgI prgJ* (D10A)	This Study
pSB5763	pWSK29::*prgI prgJ* (D11A)	This Study
pSB5764	pWSK29::*prgI prgJ* (V12A)	This Study
pSB5765	pWSK29::*prgI prgJ* (L34A)	This Study
pSB5766	pWSK29::*prgI prgJ* (K50A)	This Study
pSB5767	pWSK29::*prgI prgJ* (L51A)	This Study
pSB5768	pWSK29::*prgI prgJ* (S52A)	This Study
pSB5769	pWSK29::*prgI prgJ* (P4A)	This Study
pSB5770	pWSK29::*prgI prgJ* (S6A)	This Study
pSB5771	pWSK29::*prgI prgJ* (P38A)	This Study
pSB5772	pWSK29::*prgI prgJ* (S39A)	This Study
pSB5773	pWSK29::*prgI prgJ* (D17A)	This Study
pSB5774	pWSK29::*prgI prgJ* (T18A)	This Study
pSB5775	pWSK29::*prgI prgJ* (V20A)	This Study
pSB5776	pWSK29::*prgI prgJ* (D21A)	This Study
pSB5777	pWSK29::*prgI prgJ* (V27A)	This Study
pSB5778	pWSK29::*prgI prgJ* (T28A)	This Study
pSB5779	pWSK29::*prgI prgJ* (S62A)	This Study
pSB5780	pWSK29::*prgI prgJ* (S13A)	This Study
pSB5781	pWSK29::*prgI prgJ* (N22A)	This Study
pSB5782	pWSK29::*prgI prgJ* (Q24A)	This Study
pSB5783	pWSK29::*prgI prgJ* (T25A)	This Study
pSB5784	pWSK29::*prgI prgJ* (Q26A)	This Study
pSB5785	pWSK29::*prgI prgJ* (G7A)	This Study
pSB5786	pWSK29::*prgI prgJ* (G19A)	This Study
pSB5787	pWSK29::*prgI prgJ* (Q61A)	This Study
pSB5788	pWSK29::*prgI prgJ* (T64A)	This Study
pSB5789	pWSK29::*prgI prgJ* (V65A)	This Study
pSB5790	pWSK29::*prgI prgJ* (V67A)	This Study
pSB5791	pWSK29::*prgI prgJ* (I75A)	This Study
pSB5792	pWSK29::*prgI prgJ* (D70K)	This Study
pSB5793	pWSK29::*prgI prgJ* (R80Q)	This Study
pSB5794	pWSK29::*prgI prgJ* (T3A)	This Study
pSB5795	pWSK29::*prgI prgJ* (K15A)	This Study
pSB5796	pWSK29::*prgI prgJ* (E29A)	This Study
pSB5797	pWSK29::*prgI prgJ* (K33A)	This Study
pSB5798	pWSK29::*prgI prgJ* (K37A)	This Study

pSB5799	pWSK29::*prgI prgJ* (L43A)	This Study
pSB5800	pWSK29::*prgI prgJ* (L44A)	This Study
pSB5801	pWSK29::*prgI prgJ* (Q77R/R80Q)	This Study
pSB5802	pWSK29::*prgI prgJ* (K66Q/Q77K)	This Study
pSB5803	pWSK29::*prgI prgJ* (D70L)	This Study
pSB5804	pWSK29::*prgI prgJ* (D70N)	This Study
pSB5805	pWSK29::*prgI prgJ* (K66E)	This Study
pSB5820	pWSK29::*prgI prgJ* (K66Q)	This Study
pSB5825	pWSK29::*prgI prgJ* (L56A)	This Study
